# Supplementary material for: Supramolecular Kandinsky circles with high antibacterial activity
Source: Nat Commun. 2018 May 8;9:1815. doi: 10.1038/s41467-018-04247-z (PMC5940903; doi:10.1038/s41467-018-04247-z)
Supplement: Supplementary file 1 — Supplementary Information [file 41467_2018_4247_MOESM1_ESM.pdf]

Supramolecular Kandinsky Circles with High Antibacterial Activity

Heng Wang,[1, +] Xiaomin Qian,[1,2, +] Kun Wang,[3] Ma Su,[1] Wei-Wei Haoyang,[4] Xin Jiang,[5] Robert Brzozowski,[6] Ming Wang,[5] Xiang Gao,[7] Yiming Li,[1] Bingqian Xu,[3] Prahathees Eswara,[6] Xin-Qi Hao,[7] Weitao Gong,[2,*] Jun-Li Hou,[4,*] Jianfeng Cai,[1,*] Xiaopeng Li[1,*]

[1]*Department of Chemistry, University of South Florida, Tampa, Florida 33620, United States*

[2]*State Key Laboratory of Fine Chemicals, School of Chemical Engineering, Dalian University of Technology, Dalian, Liaoning 116024, China*

[3]*Single Molecule Study Laboratory, College of Engineering and Nanoscale Science and Engineering Center, University of Georgia, Athens, Georgia 30602, United States*

[4]*Department of Chemistry, Fudan University, Shanghai 200433, China*

[5]*State Key Laboratory of Supramolecular Structure and Materials, College of Chemistry, Jilin University, Changchun, Jilin 130012, China*

[6] *Department of Cell Biology, Microbiology and Molecular Biology, University of South Florida, Tampa, Florida 33620, United States*

[7] *College of Chemistry and Molecular Engineering, Zhengzhou University, Zhengzhou, Henan 450001, China*

[+] These authors contributed equally to this work.

*Correspondence and requests for materials should be addressed to

E-mail: xiaopengli1@usf.edu (X. L.); jianfengcai@usf.edu (J. C.); houjl@fudan.edu.cn (J.-L. H.); wtgong@dlut.edu.cn (W. G.)

Table of Content

1. Supplementary methods.....3
2. Synthesis of ligands **L1−L4** 7
3. Synthesis of the complexes **G1−G4** 27
4. ^{1}H NMR, ^{13}C NMR, 2D COSY NMR, 2D NOESY NMR, and ESI-MS spectra of ligands **L1−L4** 32
5. ^{1}H NMR, ^{13}C NMR, 2D COSY NMR, 2D NOESY NMR, 2D DOSY NMR and ESI-MS spectra of complexes **G1−G4**.....73
6. Experimental and theoretical collision cross sections 90
7. AFM, TEM and STM images 92
8. UV-Vis and florescence spectra of complexes **G2−G4**. 99
9. Fitting Procedure to derive size parameter of **G2−G4** from logD. 100
10. Supplementary References 102

1. Supplementary methods

General Procedures. All reagents were purchased from Sigma-Aldrich, Matrix Scientific, Fisher, Acros, and Alfa Aesar, and were used without further purification. Compounds **1**, **2**, **3**, and **L1** shown in Supplementy Figure 2 were prepared according to reported methods. Column chromatography was conducted using basic Al_2O_3 (Brockman I, activity, 58 Å) or SiO_2 (VWR, 40-60 µm, 60 Å) and the separated products were visualized by UV light. NMR spectra data were recorded on a 400 MHz, 500 MHz Varian NMR spectrometer, and Bruker AVANCEIII500/AVANCEIII 600 NMR spectrometer (13C DEPTQ experiment) in $CDCl_3$, CD_3CN or DMSO-*d*6 with TMS as reference. ESI-MS and TWIM-MS were recorded with a Waters Synapt G2 tandem mass spectrometer, using solutions of 0.01 mg sample in 1 mL of $CHCl_3/CH_3OH$ (1:3, v/v) for ligands or 0.5 mg sample in 1 mL of MeCN/MeOH (3:1, v/v) for complexes.

TWIM-MS. The TWIM-MS data were collected under the following conditions: ESI capillary voltage, 3 kV; sample cone voltage, 30 V; extraction cone voltage, 3.5 V; source temperature 100 ºC; desolvation temperature, 100 ºC; cone gas flow, 10 L/h; desolvation gas flow, 700 L/h (N_2); source gas control, 0 mL/min; trap gas control, 2 mL/min; helium cell gas control, 100 mL/min; ion mobility (IM) cell gas control, 30 mL/min; sample flow rate, 5 µL/min; IM traveling wave height, 25 V; and IM traveling wave velocity, 1000 m/s. Q was set in rf-only mode to transmit all ions produced by ESI into the triwave region for the acquisition of TWIM-MS data.

Collision cross-section calibration. The calibration procedure of Scrivens et al[1] was used to convert the drift time scale of the TWIM-MS experiments to a collision cross-section (CCS) scale. The calibration curve was constructed by plotting the corrected CCSs of the molecular ions of myoglobin[2] against the corrected drift times of the corresponding molecular ions measured in TWIM-MS experiments at the same traveling wave velocity, traveling wave height and ion mobility gas flow settings *viz.*, 1000 m/s, 25 V, and 30 mL/min, respectively.

Molecular modeling. Energy minimization of the macrocycles was conducted with Materials Studio version 4.2, using the Anneal and Geometry Optimization tasks in the Forcite module (Accelrys Software, Inc.). All counterions were omitted. An initially energy-minimized structure was subjected to 100 annealing cycles with initial and mid-cycle temperatures of 300 and 1000 K, respectively, twenty heating ramps per cycle, one thousand dynamic steps per ramp, and one femtosecond per dynamic step. A constant volume/constant energy (NVE) ensemble was used and

the geometry was optimized after each cycle. Geometry optimization used a universal force field with atom-based summation and cubic spline truncation for both the electrostatic and Van der Waals parameters. 100 energy-minimized structures were selected for the calculation of theoretical collision cross-sections using MOBCAL programs.

TEM. The sample mixtures were drop-casted on to a carbon-coated Cu grid (400 mesh, purchased from SPI supplies), and the extra solution was absorbed by filter paper to avoid further aggregation. The TEM images were taken with a FEI Morgagni transmission electron microscope.

AFM. AFM imaging was performed on a Digital Instrument Nanoscope Dimension 3000 system. The sample was prepared by using DMF solution (ca. 1×10^{-4} mol/L), dropped on freshly cleaved mica surface, rinsed with three drops of fresh acetonitrile, and then dried in air. Silicon cantilevers tip with spring constant of around 0.1 N/m was used for the experiments.

STM. The sample was dissolved in DMF at a concentration of 5.0 mg/mL. Solution (5 µL) was dropped on HOPG surface. After 30 seconds, surface was washed slightly with water for three times and totally dried in R.T. in air. The STM images were taken with a Pico Plus SPM system with a PicoScan 3000 Controller. The obtained STM images were processed by WSxM software.[3]

3D deconvolution fluorescence microscopy. A 100 µL aliquot of cultures, of mid-logarithmic phase *Escherichia coli* cells grown in Luria-Bertani medium and *Staphylococcus aureus* cells grown in tryptic soy broth medium, were incubated with synthetic compounds **G2** and **G3** at indicated concentrations for 5 min at room temperature. When indicated, FM4-64 (0.01 mg/mL final concentration) was added to visualize cell membrane immediately prior to imaging. 5 µL was spotted on a glass bottom culture dish (MatTek) and covered with a 1% agarose pad made with distilled water. Cells were viewed at room temperature with a DeltaVision Elite microscope system (Applied Precision/GE Healthcare) equipped with a Photometrics CoolSnap HQ2 camera and an environmental chamber. Fluorescence of FM4-64 and the synthetic compounds **G2** and **G3** were captured with TRITC and DAPI filters respectively. Fluorescence of supramolecule G4 was captured using TRITC filter in the absence of FM4-64. Seventeen planes were acquired every 200 nm, and the data were deconvolved using manufacturer-provided SoftWorx software.

Minimum inhibitory concentrations (MICs) against MRSA.[4] The antimicrobial activity of the compounds are tested against Methicillin-resistant *S. aureus* (MRSA, ATCC 33591). Briefly, a single colony of MRSA bacterium was inoculated in 3 mL TSB medium and allowed to grow overnight at 37 °C. The bacteria culture was then diluted at 1:100 and the bacteria were able to re-grow to mid-logarithmic phase in 6-8 h. Next, 50 μL compounds in 2-fold serially diluted solution with the concentrations of 0.1-25 μg/mL were added the 96-well plate containing 50 μL of bacteria suspension (1×10^6 CFU/mL) in each well. Following that, the plate was incubated at 37 °C for 16 h, and the absorption of those wells at 600 nm wavelength was read on a Biotek Synergy H1 microtiter plate reader. The MICs were determined as the lowest concentrations that completely inhibit the growth of MRSA in 24 h. The experiment were repeated at least three times with duplicates each time.

Hemolytic assay.[4] The freshly drawn human red blood cells (hRBCs) were washed with 1× PBS buffer multiple times and centrifuged at 3500 rpm for 10 min, until the supernatant became clear. Next, the supernatant was removed, and the RBCs were diluted into 5% v/v suspension. The aliquots of suspension (50 μL) were then incubated with compounds at various concentrations in 2-fold serially diluted solution (50 μL) at 37 °C for 1 h. The mixture was centrifuged at 3500 rpm for 10 min and the supernatant was collected. Following that, 100 μL PBS was added to 30 μL of the supernatant, and the absorbance of the solution at 540 nm was read on a Biotek Synergy H1 plate reader. 2% Triton X-100 was used as the positive control and 1× PBS was used the negative control. The hemolysis activity was calculated by the formula % hemolysis = $(Abs_{sample}-Abs_{PBS})/(Abs_{Triton}-Abs_{PBS})\times 100\%$. The experiment was repeated at least three times with duplicates each time.

Conductance measurement on the planar lipid bilayer. The lipids 1,2-dipalmitoyl-sn-glycero-3-phosphatidylglycerol (DPPG, Avanti Polar Lipids) and 1,2-dipalmitoyl-sn-glycero-3-phosphatidylethanolamine (DPPE, Avanti Polar Lipids) were mixed in chloroform (9:1 in molar ratio, 10 mg/mL, 20 μL). The mixture was evaporated with nitrogen gas to form a thin film and re-dissolved in n-decane (5 μL). The lipid solution (0.5 μL) was injected on to the aperture (diameter = 200 μm) of the Delrin® cup (Warner Instruments, Hamden, CT) and then evaporated with nitrogen gas. The chamber (*cis* side) and the Delrin cup (*trans* side) were filled with aqueous KCl solution (1.0 M, 1.0 mL). Ag-AgCl electrodes were applied directly to the two solutions and the *cis* one was grounded. Planar lipid bilayer was formed by painting the lipids

solution (1.0 μL) around the pretreated aperture and by judgment of capacitance (80-120 pF). The solution of the test compounds in DMSO was added to the *cis* chamber and the solution was stirred for 5 min. Membrane currents were measured using a Warner BC-535D bilayer clamp amplifier and were collected by PatchMaster (HEKA) with sample interval at 5 kHz and then filtered with a 8-pole Bessel filter at 1 kHz (HEKA). The data were analyzed by FitMaster (HEKA) with a digital filter at 100 Hz.

Ultrathin sectioning TEM experiment.[5] MRSA (obtained through the same procedure described in MICs experiment) was incubated with **G2** or **G3** (25 nM) for 45 min. After centrifugation, the bacteria was washed with buffer (100 mM Tris, pH 7.5) for twice. The bacteria was fixed in 2% glutaraldehyde for 30 min, washed twice with double-distilled water, incubated with 2% uranyl acetate for 5 min, washed twice with double-distilled water again, and stained with 2% osmium tetroxide. After centrifugation, the bacteria was dehydrated by mixing with acetone for 5 min and subsequently incubated with 1:1 acetone/epoxy and pure epoxy resin. For preparation of the epoxy resin, solution A (38% Epon 812/62% dodecyl succinic anhydride), solution B (47% Epon 812/53% methylnadic anhydride) and polymerization accelerator, tris-2,3,6-(dimethylaminomethyl)phenol (DMP30), were mixed at a ratio of 30:70:1.5, and then heated at 75 °C for 18 h. The bacteria-embedded resin was cut to 50 nm ultrathin sections and mounted on copper grids for TEM observation.

2. Synthesis of ligands L1−L4

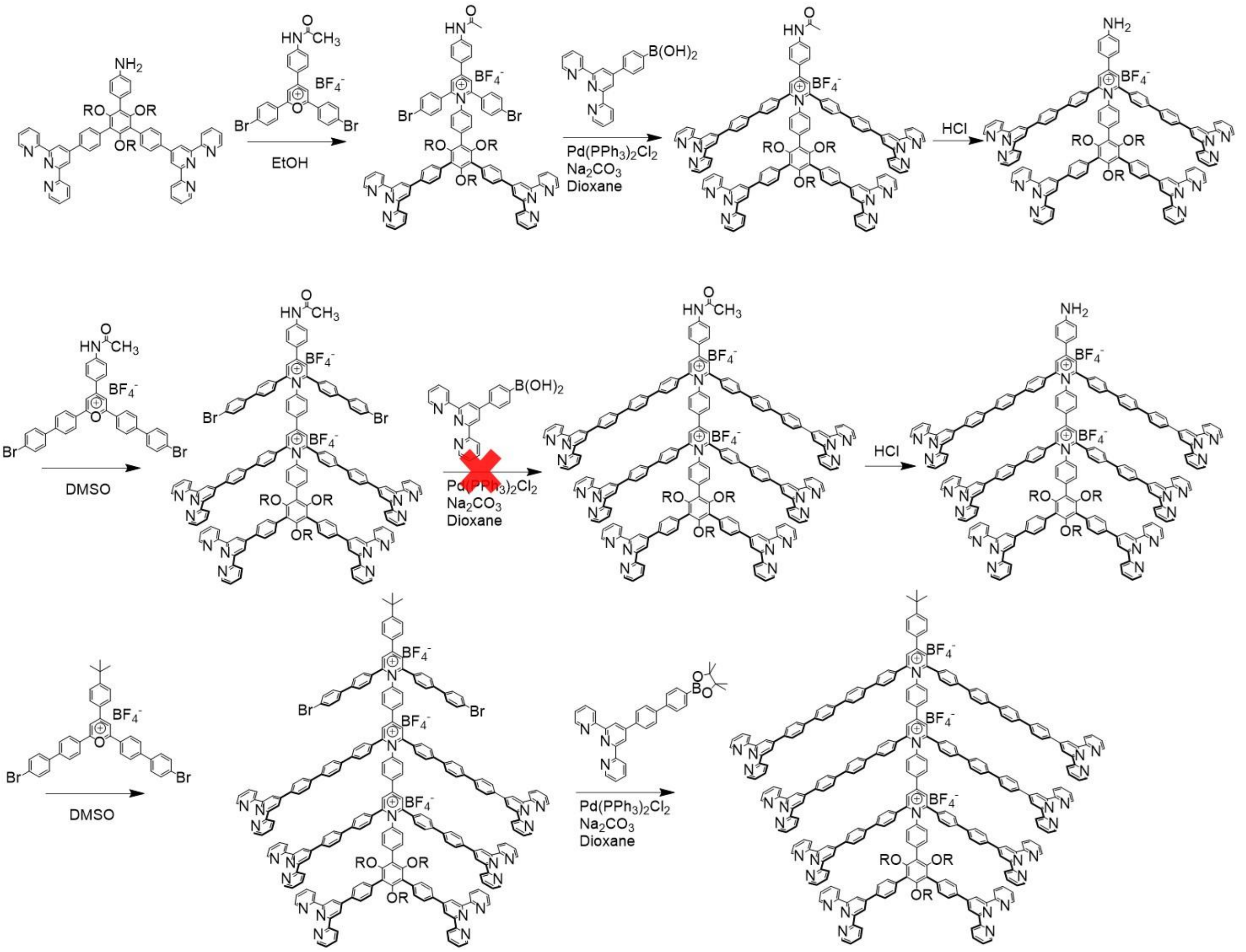

Supplementary Figure 1. Initial synthetic strategy for **L4**.

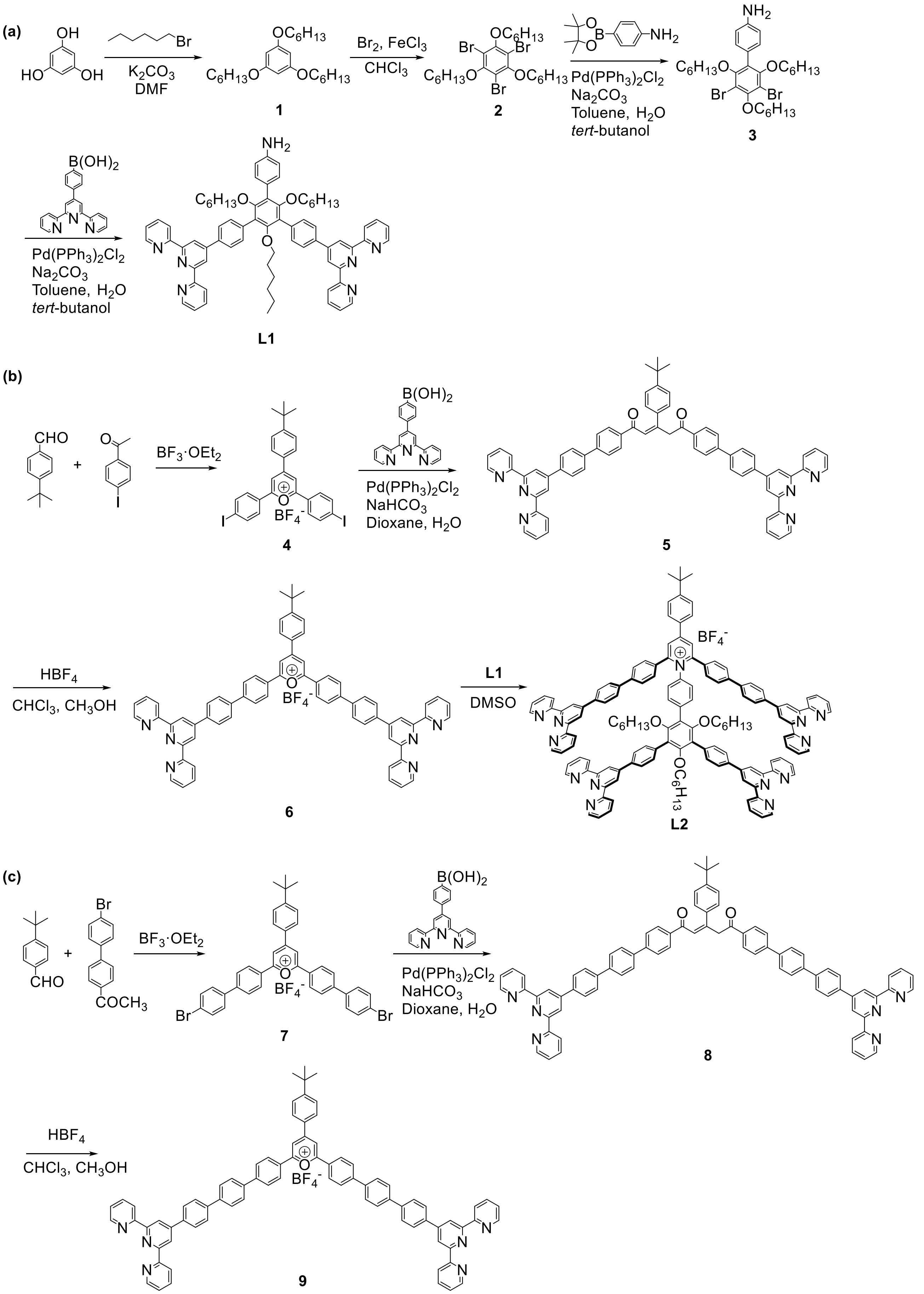
(a)
OH
HO
OH
Br
K_2CO_3
DMF
OC_6H_{13}
$C_6H_{13}O$
OC_6H_{13}
1
Br_2, $FeCl_3$
$CHCl_3$
Br
Br
Br
2
B
O
O
NH_2
$Pd(PPh_3)_2Cl_2$
Na_2CO_3
Toluene, H_2O
tert-butanol
3
$B(OH)_2$
N
L1
(b)
CHO
+
I
$BF_3 \cdot OEt_2$
BF_4^-
4
$NaHCO_3$
Dioxane, H_2O
5
HBF_4
$CHCl_3$, CH_3OH
6
L1
DMSO
L2
(c)
COCH$_3$
7
8
9

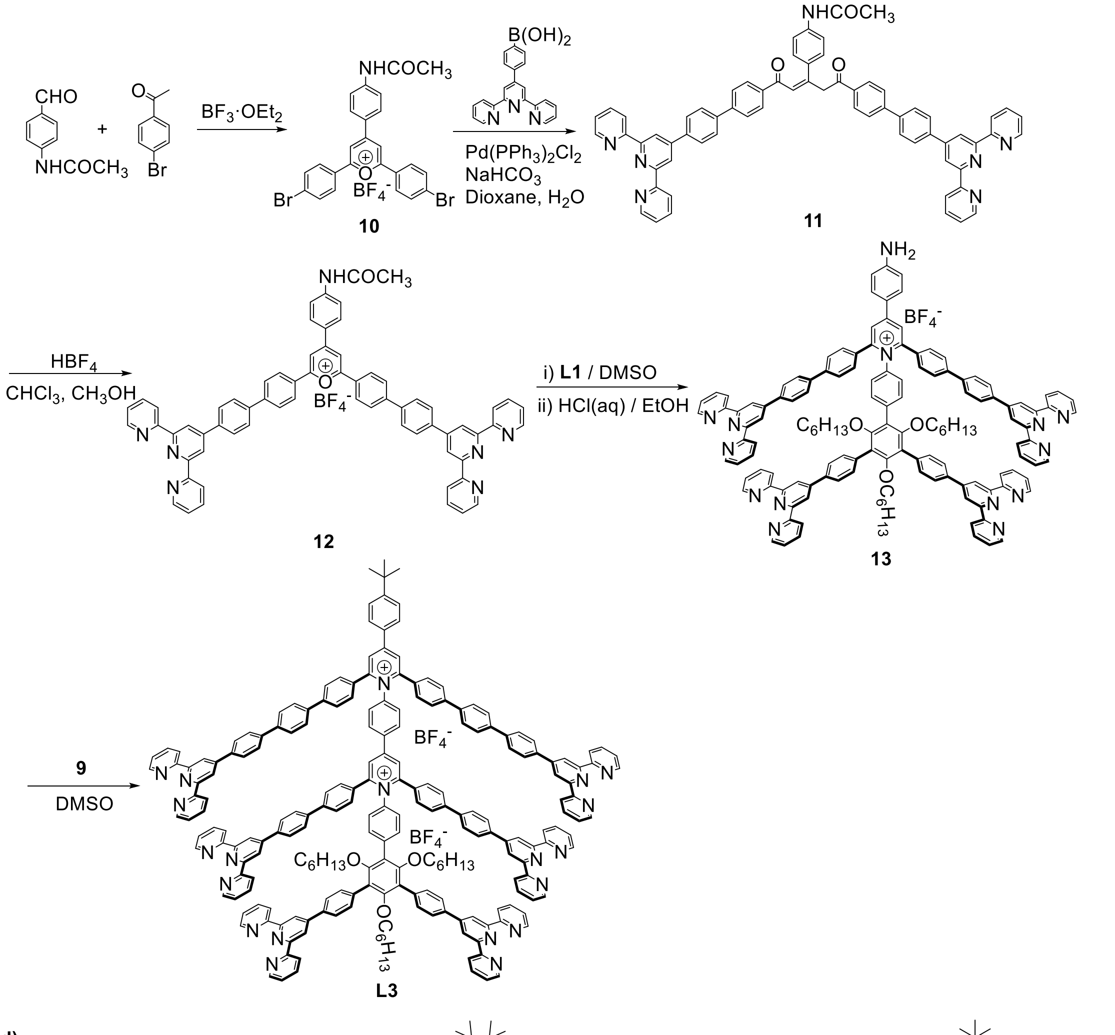
CHO
NHCOCH3
Br
BF3·OEt2
NHCOCH3
BF4-
Br
Br
10
B(OH)2
Pd(PPh3)2Cl2
NaHCO3
Dioxane, H2O
NHCOCH3
11
HBF4
CHCl3, CH3OH
NHCOCH3
BF4-
12
i) L1 / DMSO
ii) HCl(aq) / EtOH
NH2
BF4-
C6H13O
OC6H13
OC6H13
13
9
DMSO
BF4-
BF4-
C6H13O
OC6H13
OC6H13
L3

(d)

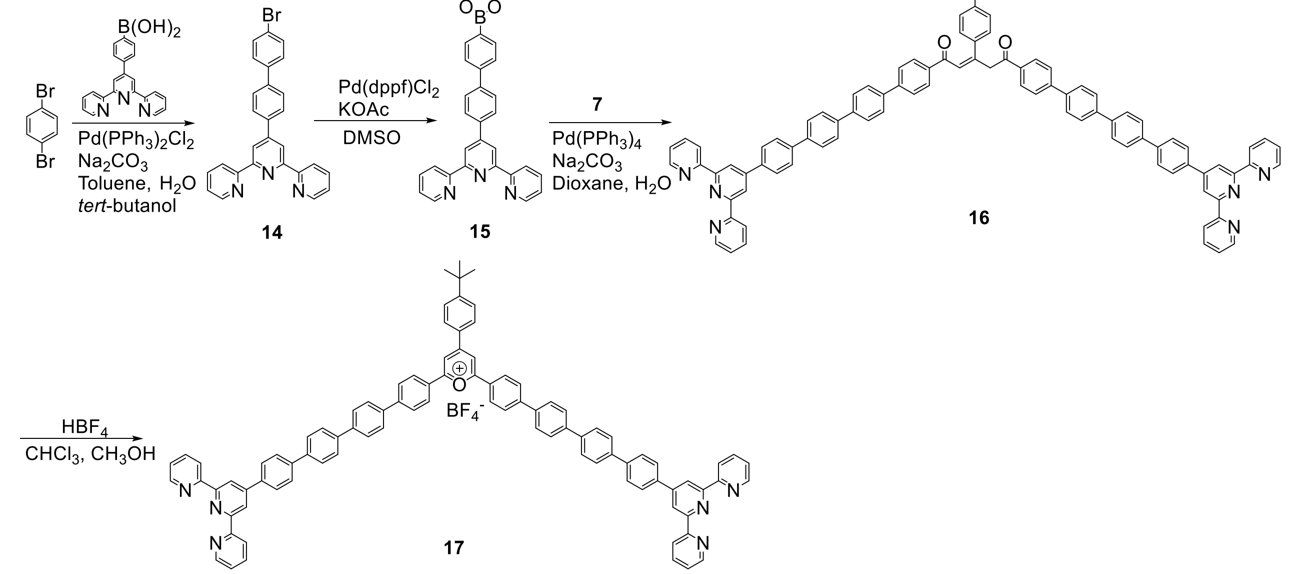
Br
Br
B(OH)2
Pd(PPh3)2Cl2
Na2CO3
Toluene, H2O
tert-butanol
Br
14
Pd(dppf)Cl2
KOAc
DMSO
15
7
Pd(PPh3)4
Na2CO3
Dioxane, H2O
16
HBF4
CHCl3, CH3OH
BF4-
17

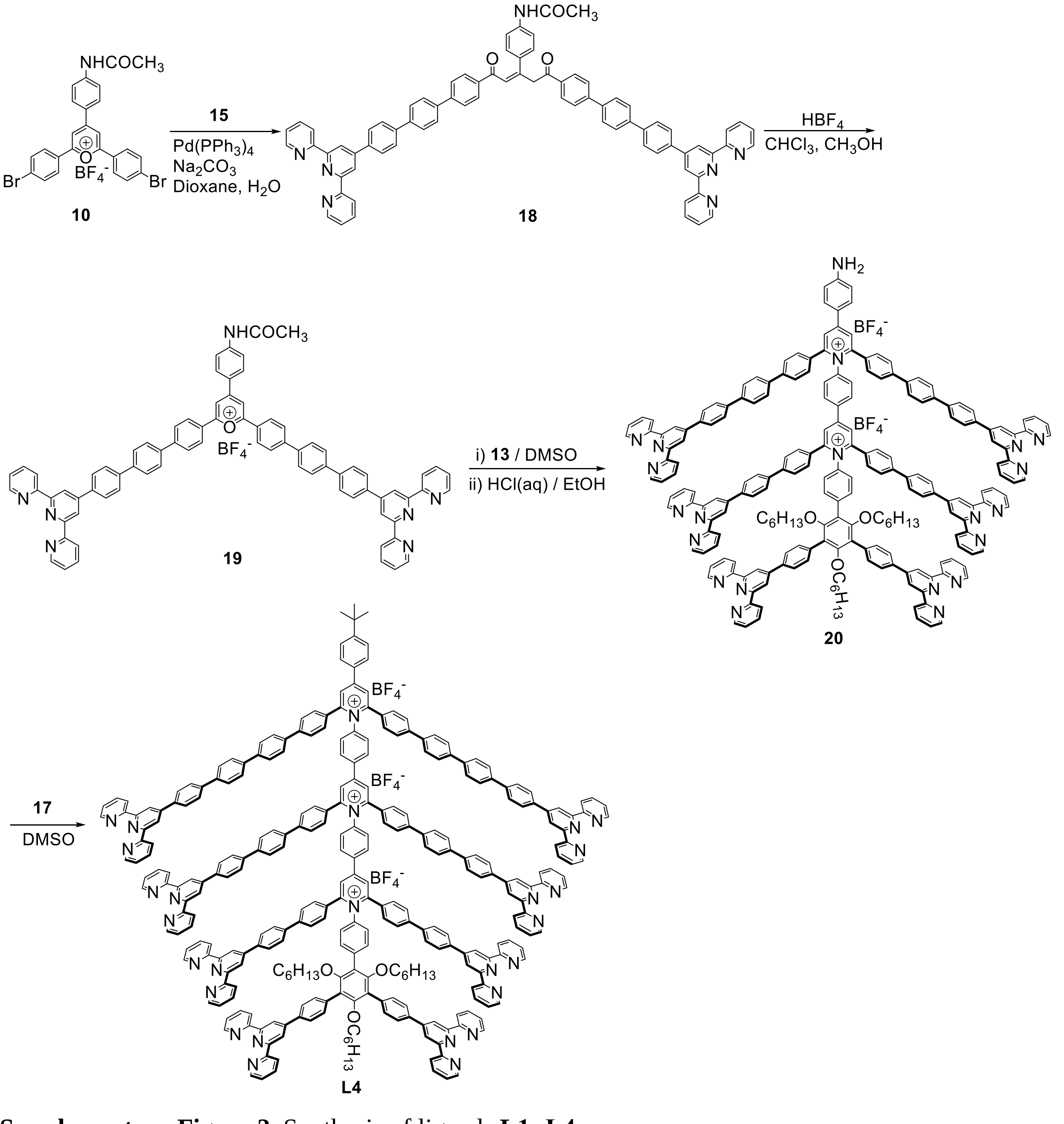

Supplementary Figure 2. Synthesis of ligands **L1−L4**

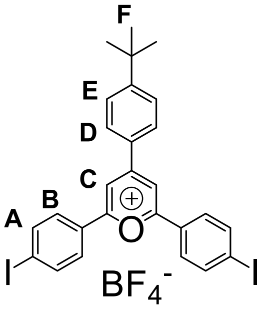

Compound 4: To a 100 mL Schlenk flask, 4-tert-Butylbenzaldehyde (1.62 g, 0.01 mol) and 4'-Iodoacetophenone (4.92 g, 0.02 mol) were added. Under N_2 atmosphere, $BF_3{\cdot}OEt_2$ (10 mL, 0.08 mol) was added and the mixture was stirred at 100℃ for 2 hours. After cooling down to room

temperature, acetone (20 mL) was added and the mixture was poured into diethyl ether (300 mL) to form precipitate. The precipitate was filtered and washed with diethyl ether, which gave compound **4** (3.14 g, 44.6%) as a dark yellow powder after drying in vacuo. ^{1}H NMR (400 MHz, DMSO-d_6) δ 9.16 (s, 2H, Py-H^{C}), 8.56 (d, J = 8.6 Hz, 2H, Ph-H^{D}), 8.32 (d, J = 8.6 Hz, 4H, Ph-H^{B}), 8.19 (d, J = 8.5 Hz, 4H, Ph-H^{A}), 7.79 (d, J = 8.6 Hz, 2H, Ph-H^{E}), 1.39 (s, 9H, *tert-butyl*-H^{F}). ^{13}C NMR (100 MHz, DMSO-d_6) δ 169.66, 165.20, 159.81, 139.20, 130.74, 130.49, 129.05, 127.26, 115.42, 104.99, 35.78, 31.10. ESI-MS (*m/z*): calcd. for $[M-BF_4^{-}]^{+}$ 616.98, found 616.99.

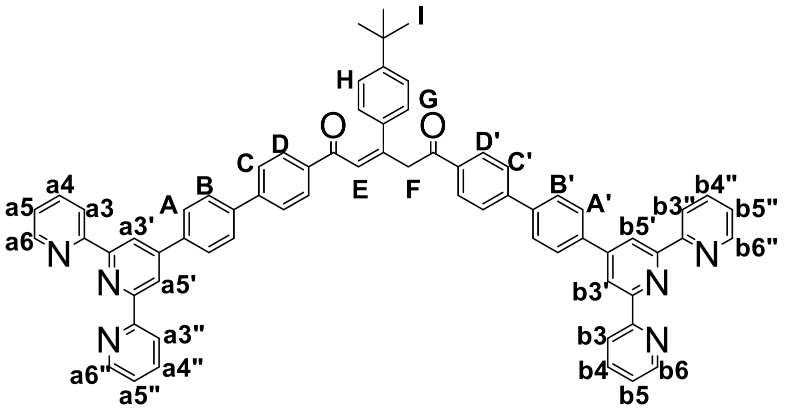

Compound 5: To a 200 mL Schlenck flask, compound **4** (0.61 g, 1 mmol), 4’-(4-boronatophenyl)-2,2’:6’,2”-terpyridine (1.059 g, 3 mmol), $Pd(PPh_3)_2Cl_2$ (70.2 mg, 0.1 mmol) and $NaHCO_3$ (0.84 g, 10 mmol) were added. Under N_2 atmosphere, dioxane (60 mL) and H_2O (20 mL) were injected into the flask. The mixture was kept stirring at 75℃ for 4 hours. After cooling down to room temperature, the solvent was evaporated, and the residue was dissolved in DCM. After washing with water for three times, the organic layer was collected and dried over anhydrous $MgSO_4$. Compound **5** (0.34 g, 34.1%) was obtained as a light orange powder after purification by Al_2O_3 column with DCM/CH_3OH (100:0.2, v/v) as the eluent. ^{1}H NMR (500 MHz, $CDCl_3$) δ 8.80 (d, J = 6.1 Hz, 4H, tpy-$H^{a3’,a5’,b3’,b5’}$), 8.75 (s, 4H, tpy-$H^{a3,a3”,b3,b3”}$), 8.69 (d, J = 7.9 Hz, 4H, tpy-$H^{a6,a6”,b6,b6”}$), 8.21 (d, J = 8.2 Hz, 2H, Ph-H^{D}), 8.12 (d, J = 8.3 Hz, 2H, Ph-$H^{D’}$), 8.03 (t, J = 7.8 Hz, 4H, Ph-$H^{A,A’}$), 7.89 (t, J = 7.7 Hz, 4H, tpy-$H^{a4,a4”}$), 7.79 (dd, J = 16.9, 8.3 Hz, 8H, Ph-$H^{B,B’,C,C’}$), 7.57 (d, J = 8.5 Hz, 2H, Ph-H^{G}), 7.56 (s, 1H, H^{E}), 7.47 (d, J = 8.4 Hz, 2H, Ph-H^{H}), 7.39 – 7.35 (m, 4H, tpy-$H^{a5,a5”,b5,b5”}$), 4.95 (s, 2H, H^{F}), 1.36 (s, 9H, *tert*-butyl-H^{I}). ^{13}C NMR (126 MHz, $CDCl_3$) δ 195.78, 190.11, 156.12, 155,95, 152.84, 152.75, 149.37, 149.12, 144.81, 144.32, 140.48, 140.41, 138.94, 138.15, 138.09, 138.07, 136.86, 136.04, 128.98, 127.81, 127.74, 127.71, 127.24, 127.12, 126.62, 125.80, 123.86, 122.71, 121.36, 118.63, 42.87, 34.76, 31.24. ESI-MS (*m/z*): calcd. for $[M+H]^{+}$ 997.42, found 997.50; calcd. for $[M+2H]^{2+}$ 499.22, found 499.22.

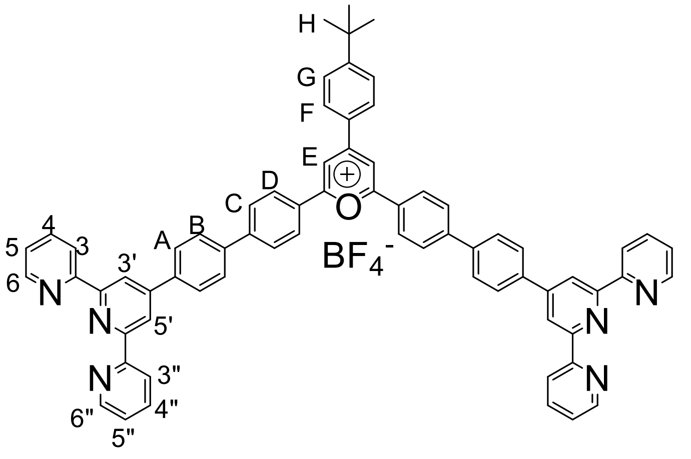

Compound 6: Compound **5** (0.3 g, 0.3 mmol) was dissolved in a mixture of $CHCl_3$ (30 mL) and CH_3OH (10 mL). To the solution, HBF_4 (1 mL, 50 wt. % in water) was added dropwise. After stirring at room temperature overnight, the precipitate was collected by filtration and washed repeatedly with DCM. Compound **6** (0.28 g, 87.5%) was obtained as a red powder after drying in vacuum. ^{1}H NMR (500 MHz, DMSO-d_6) δ 9.06 (s, 2H, Ph-H^{E}), 8.86 – 8.74 (m, 12H, tpy-$H^{3',5'}$, tpy-$H^{3,3''}$, tpy-$H^{6,6''}$), 8.60 (br, 4H, Ph-H^{D}), 8.50 (d, J = 5.7 Hz, 2H, Ph-H^{F}), 8.20 (t, J = 7.5 Hz, 4H, tpy-$H^{4,4''}$), 8.12 (d, J = 6.4 Hz, 8H, Ph-H^{A}, Ph-H^{C}), 8.07 (br, 4H, Ph-H^{B}), 7.75 (d, J = 7.0 Hz, 2H, Ph-H^{G}), 7.69 – 7.62 (m, 4H, tpy-$H^{5,5''}$), 1.40 (s, 9H, *tert*-butyl-H^{I}). ^{13}C NMR (126 MHz, DMSO-d_6) δ 168.28, 163.90, 159.77, 151.21, 150.08, 149.21, 146.23, 144.79, 142.44, 138.87, 135.73, 130.46, 129.48, 129.04, 127.79, 127.36, 126.95, 126.53, 123.36, 119.62, 113.66, 55.31, 35.64, 31.00. ESI-MS (*m/z*): calcd. for $[M-BF_4^-]^+$ 979.41, found 979.53; calcd. for $[M-BF_4^-+H]^{2+}$ 490.21, found 490.24.

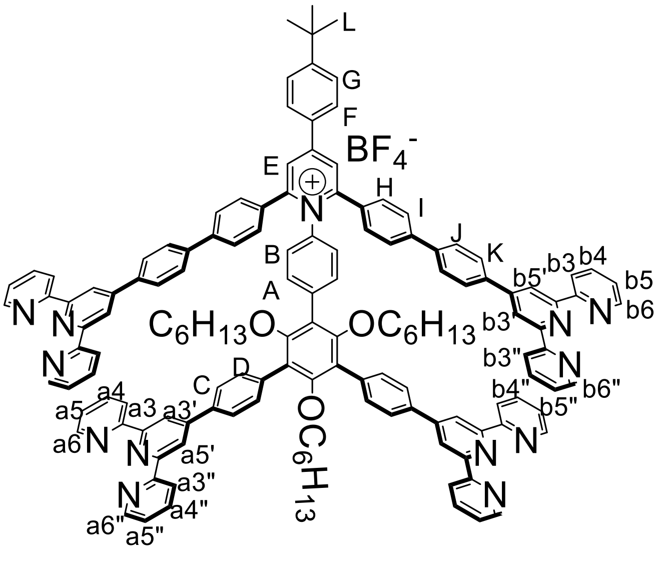

Ligand L2: Compound **6** (160 mg, 0.15 mmol) and compound **L1** (108 mg, 0.1 mmol) were dissolved in DMSO (5 mL). The mixture was kept stirring at 120 ℃ for 36 hours and then cooled

down to room temperature. DCM (100 mL) was added to dissolve the suspension. After washing with brine for three times, organic layer was collected and dried over anhydrous $MgSO_4$. After purification by Al_2O_3 column with DCM/CH_3OH (100:0.7, v/v) as the eluent, **L2** was obtained as a yellowish powder (167mg, 78.3%). ^{1}H NMR (500 MHz, $CDCl_3$) δ 8.73 (s, 4H, tpy-$H^{b3',5'}$), 8.72 (s, 4H, tpy-$H^{a3',5'}$), 8.63 (m, 16H, tpy-$H^{a3,3''}$, tpy-$H^{a6,6''}$, tpy-$H^{b3,3''}$ and tpy-$H^{b6,6''}$), 8.23 (s, 2H, Ph-H^{E}), 7.98 (d, J = 8.6 Hz, 2H, Ph-H^{F}), 7.95 (d, J = 8.3 Hz, 4H, Ph-H^{C}), 7.93 – 7.81 (m, 18H, Ph-$H^{A, I, K}$, tpy-$H^{a4,4''}$, and tpy-$H^{b4,4''}$), 7.65 (m, 8H, Ph-$H^{D, H}$), 7.61 (d, J = 8.6 Hz, 2H, Ph-H^{G}), 7.55 (m, 6H, Ph-$H^{B, J}$), 7.31 – 7.27 (m, 8H, tpy-$H^{a5,5''}$, and tpy-$H^{b5,5''}$), 3.20 (t, J = 6.4 Hz, 2H), 2.91 (t, J = 6.0 Hz, 4H), 1.38 (s, 9H, L), 1.06 – 0.94 (m, 6H), 0.89 – 0.78 (m, 10H), 0.72 – 0.68 (m, 4H), 0.59 (m, 6H), 0.52 (t, J = 7.3 Hz, 6H). ^{13}C NMR (100 MHz, $CDCl_3$) δ156.64, 156.41, 156.34, 156.17, 156.02, 155.88, 155.75, 155.08, 150.01, 149.23, 149.02, 141.72, 139.65, 138.18, 137.81, 136.93, 136.72, 136.67, 135.36, 132.46, 131.66, 131.41, 131.10, 130.70, 128.30, 128.15, 127.87, 127.33, 126.78, 126.74, 126.55, 126.10, 125.96, 123.72, 123.62, 121.20, 121.17, 118.75, 118.59, 73.60, 73.25, 35.11, 31.34, 31.26, 31.04, 29.65, 29.47, 25.34, 25.18, 22.55, 22.40, 13.91, 13.82. High resolution (HR) ESI-MS (*m/z*): calcd. for $[M-BF_4^{-}+H]^{2+}$ 1022.9936, found 1022.9971; $[M-BF_4^{-}+2H]^{3+}$ 682.3317, found 682.3277; $[M-BF_4^{-}+3H]^{4+}$ 512.0007, found 511.9995.

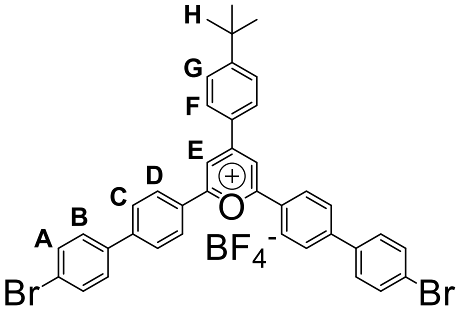

Compound 7: 4-*tert*-Butylbenzaldehyde (1.62 g, 0.01 mol) and 4-(4-bromophenyl)acetophenone (5.50 g, 0.02 mol) were placed in a 100 mL Schlenk flask. $BF_3 \cdot OEt_2$ (10 mL, 0.08 mol) was injected into the flask under N_2 atmosphere. The mixture was heated to 100℃ and kept stirring for 2 hours. After cooling to room temperature, acetone (20 mL) was added to dissolve the viscous brown oil. The dark solution was then poured into diethyl ether (1 L) to form precipitates. The precipitates were filtered and washed repeatedly with diethyl ether, which gave compound **7** as a red powder after drying in vacuum (3.7 g, 48.7%). ^{1}H NMR (400 MHz, DMSO-d_6) δ 9.17 (s, 2H, Ph-H^{E}), 8.67 (d, J = 8.5 Hz, 4H, Ph-H^{D}), 8.58 (d, J = 8.6 Hz, 2H, Ph-H^{F}), 8.13 (d, J = 8.5 Hz, 4H, Ph-H^{C}), 7.88 (d, J = 8.5 Hz, 4H, Ph-H^{B}), 7.79 (dd, J = 11.5, 8.6 Hz, 6H, Ph-H^{A}, Ph-H^{G}), 1.41 (s, 9H, *tert*-butyl-H^{J}). ^{13}C NMR (125 MHz, DMSO-d_6) δ 169.13, 164.56, 159.69, 145.09, 137.45, 132.51, 130.61, 130.07,

129.64, 128.44, 128.04, 127.13, 123.28, 114.74, 35.72, 31.08. ESI-MS (*m/z*): calcd. for $[M-BF_4^-]^+$ 673.07, found 673.09.

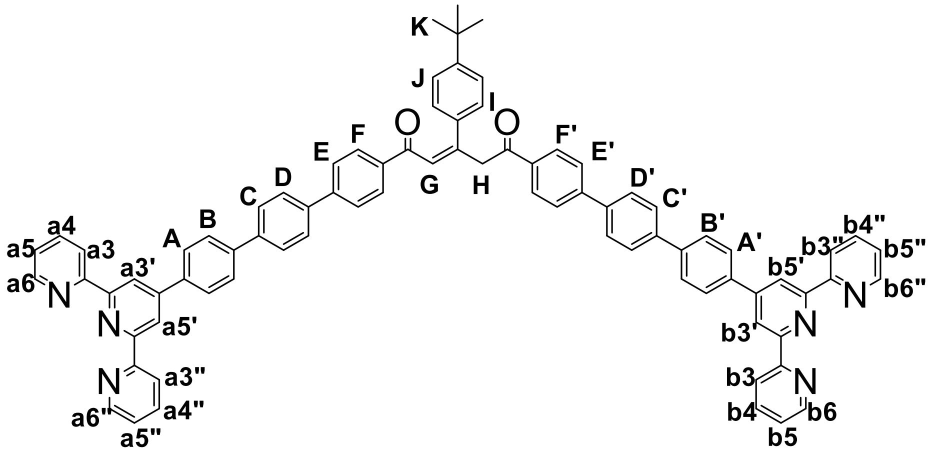

Compound 8: Compound **7** (0.76 g, 1 mmol), 4'-(4-boronatophenyl)-2,2':6',2"-terpyridine (1.059 g, 3 mmol), $Pd(PPh_3)_2Cl_2$ (70.2 mg, 0.1 mmol) and $NaHCO_3$ (0.84 g, 10 mmol) were added into a 200 mL Schlenk flask. Under N_2 atmosphere, dioxane (60 mL) and H_2O (20 mL) were injected. After stirring at 75 ℃ for 4 hours, the solvent was evaporated, and the residue was dissolved in DCM. After washing with water for three times, the organic layer was collected and dried over anhydrous $MgSO_4$. Compound **8** was obtained as a light orange powder after purification by Al_2O_3 column with DCM/CH_3OH (100:0.6, v/v) as the eluent. ^1H NMR (500 MHz, $CDCl_3$) δ 8.81 (d, *J* = 3.2 Hz, 4H, tpy-$H^{a3',a5',b3',b5'}$), 8.76 – 8.74 (m, 4H, tpy-$H^{a3,a'',b3,b3''}$), 8.69 (d, *J* = 7.4 Hz, 4H, tpy-$H^{a6,a6'',b6,b6''}$), 8.20 (d, *J* = 8.3 Hz, 2H, Ph-H^F), 8.11 (d, *J* = 8.3 Hz, 2H, Ph-$H^{F'}$), 8.03 (dd, *J* = 8.2, 3.3 Hz, 4H, Ph-$H^{A,A'}$), 7.89 (t, *J* = 7.1 Hz, 4H, tpy-$H^{a4,a4'',b4,b4''}$), 7.80 – 7.75 (m, 16H, Ph-$H^{B.B',C,C',D,D',E,E'}$), 7.57 (d, *J* = 8.6 Hz, 2H, Ph-H^I), 7.56 (s, 1H, H^G), 7.47 (d, *J* = 8.5 Hz, 2H, Ph-H^J), 7.38 – 7.36 (m, 4H, tpy-$H^{a5,a5'',b5,b5''}$), 4.95 (s, 2H, H^H), 1.36 (s, 9H, *tert-butyl-*H^K). ^{13}C NMR (125 MHz, $CDCl_3/CD_3OD$ = 9/1) δ 196.23, 190.43, 156.09, 155.83, 152.90, 152.64, 149.68, 148.95, 145.15, 144.66, 141.04, 140.07, 140.04, 139.07, 139.02, 138.72, 137.89, 137.37, 137.27, 137.15, 135.76, 128.93, 127.67, 127.52, 127.12, 127.01, 126.53, 125.75, 123.96, 121.64, 118.65, 34.69, 31.12. ESI-MS (*m/z*): calcd. for $[M+H]^+$ 1149.49, found 1149.64; calcd. for $[M+2H]^{2+}$ 575.25, found 575.26.

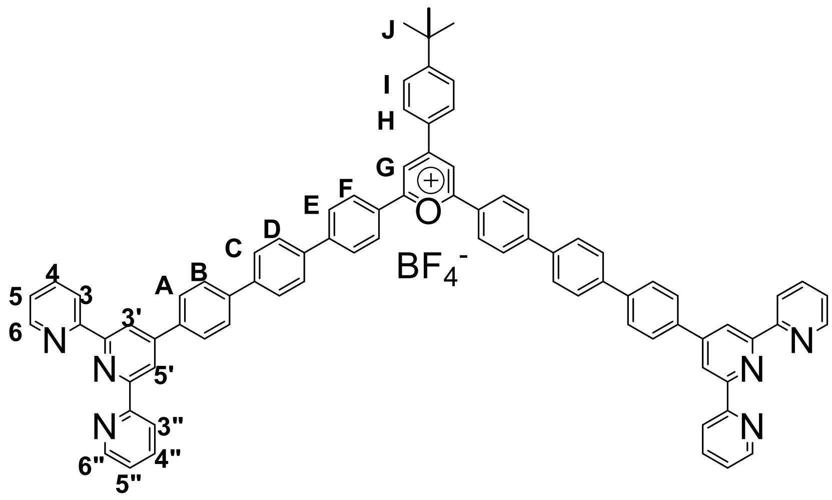

Compound 9: Compound **8** (230 mg, 0.2 mmol) was dissolved in a mixture of $CHCl_3$ (30 mL) and CH_3OH (10 mL), and then kept stirring at room temperature until the solution became clear. To the clear solution, HBF_4 (1 mL, 50 wt. % in water) was added dropwise. After stirring at room temperature overnight, the precipitate was collected by filtration and washed repeatedly with DCM. Compound **9** (212 mg, 87%) was obtained as a dark red solid after drying in vacuum, which was used for next step without further purification. ^{1}H NMR (500 MHz, DMSO-d_6) δ 9.03 (s, 2H, Py-H^G), 8.75 (d, J = 4.5 Hz, 4H, tpy-$H^{6,6''}$), 8.74 (s, 4H, tpy-$H^{3',5'}$), 8.69 (d, J = 8.1 Hz, 4H, tpy- $H^{3,3''}$), 8.56 (d, J = 8.2 Hz, 4H, Ph-H^F), 8.48 (d, J = 8.5 Hz, 2H, H^H), 8.08 (t, J = 7.5 Hz, 8H, tpy-$H^{4,4''}$, Ph-H^E), 8.02 (d, J = 8.3 Hz, 4H, Ph-H^A), 7.97 (d, J = 8.3 Hz, 4H, Ph-H^D), 7.93 (d, J = 8.2 Hz, 4H, Ph-H^B), 7.90 (d, J = 8.2 Hz, 4H, Ph-H^C), 7.72 (d, J = 8.4 Hz, 2H, Ph-H^I), 7.57 – 7.54 (m, 4H, tpy-$H^{5,5''}$), 1.38 (s, 9H, *tert-butyl*-H^J). ^{13}C NMR (125 MHz, DMSO-d_6) δ 168.57, 163.87, 162.75, 159.62, 153.14, 152.19, 149.38, 147.59, 145.55, 140.50, 139.60, 137.16, 135.68, 130.43, 129.65, 129.56, 129.29, 127.80, 127.69, 127.50, 126.92, 125.80, 122.56, 119.06, 113.80, 55.35, 35.63, 31.01. ESI-MS (*m/z*): calcd. for $[M-BF_4^-]^+$ 1131.48, found 1131.65; calcd. for $[M-BF_4^-+H]^{2+}$ 566.24, found 566.27.

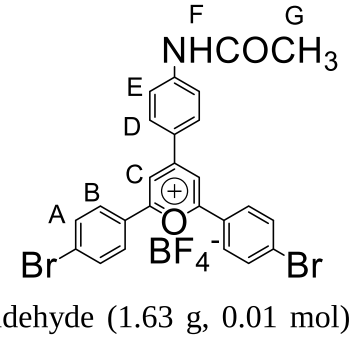

Compound 10: 4-Acetamidobenzaldehyde (1.63 g, 0.01 mol) and 4'-bromoacetophenone (3.98 g, 0.02 mol) were added to a 100 mL Schlenk flask successively. $BF_3 \cdot OEt_2$ (10 mL, 0.08 mol) was added dropwise to the mixture under N_2 atmosphere. The mixture was then heated to 100 ℃ and

kept stirring for 2 hours. After cooling down to room temperature, acetone (20 mL) was added to dissolve the viscous oil. After stirring at room temperature for 30 mins, the suspension was poured into diethyl ether (500 mL). Compound **10** was obtained as a dark red solid after filtration, which was washed repeatedly with diethyl ether and used for next step without further purification. ^{1}H NMR (400 MHz, DMSO-d_6) δ 10.65 (s, 1H, NH-H^{F}), 9.07 (s, 2H, Py-H^{C}), 8.66 (d, J = 8.9 Hz, 2H, Ph-H^{D}), 8.47 (d, J = 8.6 Hz, 4H, Ph-H^{B}), 8.01 (d, J = 8.6 Hz, 4H, Ph-H^{A}), 7.95 (d, J = 8.8 Hz, 2H, Ph-H^{E}), 2.17 (s, 3H, $COCH_3$-H^{G}). ^{13}C NMR (100 MHz, DMSO-d_6) δ 170.03, 168.05, 163.30, 147.25, 133.24, 132.56, 130.47, 129.80, 128.38, 125.81, 119.36, 113.42, 24.87. ESI-MS (*m/z*): calcd. for $[M-BF_4^{-}]^{+}$ 521.97, found 521.98.

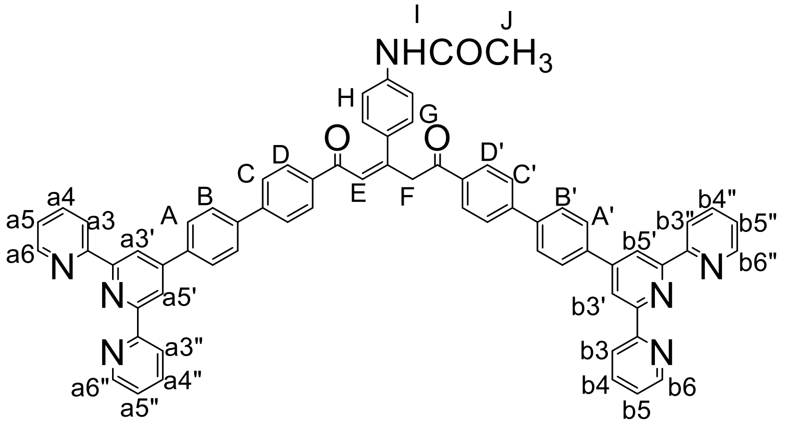

Compound 11: Compound **10** (0.61 g, 1 mmol), 4’-(4-boronatophenyl)-2,2’:6’,2”-terpyridine (1.059 g, 3 mmol), $Pd(PPh_3)_2Cl_2$ (70.2 mg, 0.1 mmol) and $NaHCO_3$ (0.84 g, 10 mmol) were added into a 200 mL Schlenk flask. Under N_2 atmosphere, dioxane (60 mL) and water (20 mL) were added. The mixture was heated to 75℃ and kept stirring for 4 hours. After cooling down to room temperature, the solvent was evaporated. The residue was dissolved in DCM, and then washed with water for 3 times. The organic layer was collected and dried over anhydrous $MgSO_4$. After purification by Al_2O_3 column with DCM/CH_3OH (100:0.6, v/v) as the eluent, compound **11** (0.47 g, 47.1%) was obtained as a pale-yellow powder. ^{1}H NMR (500 MHz, $CDCl_3$) δ 8.80 (d, J = 5.6 Hz, 4H, tpy-$H^{a3',a5',b3',b5'}$), 8.75 (s, 4H, tpy-$H^{a6,a6'',b6,b6''}$), 8.69 (d, J = 7.6 Hz, 4H, tpy-$H^{a3,a3'',b3,b3''}$), 8.21 (d, J = 8.1 Hz, 2H, Ph-$H^{D'}$), 8.12 (d, J = 8.2 Hz, 2H, Ph-H^{D}), 8.04 (t, J = 7.5 Hz, 4H, $H^{A,A'}$), 7.90 (t, J = 7.7 Hz, 4H, tpy-$H^{a4,a4'',b4,b4''}$), 7.80 (dd, J = 14.6, 7.3 Hz, 8H, $H^{B,B',C,C'}$), 7.60 (s, 4H, Ph-$H^{G,H}$), 7.53 (s, 1H, H^{E}), 7.39 – 7.35 (m, 4H,tpy-$H^{a5,a5'',b5,b5''}$), 4.94 (s, 2H, H^{F}), 2.22 (s, 3H, $COCH_3$-H^{J}). ^{13}C NMR (125 MHz, DMSO-d_6) δ 195.71, 194.70, 168.61, 155.80, 154.93, 152.09, 149.40, 148.81, 143.59, 143.24, 140.77, 139.91, 137.76, 137.52, 137.31, 128.96, 127.98, 127.80, 127.67, 126.98, 124.60, 120.98, 118.77, 117.85, 42.04, 24.09. ESI-MS (*m/z*): calcd. for $[M+H]^{+}$ 998.38, found 998.49; calcd. for

$[M+2H]^{2+}$ 499.70, found 499.71.

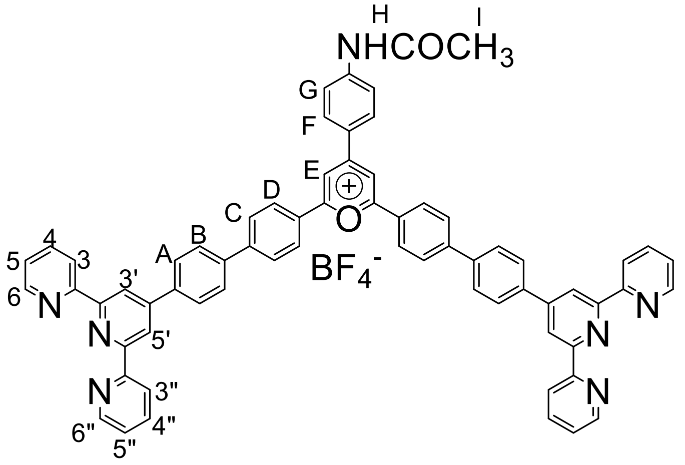

Compound 12: Compound **11** (0.39 g, 0.4 mmol) was dissolved in a mixture of $CHCl_3$ (60 mL) and CH_3OH (20 mL). The suspension was kept stirring at room temperature until a clear solution was formed. HBF_4 (1 mL, 50 wt. % in water) was added dropwise to this clear solution, then kept stirring at room temperature overnight. The precipitate was collected by centrifugation and washed repeatedly with DCM. After drying in vacuum, compound **12** was obtained as a dark red solid (0.35 g, 82.2%). ^{1}H NMR (500 MHz, DMSO-d_6) δ 10.59 (s, 1H, NH-H^H), 8.88 (s, 2H, Py-H^E), 8.72 (d, J = 4.8 Hz, 4H, tpy-$H^{6,6"}$), 8.67 (s, 4H, tpy-$H^{3',5'}$), 8.63 (d, J = 7.9 Hz, 4H, tpy-$H^{3,3"}$), 8.55 (d, J = 8.8 Hz, 2H, Ph-H^F), 8.51 (d, J = 8.1 Hz, 4H, Ph-H^D), 8.04 – 7.98 (m, 16H, tpy-$H^{4,4"}$, Ph-$H^{A,B,C}$), 7.87 (d, J = 8.7 Hz, 2H, Ph-H^G), 7.53 (m, 4H, tpy-$H^{5,5"}$), 2.17 (s, 3H, $COCH_3$-H^I). ^{13}C NMR (150 MHz, DMSO-d_6) δ 164.05, 162.42, 161.52, 159.04, 158.26, 152.83, 151.75, 149.65, 147.29, 143.67, 141.37, 139.78, 136.51, 128.10, 126.20, 123.06, 119.66, 60.81. ESI-MS (*m/z*): calcd. for $[M-BF_4^-]^+$ 980.37, found 980.47; calcd. for $[M-BF_4^-+H]^{2+}$ 490.69, found 490.71

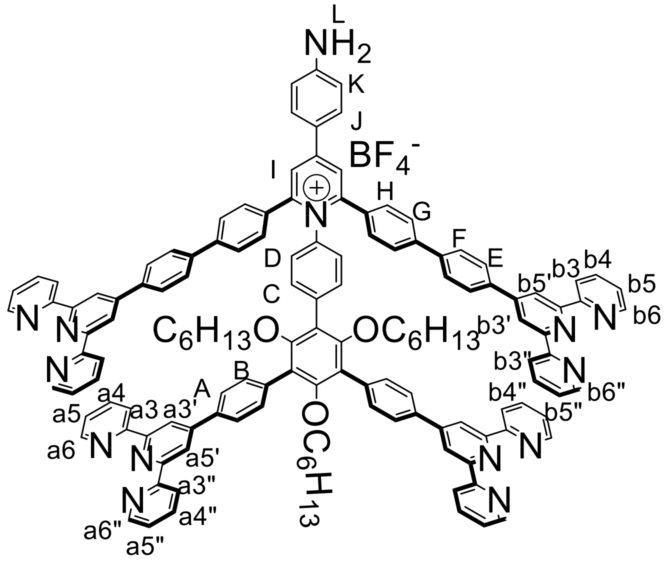

Compound 13: To a 50 mL round bottom flask, **L1** (217 mg, 0.2 mmol), compound **2** (427 mg, 0.4

mmol) and DMSO (8 mL) were added successively. The mixture was heated to 120℃ and kept stirring for 36 hours. After cooling down to room temperature, the mixture was diluted with 5 mL DCM, and then suspended in ethanol (60 mL). To the suspension, HCl (5 mL, 4 mol/L) was added dropwise. After stirring at 85℃ overnight, the suspension was cooled to room temperature and neutralized with $NaHCO_3$. The solvent was evaporated under reduced pressure and the residue was dissolved in DCM. After washing three time with water, the organic layer was dried over anhydrous $MgSO_4$. Compound **13** (169 mg, 40%) was obtained as an orange powder after purification by Al_2O_3 column chromatography (DCM/CH_3OH, 100:1.2, v/v). ^{1}H NMR (400 MHz, $CDCl_3$) δ 8.73 (s, 4H, tpy-$H^{ab3',5'}$), 8.71 (s, 4H, tpy-$H^{a3',5'}$), 8.63 (m, 16H, tpy-$H^{a3,3''}$, tpy-$H^{a6,6''}$, tpy-$H^{b3,3''}$ and tpy-$H^{b6,6''}$), 7.92 (m, 10H, Ph-$H^{I, A, H}$), 7.88 – 7.80 (m, 8H, tpy-$H^{a4,4''}$, and tpy-$H^{b4,4''}$), 7.71 (d, J = 8.7 Hz, 2H, Ph-H^{J}), 7.70 – 7.60 (m, 12H, Ph-$H^{C, D, E, G}$), 7.59 – 7.51 (m, 8H, Ph-$H^{B, F}$), 7.29 (dd, J = 7.2, 4.3 Hz, 8H, tpy-$H^{a5,5''}$, and tpy-$H^{b5,5''}$), 6.89 (d, J = 8.7 Hz, 2H, Ph-H^{K}), 5.74 (s, 2H, NH^{L}), 3.21 (t, J = 6.3 Hz, 2H), 2.98 – 2.85 (m, 4H), 1.25 (dd, J = 9.3, 4.7 Hz, 2H), 1.00 (dd, J = 12.9, 6.9 Hz, 4H), 0.94 – 0.69 (m, 14H), 0.69 – 0.46 (m, 18H). ^{13}C NMR (125 MHz, $CDCl_3$) δ 156.17, 155.98, 155.86, 155.76, 155.09, 149.98, 149.02, 141.57, 139.52, 138.16, 136.95, 136.72, 135.34, 132.69, 131.78, 131.40, 130.46, 130.26, 128.21, 127.86, 127.27, 126.74, 126.56, 126.12, 123.74, 123.65, 121.96, 121.19, 118.74, 118.54, 115.36, 73.63, 73.20, 36.14, 31.35, 31.27, 29.68, 29.47, 25.36, 25.18, 22.56, 22.40, 13.92, 13.83. HR ESI-MS (m/z): calcd. for $[M-BF_4^-+H]^{2+}$ 1002.4677, found 1002.4836; calcd. for $[M-BF_4^-+2H]^{3+}$ 668.6478, found 668.6466.

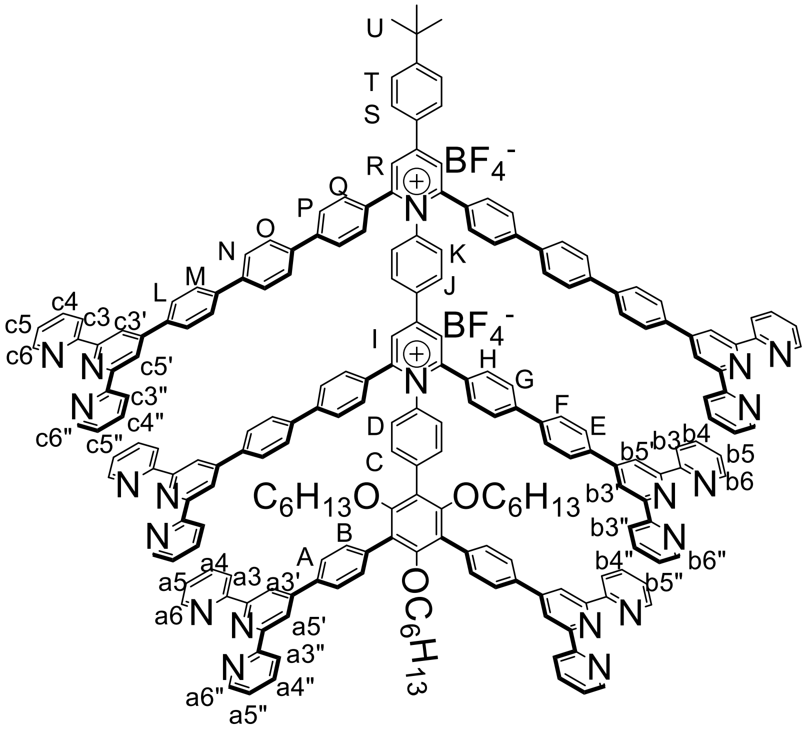

Ligand L3: A mixture of compound **13** (150 mg, 0.064 mmol), compound **9** (131 mg, 0.11 mmol), and molecular sieve 4Å (10 mg) in DMSO (8 mL) was stirred at 135℃ for 36 hours. After cooling down to room temperature, the mixture was dissolved in DCM (100 mL) and washed repeatedly with brine water. The organic layer was collected and dried over anhydrous $MgSO_4$. After removal of the solvent under reduced pressure, the crude product was submitted to Al_2O_3 column chromatography with DCM/CH_3OH (100:1, v/v) as the eluent for twice, which gave ligand **L3** as a yellowish powder. Yield: 65 mg, 31%. ^{1}H NMR (500 MHz, $CDCl_3$) δ 8.72 (s, 4H, tpy-$H^{b3',5'}$), 8.70 (s, 4H, tpy-$H^{a3',5'}$), 8.67 (d, J = 4.2 Hz, 4H, tpy-$H^{b3,3''}$), 8.65 – 8.54 (m, 24H, tpy-$H^{c3',5'}$, tpy-$H^{a6,6''}$, tpy-$H^{a3,3''}$, tpy-$H^{b6,6''}$, tpy-$H^{c3,3''}$ and tpy-$H^{c6,6''}$), 8.25 (m, 4H, Ph-$H^{I,R}$), 7.94 (t, J = 7.8 Hz, 6H, Ph-$H^{E,J}$), 7.91 – 7.86 (m, 10H, Ph-$H^{A,Q,S}$), 7.86 – 7.77 (m, 16H, Ph-H^{H}, tpy-$H^{a4,4''}$, tpy-$H^{b4,4''}$and tpy-$H^{c4,4''}$), 7.76 – 7.61 (m, 24H, Ph-$H^{D,F,G,K,L,M,O}$), 7.58 (t, J = 8.1 Hz, 10H, Ph-$H^{C,P,N}$), 7.53 (m, 6H, Ph-$H^{B,T}$), 7.28 (m, overlapped with C*H*Cl_3, 8H, tpy-$H^{a5,5''}$and tpy-$H^{b5,5''}$), 7.24 (dd, J = 6.8, 5.3 Hz, 4H, tpy-$H^{c5,5''}$), 3.19 (t, J = 6.3 Hz, 2H), 2.91 (t, J = 5.3 Hz, 4H), 1.40 (s, 9H, U), 1.07 – 0.92 (m, 6H), 0.92 – 0.66 (m, 15H), 0.55 (dt, J = 46.9, 7.3 Hz, 18H). ^{13}C NMR (125 MHz, $CDCl_3$) δ 156.66, 156.17, 155.96, 155.74, 155.05, 150.00, 149.01, 142.03, 139.18, 138.22, 137.36, 136.97, 136.69, 135.29, 131.56, 131.39, 130.88, 130.61, 128.34, 127.86, 127.66, 127.45, 127.37, 127.30, 127.23, 127.01, 126.85, 126.75, 126.57, 126.17, 125.67, 123.64, 123.39, 121.19, 118.78, 118.57, 118.46, 73.64, 73.22, 31.57, 31.29, 31.06, 29.69, 29.47, 25.35, 25.26, 25.19, 22.64, 22.54, 22.42, 14.12, 13.91, 13.85. HR ESI-MS (*m/z*): calcd. for $[M-2BF_4^{-}+H]^{3+}$ 1039.4667, found 1039.5105; calcd. for $[M-2BF_4^{-}+2H]^{4+}$ 779.8519, found 779.8520; calcd. for $[M-2BF_4^{-}+3H]^{5+}$ 624.0831, found 624.0800.

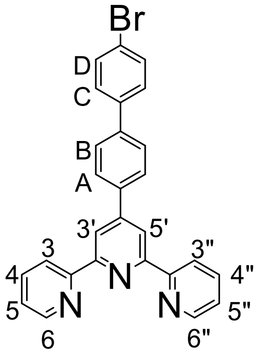

Compound 14: 1,4-Dibromobenzene (7.08g, 0.03 mol), 4'-(4-boronatophenyl)-2,2':6',2"-terpyridine (3.53 g, 0.01 mol), $Pd(PPh_3)_2Cl_2$ (210 mg, 0.3 mmol) and Na_2CO_3 (5.3 g, 0.05 mol) were mixed in toluene (60 mL), H_2O (25 mL) and *tert*-butanol (5 mL). The mixture was stirred at 75℃ for 20 hours under N_2 atmosphere. After cooling down to room

temperature, the solvent was evaporated in vacuo and the residue was dissolved in DCM. After washing with water for several times, the organic layer was collected, dried over anhydrous $MgSO_4$ and removed under reduced pressure. Compound **14** (3.51 g, 75.8%) was obtained as a white powder after purification by silica gel column chromatography. (DCM/CH_3OH, 100:0.2, v/v). ^1H NMR (500 MHz, $CDCl_3$) δ 8.79 (s, 2H, tpy-$H^{3', 5'}$), 8.75 (d, J = 4.2 Hz, 2H, tpy-$H^{6, 6''}$), 8.69 (d, J = 7.9 Hz, 2H, tpy-$H^{3,3''}$), 8.00 (d, J = 8.3 Hz, 2H, Ph-H^A), 7.89 (td, J = 7.8, 1.7 Hz, 2H, tpy-$H^{4,4''}$), 7.70 (d, J = 8.3 Hz, 2H, Ph-H^B), 7.60 (d, J = 8.4 Hz, 2H, Ph-H^C), 7.54 (d, J = 8.5 Hz, 2H, Ph-H^D), 7.37 (dd, J = 6.9, 5.3 Hz, 2H, tpy-$H^{5,5''}$). ^{13}C NMR (125 MHz, $CDCl_3$) δ 156.16, 155.95, 149.47, 149.11, 140.48, 139.25, 137.61, 136.85, 131.94, 128.65, 127.80, 127.33, 123.85, 121.90, 121.35, 118.61. ESI-MS (*m/z*): calcd. for $[M+H]^+$ 464.08, found 464.08.

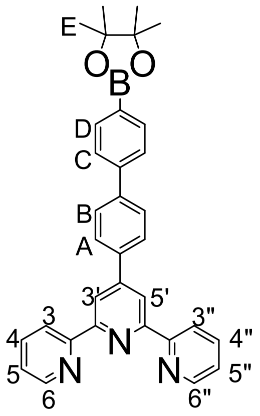

Compound 15: Compound **14** (1.389 g, 3 mmol), bis(pinacolato)diboron (0.724 g, 2.85 mmol), KOAc (1.177 g, 15 mmol) and Pd(dppf)Cl_2 (0.123 g, 0.15 mmol) were placed in a 100 mL flask. DMSO (20 mL) was added and the mixture was heated at 85℃ for 12 hours under N_2 atmosphere. After cooling down to room temperature, brine water was added and the mixture was extracted with DCM for 3 times. The organic layer was collected, dried over anhydrous $MgSO_4$ and removed to dryness. The residue was submitted to Al_2O_3 column (DCM/CH_3OH, 100:0.5, v/v), which gave compound **15** (1.14 g, 74.1%) as a light purple solid. ^1H NMR (400 MHz, $CDCl_3$) δ 8.79 (s, 2H, tpy-$H^{3', 5'}$), 8.75 (d, J = 4.0 Hz, 2H, tpy-$H^{6, 6''}$), 8.69 (d, J = 7.9 Hz, 2H, tpy-$H^{3,3''}$), 8.01 (d, J = 8.3 Hz, 2H, Ph-H^A), 7.92 (d, J = 8.1 Hz, 2H, Ph-H^C), 7.88 (dd, J = 7.7, 1.6 Hz, 2H, tpy-$H^{4,4''}$), 7.77 (d, J = 8.3 Hz, 2H, Ph-H^B), 7.69 (d, J = 8.1 Hz, 2H, Ph-H^D), 7.36 (dd, J = 6.9, 5.3 Hz, 2H, tpy-$H^{5,5''}$), 1.38 (s, 12H, H^E). ^{13}C NMR (100 MHz, $CDCl_3$) δ 156.21, 155.94, 149.64, 149.10, 143.00, 141.56, 137.54, 136.83, 135.33, 127.66, 126.37, 123.80, 121.35, 118.66, 83.84, 24.88. ESI-MS (*m/z*): calcd. for $[M+H]^+$ 511.26, found 511.27.

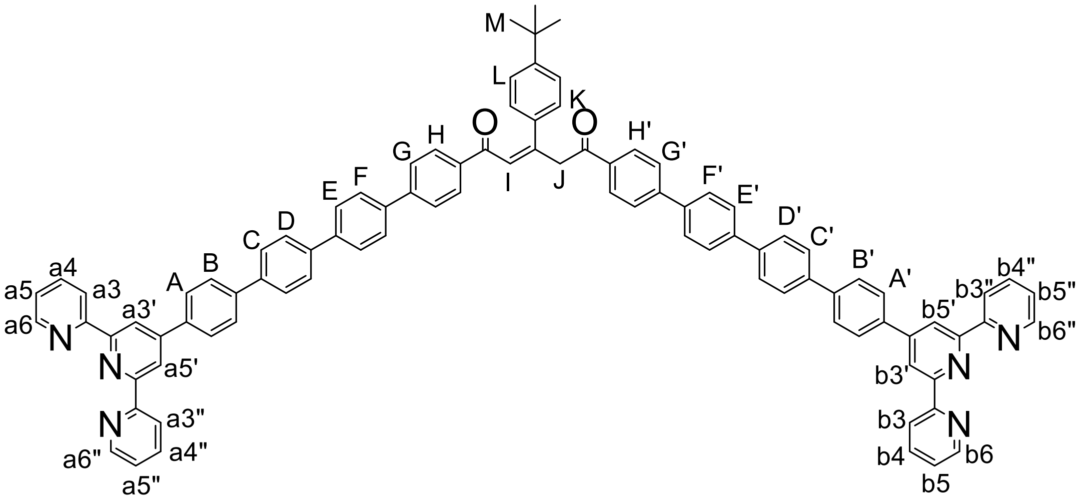

Compound 16: Compound **7** (381 mg, 0.5 mmol), compound **15** (766 mg, 1.5 mmol), $Pd(PPh_3)_4$ (57.8 mg, 0.05 mmol) and Na_2CO_3 (530 mg, 5 mmol) were added into a mixture of dioxane (60 mL) and water (20 mL) under N_2 atmosphere. The mixture was kept stirring at 75℃ for 4 hours. After the solvent was evaporated in vacuo, the residue was dissolved in DCM and washed repeatedly with water. The organic layer was collected and dried over anhydrous $MgSO_4$. After purification with Al_2O_3 column (DCM/CH_3OH, 100:0.8, v/v), compound **16** (298 mg, 45.8%) was obtained as a light orange powder. ^{1}H NMR (500 MHz, $CDCl_3$) δ 8.81 (d, J = 1.6 Hz, 4H, tpy-$H^{a3',a5',b3',b5'}$), 8.75 (d, J = 3.1 Hz, 4H, tpy-$H^{a6,a6'',b6,b6''}$), 8.70 (d, J = 7.9 Hz, 4H, tpy-$H^{a3,a3'',b3,b3''}$), 8.20 (d, J = 7.9 Hz, 2H, Ph-H^{H}), 8.11 (d, J = 7.9 Hz, 2H, Ph-$H^{H'}$), 8.04 (d, J = 7.4 Hz, 4H, Ph-$H^{A,A'}$), 7.90 (t, J = 7.7 Hz, 4H, tpy-$H^{a4,a4'',b4,b4''}$), 7.78 (dd, J = 7.2, 4.1 Hz, 24H), 7.57 (d, J = 8.6 Hz, 2H, Ph-H^{K}), 7.56 (s, 1H, H^{I}), 7.47 (d, J = 8.4 Hz, 2H, Ph-H^{L}), 7.39 – 7.36 (m, 4H, tpy-$H^{a5,a5'',b5,b5''}$), 4.95 (s, 2H, H^{J}), 1.36 (s, 9H, *tert-butyl*-H^{M}). ^{13}C NMR (125 MHz, $CD_3OD/CDCl_3$ = 1/4) δ 156.07, 155.85, 149.85, 148.84, 141.38, 139.59, 139.17, 137.46, 136.96, 127.57, 127.50, 127.40, 124.14, 121.92, 118.67, 31.00, 30.88, 30.73, 29.58. ESI-MS (*m/z*): calcd. for $[M+H]^+$ 1301.55, found 1301.60; calcd. for $[M+2H]^{2+}$ 651.28, found 651.29.

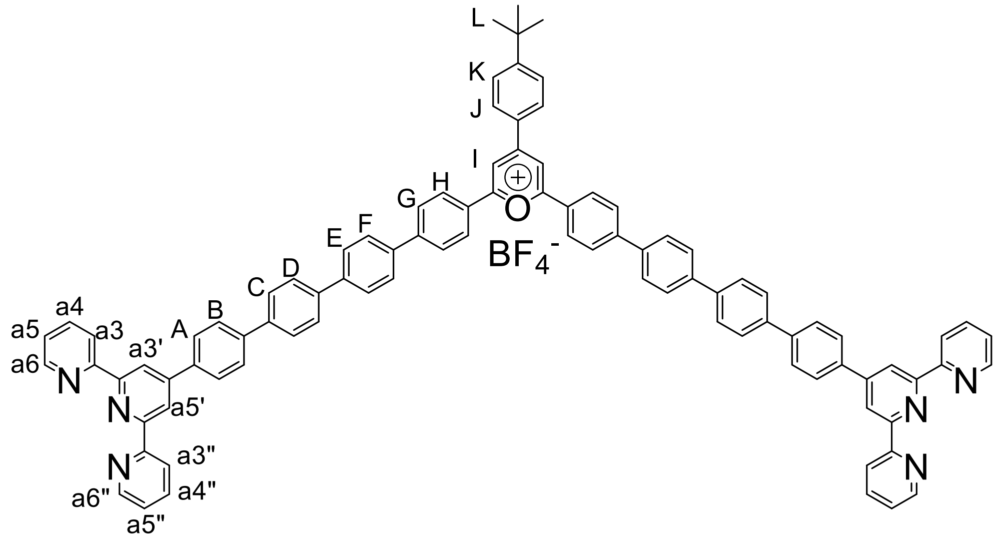

Compound 17: Compound **16** (130 mg, 0.1 mmol) was dissolved in a mixture of $CHCl_3$ (30 mL) and CH_3OH (10 mL). The suspension was stirred at room temperature until a clear solution was formed. To this clear solution, HBF_4 (1 mL, 50% wt. in water) was added dropwise. After stirring at room temperature overnight, the precipitate was collected by centrifugation and washed repeatedly with DCM. Compound **17** was obtained as a dark red solid after drying in vacuo. ^{1}H NMR (500 MHz, DMSO-d_6) δ 8.93 (s, 2H, Py-H^I), 8.72 (d, J = 3.7 Hz, 4H, tpy-$H^{6,6''}$), 8.66 (d, J = 9.5 Hz, 8H, tpy-$H^{3',5'}$, tpy-$H^{3,3''}$), 8.44 (d, J = 7.2 Hz, 6H,Ph-$H^{H,J}$), 8.09 – 8.04 (m, 4H, tpy-$H^{4,4''}$), 7.99 (d, J = 7.5 Hz, 4H, Ph-H^G), 7.94 (d, J = 7.4 Hz, 4H, Ph-H^A), 7.87 (s, 8H, Ph-$H^{E,F}$), 7.82 (d, J = 8.9 Hz, 12H, Ph-$H^{B,C,D}$), 7.70 (d, J = 8.1 Hz, 2H, Ph-H^K), 7.56 – 7.50 (m, 4H, tpy-$H^{5,5''}$), 1.39 (s, 9H, *tert*-butyl-H^L). ^{13}C NMR (125 MHz, DMSO-d_6) δ 168.39, 161.11, 159.20, 153.49, 152.59, 149.03, 147.69, 145.29, 140.39, 139.74, 139.43, 138.31, 138.22, 136.69, 135.61, 130.05, 129.42, 127.74, 127.61, 127.48, 127.31, 127.15, 127.06, 125.14, 121.86, 118.50, 35.25, 30.62.ESI-MS (*m/z*): calcd. for $[M{-}BF_4^-{+}H]^{2+}$ 642.27, found 642.31.

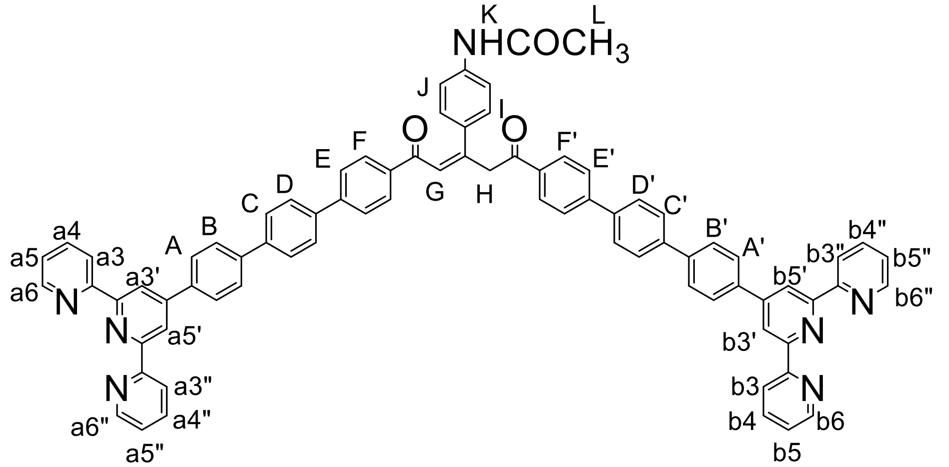

Compound 18: Compound **10** (0.61 g, 1 mmol), compound **15** (1.53 g, 3 mmol), $Pd(PPh_3)_4$ (105.6

mg, 0.1 mmol) and Na_2CO_3 (1.06 g, 10 mmol) were suspended in a mixture of dioxane (60 mL) and H_2O (20 mL) under N_2 atmosphere. The suspension was kept stirring at 75℃ for 4 hours, followed by evaporation of the solvent. The residue was dissolved in DCM, and then washed with brine water for several times. The organic phase was dried over anhydrous $MgSO_4$ and then removed under reduced pressure. Compound **18** (0.48 g, 42.3%) was obtained a pale yellow solid after purification by Al_2O_3 column with a mixture eluent of DCM and CH_3OH (100:0.8, v/v). ^{1}H NMR (500 MHz, $CDCl_3$) δ 8.81 (s, 4H, tpy-$H^{a3',a5',b3',b5'}$), 8.75 (s, 4H, tpy-$H^{a6,a6'',b6,b6''}$), 8.70 (d, J = 7.0 Hz, 4H, tpy-$H^{a3,a3'',b3,b3''}$), 8.20 (d, J = 6.7 Hz, 2H, Ph-$H^{F'}$), 8.11 (d, J = 6.8 Hz, 2H, Ph-H^{F}), 8.03 (m, 4H, Ph-$H^{A,A'}$), 7.89 (d, J = 6.3 Hz, 4H, tpy-$H^{a4,a4'',b4,b4''}$), 7.80 (d, J = 7.3 Hz, 16H, Ph-$H^{B,B',C,C',D,D',E,E'}$), 7.60 (s, 4H, Ph-$H^{I,J}$), 7.53 (s, 1H, H^{G}), 7.37 (s, 4H, tpy-$H^{a5,a5'',b5,b5''}$), 4.94 (s, 2H, H^{H}), 2.22 (s, 3H, H^{L}). 13C NMR (150 MHz, $CD_3OD/CDCl_3$ 1/4) δ 221.73, 203.14, 170.16, 156.09, 155.87, 148.87, 141.40, 141.10, 140.08, 139.27, 137.41, 127.59, 124.12, 121.89, 118.71, 23.61. ESI-MS (*m/z*): calcd. for $[M+H]^+$ 1150.44, found 1150.62; calcd. for $[M+2H]^{2+}$ 575.73, found 575.75.

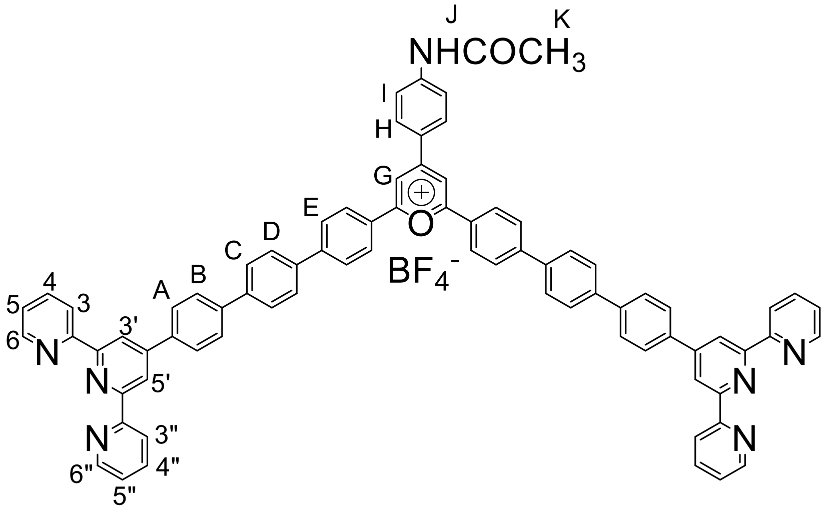

Compound 19: Compound **18** (450 mg, 0.39 mmol) was dissolved in a mixture of $CHCl_3$ (90 mL) and CH_3OH (30 mL). The suspension was stirred at room temperature until it became a clear solution. To this clear solution, HBF_4 (1 mL, 50% wt. in water) was added dropwise. After stirring at room temperature for 24 hours, the precipitate was collected by filtration and it was washed repeatedly with DCM, which gave compound **19** (415 mg) as a dark red solid, yield: 92%. ^{1}H NMR (500 MHz, DMSO-d_6) δ 10.53 (s, 1H, NH^{J}), 8.71 (s, 2H, Ph-H^{G}), 8.67 (d, J = 4.2 Hz, 4H, tpy-$H^{6,6''}$), 8.63 – 8.54 (m, 8H, tpy-$H^{3,3'',3',5'}$), 8.44 (d, J = 8.4 Hz, 2H, Ph-H^{H}), 8.35 (d, J = 7.9 Hz, 4H, Ph-H^{F}), 8.08 – 8.00 (m, 4H, tpy-$H^{4,4''}$), 7.93 – 7.85 (m, 8H, Ph-$H^{E, A}$), 7.83 – 7.70 (m, 14H, Ph-$H^{B, C, D, I}$), 7.53 – 7.47 (m, 4H, tpy-$H^{5,5''}$), 2.13 (s, 3H, H^{L}). ^{13}C NMR (150 MHz, DMSO-d_6) δ 168.28, 164.43,

155.33, 153.72, 153.43, 152.75, 150.90, 150.21, 149.67, 147.92, 147.64, 140.46, 136.17, 128.29, 128.07, 127.77, 126.21, 125.86, 122.97, 122.63, 119.73, 119.25. ESI-MS (*m/z*): calcd. for $[M-BF_4^-]^+$ 1132.43, found 1132.60; calcd. for $[M-BF_4^-+H]^{2+}$ 566.72, found 566.75.

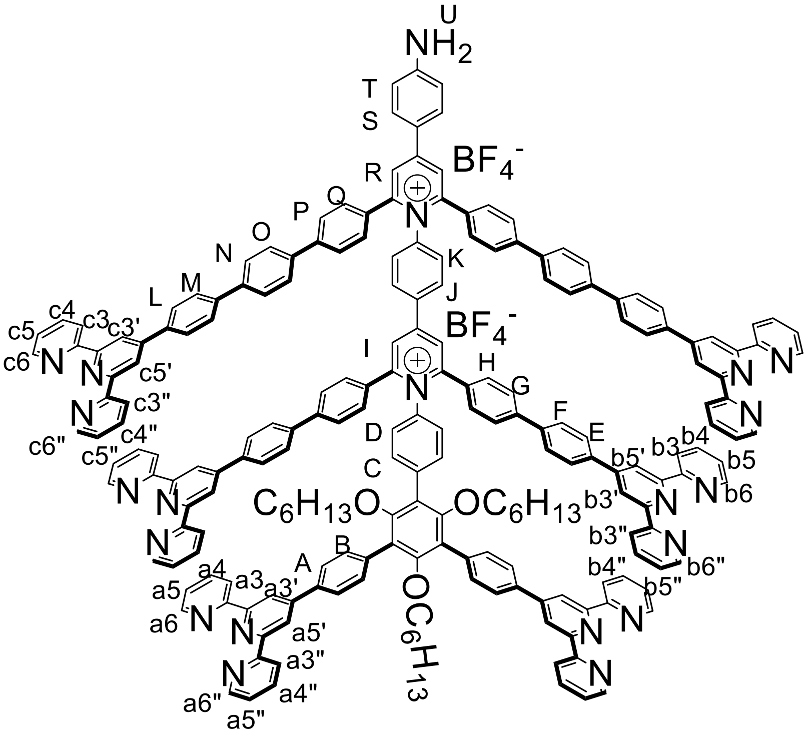

Compound 20: Compound **13** (235 mg, 0.112 mmol), compound **19** (247 mg, 0.202 mmol), and molecular sieve 4Å (50 mg) were suspended in DMSO (12 mL). The solution was stirred at 135°C for 36 hours. After cooling down to room temperature, the mixture was added into ethanol. To this suspension, HCl (9 mL, 4 mol/L) was added dropwise. The mixture was stirred at 85°C overnight. After cooling down to room temperature, $NaHCO_3$ was used to neutralize the mixture. The solvent was removed in vacuo, and the residue was extracted with DCM and washed with water for several times. The organic phase was collected and dried over anhydrous $MgSO_4$. Purification of the crude product with Al_2O_3 column gave compound **20** (DCM/CH_3OH, 100:2.4, v/v) as an orange powder. Yield: 126 mg, 28%. ^{1}H NMR (500 MHz, $CDCl_3$) δ 8.70 (s, 8H, tpy-$H^{b3',5'}$ and tpy-$H^{b3,3''}$), 8.66 (s, 4H, tpy-$H^{b6,6''}$), 8.64 (s, 4H, tpy-$H^{a3',5'}$), 8.58 (m, 20H, tpy-$H^{c3',5'}$, tpy-$H^{a6,6''}$, tpy-$H^{a3,3''}$, tpy-$H^{c6,6''}$ and tpy-$H^{c3,3''}$), 8.22 (s, 2H, Ph-H^{I}), 8.16 (s, 2H, Ph-H^{R}), 8.02 (br, 2H, Ph-H^{J}), 7.91 (m, 8H, Ph-H^{H}, tpy-$H^{b4,4''}$), 7.87 (m, 8H, Ph-$H^{A,Q}$), 7.81 (m, 10H, Ph-$H^{E,S}$, tpy-$H^{b4,4''}$), 7.75 (m, 6H, Ph-H^{C}, tpy-$H^{c4,4''}$), 7.64 (br, 18H, Ph-$H^{D,L,M,N,O}$), 7.56 (m, 10H, Ph-$H^{F,K,P}$), 7.51 (m, 8H, Ph-$H^{G,B}$), 7.25 – 7.18 (m, overlapped with $CHCl_3$, 12H, tpy-$H^{a5,5''}$, tpy-$H^{b5,5''}$ and tpy-$H^{c5,5''}$), 6.84 (br, 2H, Ph-H^{T}), 5.17 (s, 2H, U), 3.18 (s, 2H), 2.88 (s, 4H), 0.98 (dd, J = 14.6, 7.2 Hz, 7H), 0.79 (m, 12H), 0.69 (m, 6H), 0.64 – 0.41 (m, 19H). ^{13}C NMR (125 MHz, $CDCl_3$) δ 156.22, 155.97, 155.79, 155.09, 150.02,

149.05, 139.37, 138.24, 137.01, 136.68, 135.35, 131.44, 127.86, 127.28, 126.60, 126.12, 123.64, 121.18, 118.79, 118.50, 73.63, 73.23, 31.29, 29.72, 29.49, 25.31, 25.21, 22.54, 22.42, 13.92, 13.85. HR ESI-MS (*m/z*): calcd. for $[M-2BF_4^-+H]^{3+}$ 1025.7827, found 1025.7850; calcd. for $[M-2BF_4^-+2H]^{4+}$ 769.5890, found 769.5920; calcd. for $[M-2BF_4^-+3H]^{5+}$ 615.8728, found 615.8691.

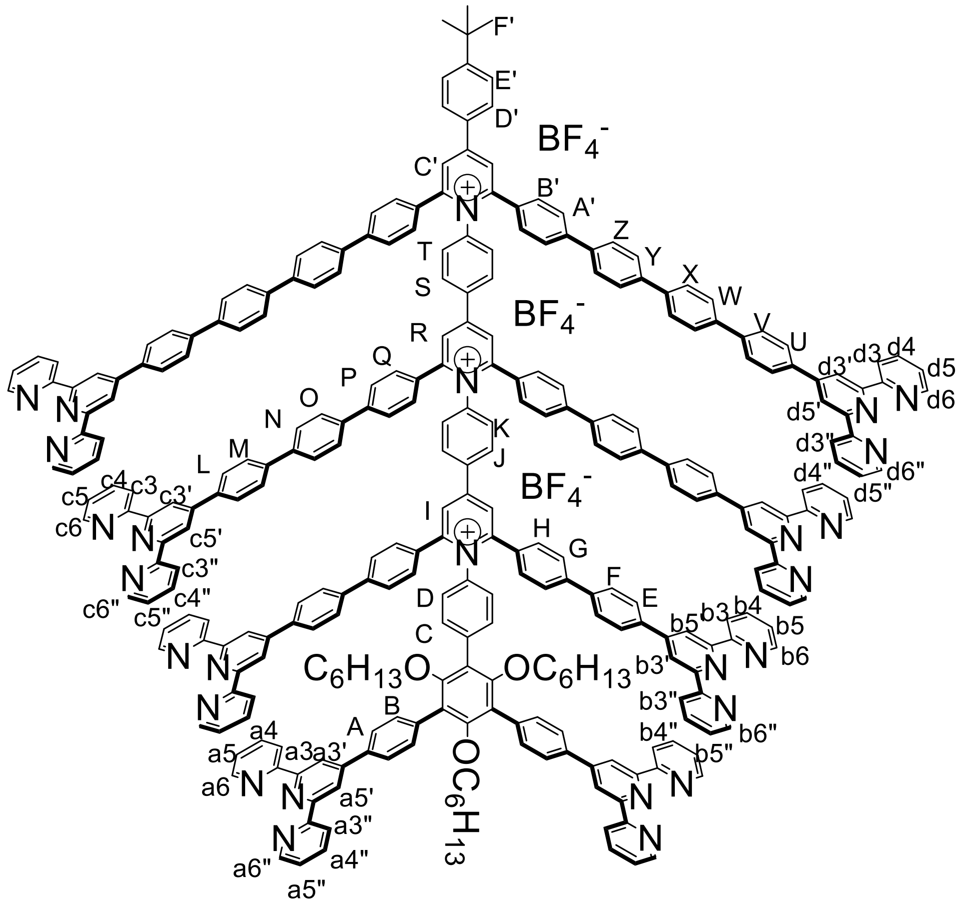

Ligand L4: compound **20** (120 mg, 37 μmmol), compound **17** (100 mg, 74 μmmol) and molecular sieve 4Å (10 mg) were suspended in DMSO (20 mL). The mixture was heated to 135°C and kept stirring for 36 hours. After cooling down to temperature, DCM was added to dissolve the mixture. The solution was washed with brine water for 3 times. Ligand **L4** was obtained as a yellow powder after purification on Al_2O_3 column with eluent DCM/CH_3OH, 100:1.8, v/v. Yield: 38 mg, 22%.^{1}H NMR (500 MHz, $CDCl_3$) δ 8.68 (br, 8H, tpy-$H^{c3',5'}$ and tpy-$H^{c3,3''}$), 8.59 (m, tpy-$H^{a3',5'}$, tpy-$H^{b3',5'}$, tpy-$H^{d3',5'}$, tpy-$H^{c6,6''}$, tpy-$H^{b6,6''}$, tpy-$H^{b3,3''}$, and Ph-H^{R}), 8.52 (br, 18H, tpy-$H^{a3,3''}$, tpy-$H^{d3,3''}$, tpy-$H^{a6,6''}$, tpy-$H^{d6,6''}$, and Ph-$H^{C'}$), 8.26 (s, 2H, Ph-H^{I}), 8.01 (br, 2H, Ph-H^{J}), 7.94 (m, 4H, Ph-H^{H}), 7.93 – 7.72 (m, 42H, Ph-$H^{A,E,J,L,Q,T,B',D'}$, tpy-$H^{a4,4''}$ tpy-$H^{b4,4''}$ tpy-$H^{c4,4''}$ and tpy-$H^{d4,4''}$), 7.71 – 7.42 (m, 62, Ph-$H^{B,C,D,F,G,J,M,N,O,P,S,U,V,W,X,Y,Z,A'}$), 7.37 (br, 2H, Ph-$H^{E'}$) 7.21 (m overlapped with C*H*Cl_3, 16H, tpy-$H^{a5,5''}$, tpy-$H^{b5,5''}$, tpy-$H^{c5,5''}$, tpy-$H^{d5,5''}$), 3.15 (s, 2H), 2.84 (s, 4H), 1.37 (s, 9H, F'), 0.97 (m,

10.6 Hz, 6H), 0.81 (m, 14H), 0.60 – 0.41 (m, 18H). ^{13}C NMR (150 MHz, $CDCl_3$) δ 156.19, 155.99, 155.95, 155.75, 155.05, 149.01, 140.21, 139.96, 139.30, 138.28, 137.77, 137.30, 136.66, 131.39, 127.84, 127.58, 127.27, 126.83, 126.71, 126.56, 126.13, 123.60, 121.15, 118.77, 118.54, 118.41, 73.60, 73.17, 31.27, 31.05, 29.62, 29.45, 25.29, 25.17, 22.50, 22.38, 13.87, 13.81. HR ESI-MS (*m/z*): calcd. for $[M-3BF_4^-]^{3+}$ 1447.2892, found 1447.2853; $[M-3BF_4^-+H]^{4+}$ 1085.7189, found 1085.7272; $[M-3BF_4^-+2H]^{5+}$ 868.7766, found 868.7776; $[M-3BF_4^-+3H]^{6+}$ 724.1459, found 724.1483.

3. Synthesis of the complexes G1−G4

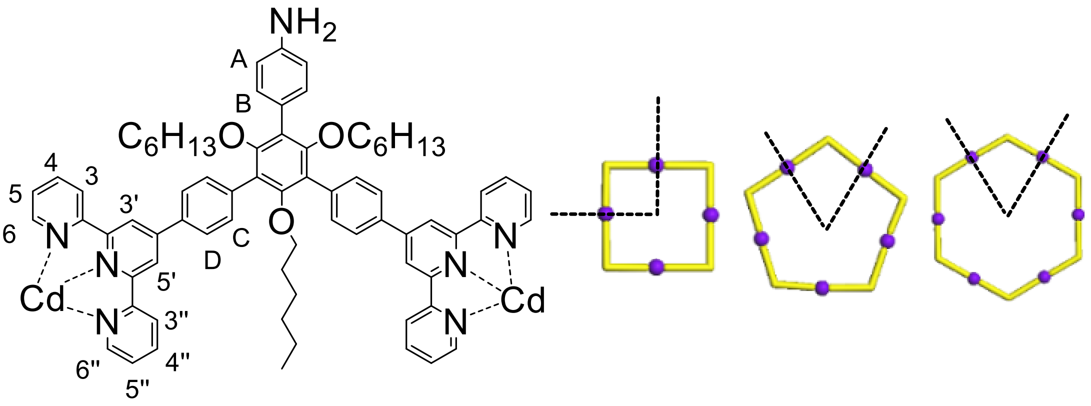

G1: To a solution of ligand **L1** (5.0 mg, 4.6 μmol) in $CHCl_3$ (1.5 mL), a solution of $Cd(NO_3)_2{\cdot}4H_2O$ (1.4 mg, 4.6μmol) in MeOH (4.5 mL) was added, and then the mixture was stood at 50 ºC for 3 h. After cooling to room temperature, 60 mg of NH_4PF_6 was added and brown precipitate was observed. The precipitate was washed by distilled water and the final product was obtained with a yield of 90%. ^{1}H NMR (400 MHz, CD_3CN) δ 9.01 (s, 4H, tpy-$H^{3',5'}$), 8.81 (d, J = 7.3 Hz, 4H, tpy-$H^{3,3''}$), 8.26 (m, J = 14.0 Hz, 8H, Ph-H^{D} and tpy-$H^{4,4''}$), 8.14 (br, 4H, tpy-$H^{6,6''}$), 7.86 (d, J = 5.6 Hz, 4H, Ph-H^{C}), 7.53 (br, 4H, tpy-$H^{6,6''}$), 7.27 (d, J = 7.1 Hz, 2H, Ph-H^{B}), 6.76 (d, J = 7.2 Hz, 2H, Ph-H^{A}), 4.26 (m, 2H), 3.37 (m, 4H), 1.95 (br, 4H), 1.15 (br, 13H), 0.95 (br, 12H), 0.76 (t, J = 6.6 Hz, 6H), 0.68 (br, 3H). ^{13}C NMR (100 MHz, CD_3CN) δ 174.51, 149.68, 141.26, 132.20, 131.40, 127.20, 127.00, 125.57, 123.63, 121.45, 113.77, 113.01, 72.94, 31.11, 29.42, 25.16, 22.34, 13.39.ESI-MS (*m/z*): 1342.0 $[M3-3PF_6^-]^{3+}$ (calcd *m/z*: 1342.0), 970.2 $[M3-4PF_6^-]^{4+}$ (calcd *m/z*: 970.1), 747.1 $[M3-5PF_6^-]^{5+}$ (calcd *m/z*: 747.1), 598.4 $[M3-6PF_6^-]^{6+}$ (calcd *m/z*: 598.4); 1342.0 $[M4-4PF_6^-]^{4+}$ (calcd *m/z*: 1342.0), 1044.7 $[M4-5PF_6^-]^{5+}$ (calcd *m/z*: 1044.6), 846.5 $[M4-6PF_6^-]^{6+}$ (calcd *m/z*: 846.6), 704.8 $[M4-7PF_6^-]^{7+}$ (calcd *m/z*: 704.8), 598.4 $[M4-8PF_6^-]^{8+}$ (calcd *m/z*: 598.4); 1342.0 $[M5-5PF_6^-]^{5+}$ (calcd *m/z*: 1342.0), 1094.0 $[M5-6PF_6^-]^{6+}$ (calcd *m/z*: 1094.0), 917.1 $[M5-7PF_6^-]^{7+}$ (calcd *m/z*: 917.0), 784.3 $[M5-8PF_6^-]^{8+}$ (calcd *m/z*: 784.3), 681.0 $[M5-9PF_6^-]^{9+}$ (calcd *m/z*: 681.0), 598.4 $[M5-10PF_6^-]^{10+}$ (calcd *m/z*: 598.4); 1342.0 $[M6-PF_6^-]^{6+}$ (calcd *m/z*: 1342.0), 1129.5 $[M6-7PF_6^-]^{7+}$ (calcd *m/z*: 1129.5), 970.2 $[M6-8PF_6^-]^{8+}$ (calcd *m/z*: 970.1), 846.5 $[M6-9PF_6^-]^{9+}$ (calcd *m/z*: 846.5), 747.1 $[M6-10PF_6^-]^{10+}$ (calcd *m/z*: 747.1), 666.1 $[M6-11PF_6^-]^{11+}$ (calcd *m/z*: 666.1); Macrocycle complexes are named Mn^{x+}, where M designates the repeat unit <tpy-Cd-tpy>, n is the number of repeat units, and x is the number of charges.

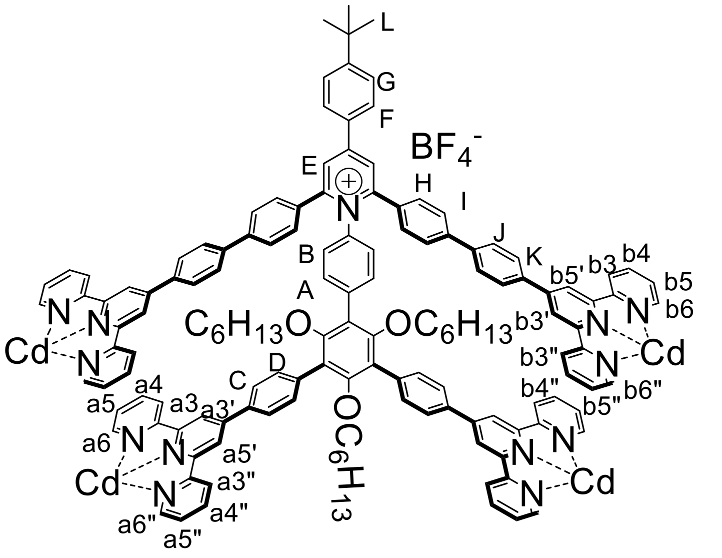

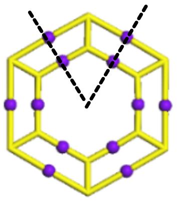

G2: To a solution of ligand **L2** (10.7 mg, 5.0 μmol) in $CHCl_3$ (2.0 mL), a solution of $Cd(NO_3)_2{\cdot}4H_2O$ (3.1 mg, 10.0 μmol) in MeOH (6 mL) was added, and then the mixture was kept at 50 ºC for 3 h. After cooling to room temperature, 100 mg of NH_4PF_6 was added and yellow precipitate was observed. The precipitate was washed by water, and 13.2 mg yellow solid was obtained, yield: 88%. ^{1}H NMR (600 MHz, CD_3CN) δ 8.93 (s, 4H, tpy-$H^{b3',5'}$), 8.91 (s, 4H, tpy-$H^{a3',5'}$), 8.75 – 8.62 (m, 8H, tpy-$H^{a3,3''}$ and tpy-$H^{b3,3''}$), 8.56 (s, 2H, Ph-H^{E}), 8.29 (d, J = 8.2 Hz, 4H, Ph-H^{K}), 8.21 (m, 6H, Ph-$H^{D,F}$), 8.11 (d, J = 7.7 Hz, 4H, Ph-H^{J}), 8.04 – 7.96 (m, 12H, tpy-$H^{a6,6''}$, tpy-$H^{b6,6''}$, Ph-H^{I}), 7.90 (m, 8H, tpy-$H^{a4,4''}$and tpy-$H^{b4,4''}$), 7.81 (d, J = 8.3 Hz, 2H, Ph-H^{G}), 7.79 – 7.71 (m, 8H, Ph-$H^{H,C}$), 7.67 (d, J = 7.3 Hz, 2H, Ph-H^{B}), 7.56 (d, J = 7.1 Hz, 2H, Ph-H^{A}), 7.16 (m, 4H, tpy-$H^{b5,5''}$), 7.12 (m, 4H, tpy-$H^{a5,5''}$), 3.37 (br, 2H), 3.05 (br, 4H), 1.46 (s, 9H, L), 1.06 (m, 4H), 0.89 (m, 10H), 0.70 (br, 4H), 0.65 (t, J = 7.2 Hz, 4H), 0.52 (t, J = 7.3 Hz, 12H). 13C NMR (150 MHz, CD3CN) δ 156.70, 156.24, 155.52, 155.27, 154.72, 154.33, 149.77, 149.19, 148.26, 140.75, 140.30, 137.80, 137.43, 135.83, 134.48, 132.84, 131.91, 131.28, 130.51, 130.46, 128.52, 128.23, 127.55, 127.02, 126.74, 126.48, 125.55, 123.94, 123.30, 122.82, 121.43, 121.24, 73.32, 34.61, 30.96, 30.70, 29.95, 29.37, 28.91, 25.13, 24.69, 22.52, 22.45, 21.97, 13.41, 13.03. ESI-MS (*m/z*): 1652.8 $[M-10PF_6^-]^{10+}$ (calcd *m/z*: 1652.8), 1489.4 $[M-11PF_6^-]^{11+}$ (calcd *m/z*: 1489.4), 1353.2 $[M-12PF_6^-]^{12+}$ (calcd *m/z*: 1353.2), 1237.9 $[M-13PF_6^-]^{13+}$ (calcd *m/z*: 1237.9), 1139.1 $[M-14PF_6^-]^{14+}$ (calcd *m/z*: 1139.1), 1053.5 $[M-15PF_6^-]^{15+}$ (calcd *m/z*: 1053.5), 978.6 $[M-16PF^{-}]^{16+}$ (calcd *m/z*: 978.6), 912.5 $[M-17PF_6^-]^{17+}$ (calcd *m/z*: 912.5), 853.7 $[M-18PF_6^-]^{18+}$ (calcd *m/z*: 853.7), 801.2 $[M-19PF^{-}]^{19+}$ (calcd *m/z*: 801.2), 753.9 $[M-20PF_6^-]^{20+}$ (calcd *m/z*: 753.9), 711.1 $[M-21PF_6^-]^{21+}$ (calcd *m/z*: 711.1), 672.2 $[M-22PF^{-}]^{22+}$ (calcd *m/z*: 672.2), 636.7 $[M-23PF_6^-]^{23+}$ (calcd *m/z*: 636.7), 604.1 $[M-24PF_6^-]^{24+}$ (calcd *m/z*: 604.1).

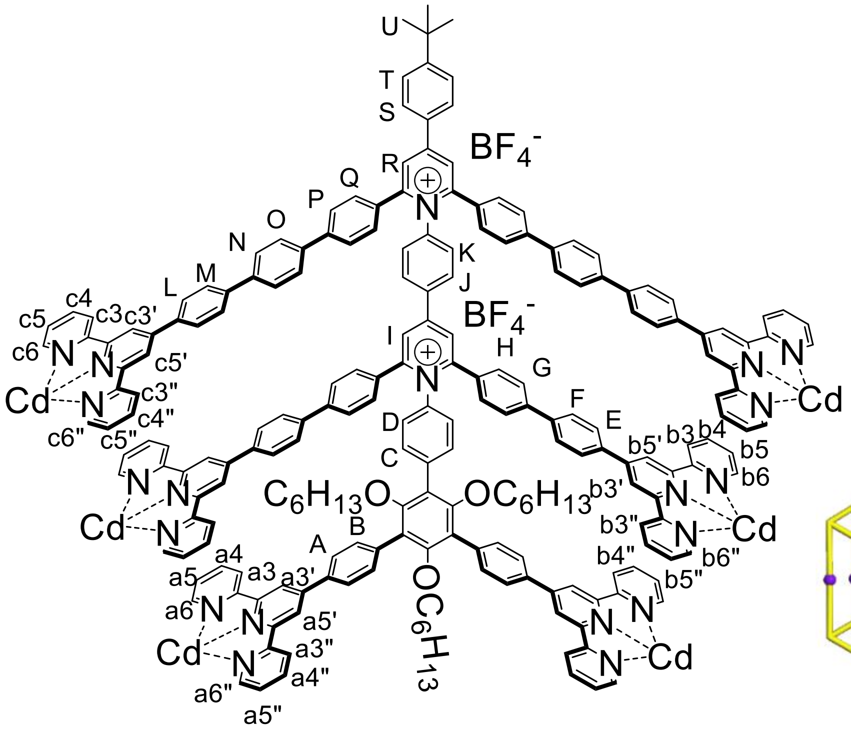

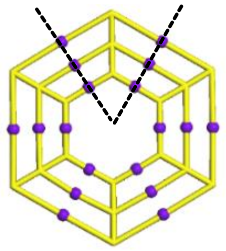

G3: To a solution of ligand **L3** (7.8 mg, 2.5 μmol) in $CHCl_3$ (2.0 mL), a solution of $Cd(NO_3)_2{\cdot}4H_2O$ (2.3 mg, 7.5 μmol) in MeOH (6 mL) was added, and then the mixture was kept at 50 ºC for 3 h. After cooling to room temperature, 80 mg of NH_4PF_6 was added and yellow precipitate was observed. The precipitate was washed by water, and 9.8 mg yellow solid was obtained, yield: 85%. ^{1}H NMR (500 MHz, CD_3CN) δ 8.98 (s, 4H, tpy-$H^{b3',5'}$), 8.86 (br, 10H, tpy-$H^{a3',5'}$, tpy-$H^{c3',5'}$, Ph-H^{I}), 8.67 (d, J = 7.6 Hz, 4H, tpy-$H^{b3,3''}$), 8.62 (br, 6H, tpy-$H^{a3,3''}$, Ph-H^{R}), 8.52 (d, J = 7.3 Hz, 4H, tpy-$H^{c3,3''}$), 8.30 (m, 4H, Ph-H^{E}), 8.23 (m, 8H, Ph-$H^{K,S,L}$), 8.18 (m, 4H, Ph-H^{A}), 8.10 (m, 4H, Ph-H^{F}), 7.95−8.07 (m, 20 H, tpy-$H^{b6,6''}$ and Ph-$H^{J,M,G,H}$) 7.90−7.95 (m, 16H, tpy-$H^{b4,4''}$, tpy-$H^{a6,6''}$ and Ph-$H^{P,O}$), 7.86 (m, 4H, tpy-$H^{a4,4''}$), 7.81 (br, 6H, tpy-$H^{c6,6''}$ and Ph-H^{T}), 7.69 (br, 8H, Ph-$H^{B,N}$), 7.63 (br, 6H, Ph-$H^{D,Q}$), 7.58 (m, 4H, tpy-$H^{c4,4''}$), 7.51 (m, 2H, Ph-H^{C}), 6.98−7.18 (m, 8*H*, tpy-$H^{b5,5''}$ and tpy-$H^{a5,5''}$), 6.53 (br, 4H, tpy-$H^{c5,5''}$), 3.30 (br, 2H), 3.01 (br, 4H), 1.47 (s, 9H, U), 1.10 – 0.97 (m, 7H), 0.95 – 0.76 (m, 14H), 0.66 (m, 10H), 0.58 – 0.35 (m, 17H). ^{13}C NMR (150 MHz, CD_3CN) δ 156.48, 154.32, 152.81, 152.54, 152.09, 151.66, 151.23, 149.69, 148.30, 140.64, 133.24, 132.10, 131.75, 130.47, 128.40, 127.44, 127.01, 126.74, 123.36, 121.49, 112.57, 111.29, 73.40, 30.92, 30.67, 29.95, 29.33, 28.86, 25.06, 24.65, 22.48, 21.94, 13.30, 12.98. ESI-MS (*m/z*): 1702.8 $[M-15PF_6^-]^{15+}$ (calcd *m/z*: 1702.8), 1586.3 $[M-16PF_6^-]^{16+}$ (calcd *m/z*: 1586.3), 1484.6 $[M-17PF_6^-]^{17+}$ (calcd *m/z*: 1484.6), 1394.0 $[M-18PF_6^-]^{18+}$ (calcd *m/z*: 1394.0), 1313.0 $[M-19PF_6^-]^{19+}$ (calcd *m/z*: 1313.0), 1240.1 $[M-20PF_6^-]^{20+}$ (calcd *m/z*: 1240.1), 1174.1 $[M-21PF^{-}]^{21+}$ (calcd *m/z*: 1174.1), 1114.1

[M−22PF$_6^-$]$^{22+}$ (calcd *m/z*: 1114.1), 1059.4 [M−23PF$_6^-$]$^{23+}$ (calcd *m/z*: 1059.4), 1009.2 [M−24PF^{-}]$^{24+}$ (calcd *m/z*: 1009.2), 963.0 [M−25PF$_6^-$]$^{25+}$ (calcd *m/z*: 963.0), 920.5 [M−26PF$_6^-$]$^{26+}$ (calcd *m/z*: 920.5),

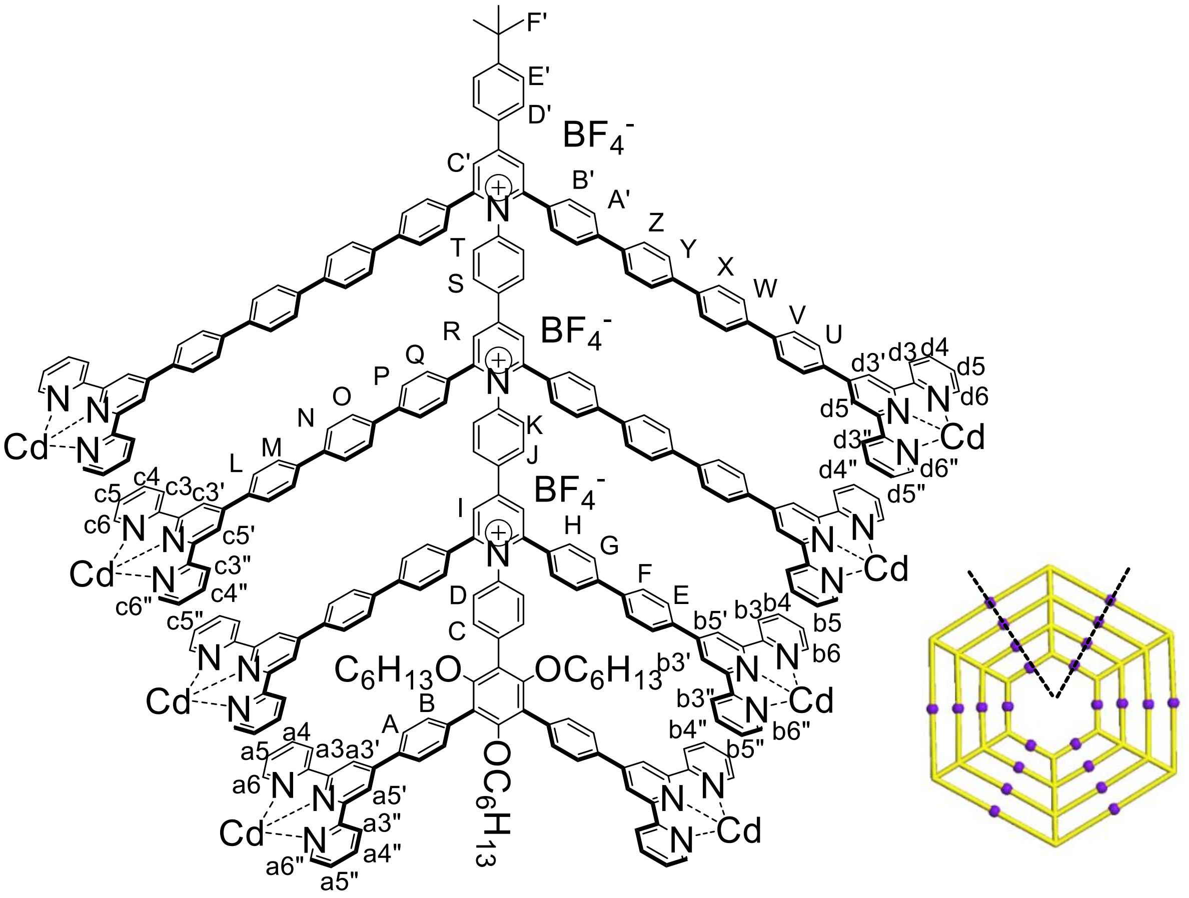

G4: To a solution of ligand **L4** (9.2 mg, 2.0 μmol) in $CHCl_3$ (9.0 mL), a solution of $Cd(NO_3)_2 \cdot 4H_2O$ (2.5 mg, 8.0 μmol) in MeOH (27 mL) was added, and then the mixture was kept at 50 ºC for 3 h. After cooling to room temperature, 80 mg of NH_4PF_6 was added and yellow precipitate was observed. The precipitate was washed by water, and 10.5 mg yellow solid was obtained, yield: 82%. ^{1}H NMR (500 MHz, DMSO-*d*$_6$) δ 8.88 (s, 2H, Ph-H^{R}), 8.67 (br, 12H, tpy-$H^{a3',5'}$, tpy-$H^{b3',5'}$and tpy-$H^{c3',5'}$), 8.64 – 8.57 (m, 16H, Ph-$H^{I,C'}$, tpy-$H^{d3',5'}$, tpy-$H^{b3,3''}$, and tpy-$H^{c3,3''}$), 8.54 (br, 16H, Ph-$H^{E,L}$, tpy-$H^{b3,3''}$, and tpy-$H^{d3,3''}$), 8.46 (br, 4H, Ph-H^{U}), 8.40 (d, *J* = 8.4 Hz, 2H, Ph-H^{T}), 8.32 (m, 4H, Ph-$H^{K,D}$), 7.96 (m, 28H, Ph-$H^{A,H,Q,B'}$, tpy-$H^{a4,4''}$, tpy-$H^{d4,4''}$, tpy-$H^{a6,6''}$), 7.89 – 7.79 (m, 36H, Ph-$H^{B,G,J,N,P,W,A',D'}$, tpy-$H^{b4,4''}$and tpy-$H^{c4,4''}$), 7.79 – 7.68 (m, 20H, Ph-$H^{C,S,Y,Z}$, tpy-$H^{b6,6''}$ and tpy-$H^{c6,6''}$), 7.57 – 7.63 (m, 12H, Ph-$H^{O,X}$ and tpy-$H^{d6,6''}$), 7.53 (m, 6H, Ph-$H^{E'}$ and tpy-$H^{c5,5''}$), 7.48 (br, 4H, tpy-$H^{b5,5''}$), 7.43 (m, 20H, Ph-$H^{M,F,V}$, tpy-$H^{a5,5''}$ and tpy-$H^{d5,5''}$), 2.71 (s, 4H), 1.38 (s, 9H, F'), 1.22 (s, 2H), 0.91 (s, 2H), 0.87 – 0.74 (m, 5H), 0.68 (s, 4H), 0.59 – 0.30 (m, 10H), 0.13 (s, 12H). ^{13}C

NMR (125 MHz, DMSO-d_6) δ 156.56, 155.59, 155.29, 154.60, 151.77, 149.67, 142.56, 142.20, 141.79, 141.36, 141.14, 141.03, 140.53, 140.27, 139.84, 138.90, 138.71, 138.23, 137.97, 137.66, 137.30, 131.27, 130.44, 129.80, 129.46, 127.88, 126.76, 121.44, 118.33, 73.42, 31.32, 31.04, 29.77, 29.46, 25.43, 25.07, 22.67, 22.37, 14.06. ESI-MS (*m/z*): 1680.4 $[M-21PF_6^-]^{21+}$ (calcd *m/z*: 1680.6), 1597.7 $[M-22PF_6^-]^{22+}$ (calcd *m/z*: 1597.7), 1521.9 $[M-23PF_6^-]^{23+}$ (calcd *m/z*: 1521.9), 1452.4 $[M-24PF_6^-]^{24+}$ (calcd *m/z*: 1452.4), 1388.5 $[M-25PF_6^-]^{25+}$ (calcd *m/z*: 1388.5), 1329.6 $[M-26PF_6^-]^{26+}$ (calcd *m/z*: 1329.6), 1275.0 $[M-27PF_6^-]^{27+}$ (calcd *m/z*: 1274.9), 1224.2 $[M-28PF_6^-]^{28+}$ (calcd *m/z*: 1224.2), 1177.0 $[M-29PF_6^-]^{29+}$ (calcd *m/z*: 1177.0), 1132.9 $[M-30PF_6^-]^{30+}$ (calcd *m/z*: 1132.9), 1091.7 $[M-31PF_6^-]^{31+}$ (calcd *m/z*: 1091.7), 1053.1 $[M-32PF_6^-]^{32+}$ (calcd *m/z*: 1053.1)

4. ^{1}H NMR, ^{13}C NMR, 2D COSY NMR, 2D NOESY NMR, and ESI-MS spectra of ligands L1−L4

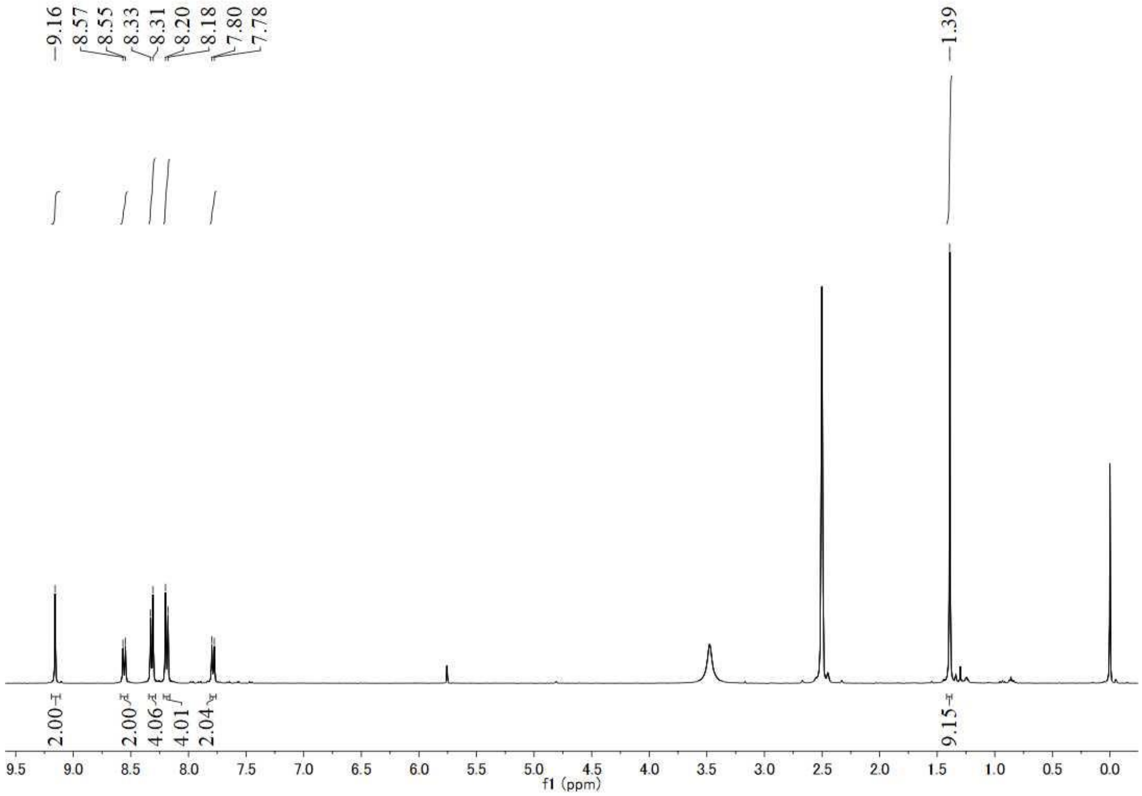

Supplementary Figure 3. ^{1}H NMR (500 MHz, DMSO-d_6, 300 K) spectrum of compound **4.**

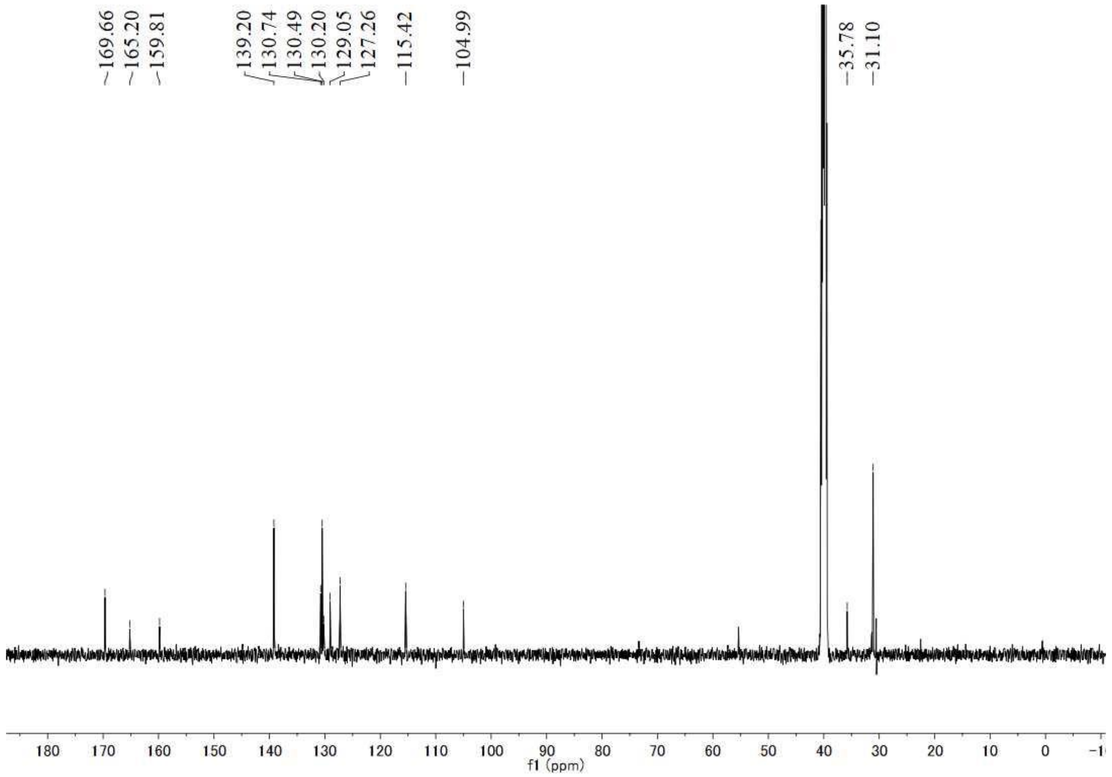

Supplementary Figure 4. ^{13}C NMR (125 MHz, DMSO-d_6, 300 K) spectrum of compound **4.**

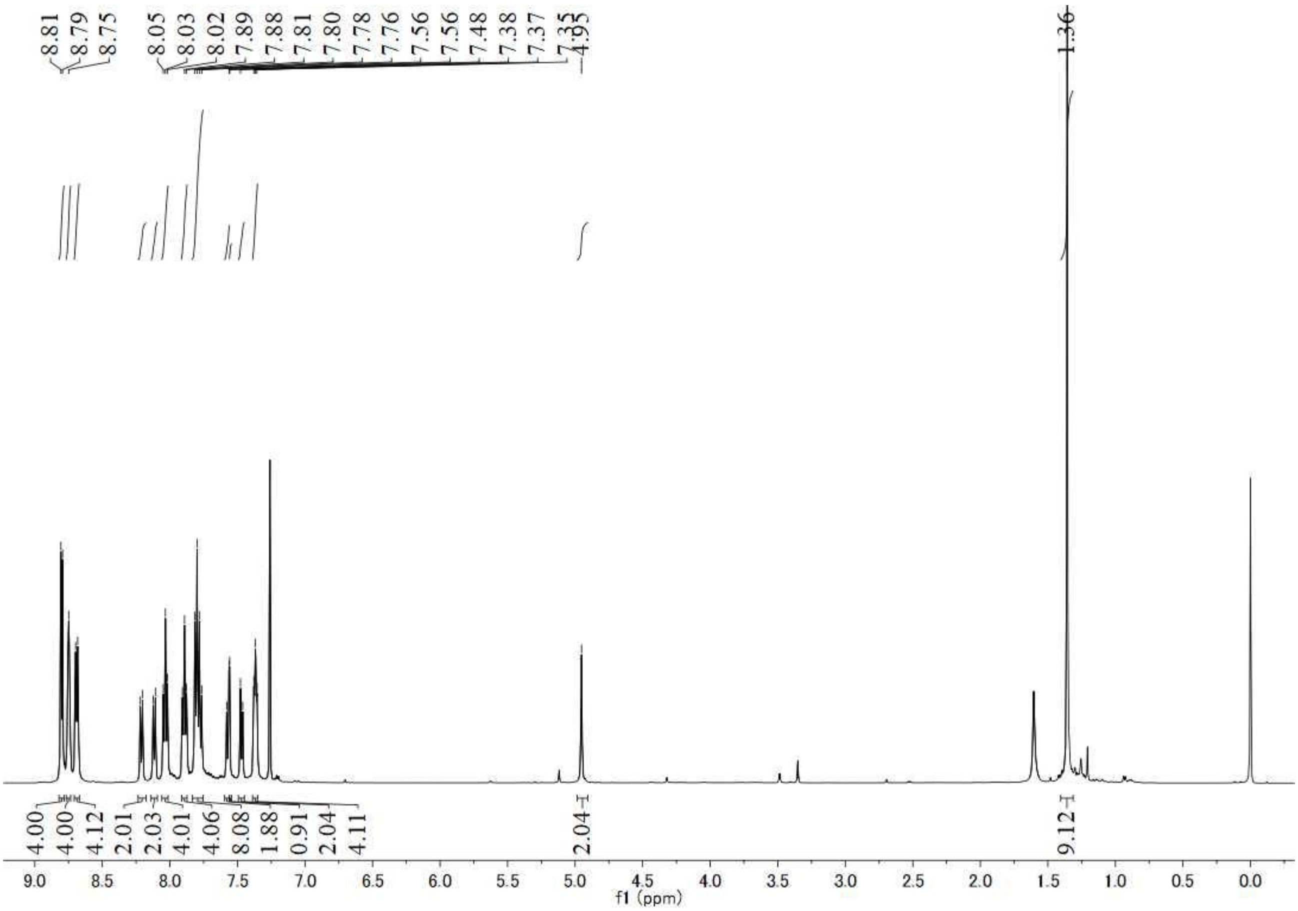

Supplementary Figure 5. ^{1}H NMR (500 MHz, $CDCl_3$, 300 K) spectrum of compound **5.**

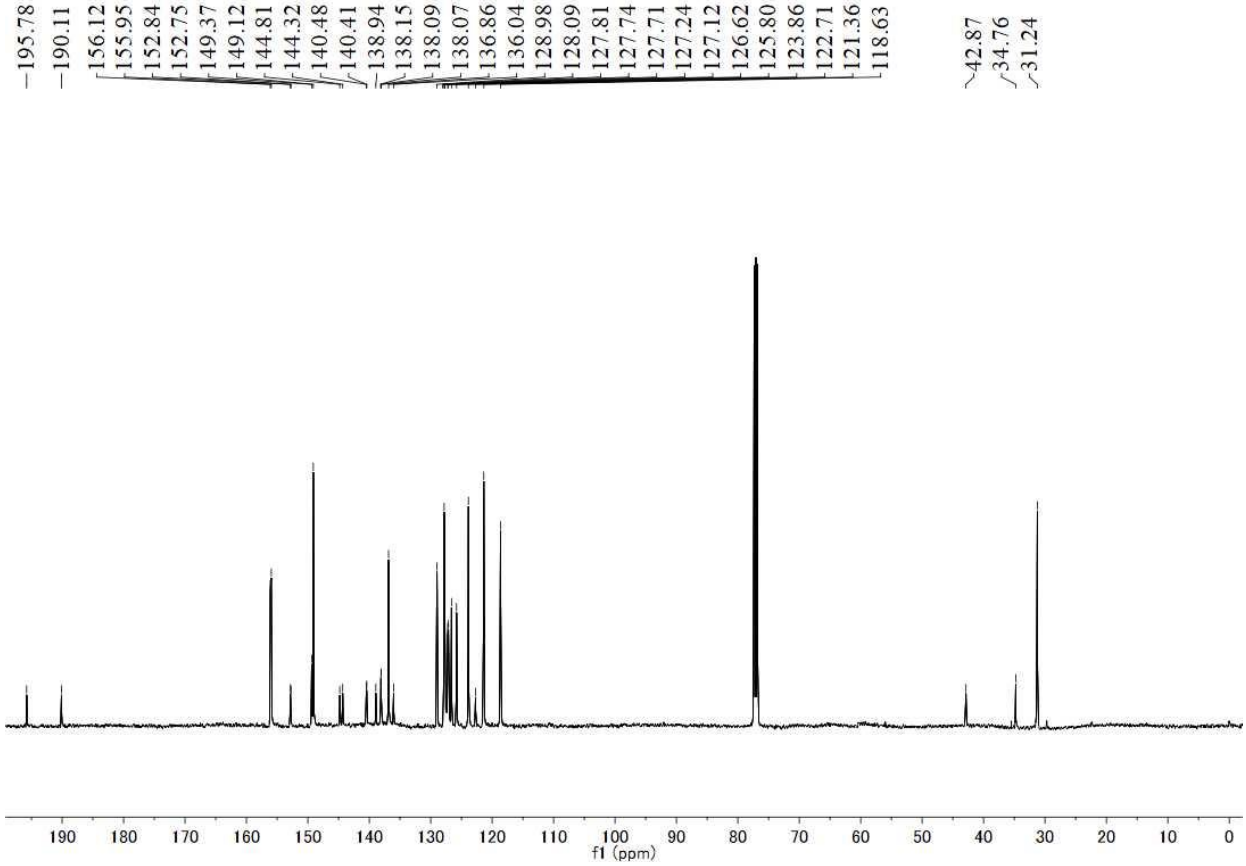

Supplementary Figure 6. ^{13}C NMR (125 MHz, $CDCl_3$, 300 K) spectrum of compound **5.**

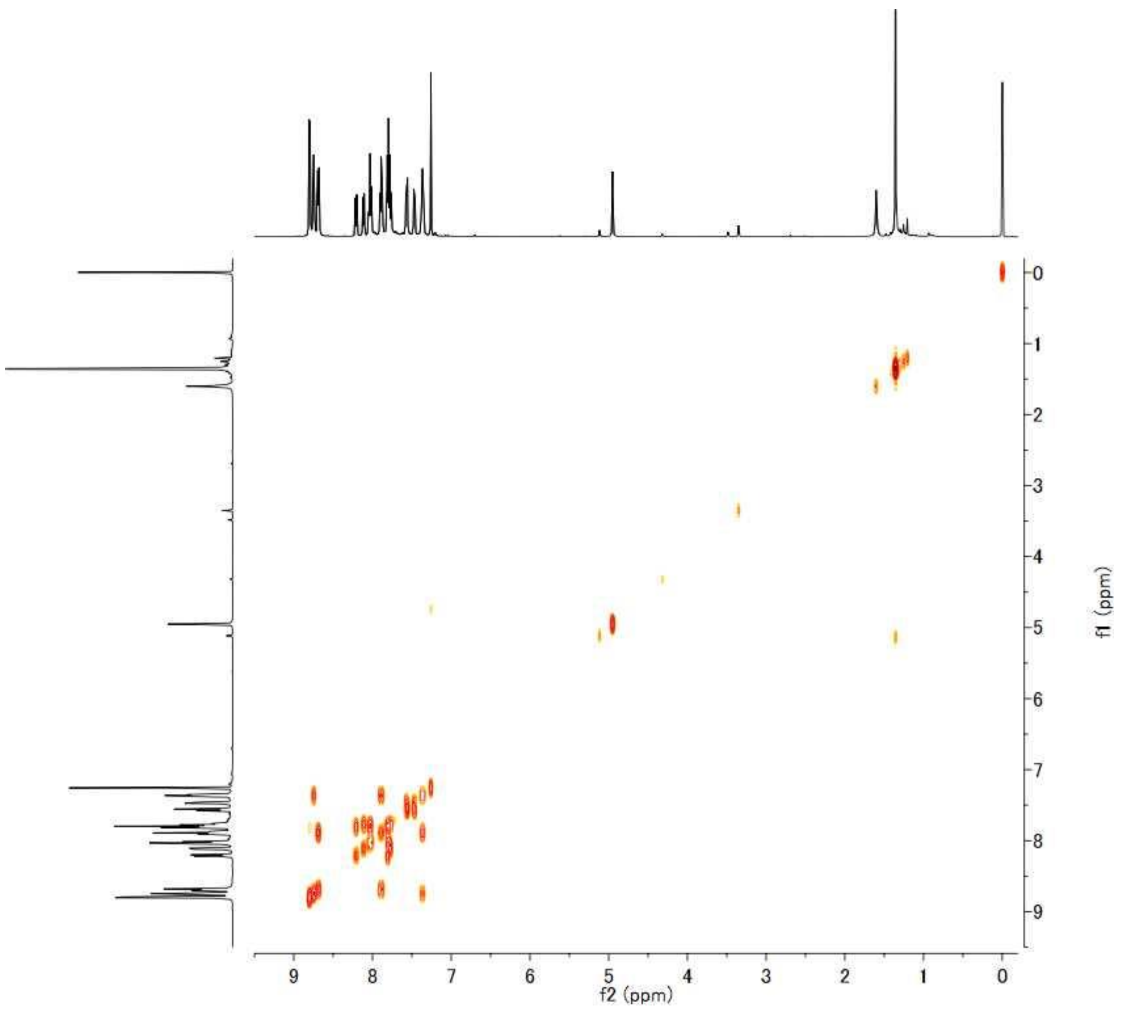

Supplementary Figure 7. 2D COSY NMR (500 MHz, $CDCl_3$, 300 K) spectrum of compound **5.**

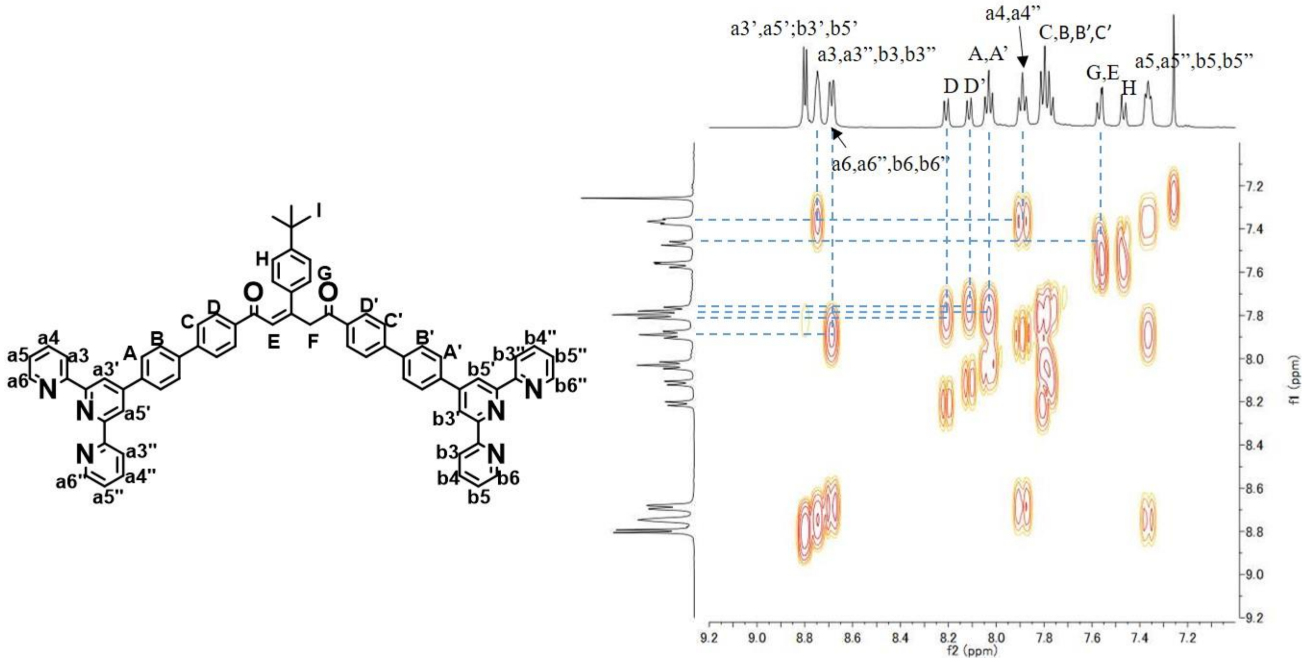

Supplementary Figure 8. 2D COSY NMR (500 MHz, $CDCl_3$, 300 K) spectrum (aromic region) of compound **5.**

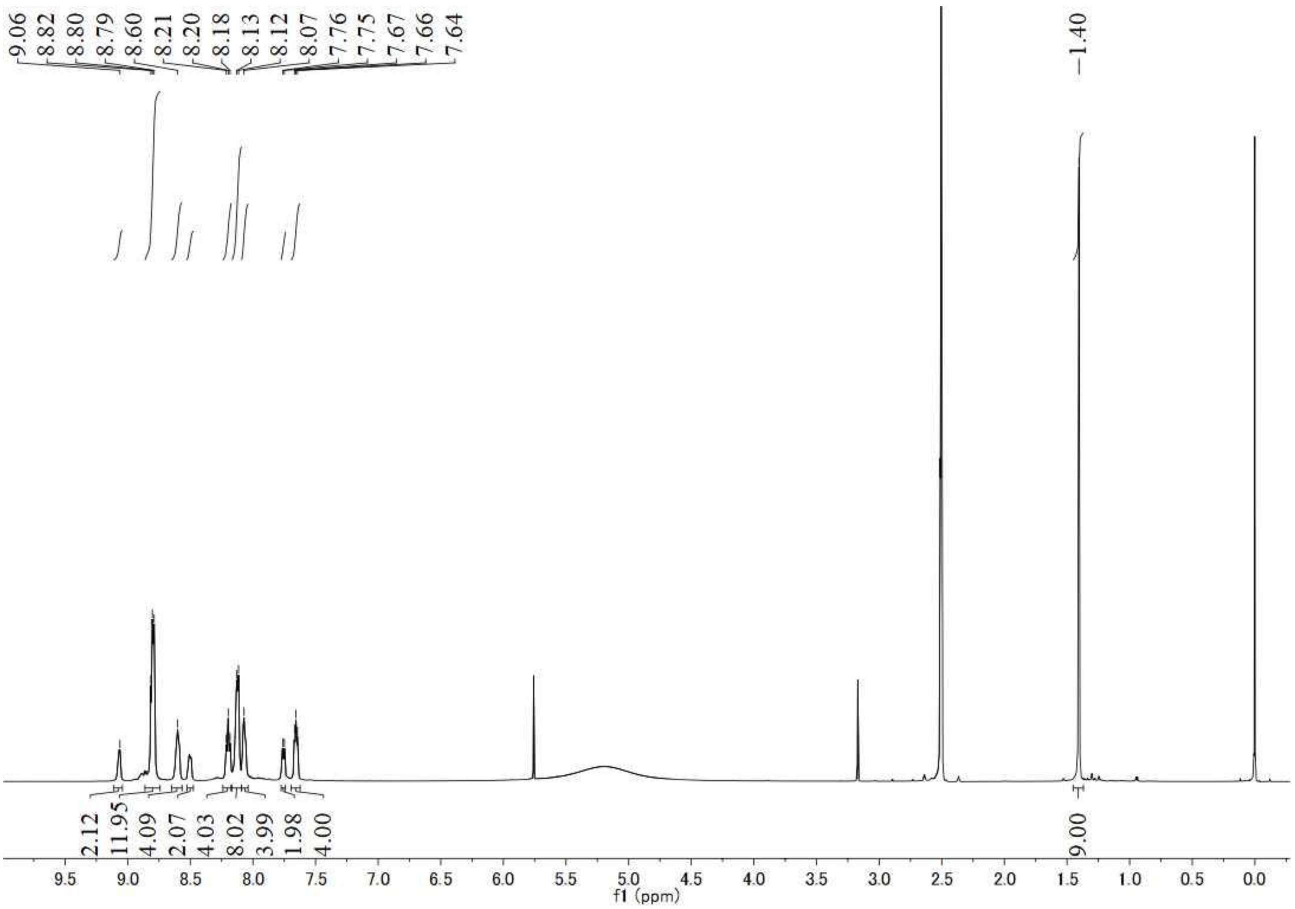

Supplementary Figure 9. ^{1}H NMR (500 MHz, DMSO-d_6, 300 K) spectrum of compound **6.**

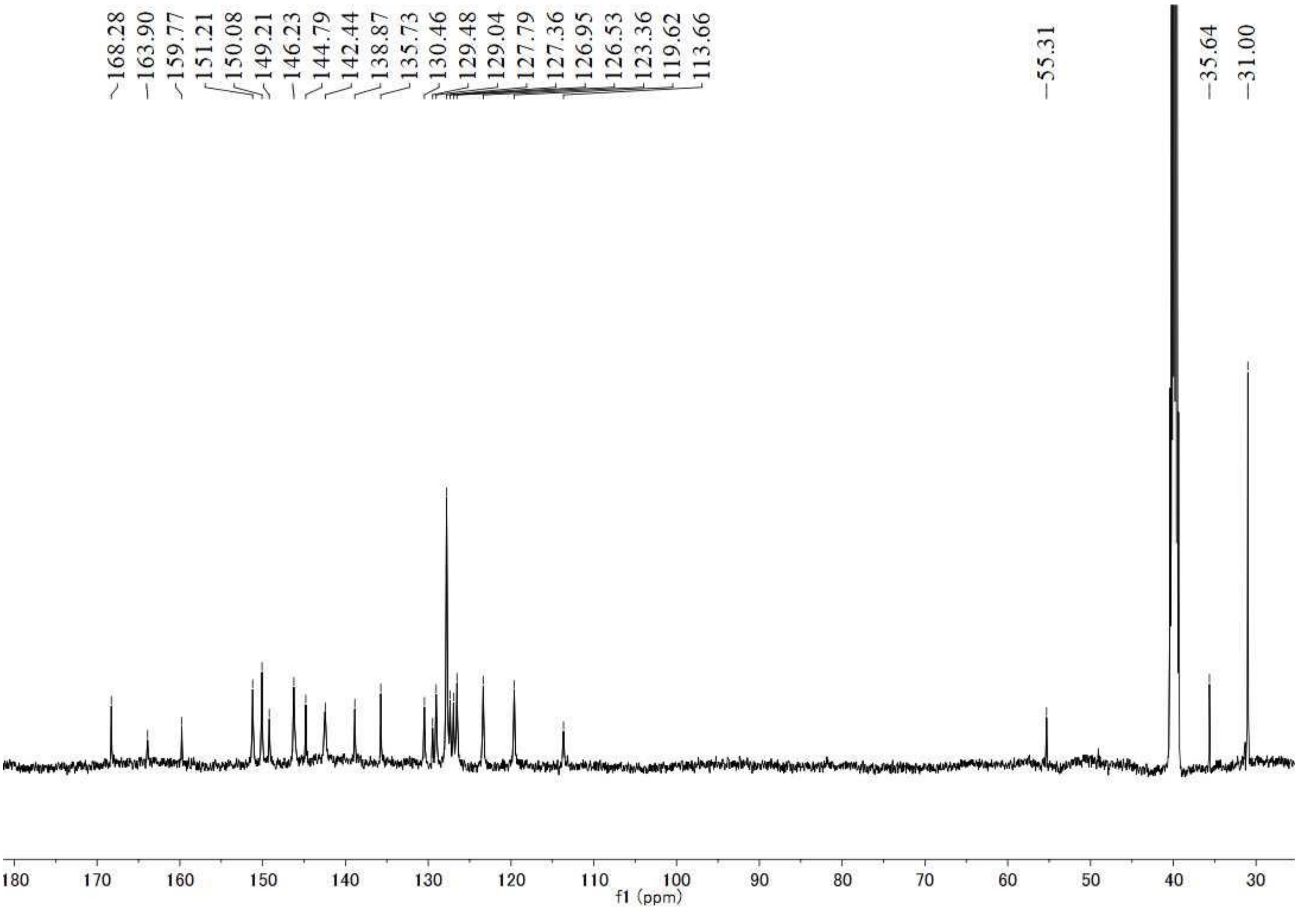

Supplementary Figure 10. ^{13}C NMR (125 MHz, DMSO-d_6, 300 K) spectrum of compound **6.**

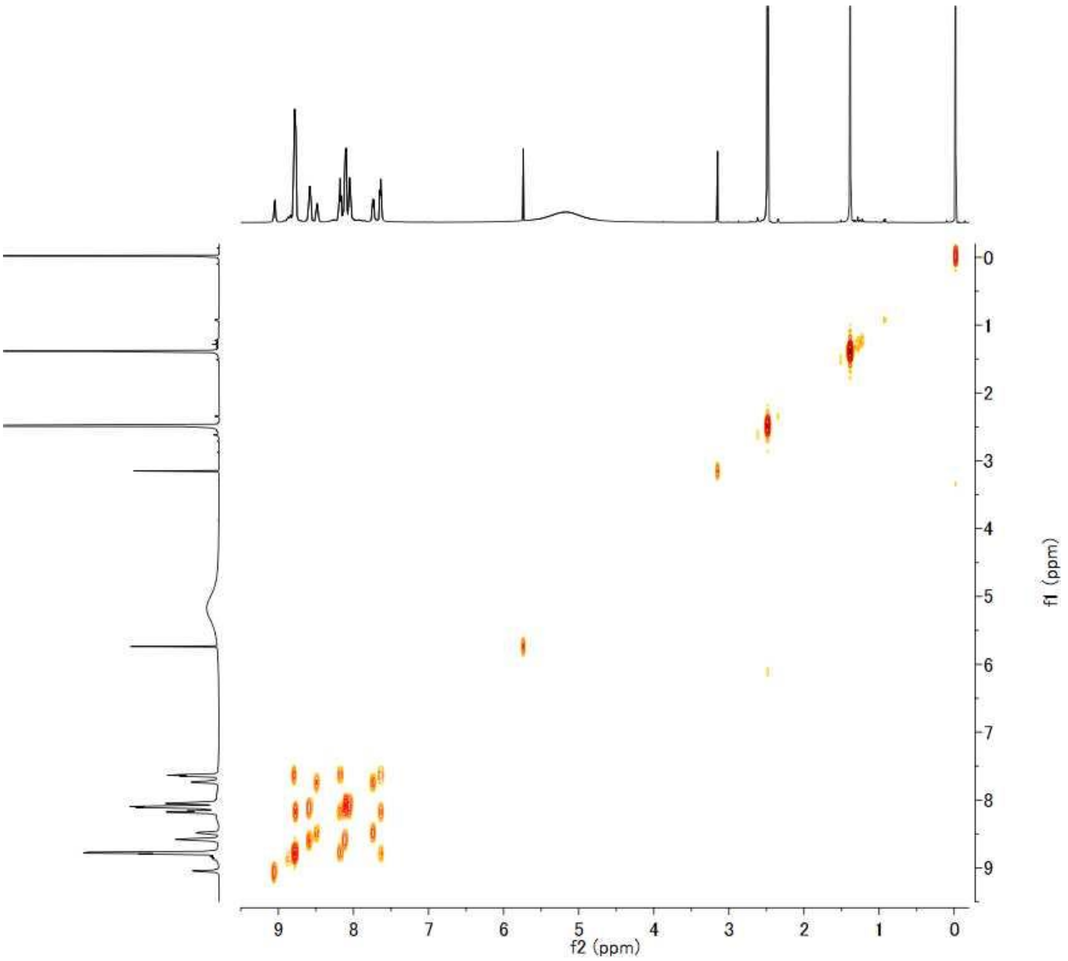

Supplementary Figure 11. 2D COSY NMR (500 MHz, DMSO-d_6, 300 K) spectrum of compound **6.**

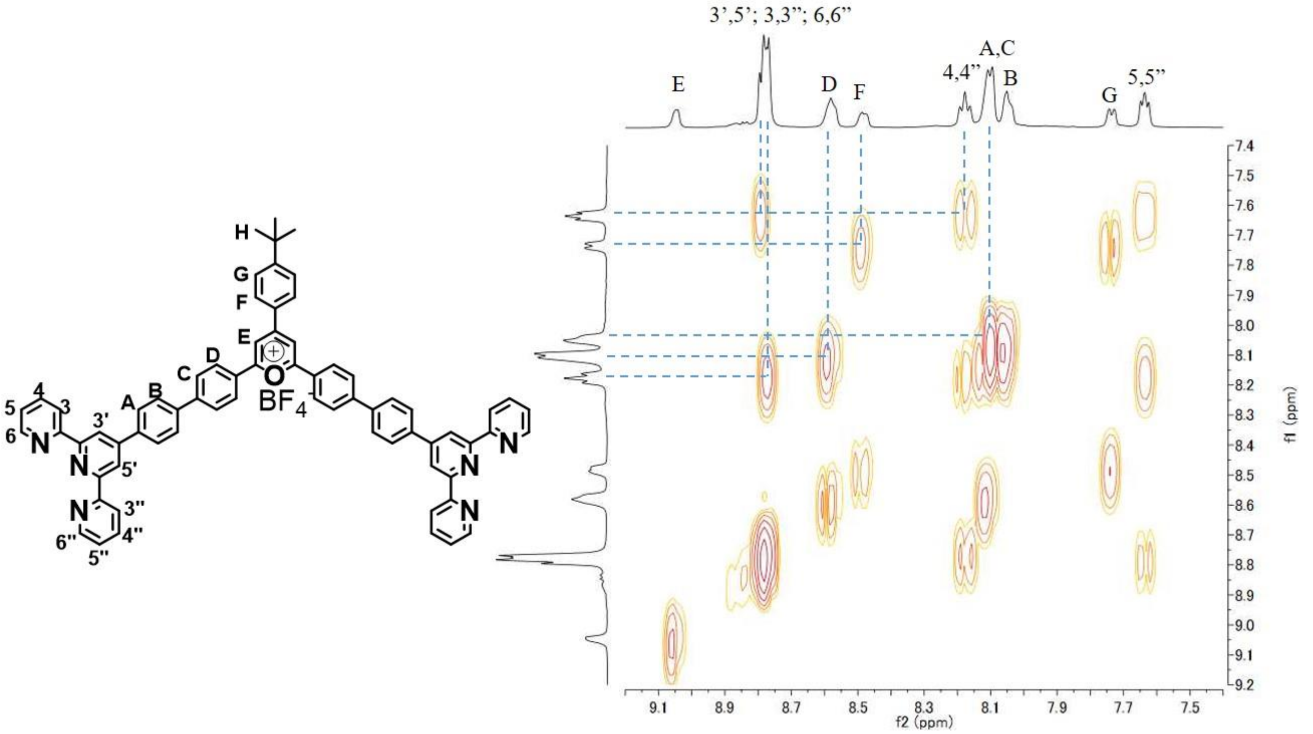

Supplementary Figure 12. 2D COSY NMR (500 MHz, DMSO-d_6, 300 K) spectrum (aromic region) of compound **6.**

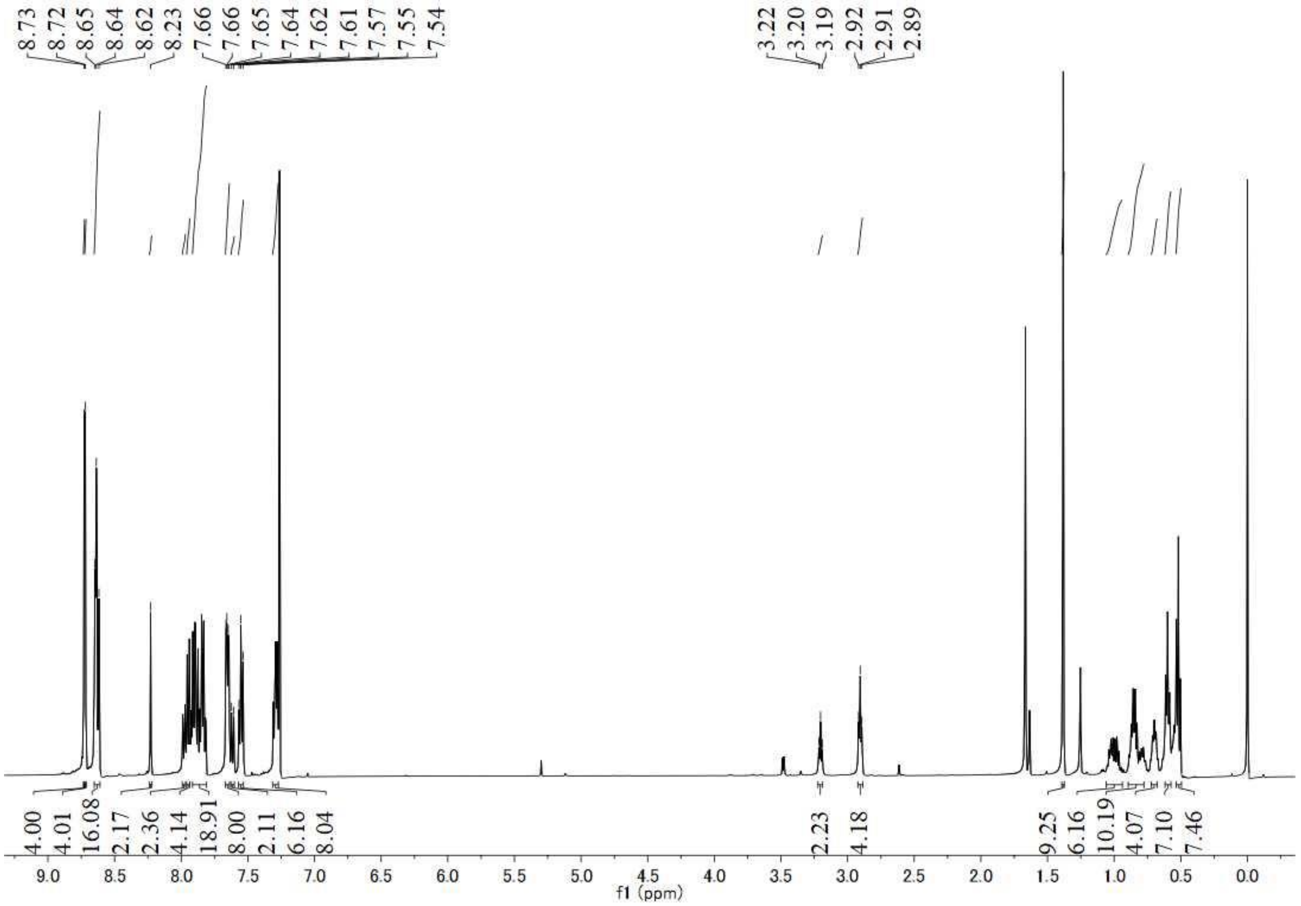

Supplementary Figure 13. ^{1}H NMR (500 MHz, $CDCl_3$, 300 K) spectrum of ligand **L2.**

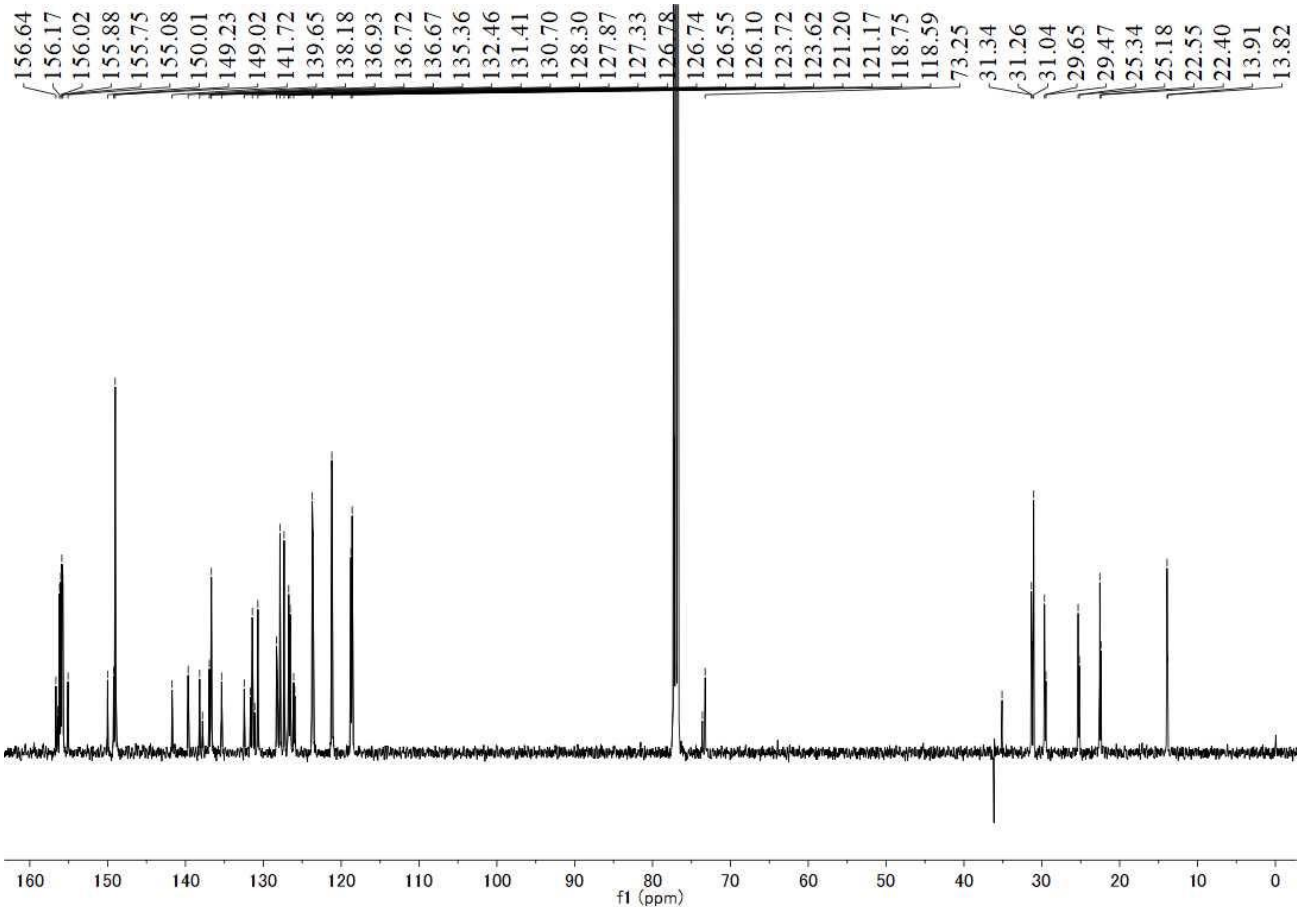

Supplementary Figure 14. ^{13}C NMR (125 MHz, $CDCl_3$, 300 K) spectrum of ligand **L2.**

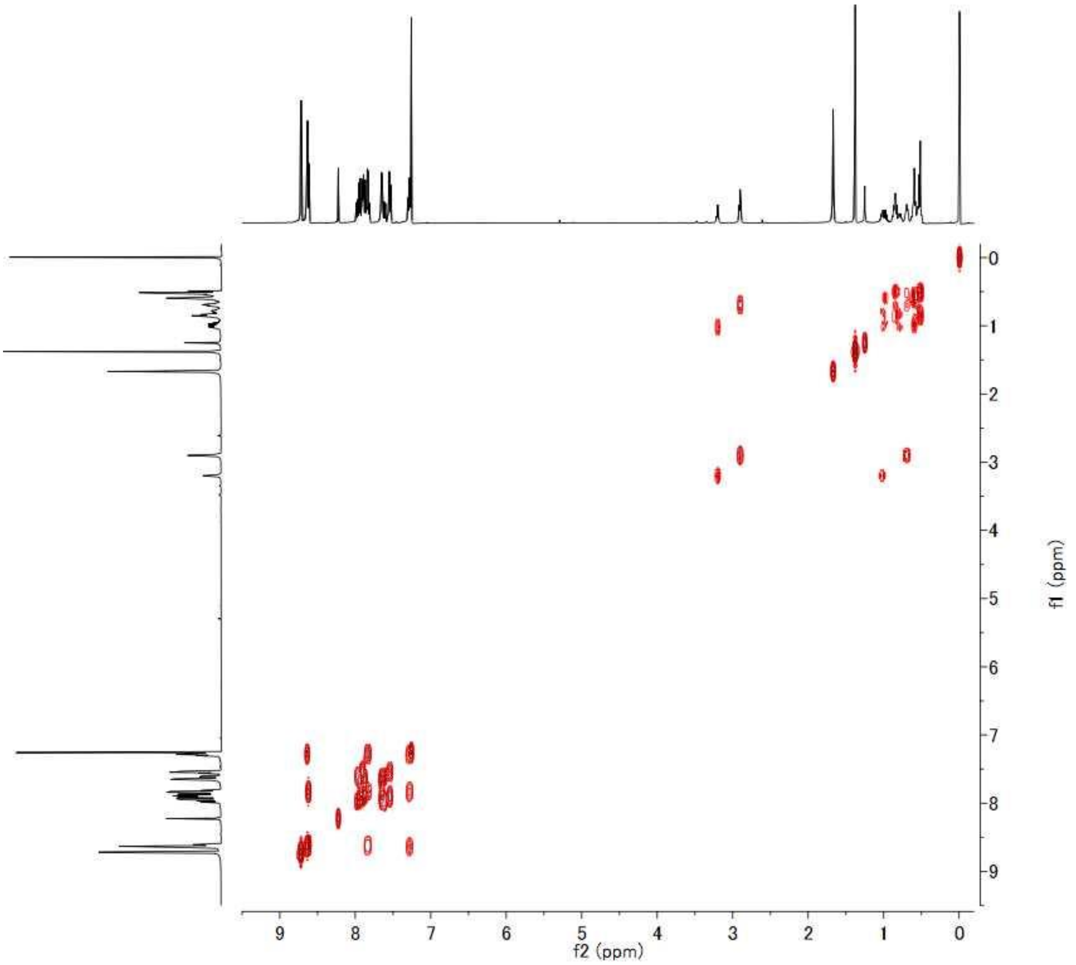

Supplementary Figure 15. 2D COSY NMR (500 MHz, $CDCl_3$, 300 K) spectrum of ligand **L2.**

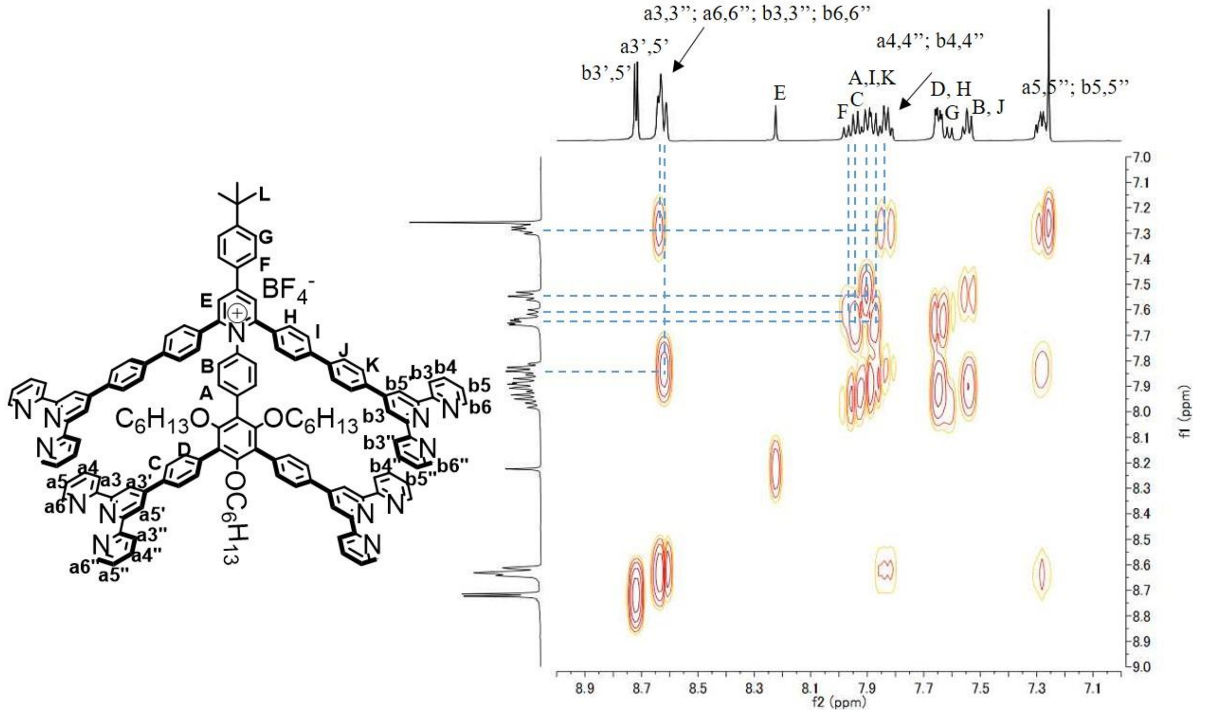

Supplementary Figure 16. 2D COSY NMR (500 MHz, $CDCl_3$, 300 K) spectrum (aromic region) of ligand **L2.**

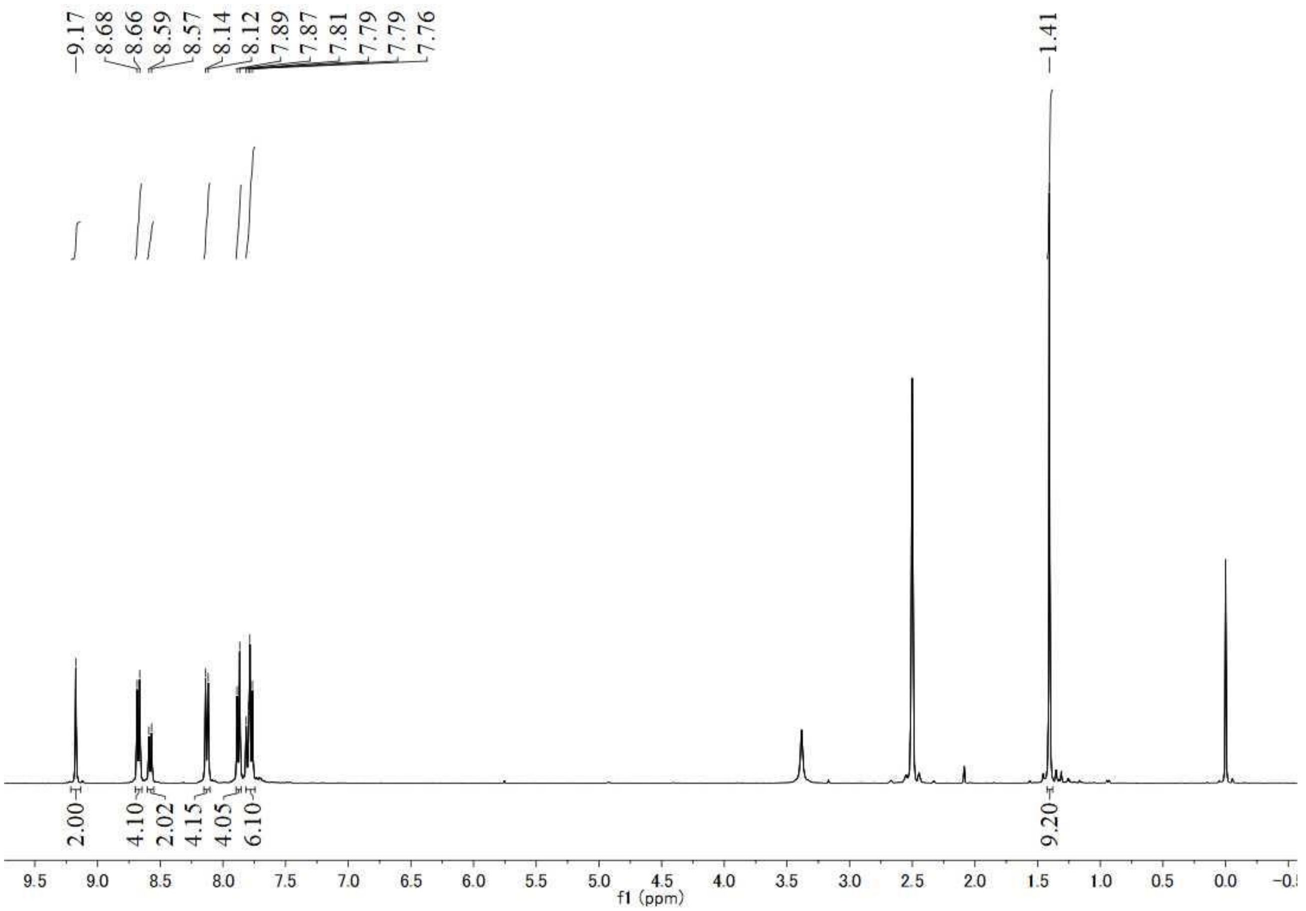

Supplementary Figure 17. ^{1}H NMR (500 MHz, $CDCl_3$, 300 K) spectrum of compound **7.**

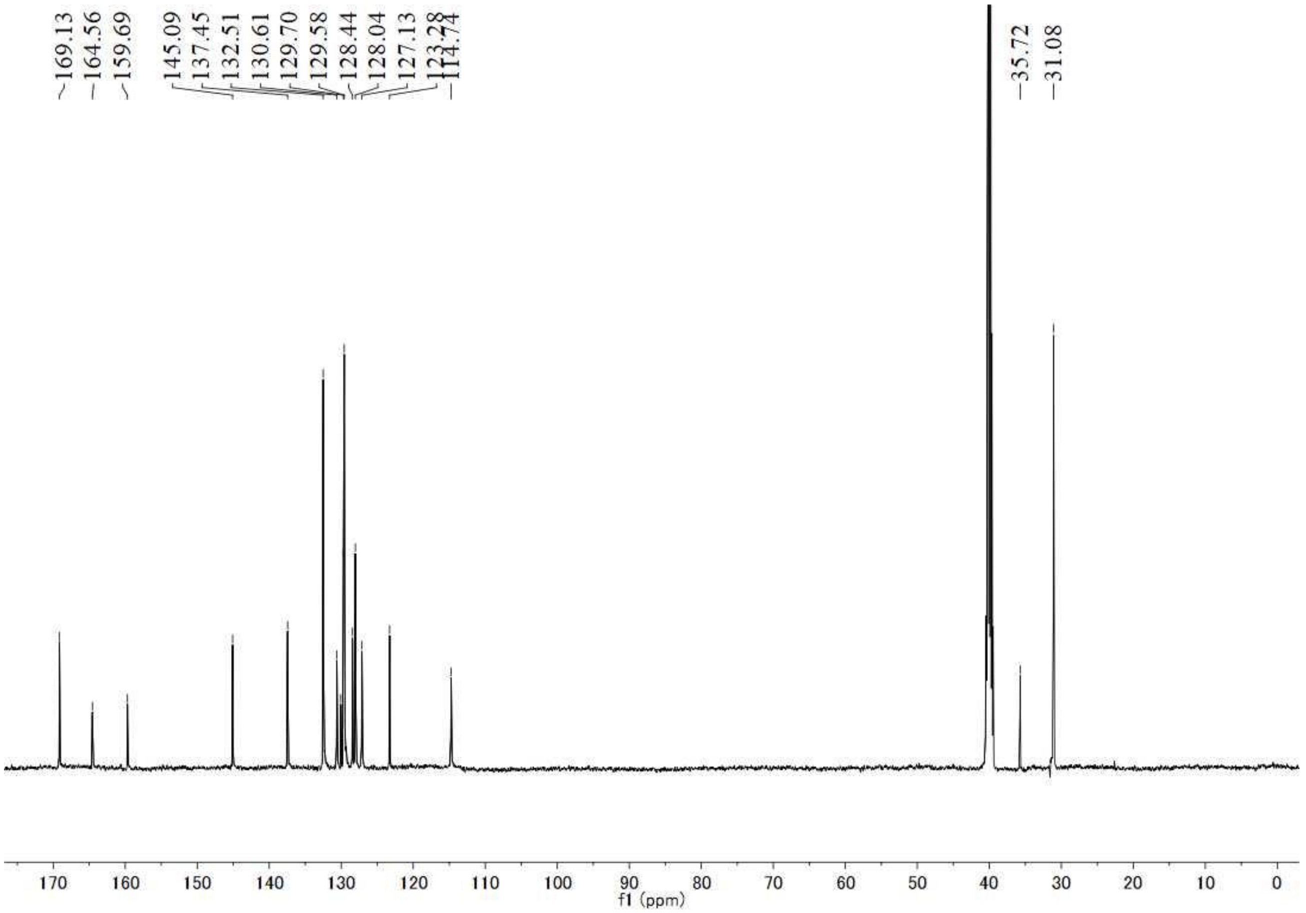

Supplementary Figure 18. ^{13}C NMR (125 MHz, $CDCl_3$, 300 K) spectrum of compound **7.**

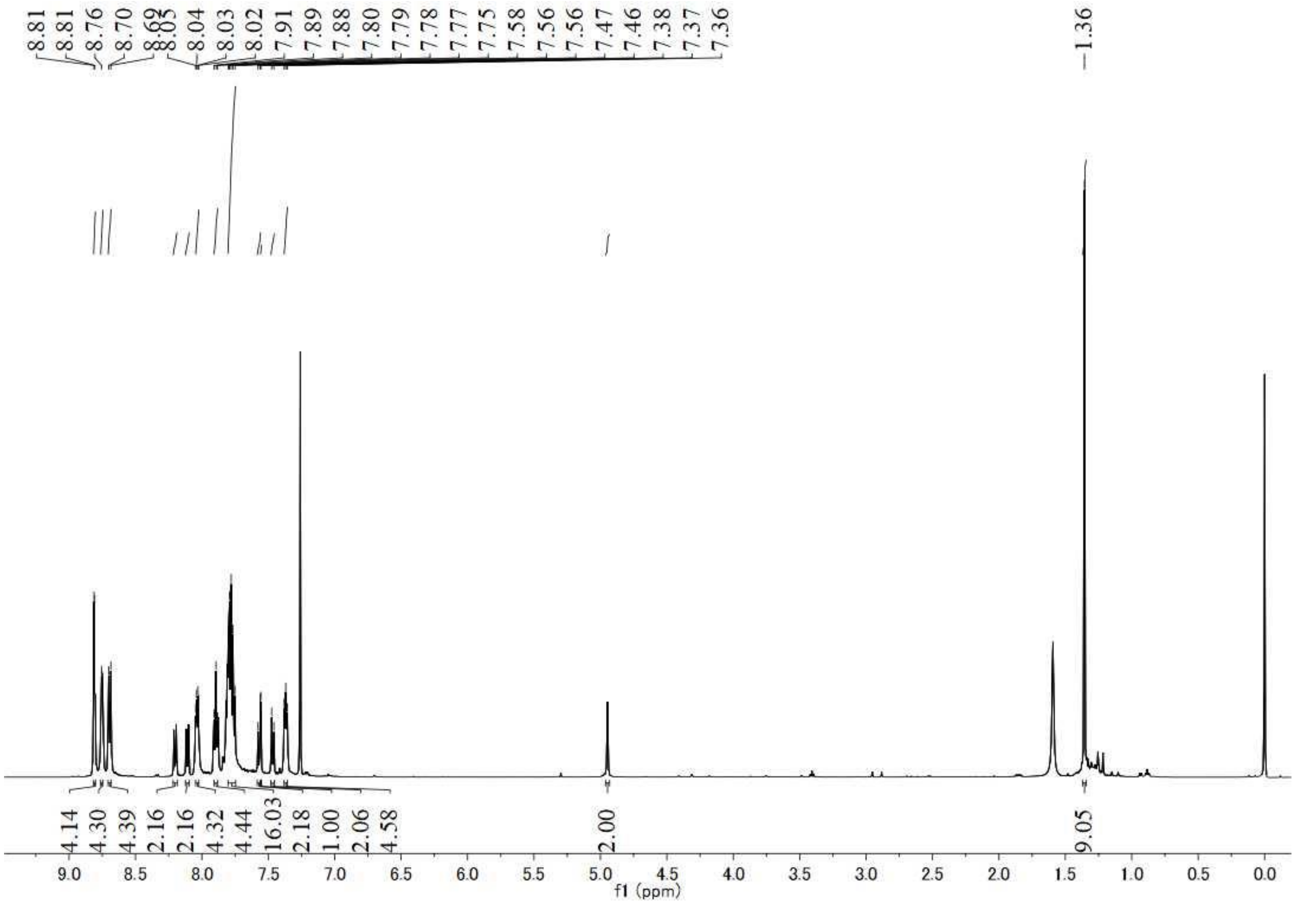

Supplementary Figure 19. ^{1}H NMR (500 MHz, $CDCl_3$, 300 K) spectrum of compound **8.**

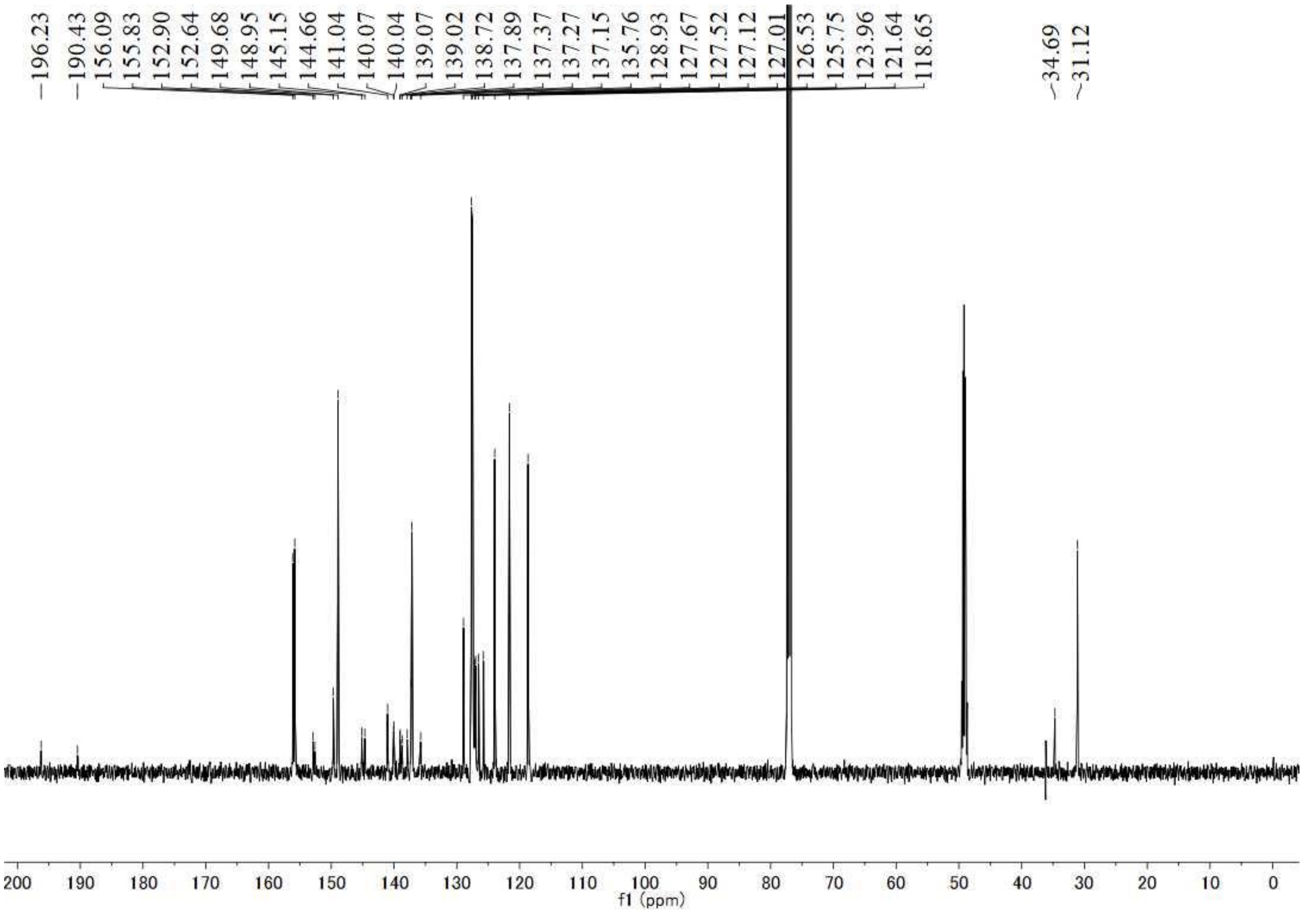

Supplementary Figure 20. ^{13}C NMR (125 MHz, $CDCl_3$, 300 K) spectrum of compound **8.**

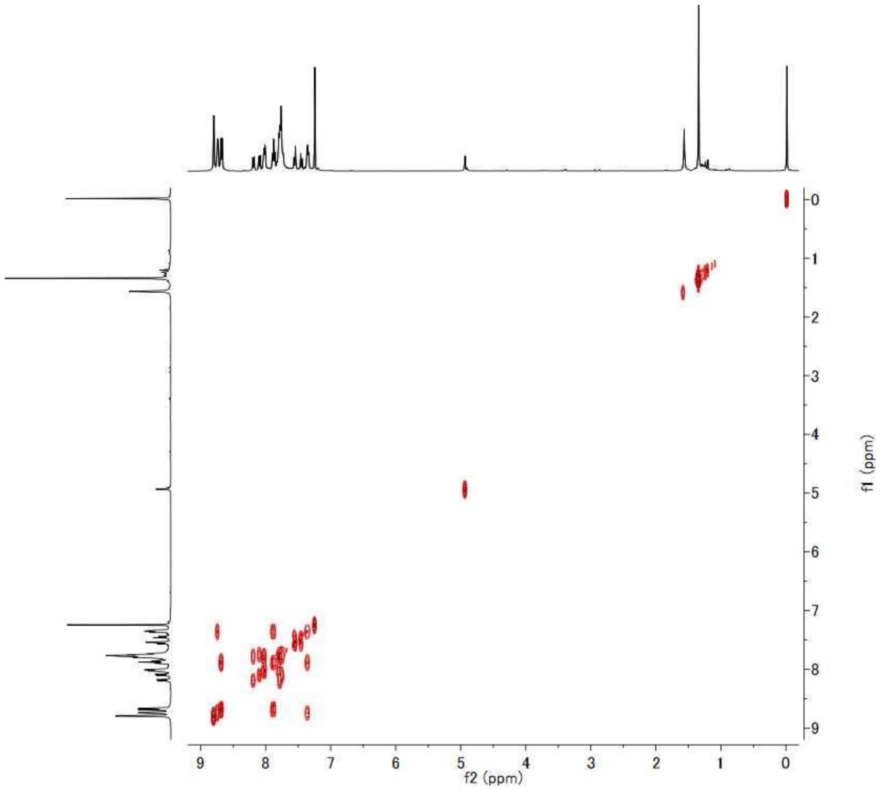

Supplementary Figure 21. 2D COSY NMR (500 MHz, $CDCl_3$, 300 K) spectrum of compound **8.**

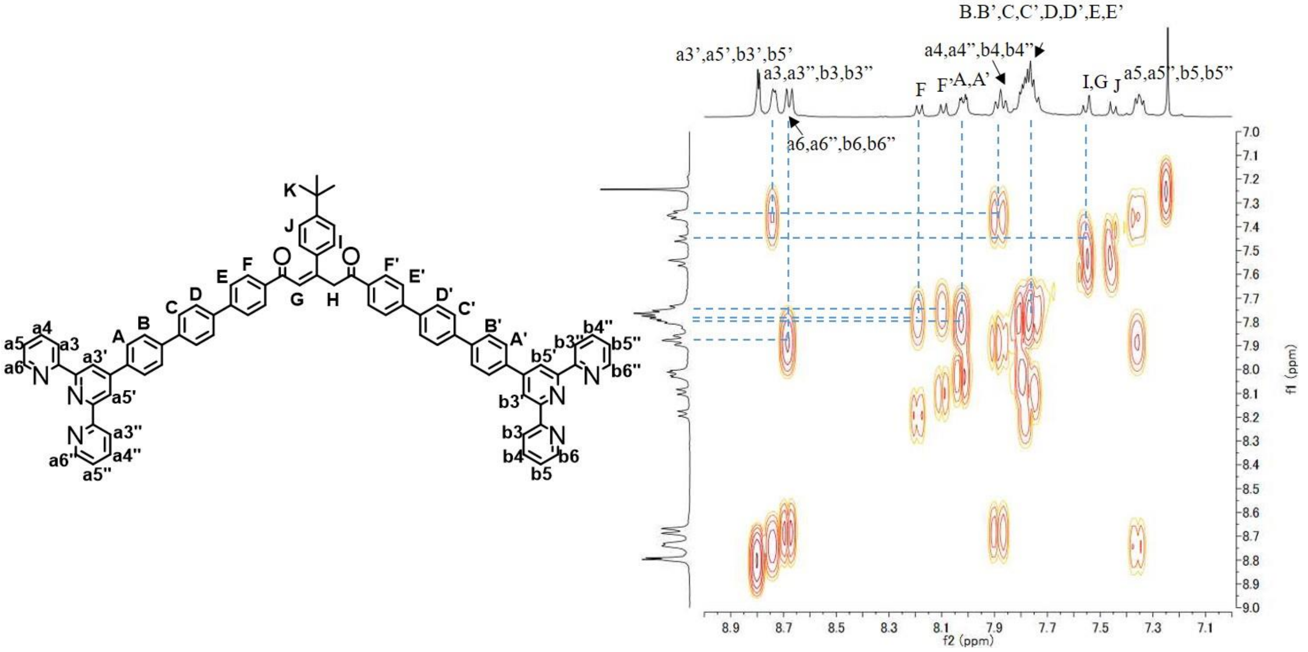

Supplementary Figure 22. 2D COSY NMR (500 MHz, $CDCl_3$, 300 K) spectrum (aromic region) of compound **8.**

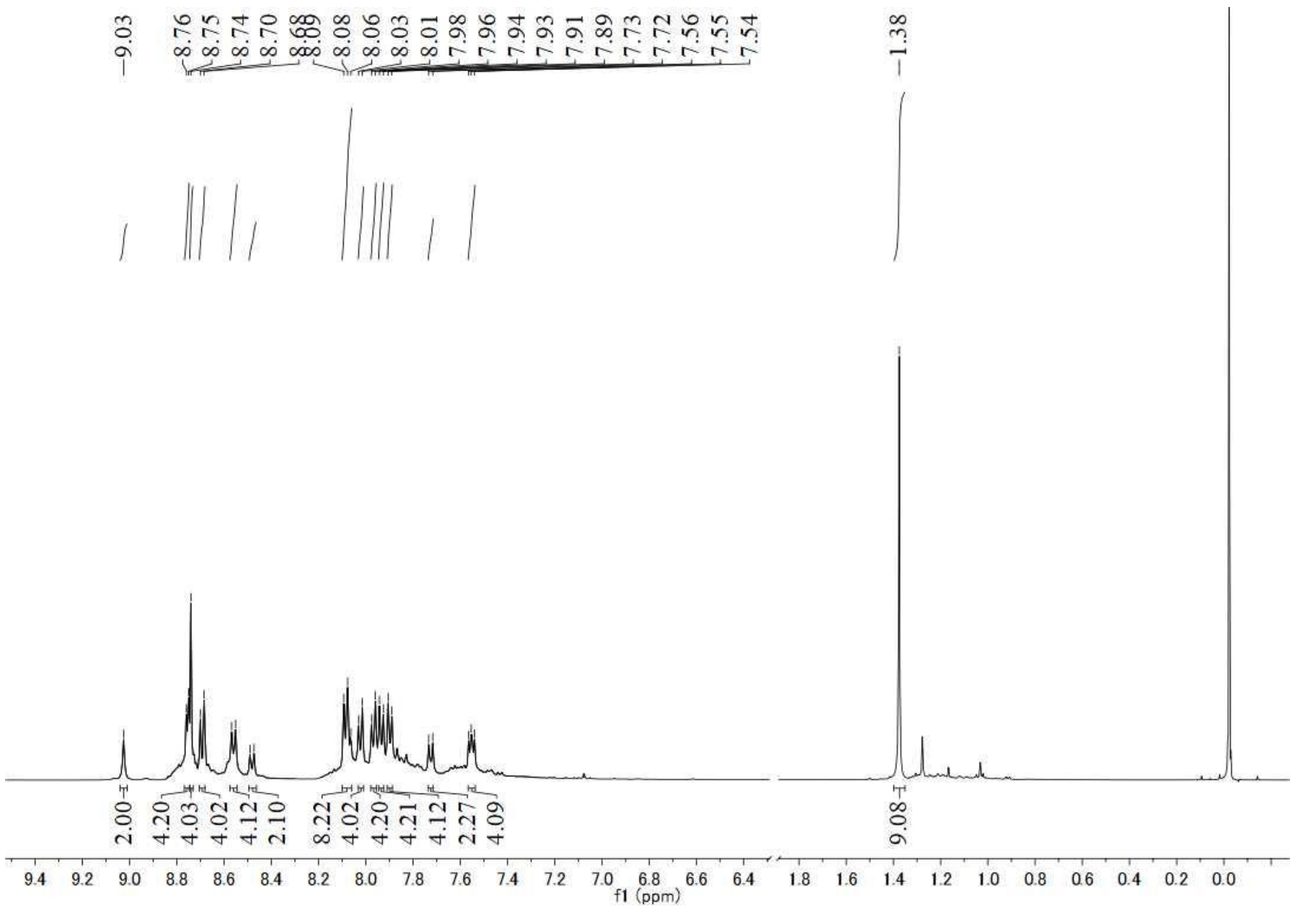

Supplementary Figure 23. ^{1}H NMR (500 MHz, DMSO-d_6, 300 K) spectrum of compound **9.**

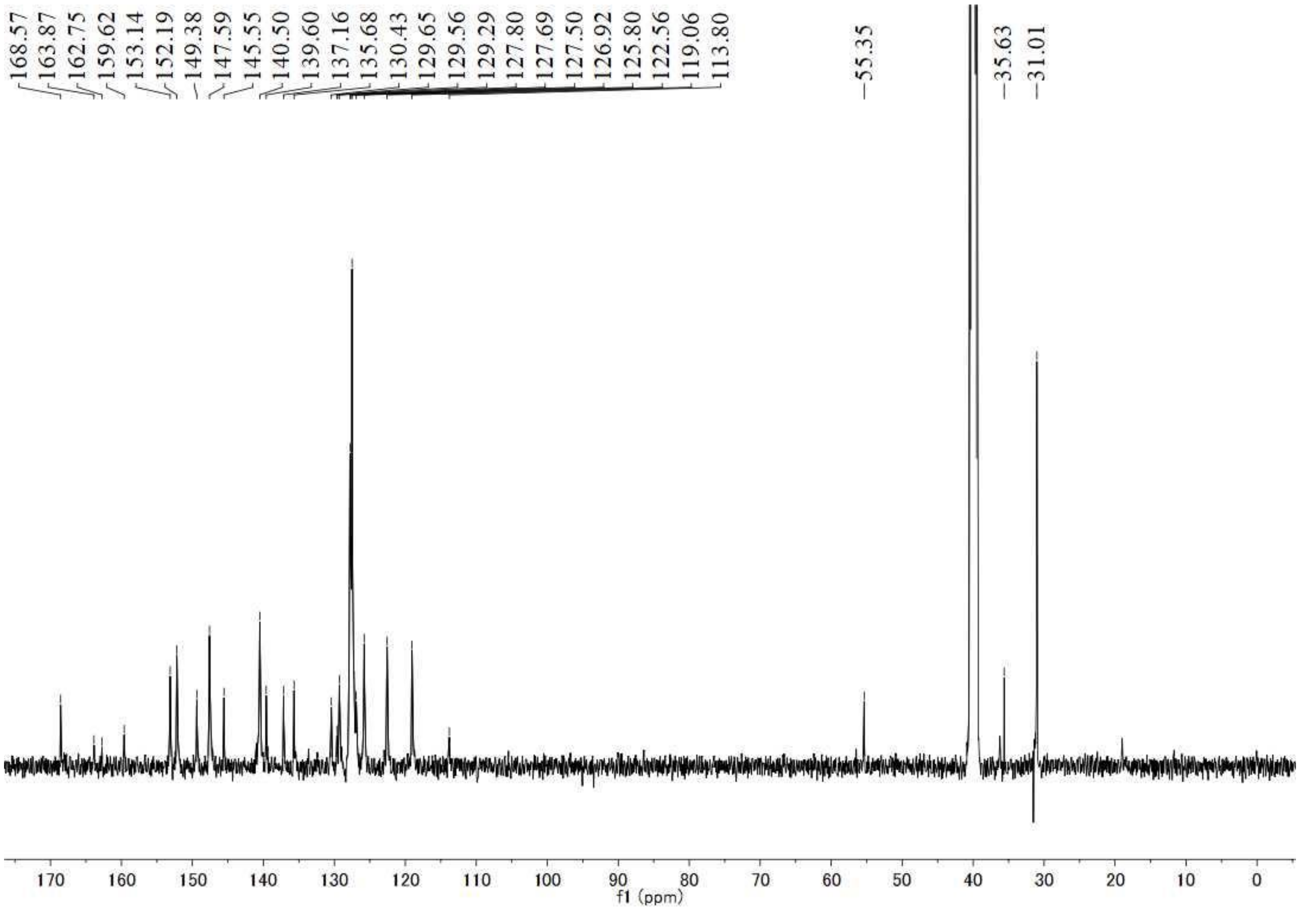

Supplementary Figure 24. ^{13}C NMR (125 MHz, DMSO-d_6, 300 K) spectrum of compound **9.**

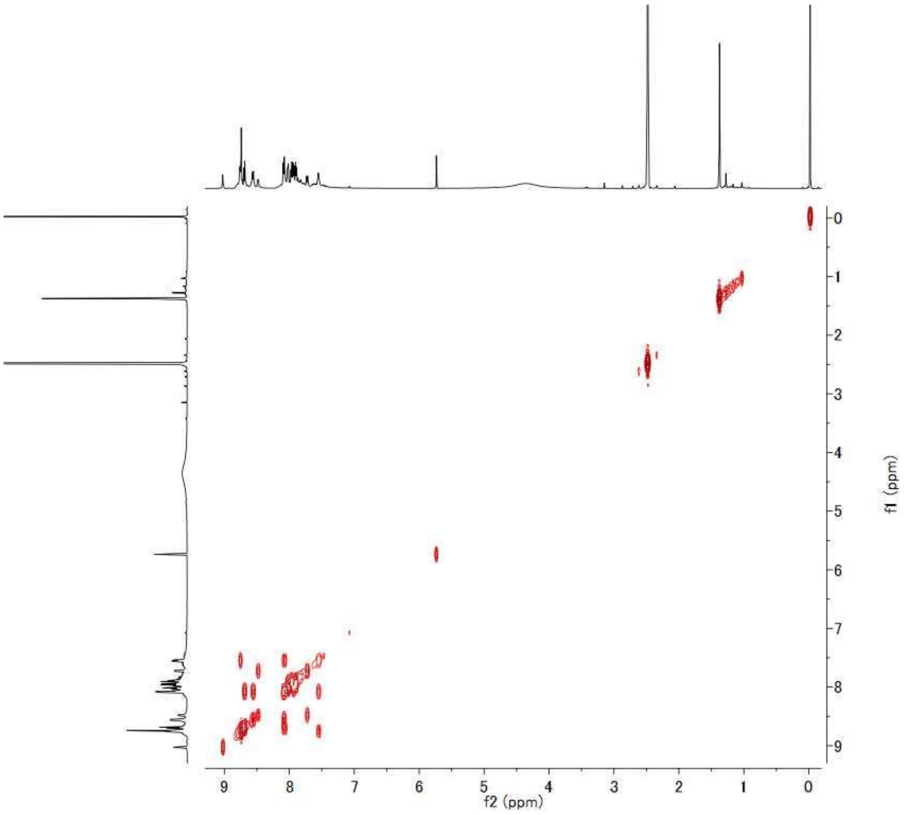

Supplementary Figure 25. 2D COSY NMR (500 MHz, DMSO-d_6, 300 K) spectrum of compound **9.**

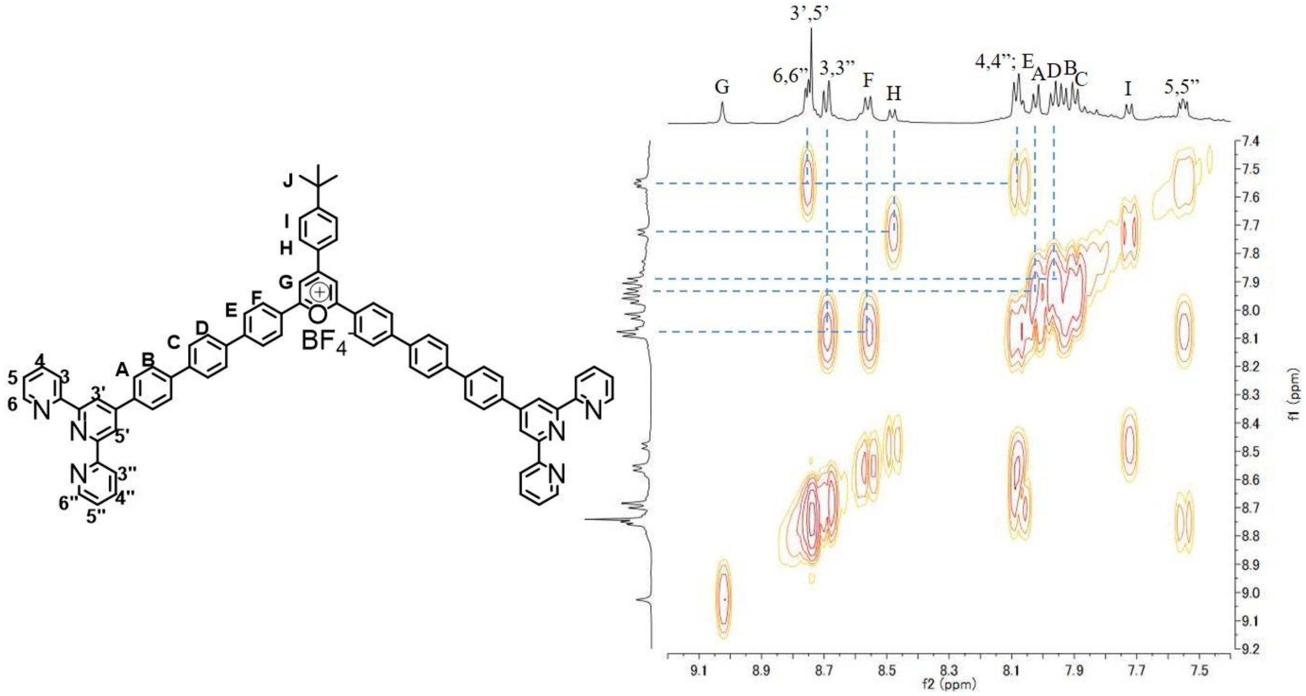

Supplementary Figure 26. 2D COSY NMR (500 MHz, DMSO-d_6, 300 K) spectrum (aromic region) of compound **9.**

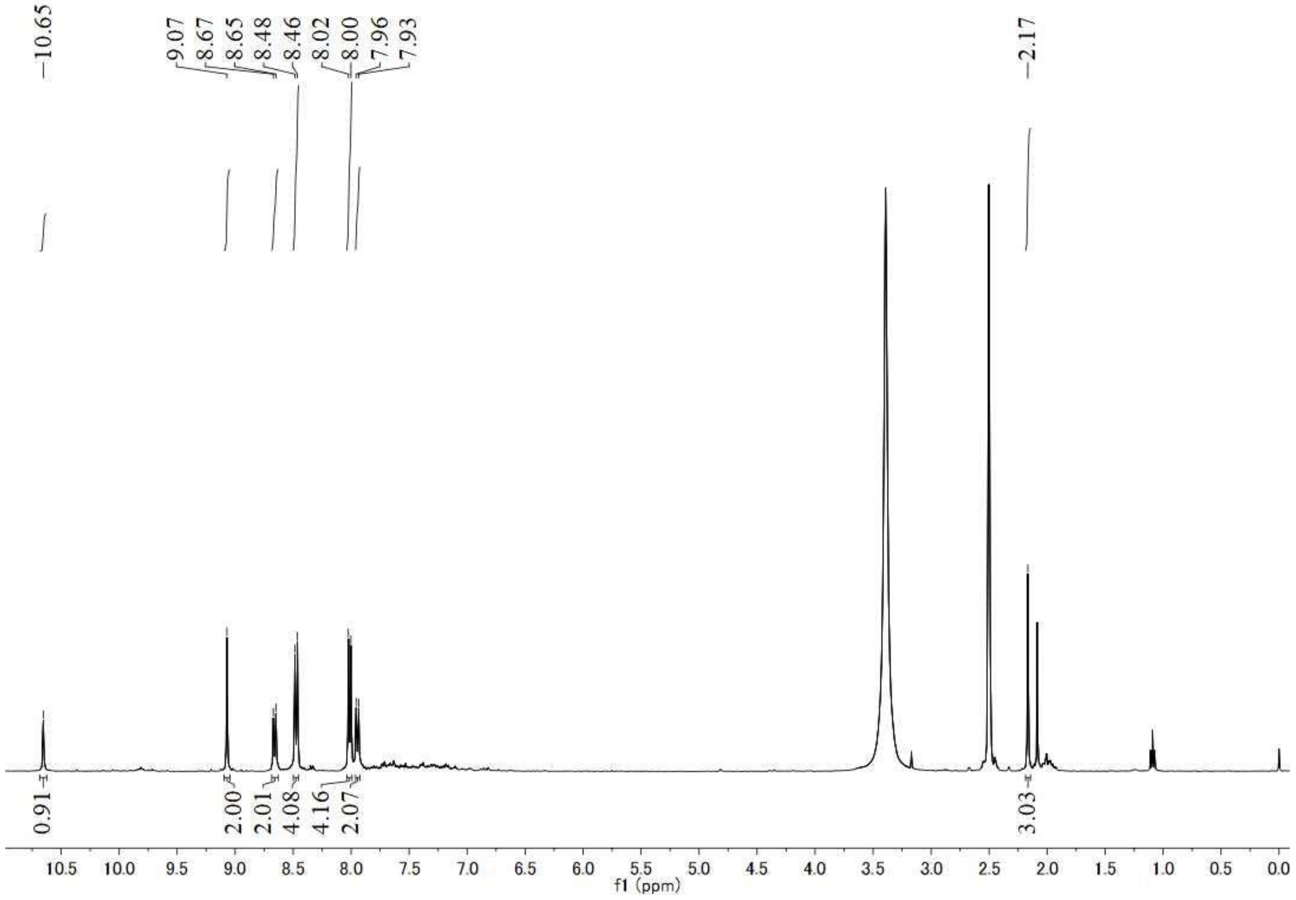

Supplementary Figure 27. ^{1}H NMR (500 MHz, DMSO-d_6, 300 K) spectrum of compound **10.**

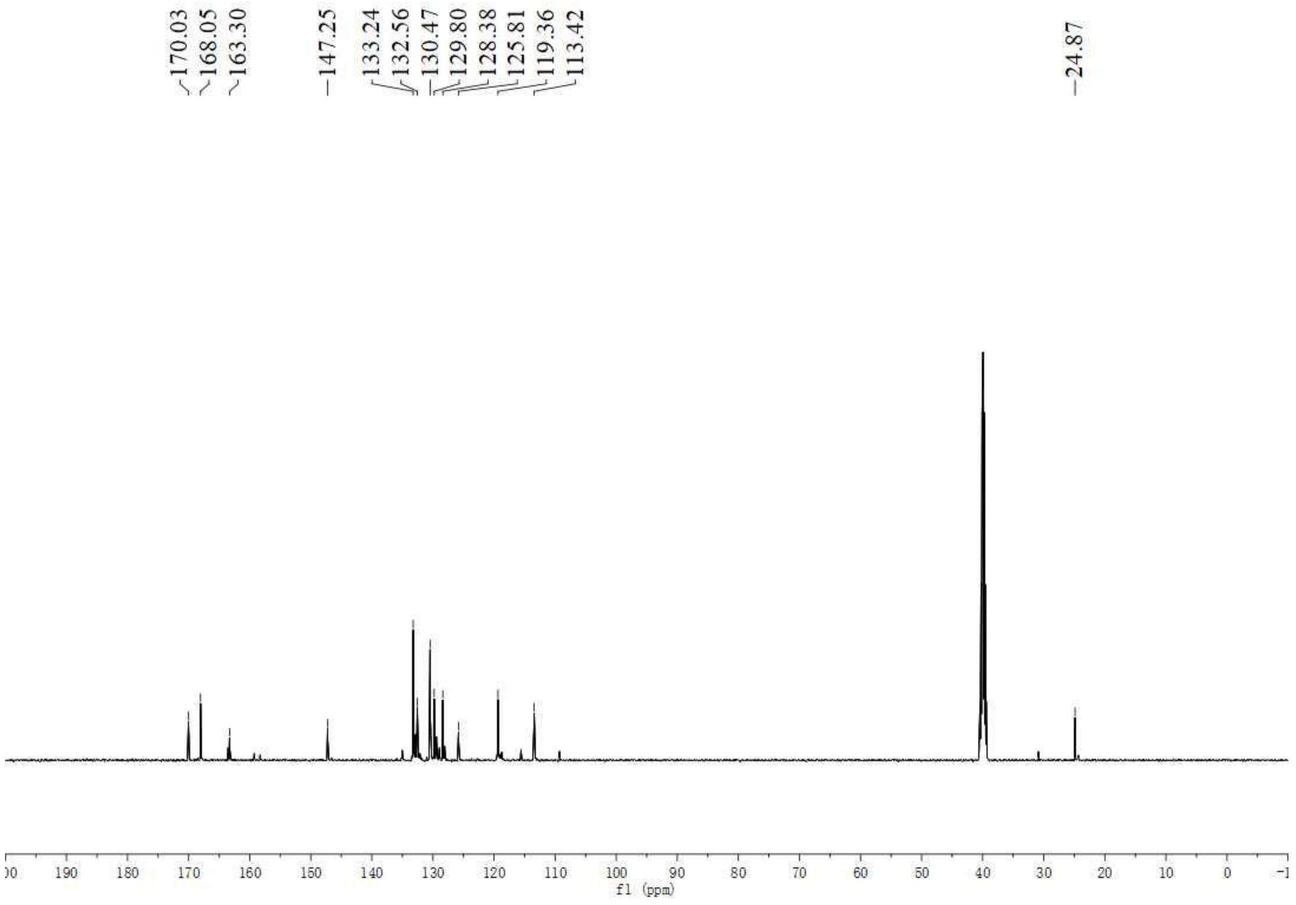

Supplementary Figure 28. ^{13}C NMR (125 MHz, DMSO-d_6, 300 K) spectrum of compound **10.**

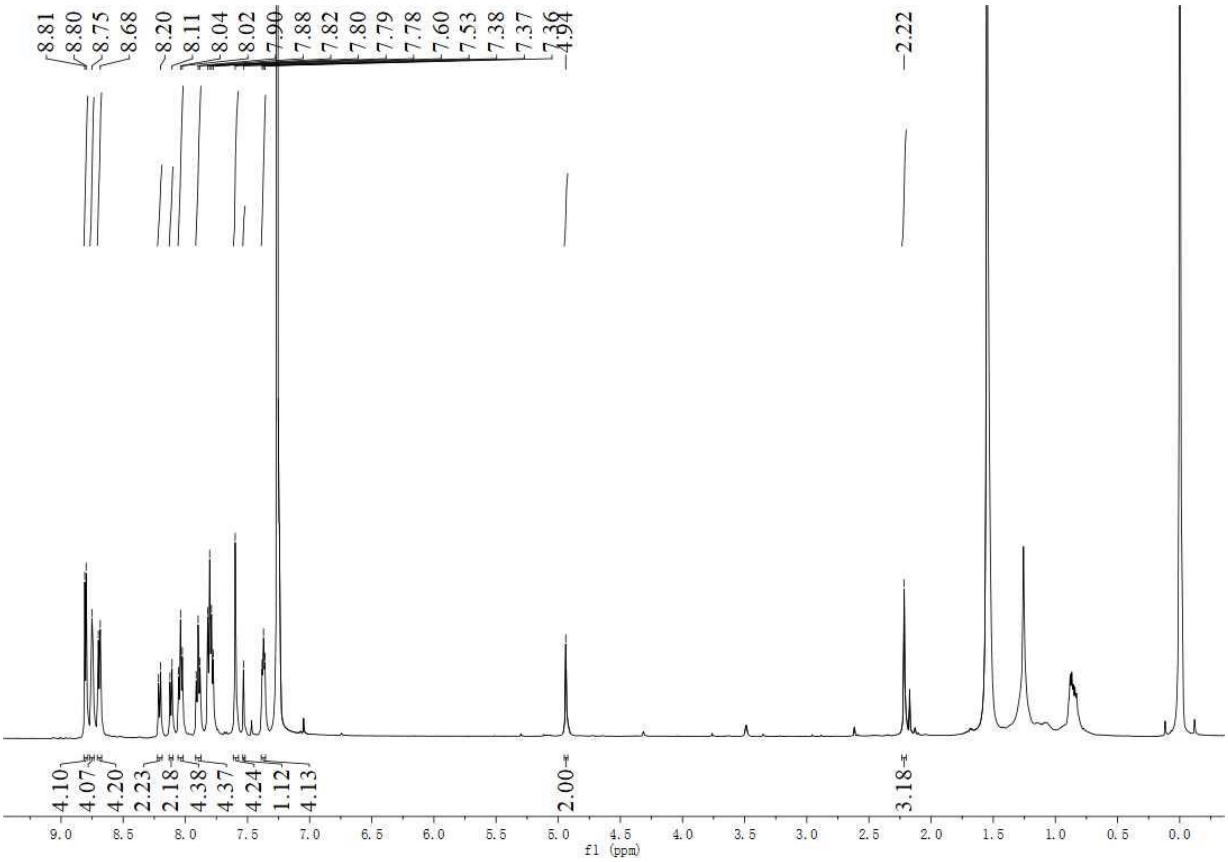

Supplementary Figure 29. ^{1}H NMR (500 MHz, $CDCl_3$, 300 K) spectrum of compound **11.**

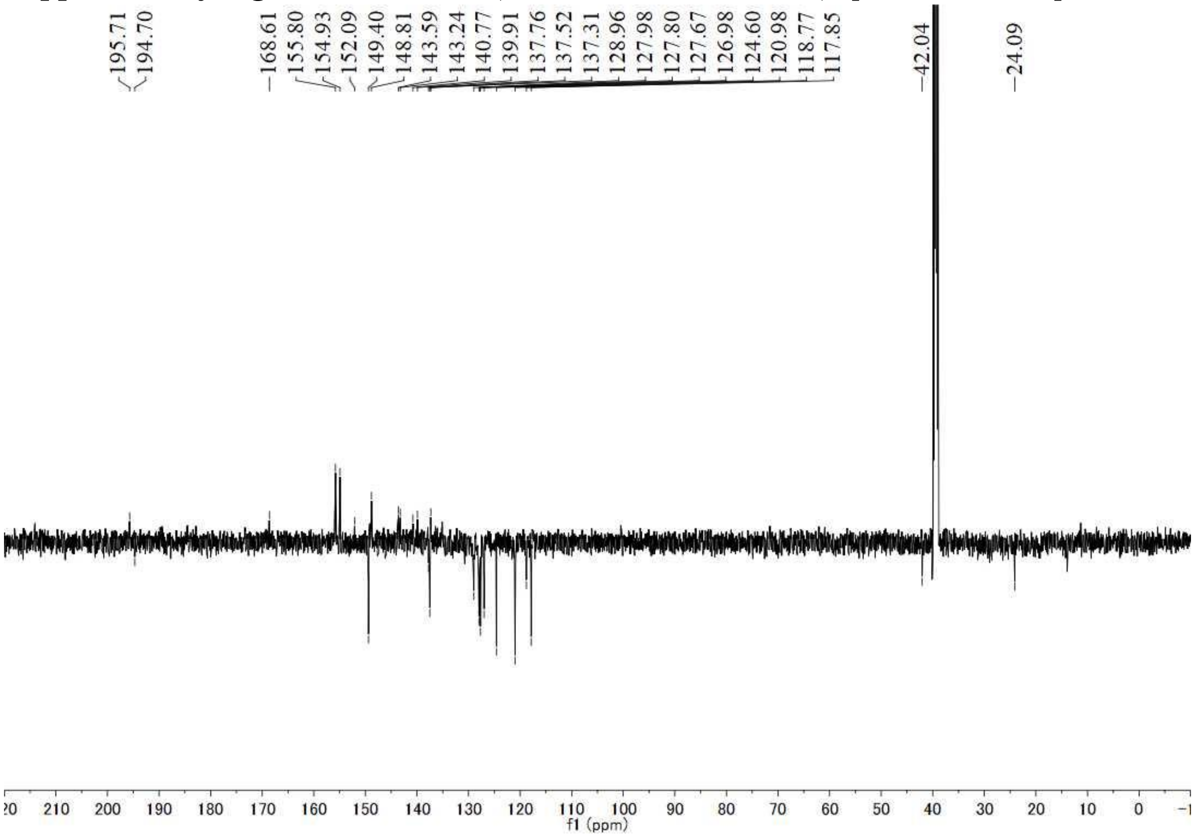

Supplementary Figure 30. ^{13}C DEPTQ NMR (125 MHz, DMSO-d_6, 300 K) spectrum of compound **11.**

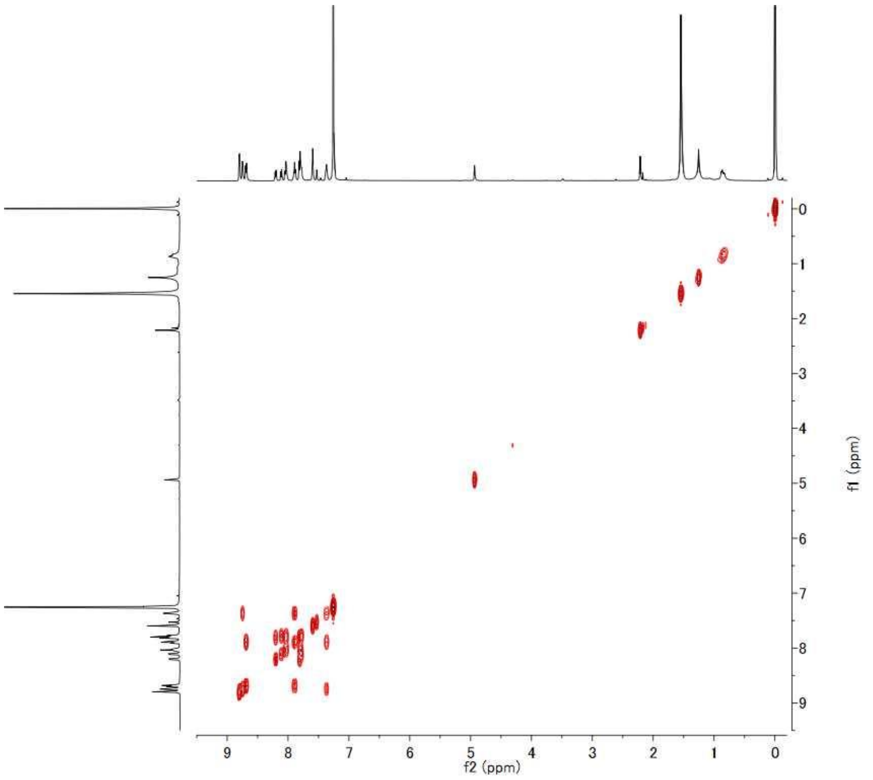

Supplementary Figure 31. 2D COSY NMR (500 MHz, $CDCl_3$, 300 K) spectrum of compound **11.**

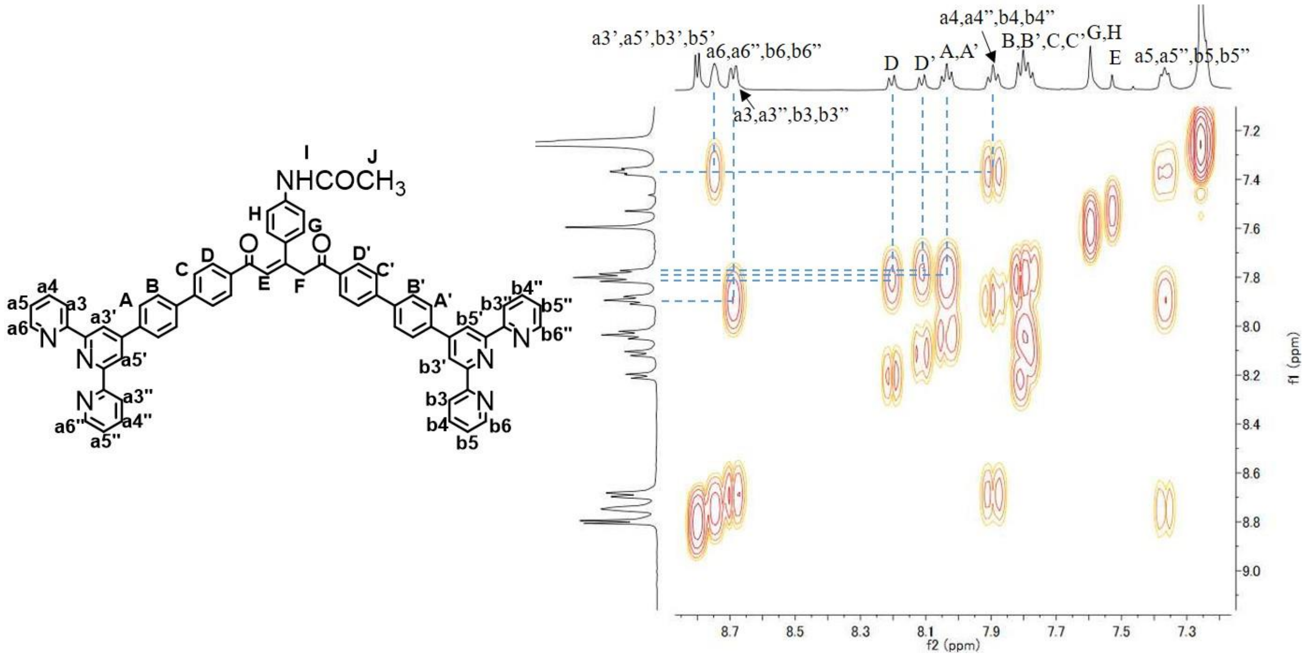

Supplementary Figure 32. 2D COSY NMR (500 MHz, $CDCl_3$, 300 K) spectrum (aromic region) of compound **11.**

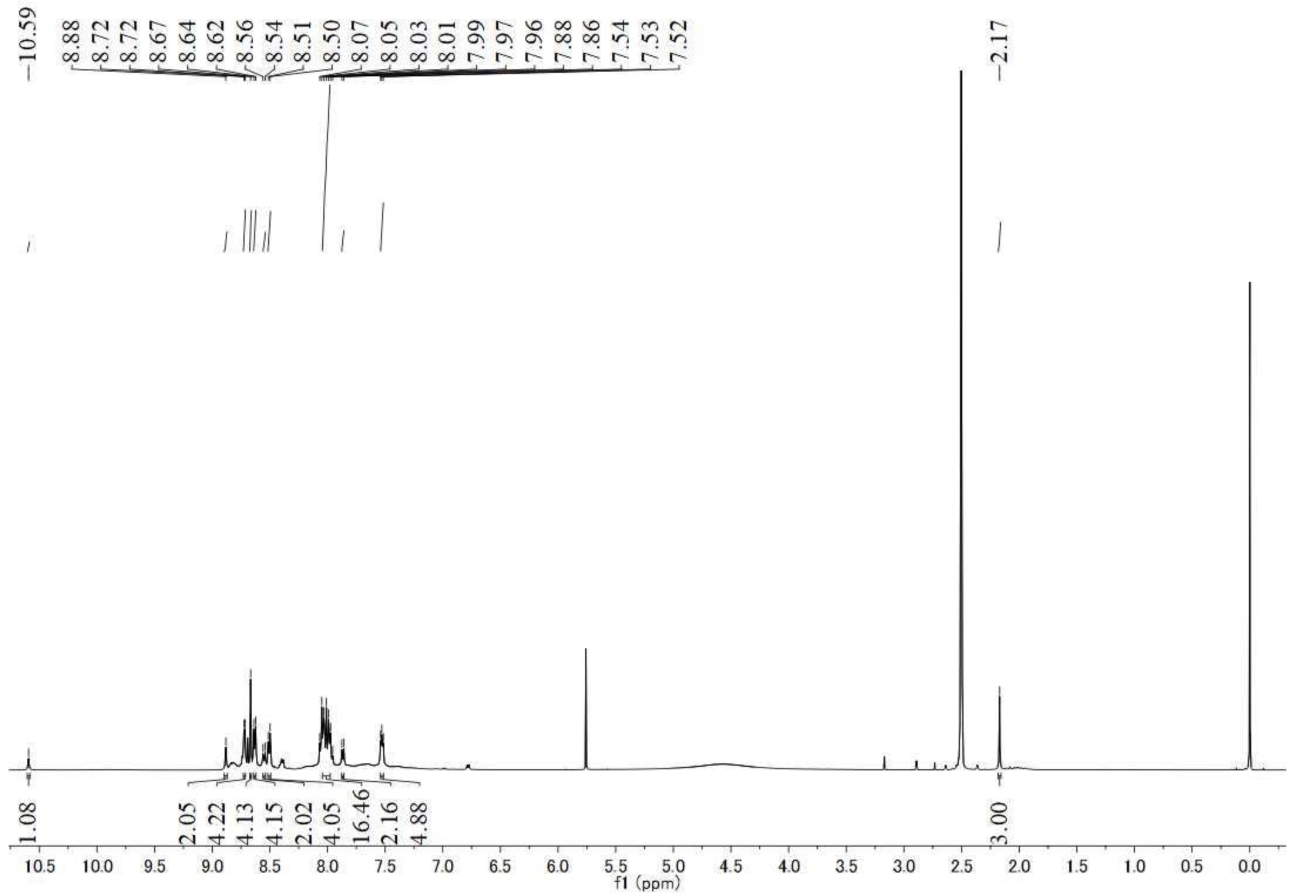

Supplementary Figure 33. ^{1}H NMR (500 MHz, DMSO-d_6, 300 K) spectrum of compound **12.**

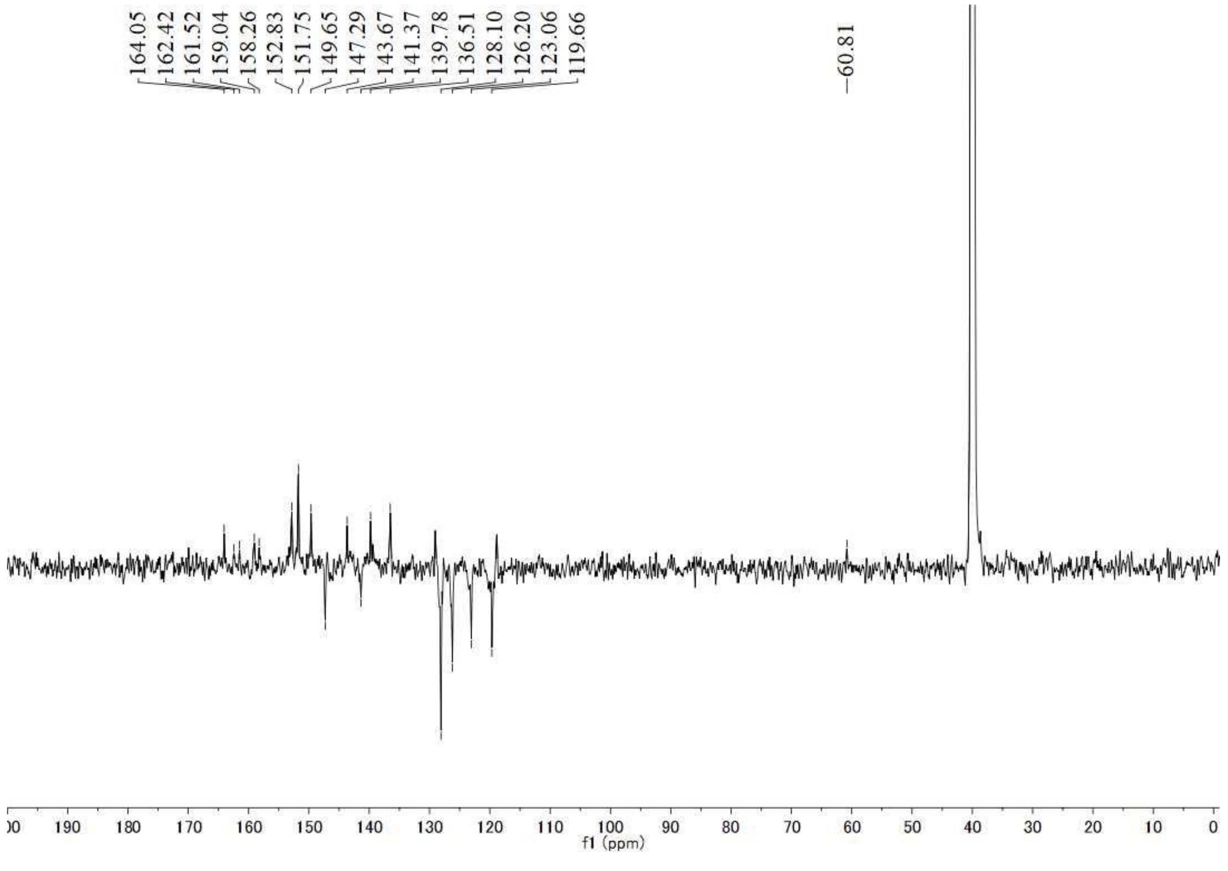

Supplementary Figure 34. ^{13}C DEPTQ NMR (150 MHz, DMSO-d_6, 300 K) spectrum of compound **12.**

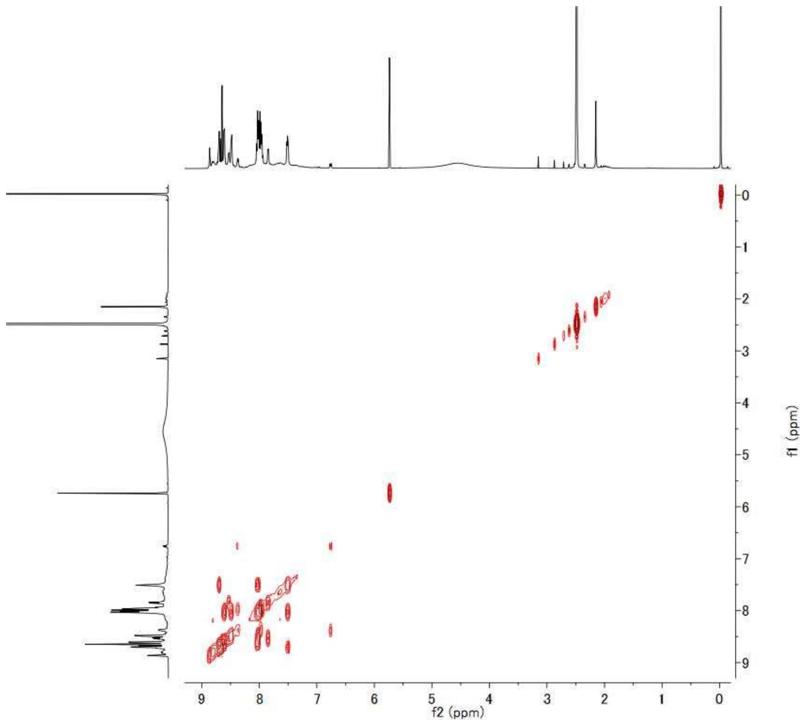

Supplementary Figure 35. 2D COSY NMR (500 MHz, DMSO-d_6, 300 K) spectrum of compound **12.**

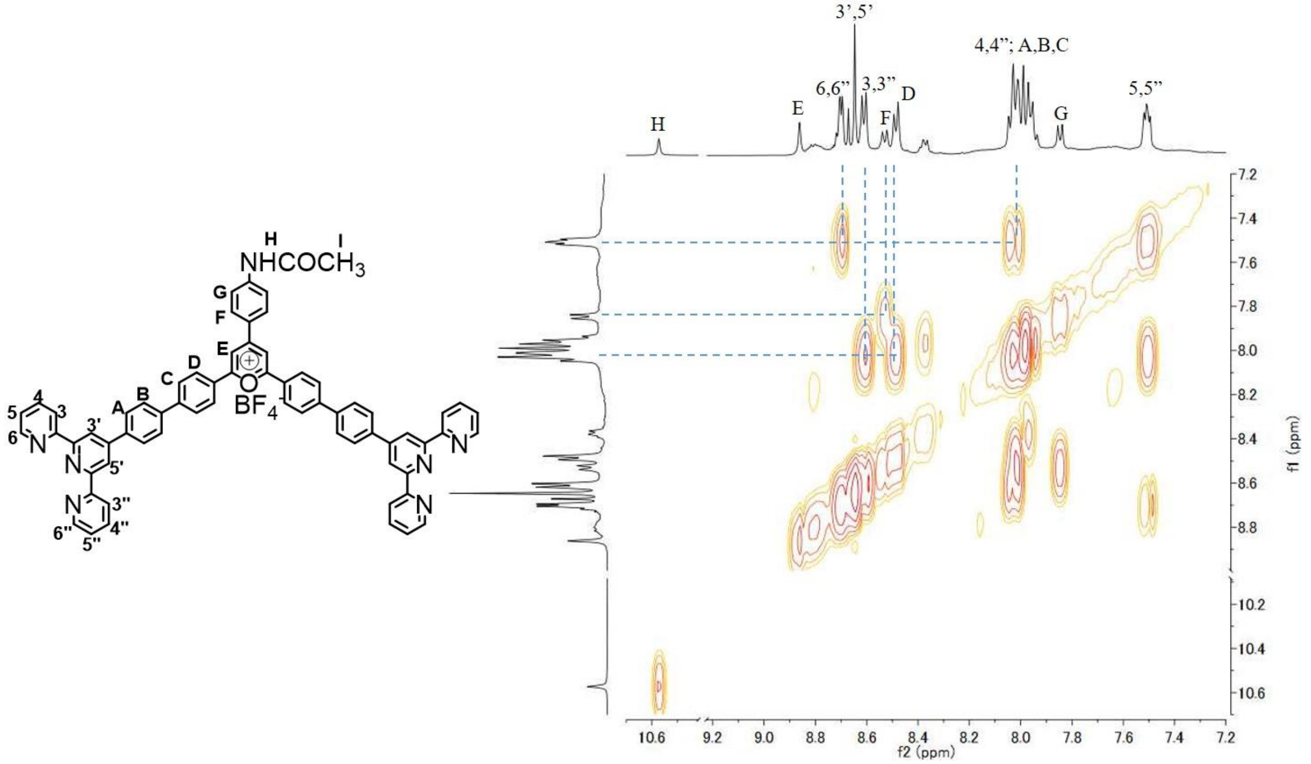

Supplementary Figure 36. 2D COSY NMR (500 MHz, DMSO-d_6, 300 K) spectrum (aromic region) of compound **12.**

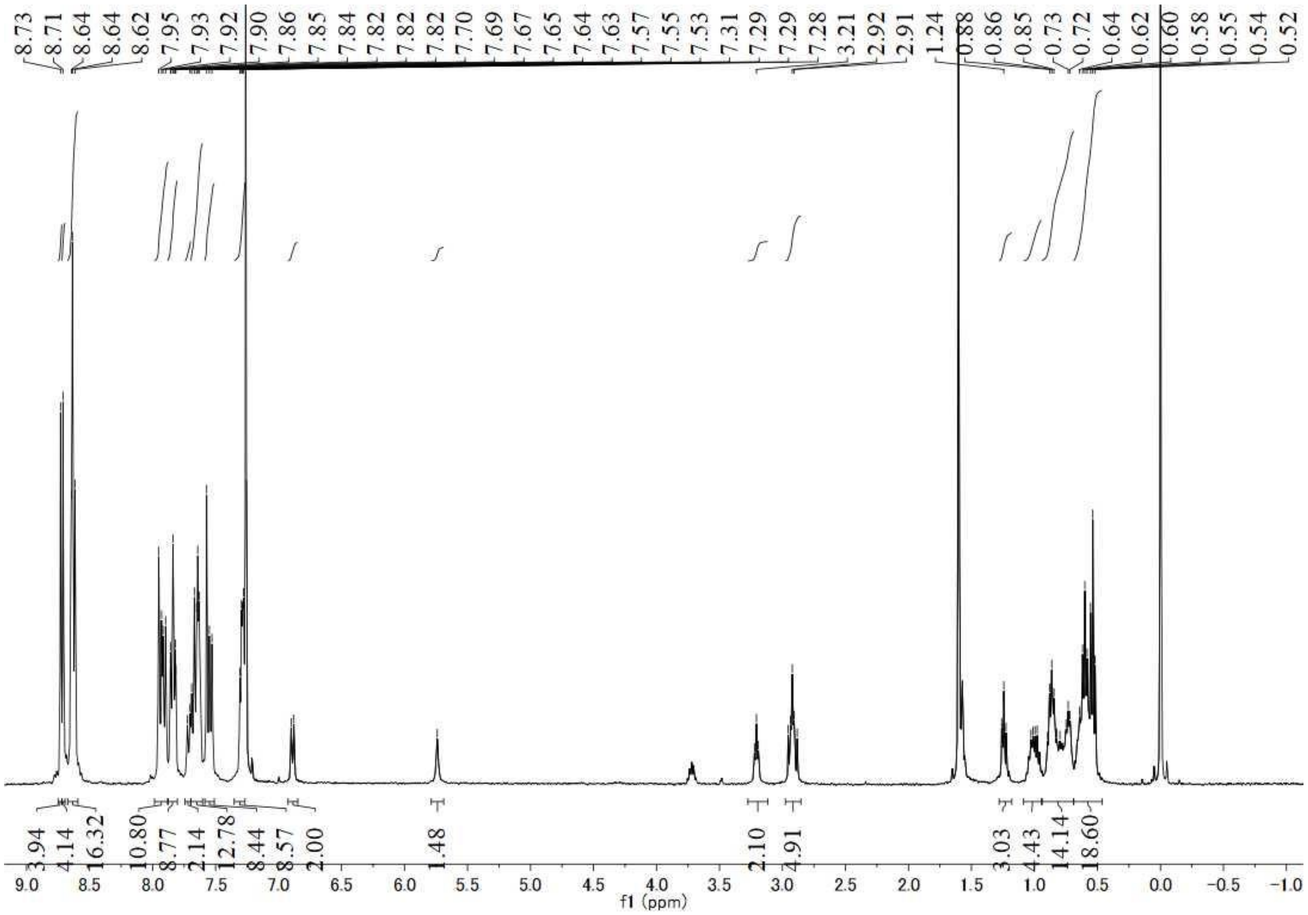

Supplementary Figure 37. ^{1}H NMR (400 MHz, $CDCl_3$, 300 K) spectrum of compound **13.**

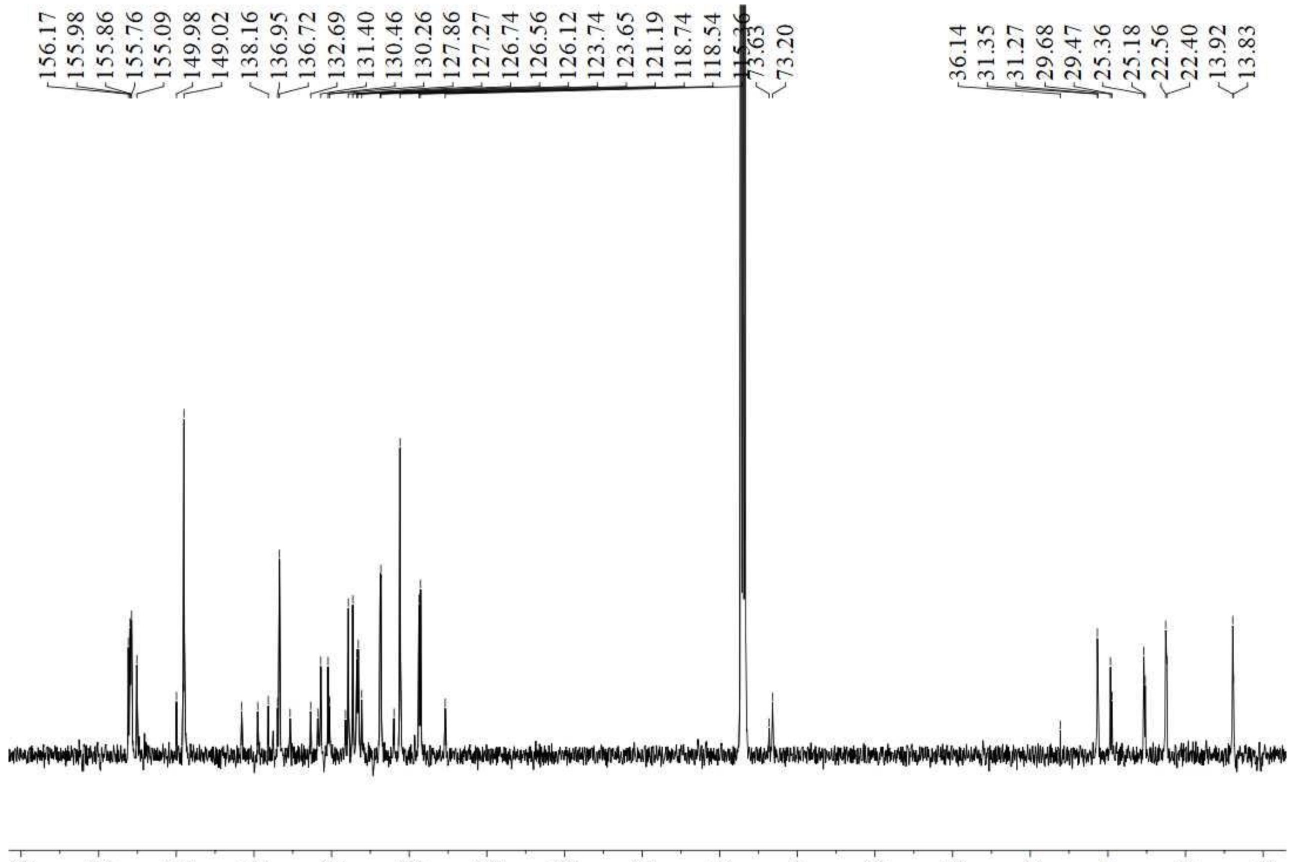

Supplementary Figure 38. ^{13}C NMR (125 MHz, $CDCl_3$, 300 K) spectrum of compound **13.**

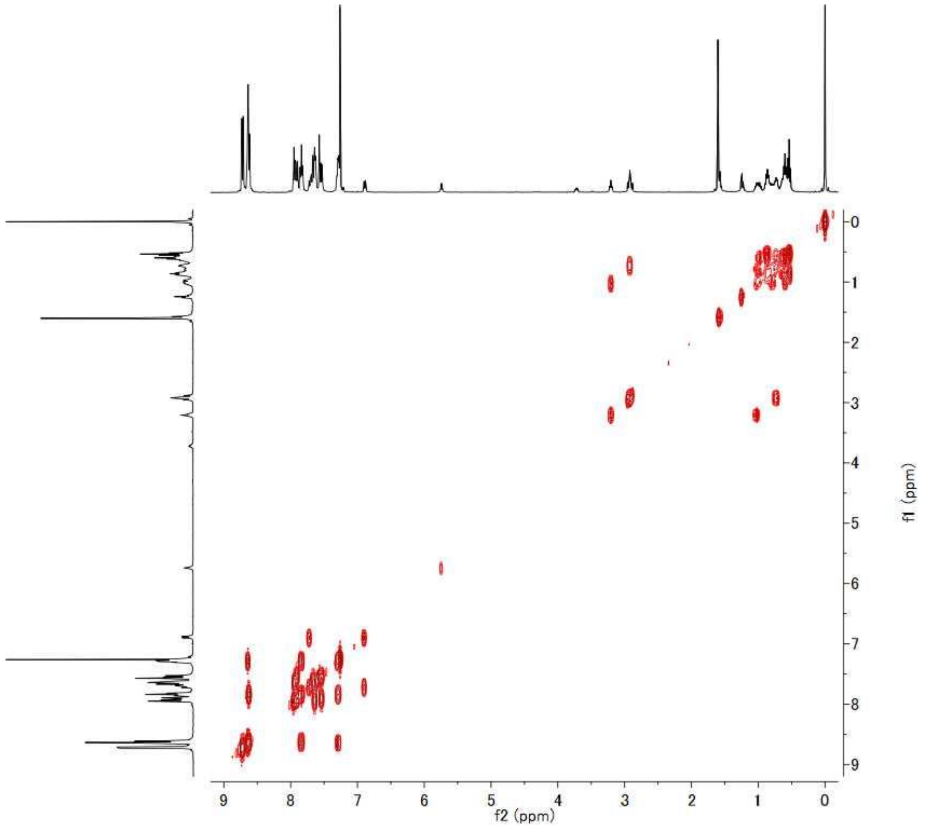

Supplementary Figure 39. 2D COSY NMR (400 MHz, $CDCl_3$, 300 K) spectrum of compound **13.**

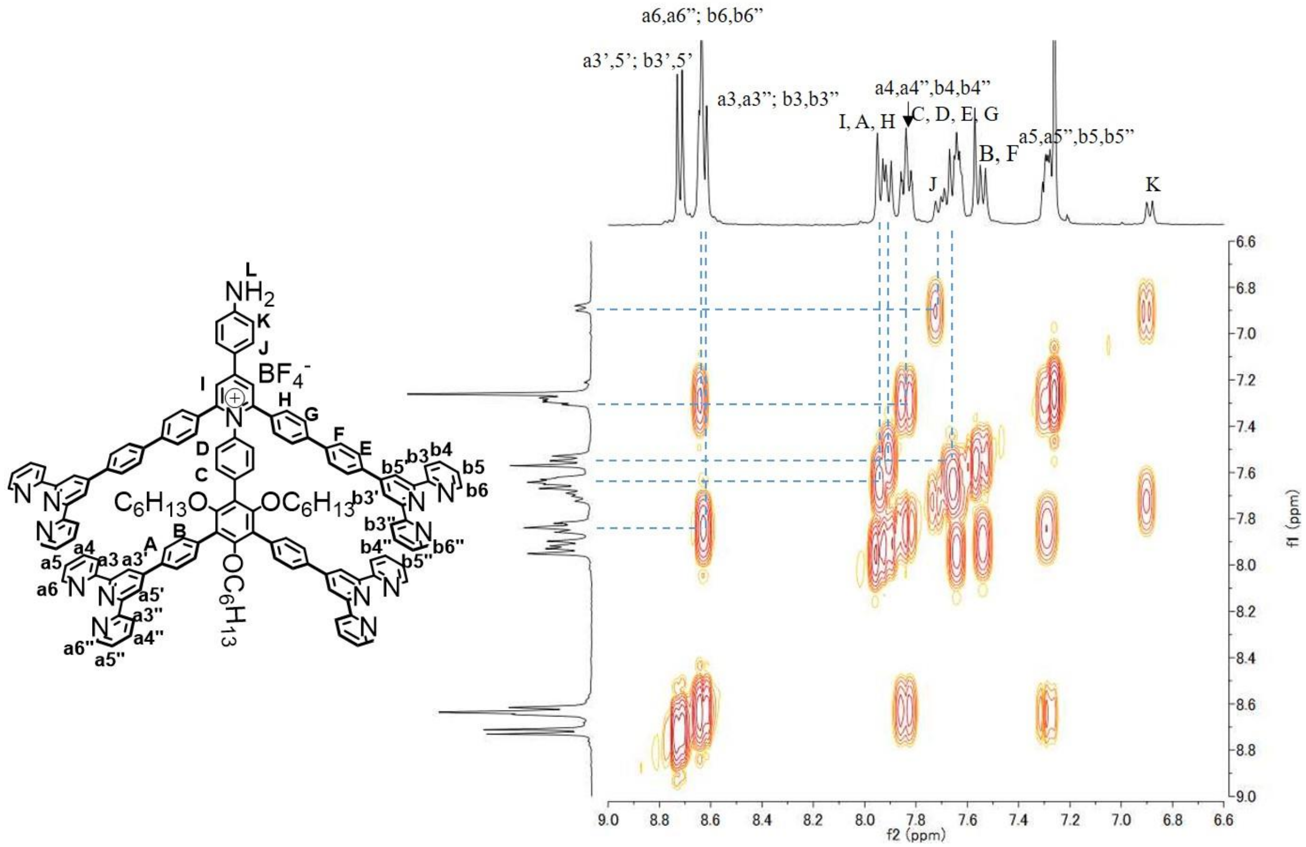

Supplementary Figure 40. 2D COSY NMR (400 MHz, $CDCl_3$, 300 K) spectrum (aromic region) of compound **13.**

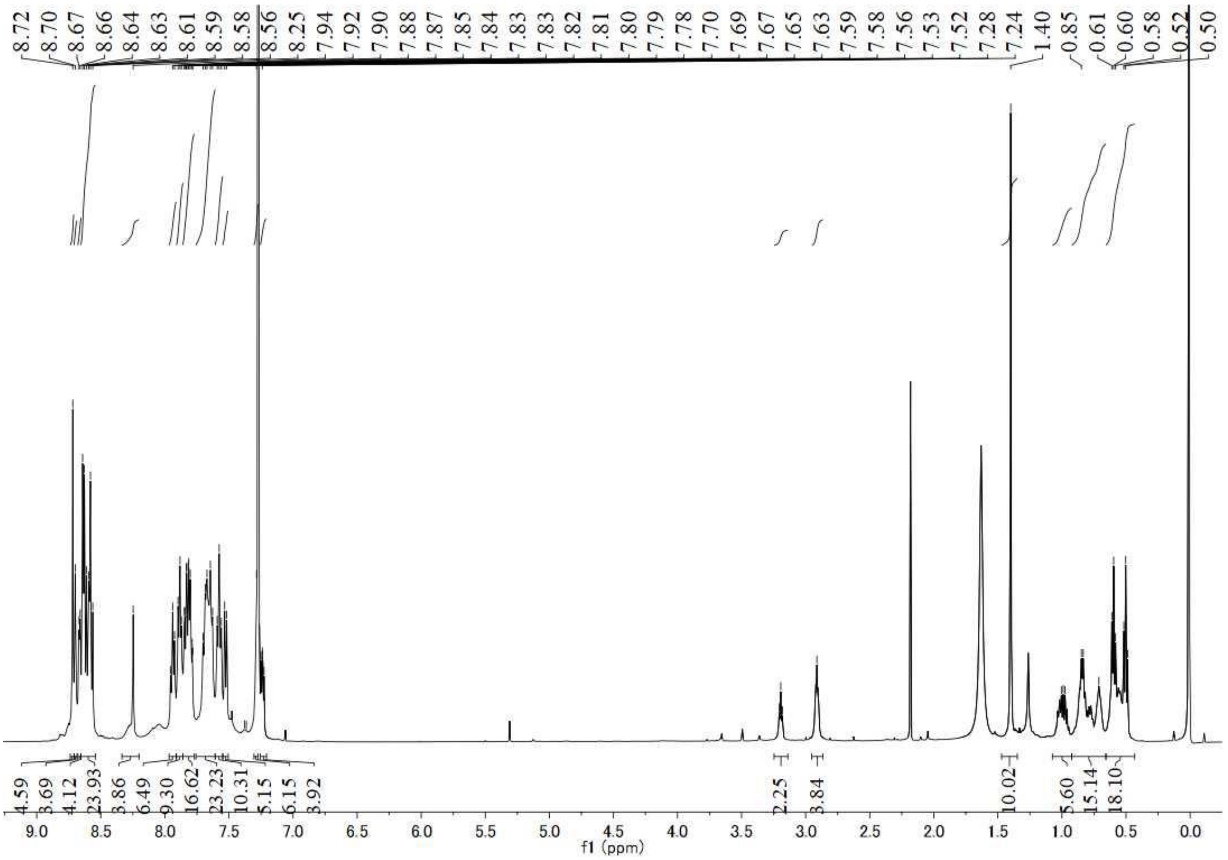

Supplementary Figure 41. ^{1}H NMR (500 MHz, $CDCl_3$, 300 K) spectrum of ligand **L3.**

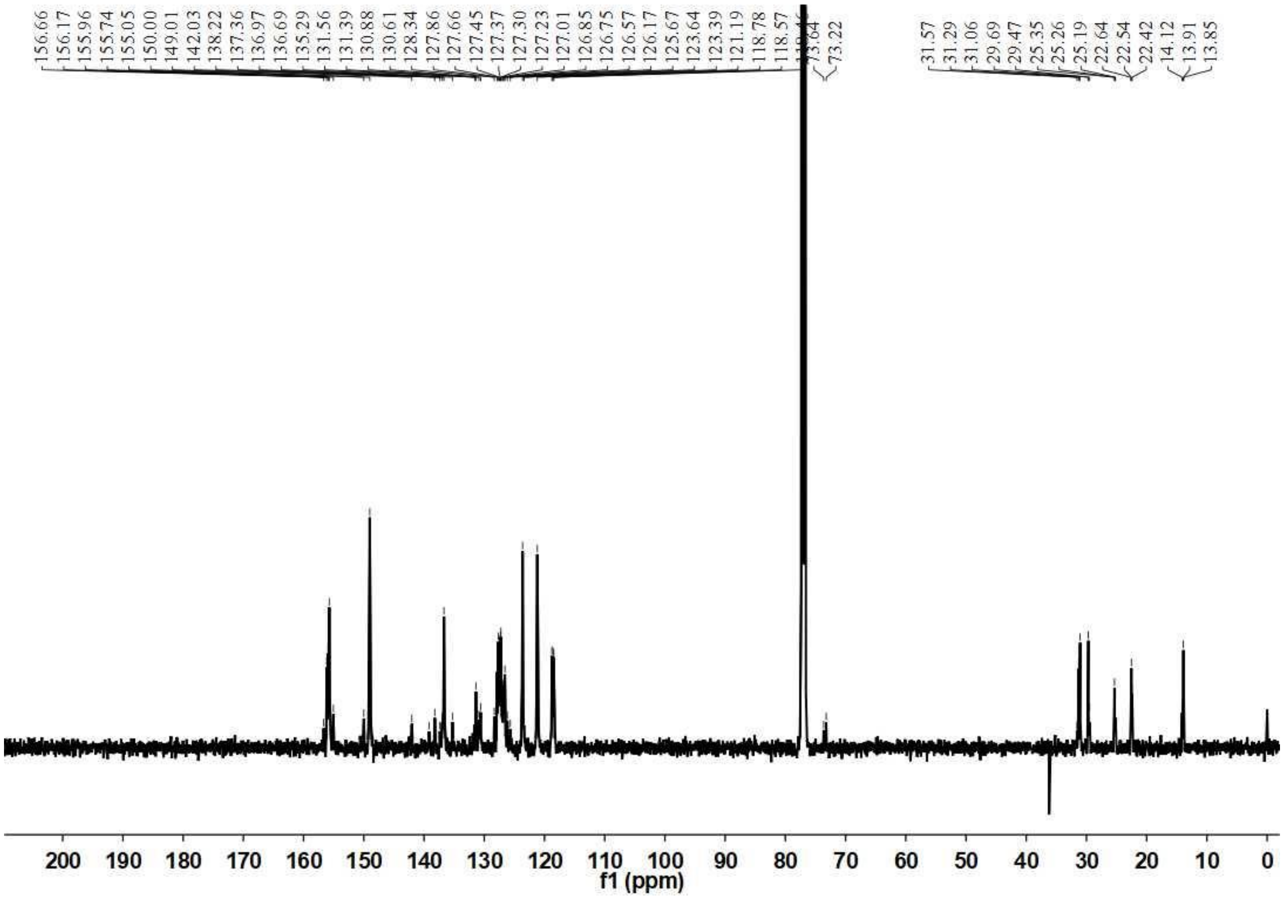

Supplementary Figure 42. ^{13}C NMR (125 MHz, $CDCl_3$, 300 K) spectrum of ligand **L3.**

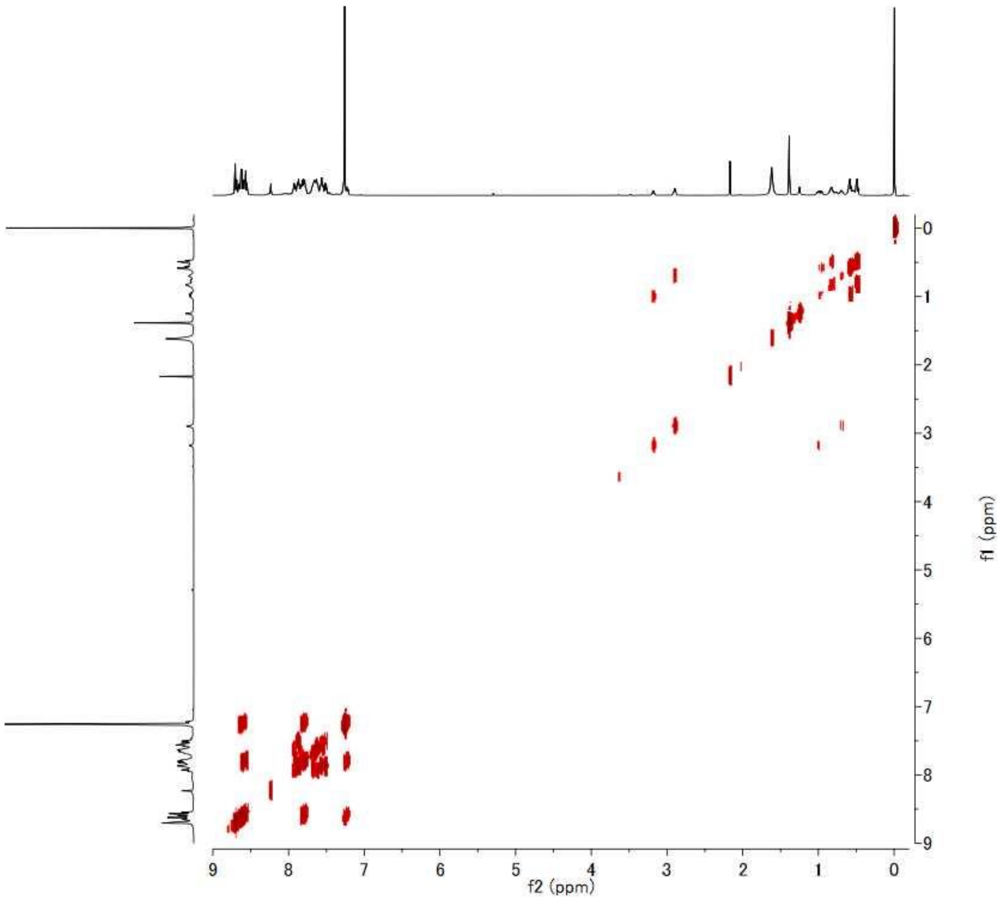

Supplementary Figure 43. 2D COSY NMR (500 MHz, $CDCl_3$, 300 K) spectrum of ligand **L3.**

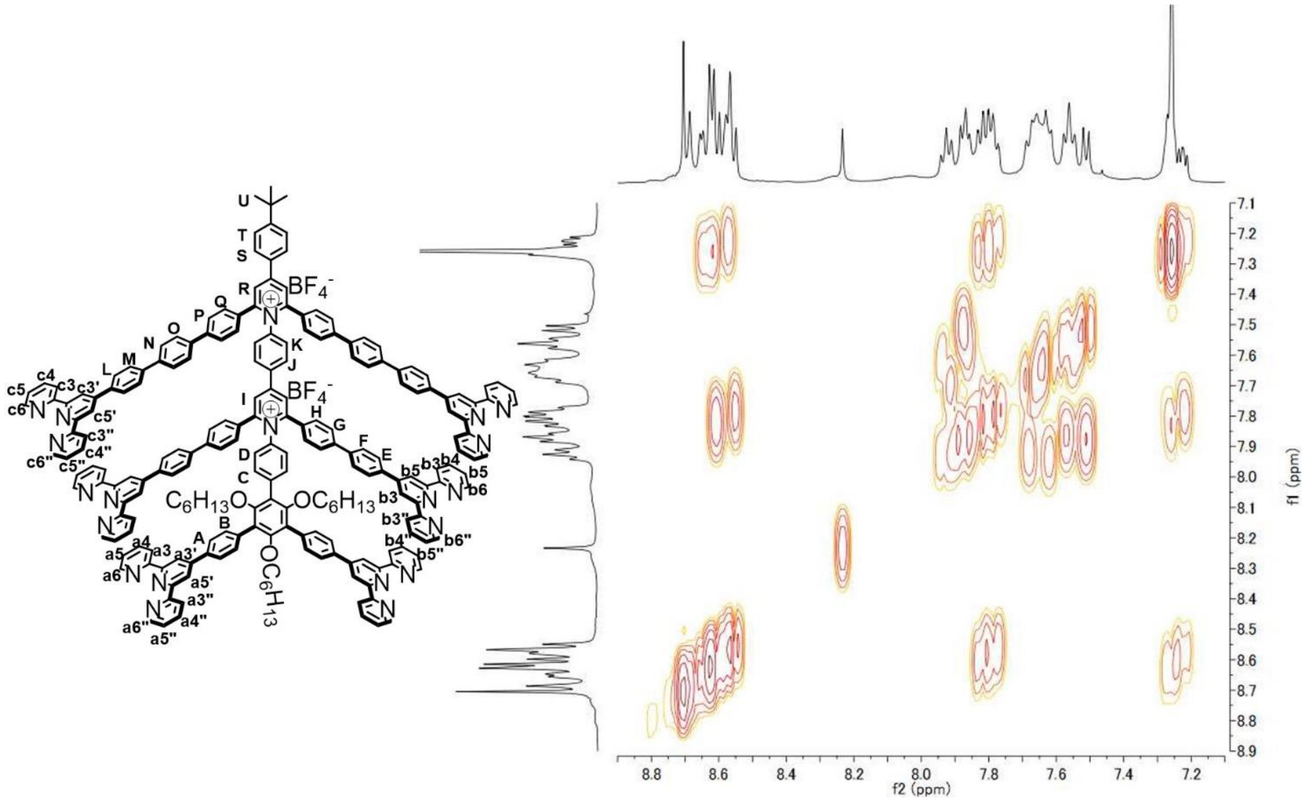

Supplementary Figure 44. 2D COSY NMR (500 MHz, $CDCl_3$, 300 K) spectrum (aromic region) of ligand **L3.**

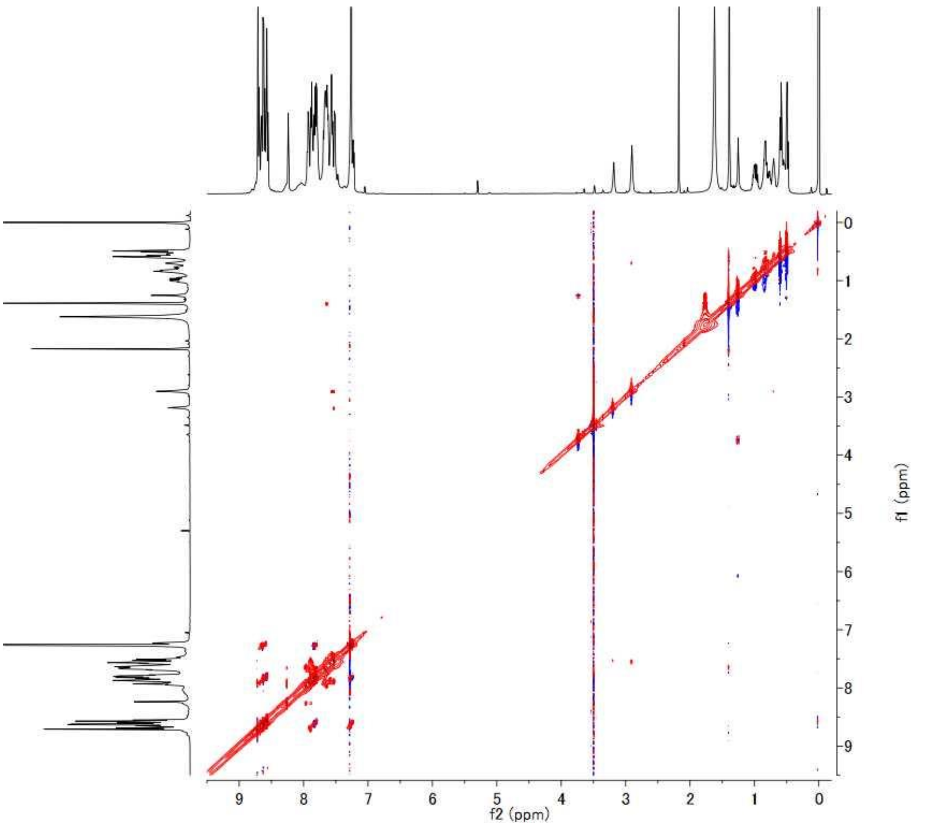

Supplementary Figure 45. 2D NOESY NMR (500 MHz, $CDCl_3$, 300 K) spectrum of ligand **L3.**

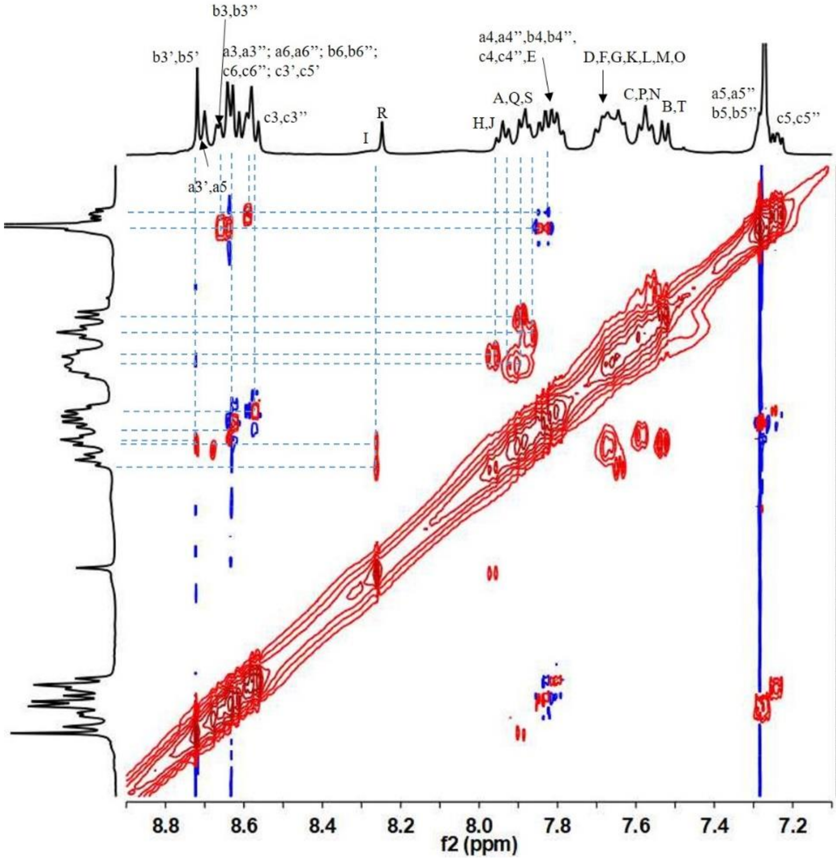

Supplementary Figure 46. 2D NOESY NMR (500 MHz, $CDCl_3$, 300 K) spectrum (aromic region) of ligand **L3.**

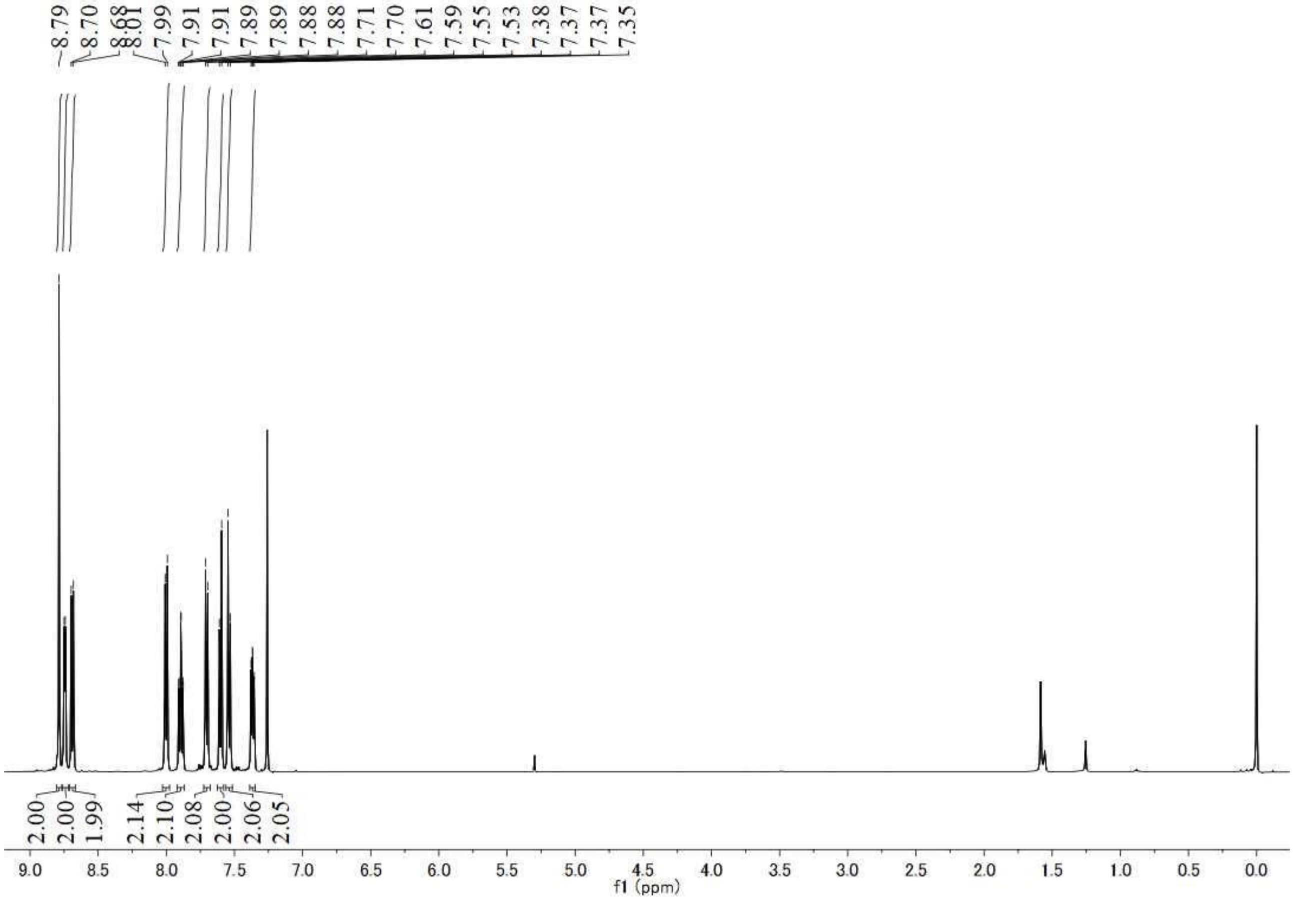

Supplementary Figure 47. ^{1}H NMR (500 MHz, $CDCl_3$, 300 K) spectrum of compound **14.**

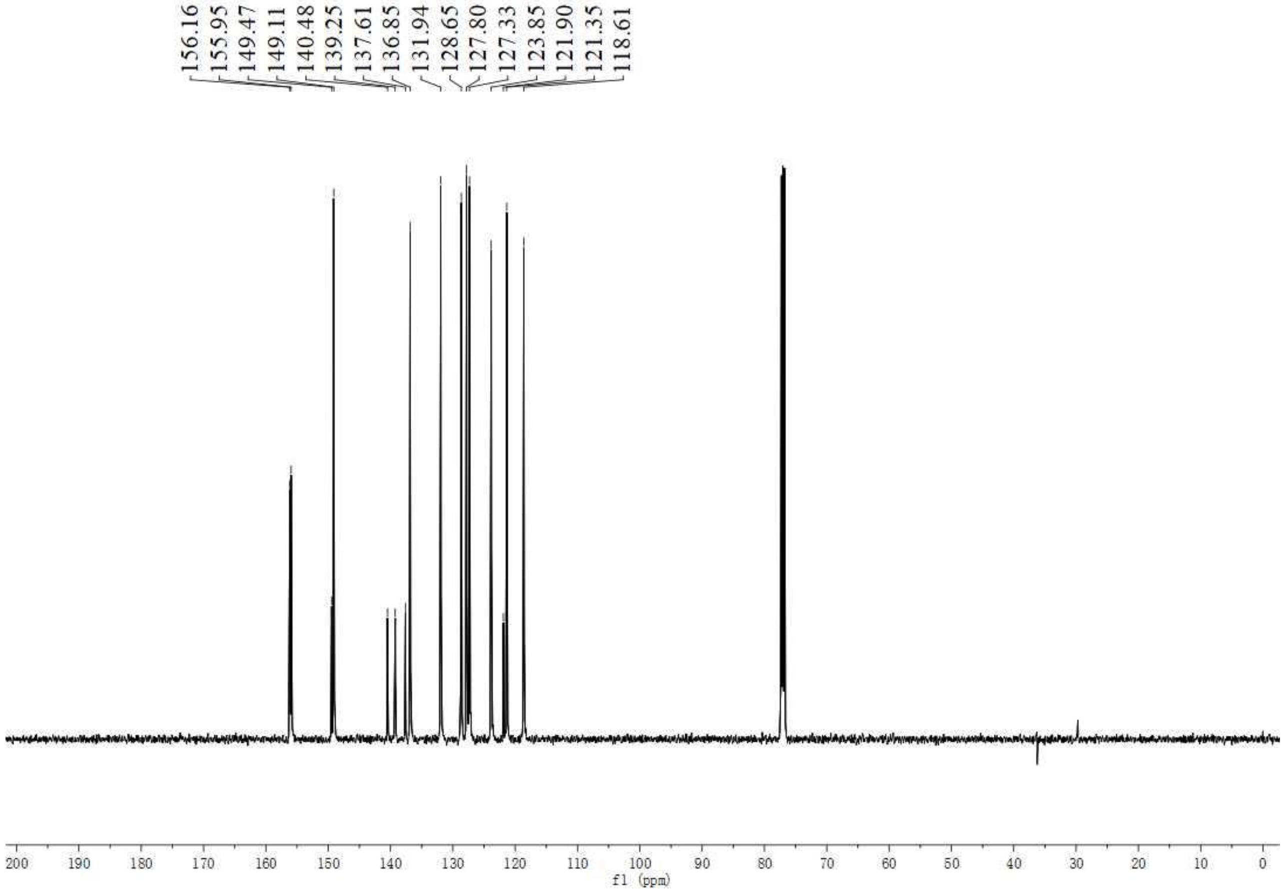

Supplementary Figure 48. ^{13}C NMR (125 MHz, $CDCl_3$, 300 K) spectrum of compound **14.**

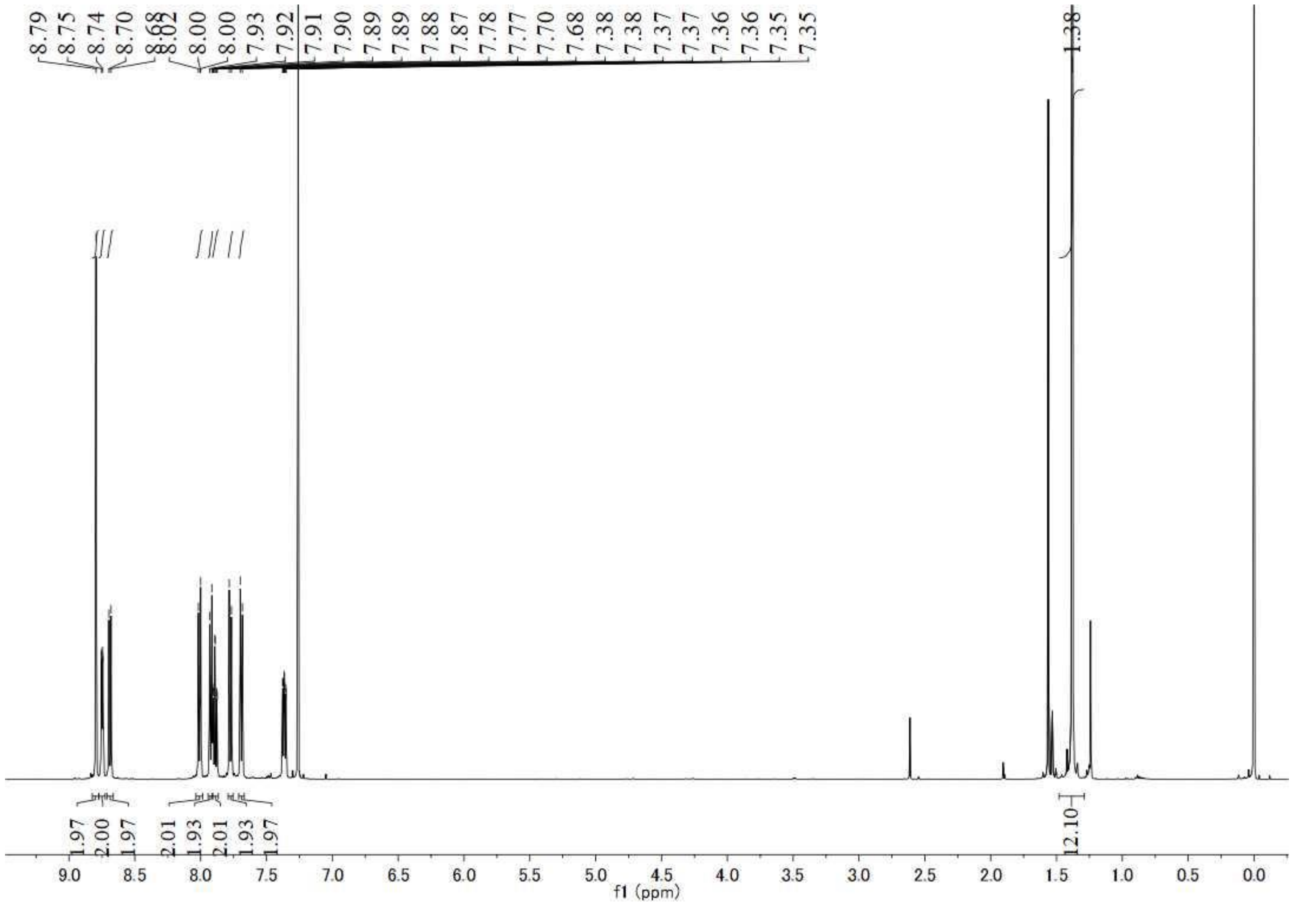

Supplementary Figure 49. ^{1}H NMR (400 MHz, $CDCl_3$, 300 K) spectrum of compound **15.**

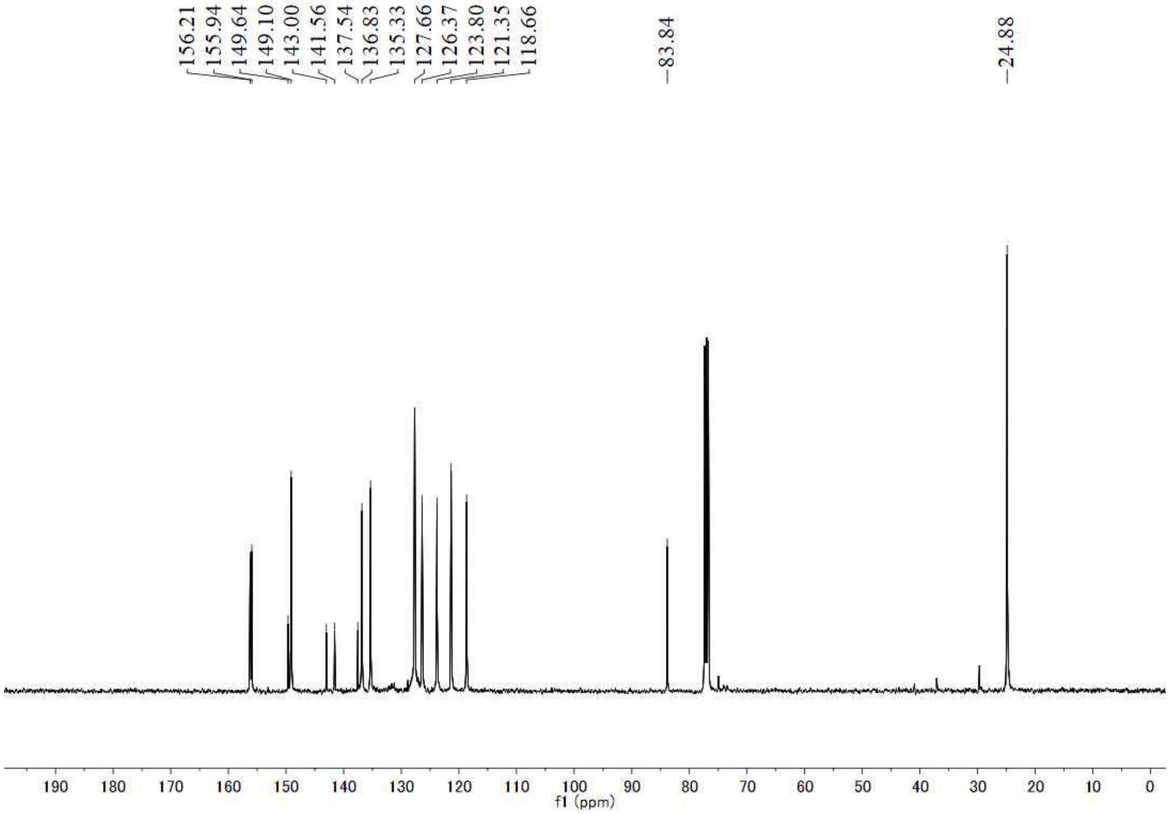

Supplementary Figure 50. ^{13}C NMR (100 MHz, $CDCl_3$, 300 K) spectrum of compound **15.**

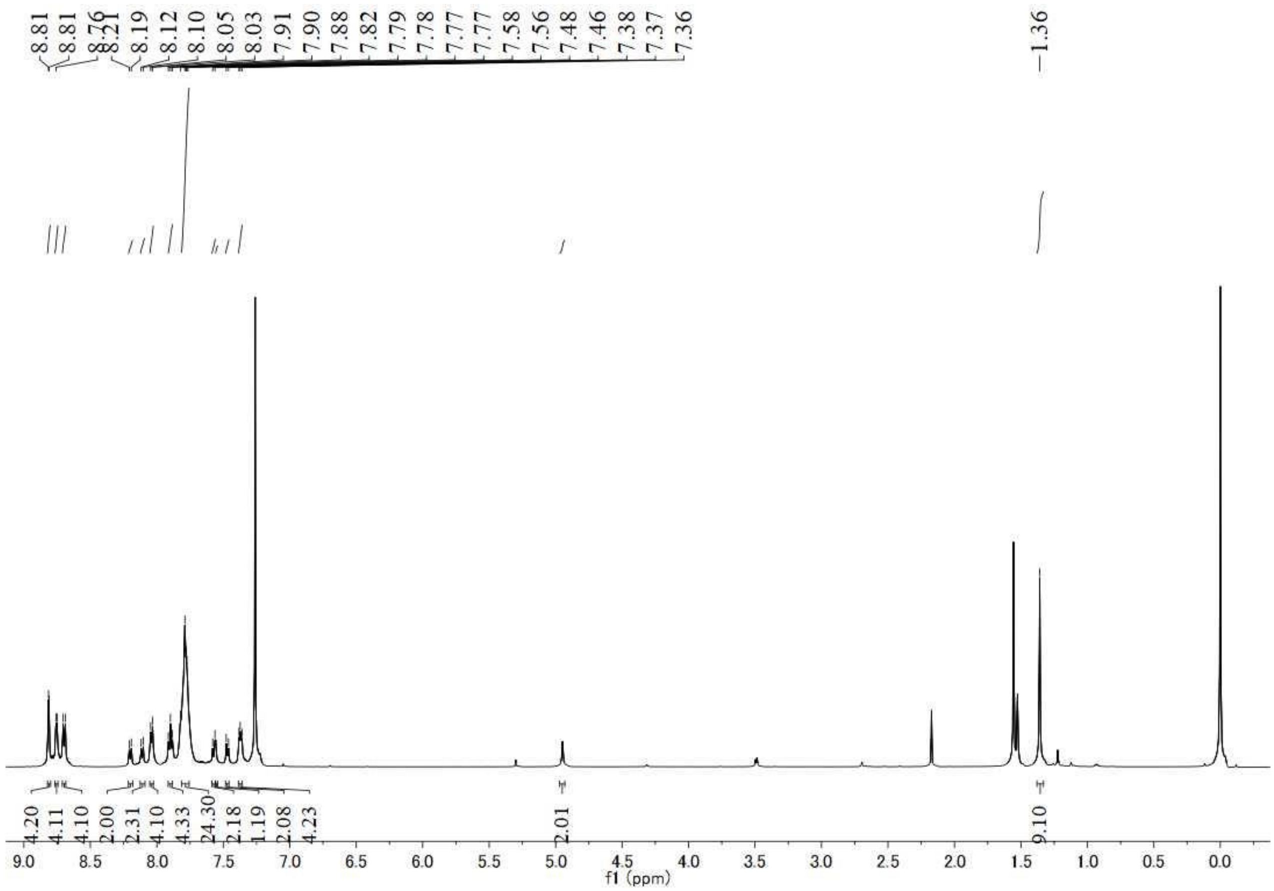

Supplementary Figure 51. ^{1}H NMR (500 MHz, $CDCl_3$, 300 K) spectrum of compound **16.**

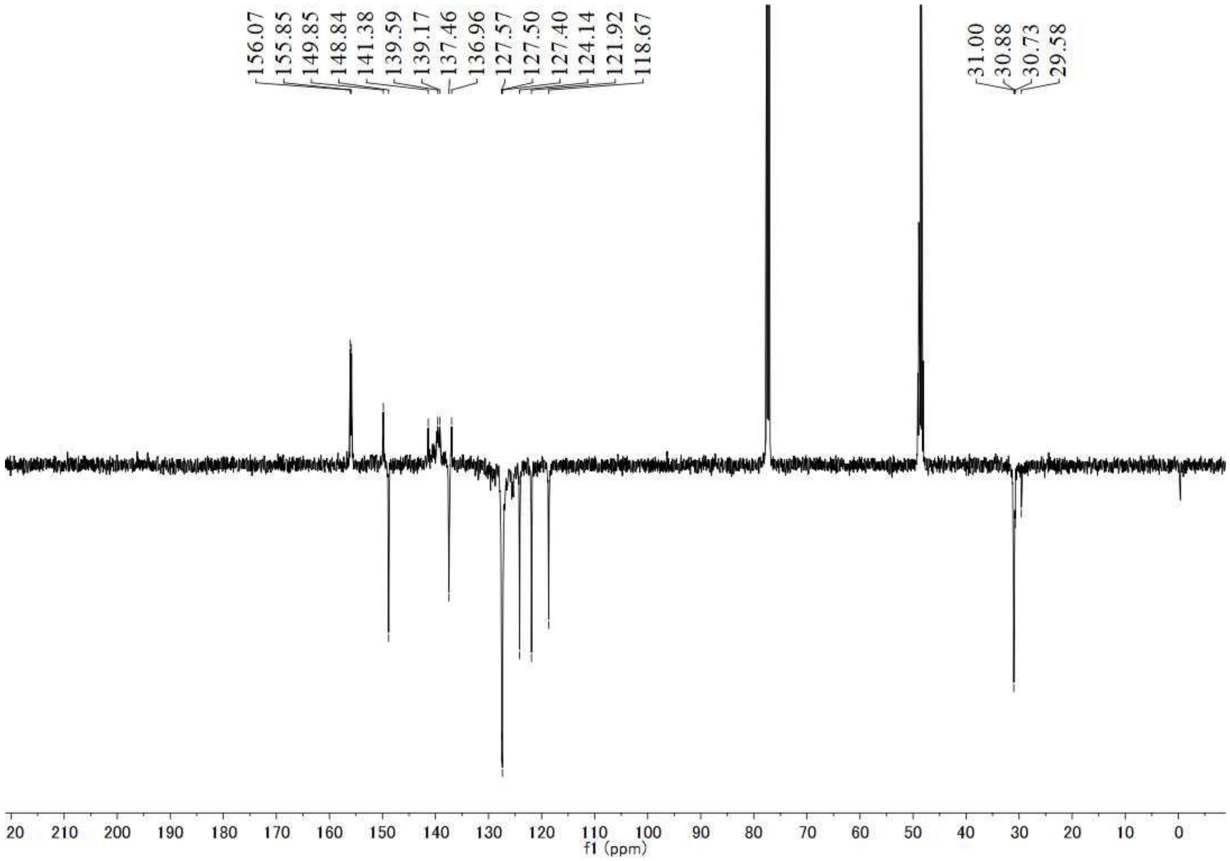

Supplementary Figure 52. ^{13}C DEPTQ NMR (125 MHz, $CD_3OD/CDCl_3$ 1/4, 300 K) spectrum of compound **16**.

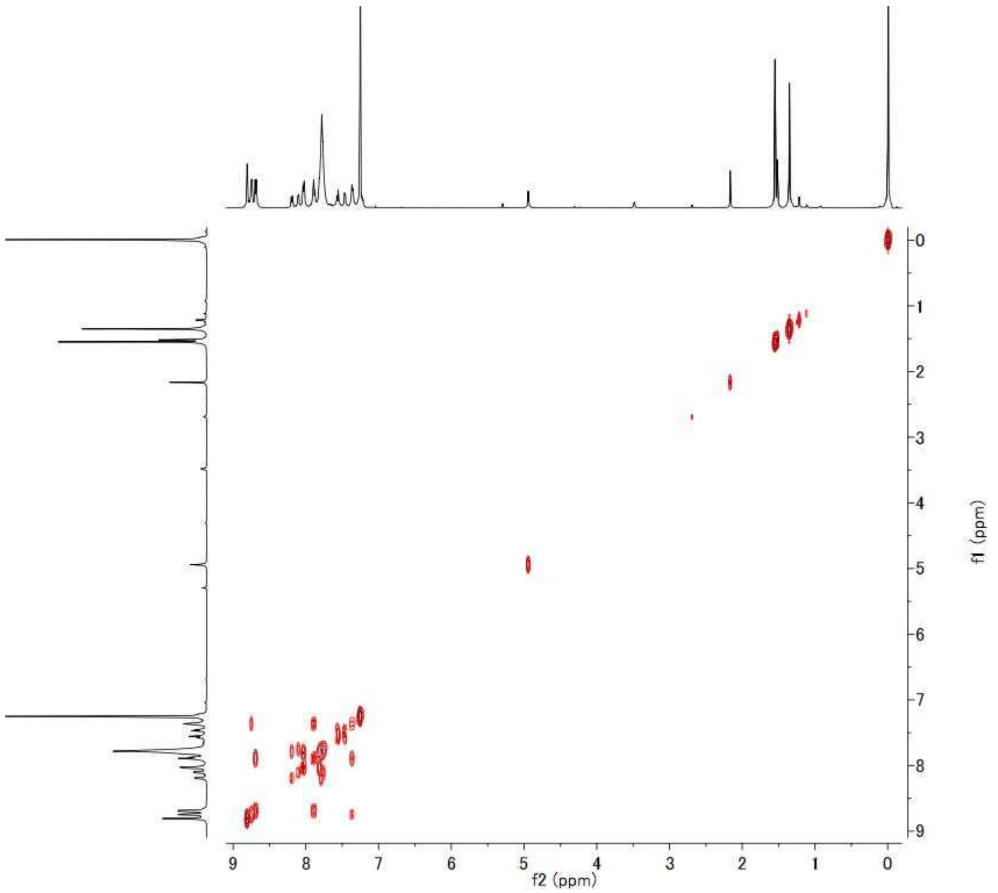

Supplementary Figure 53. 2D COSY NMR (500 MHz, $CDCl_3$, 300 K) spectrum of compound **16.**

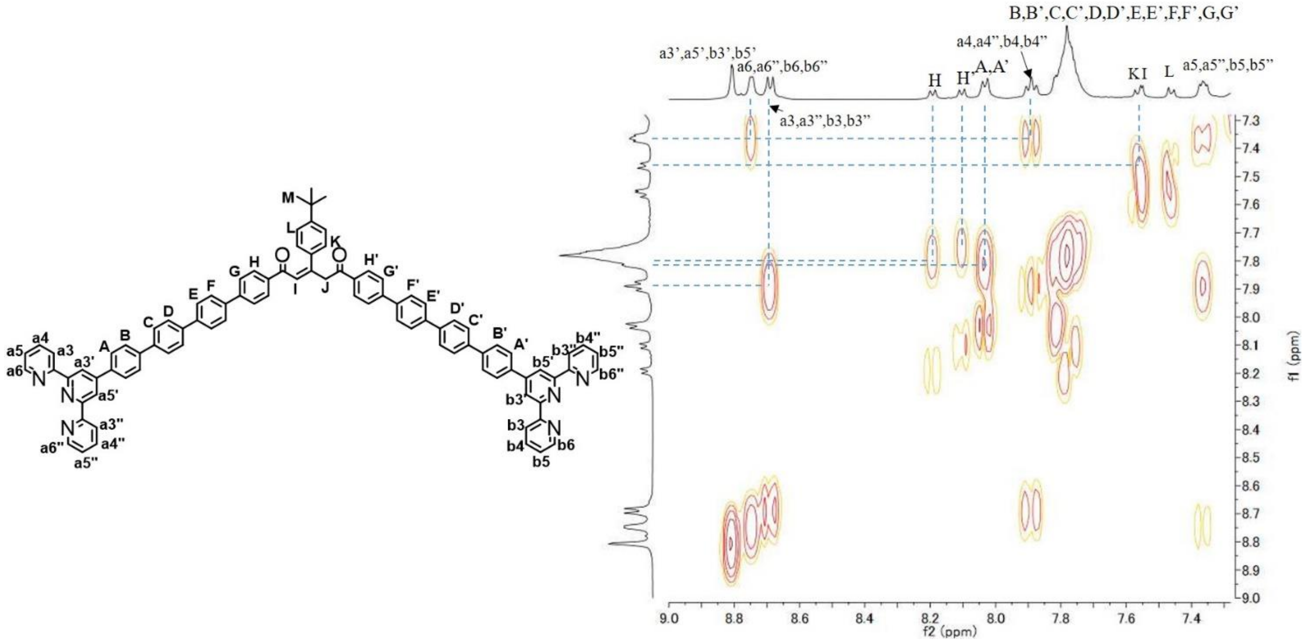

Supplementary Figure 54. 2D COSY NMR (500 MHz, $CDCl_3$, 300 K) spectrum (aromic region) of compound **16.**

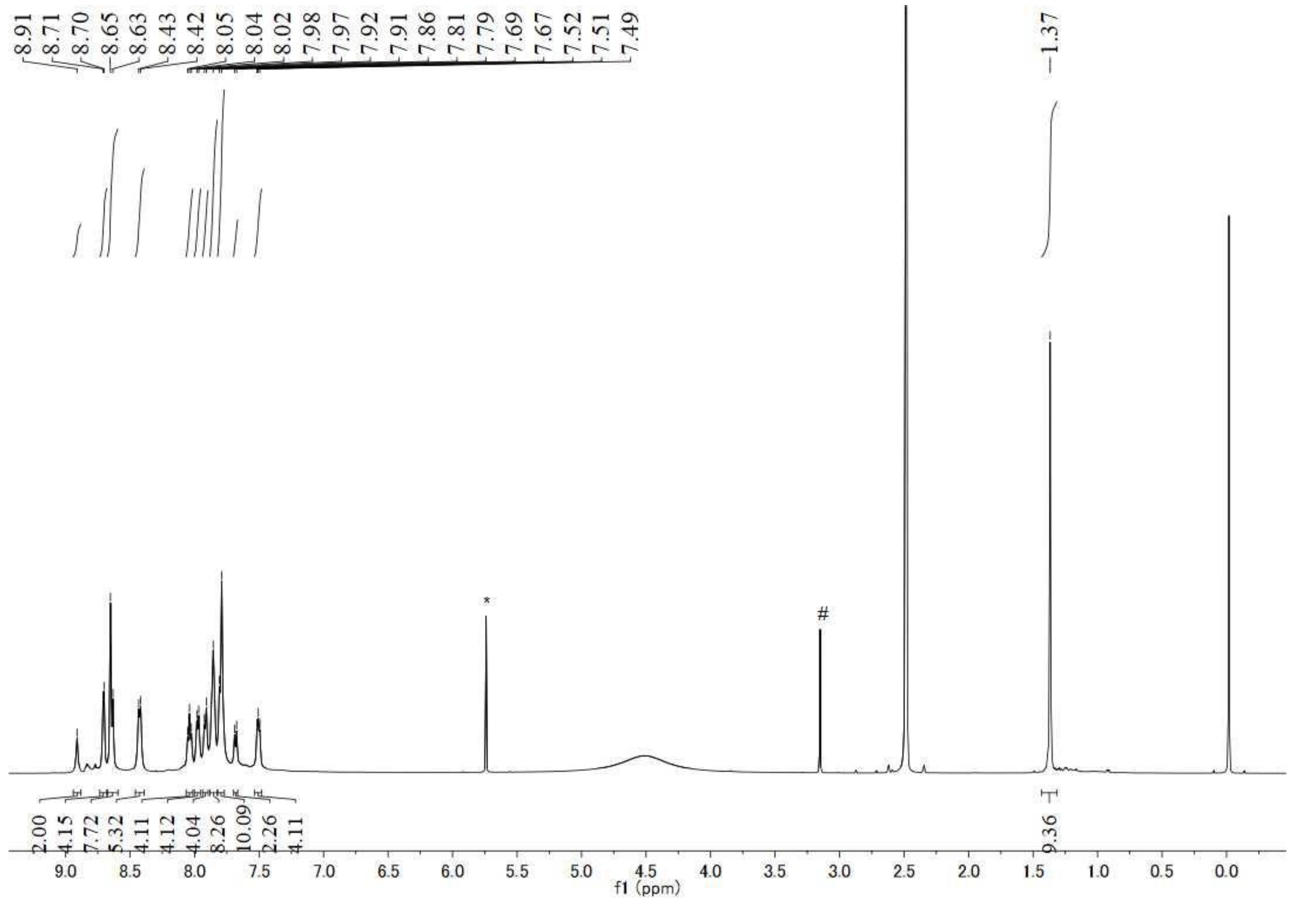

Supplementary Figure 55. ^{1}H NMR (500 MHz, DMSO-*d*$_6$, 300 K) spectrum of compound **17**, * and # refer to the peak of DCM and methanol.

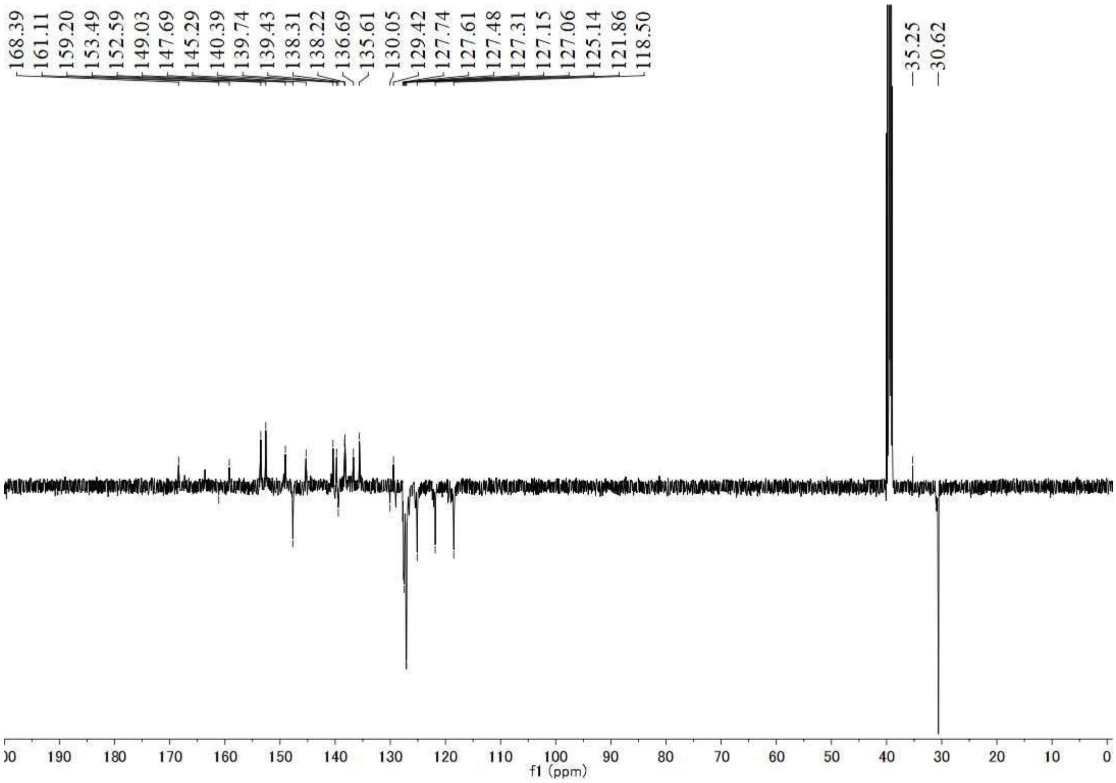

Supplementary Figure 56. ^{13}C DEPTQ NMR (125 MHz, $CD_3OD/CDCl_3$ 1/4, 300 K) spectrum of compound **17.**

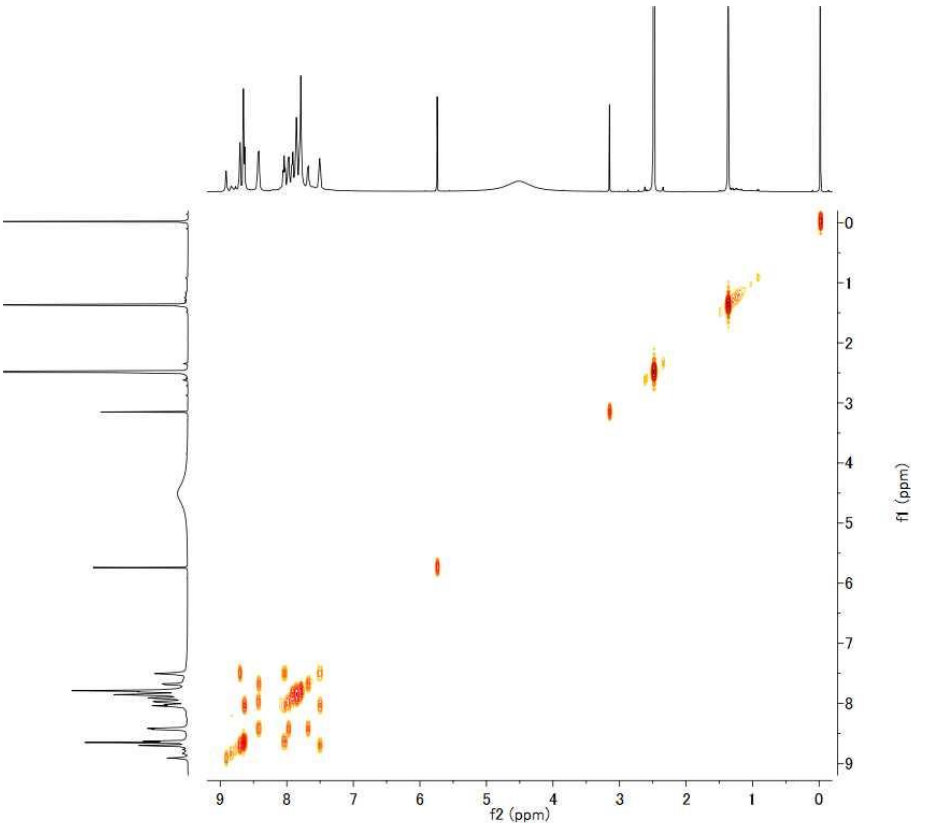

Supplementary Figure 57. 2D COSY NMR (500 MHz, DMSO-d_6, 300 K) spectrum of compound **17.**

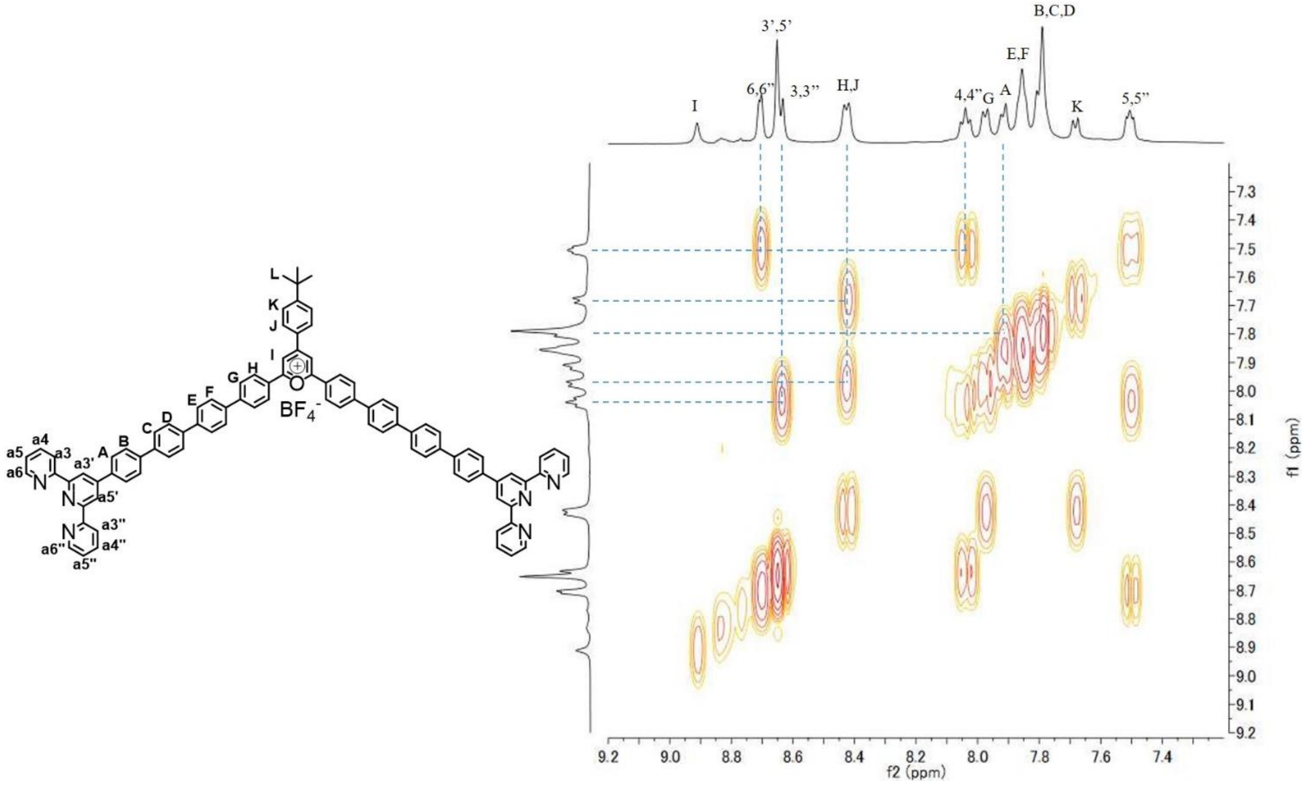

Supplementary Figure 58. 2D COSY NMR (500 MHz, DMSO-d_6, 300 K) spectrum (aromic region) of compound **17.**

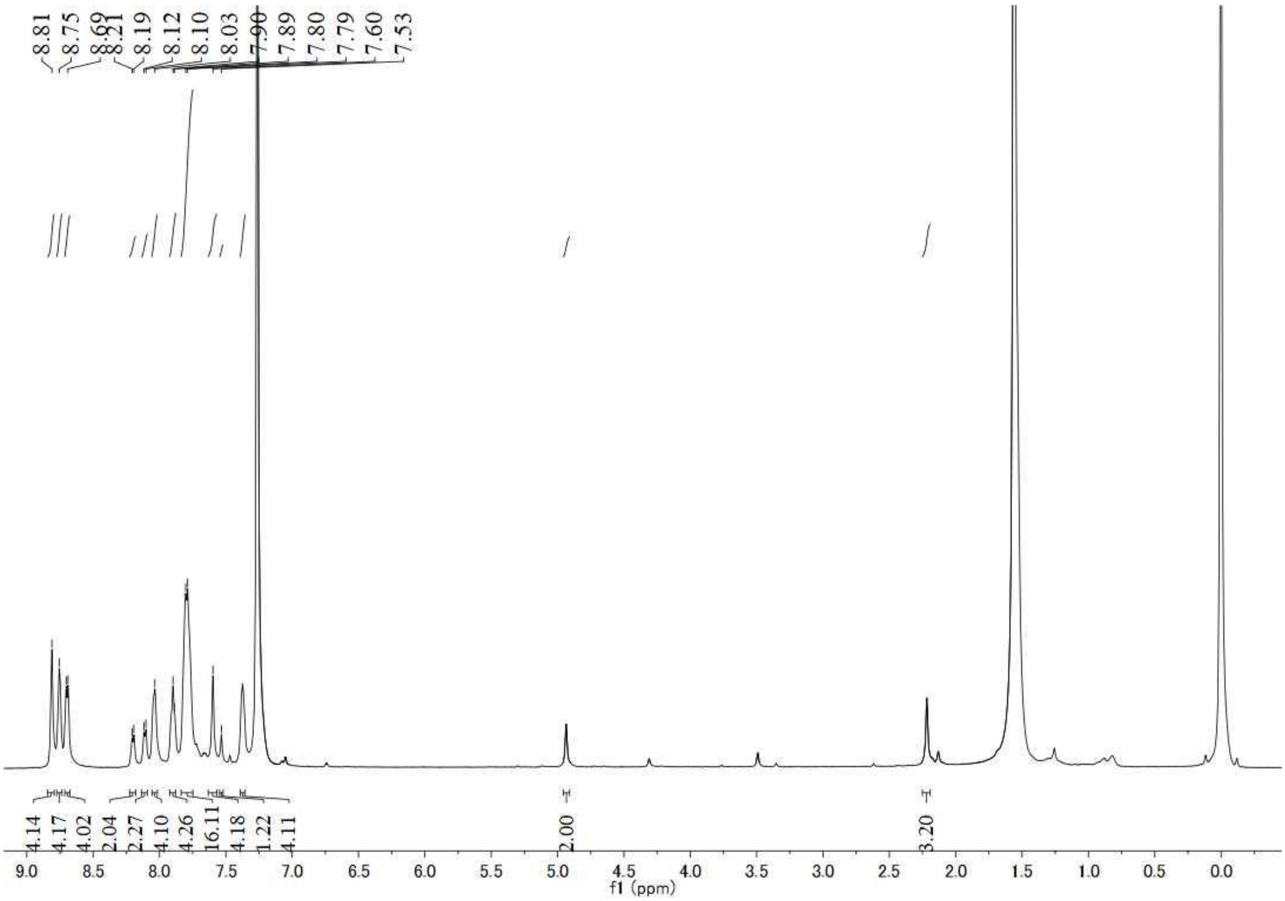

Supplementary Figure 59. ^{1}H NMR (500 MHz, $CDCl_3$, 300 K) spectrum of compound **18.**

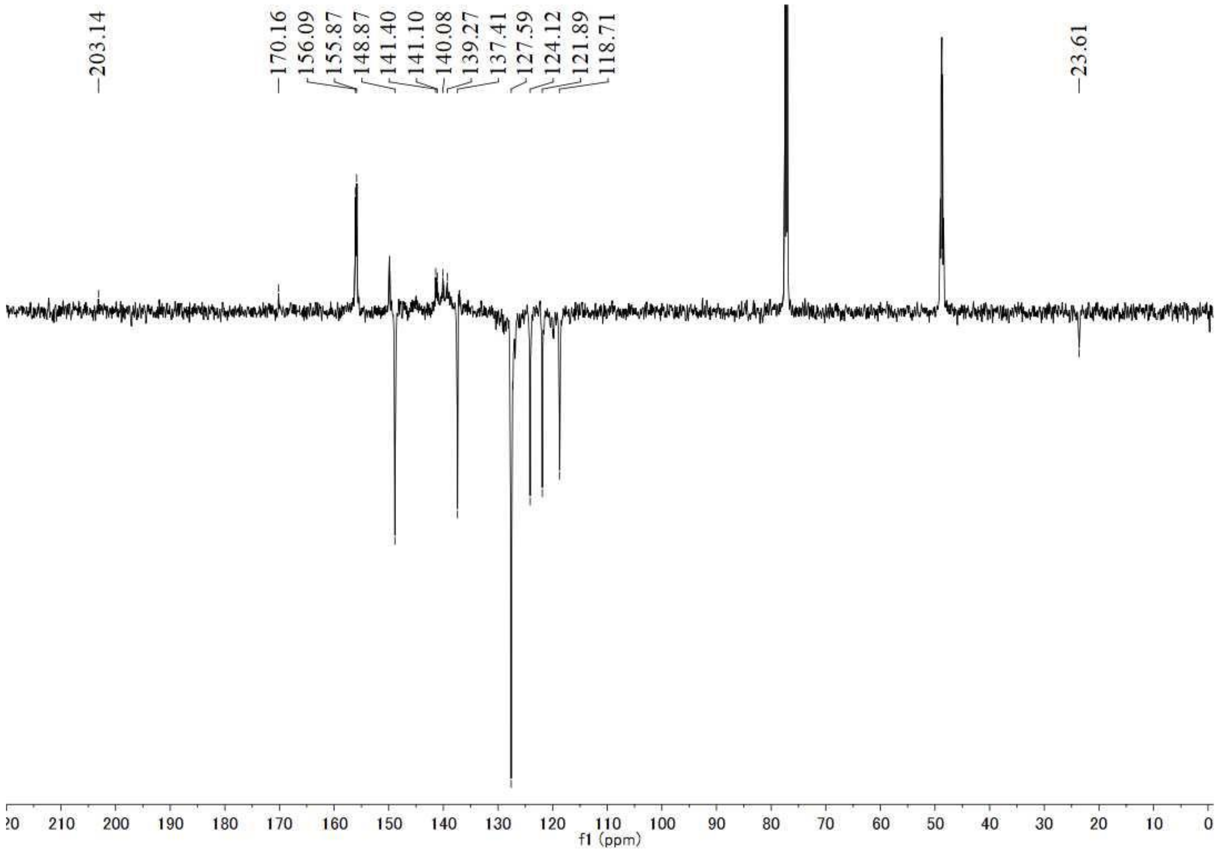

Supplementary Figure 60. ^{13}C DEPTQ NMR (150 MHz, $CD_3OD/CDCl_3$ 1/4) spectrum of compound **18**.

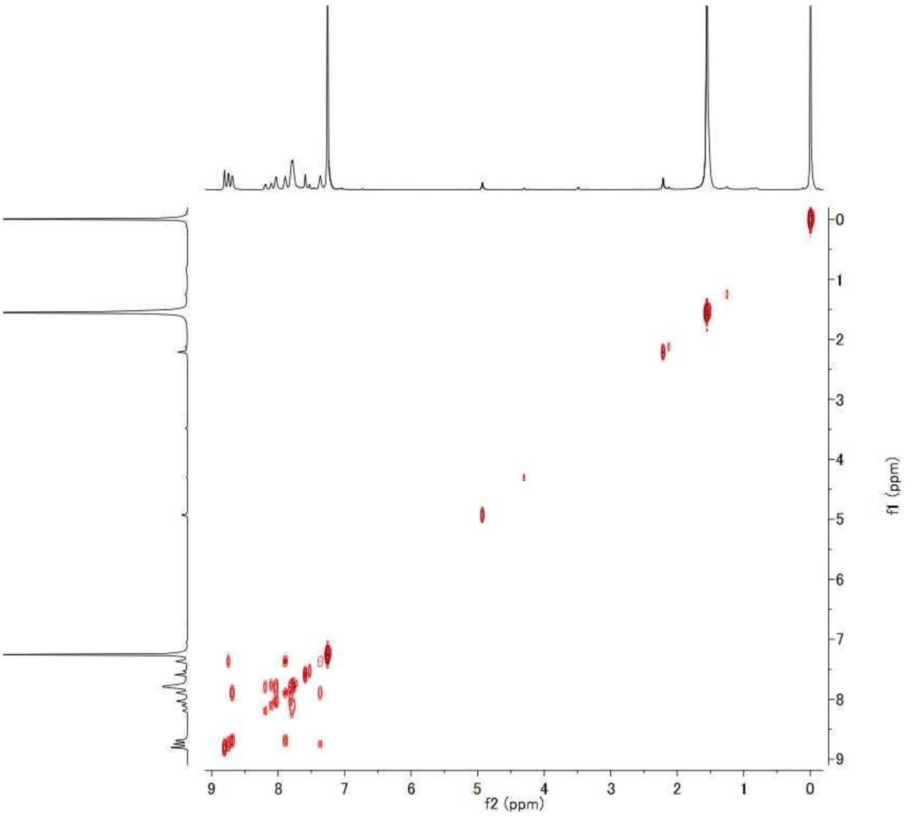

Supplementary Figure 61. 2D COSY NMR (500 MHz, $CDCl_3$, 300 K) spectrum of compound **18.**

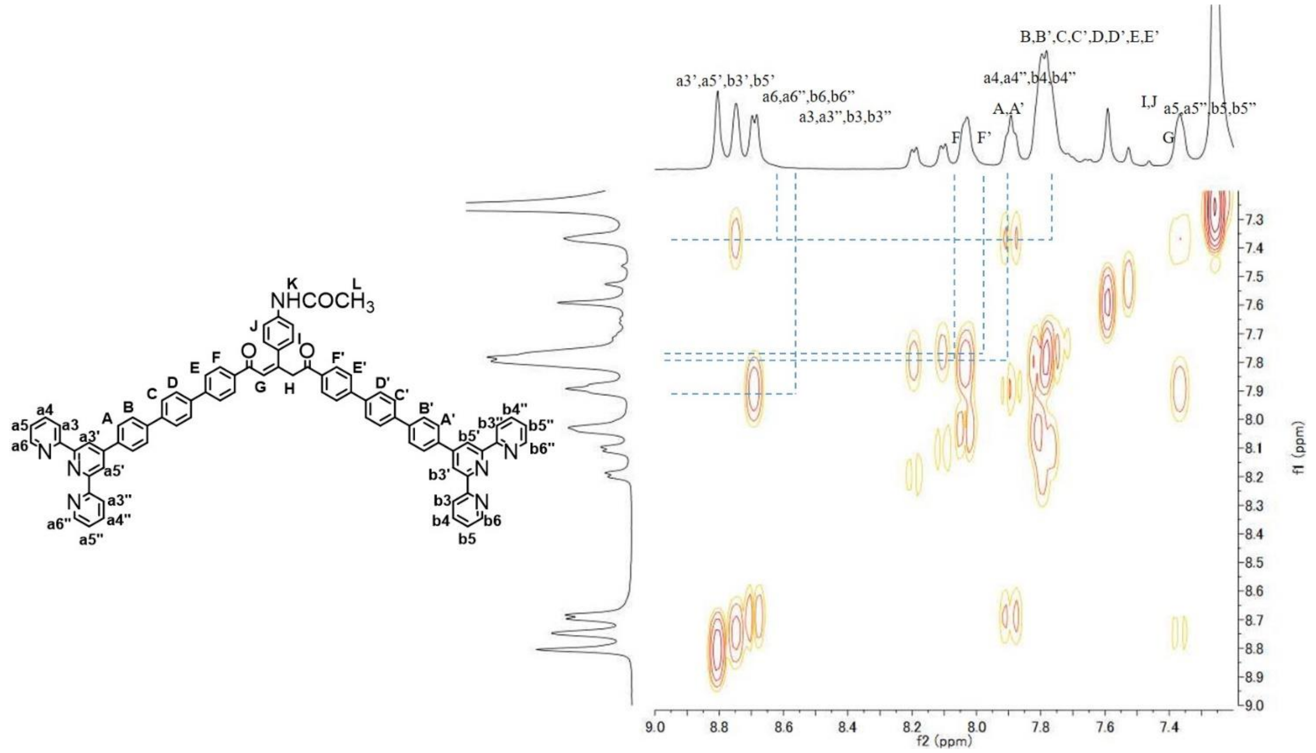

Supplementary Figure 62. 2D COSY NMR (500 MHz, $CDCl_3$, 300 K) spectrum (aromic region) of compound **18.**

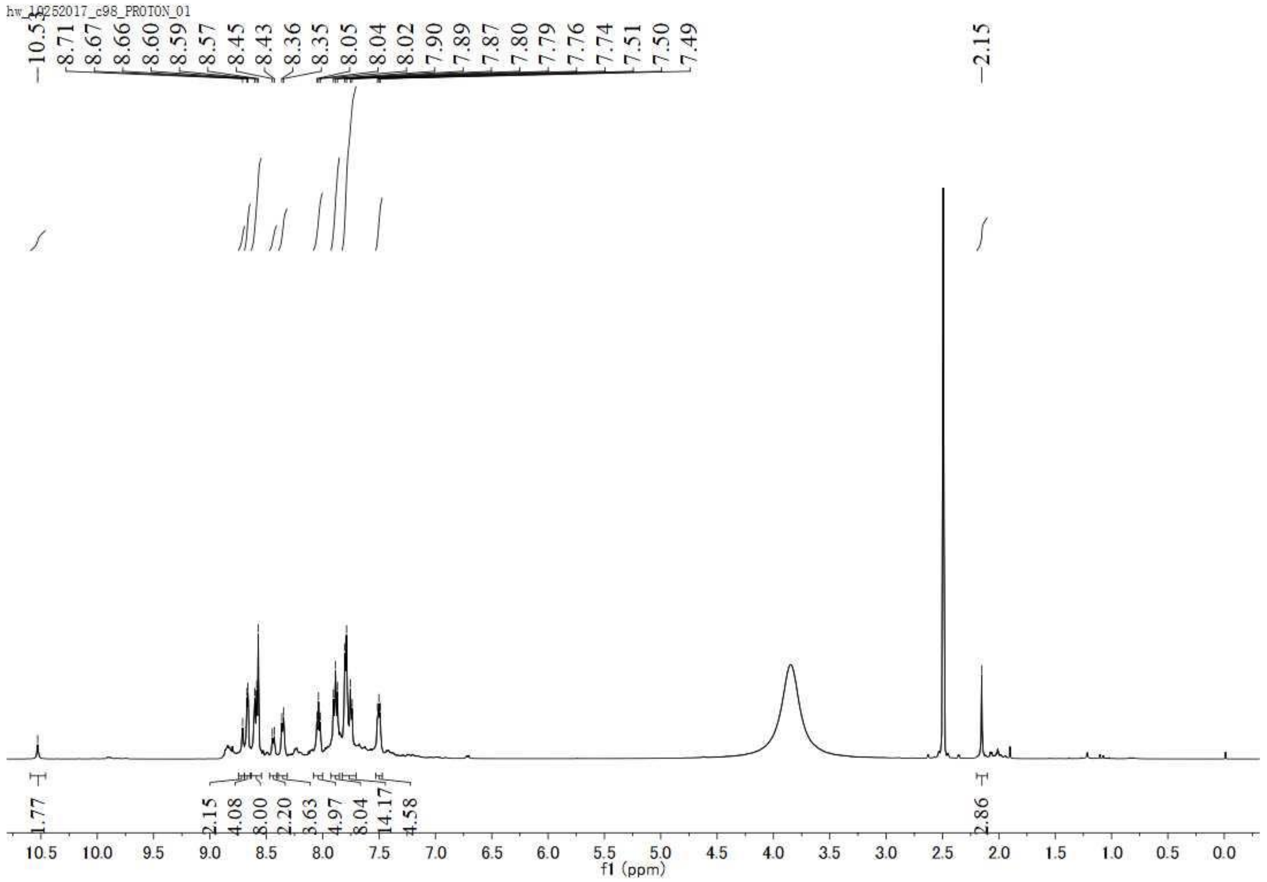

Supplementary Figure 63. ^{1}H NMR (500 MHz, DMSO-d_6, 300 K) spectrum of compound **19.**

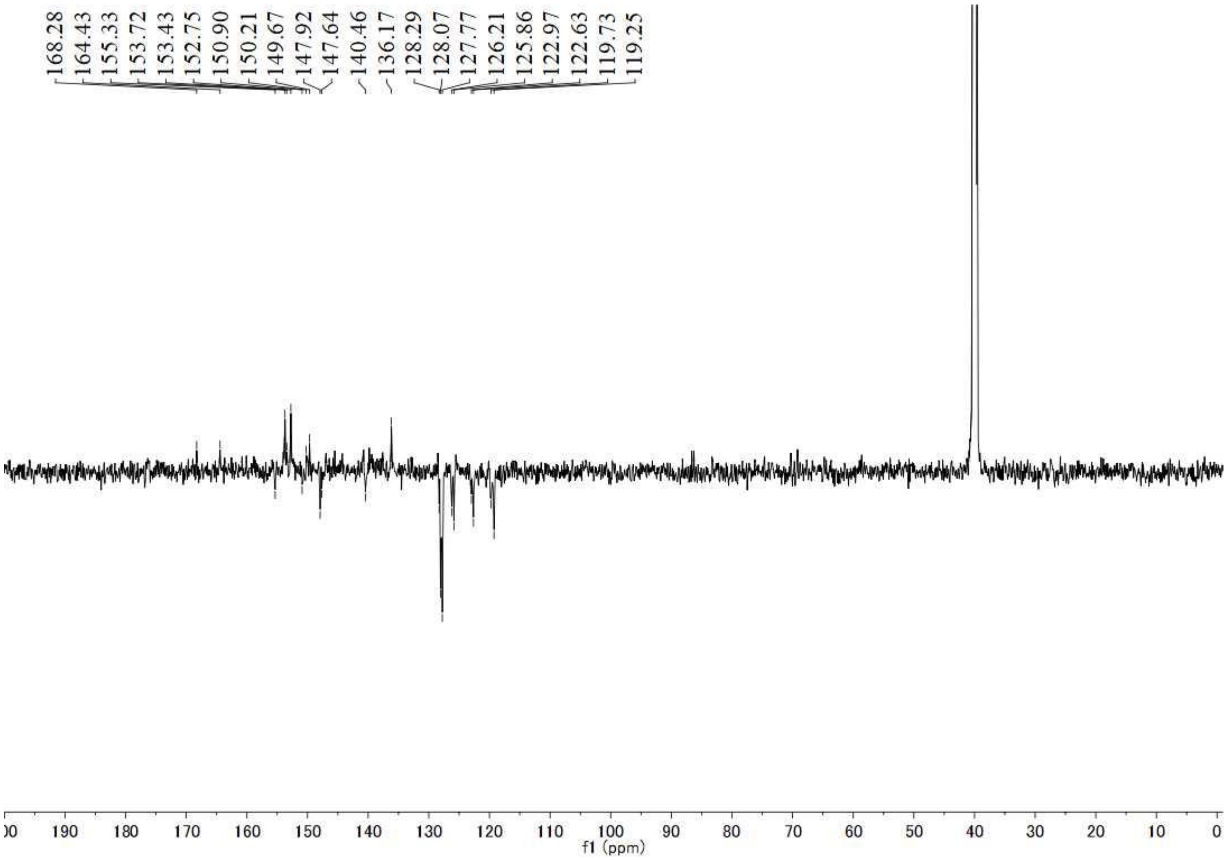

Supplementary Figure 64. ^{13}C DEPTQ NMR (150 MHz, DMSO-d_6, 300 K) spectrum of compound **19.**

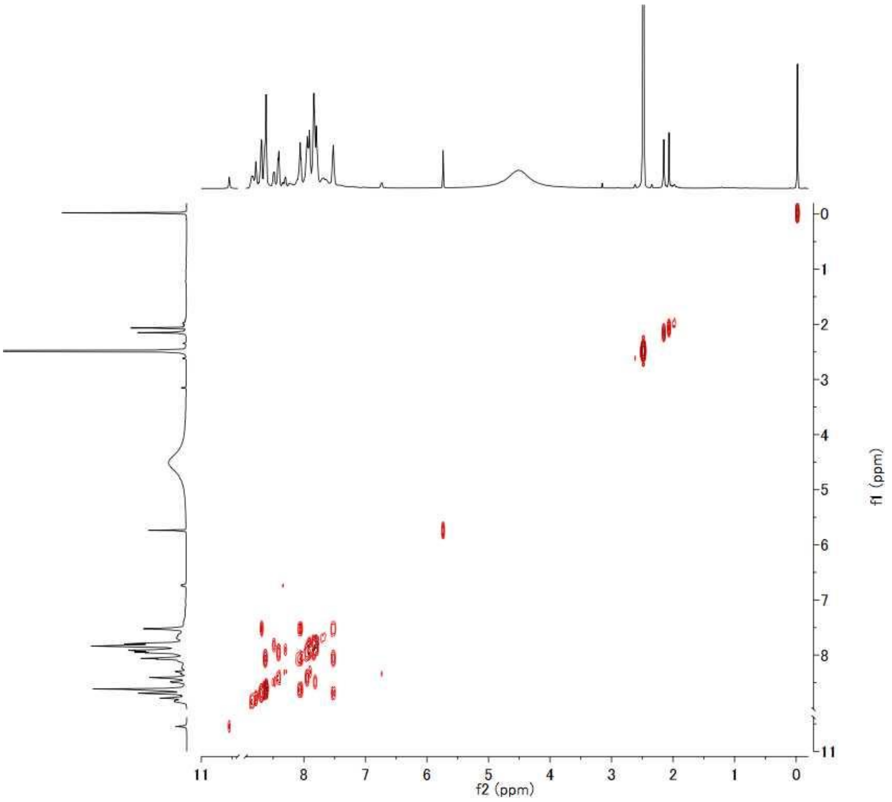

Supplementary Figure 65. 2D COSY NMR (500 MHz, DMSO-d_6, 300 K) spectrum of compound **19.**

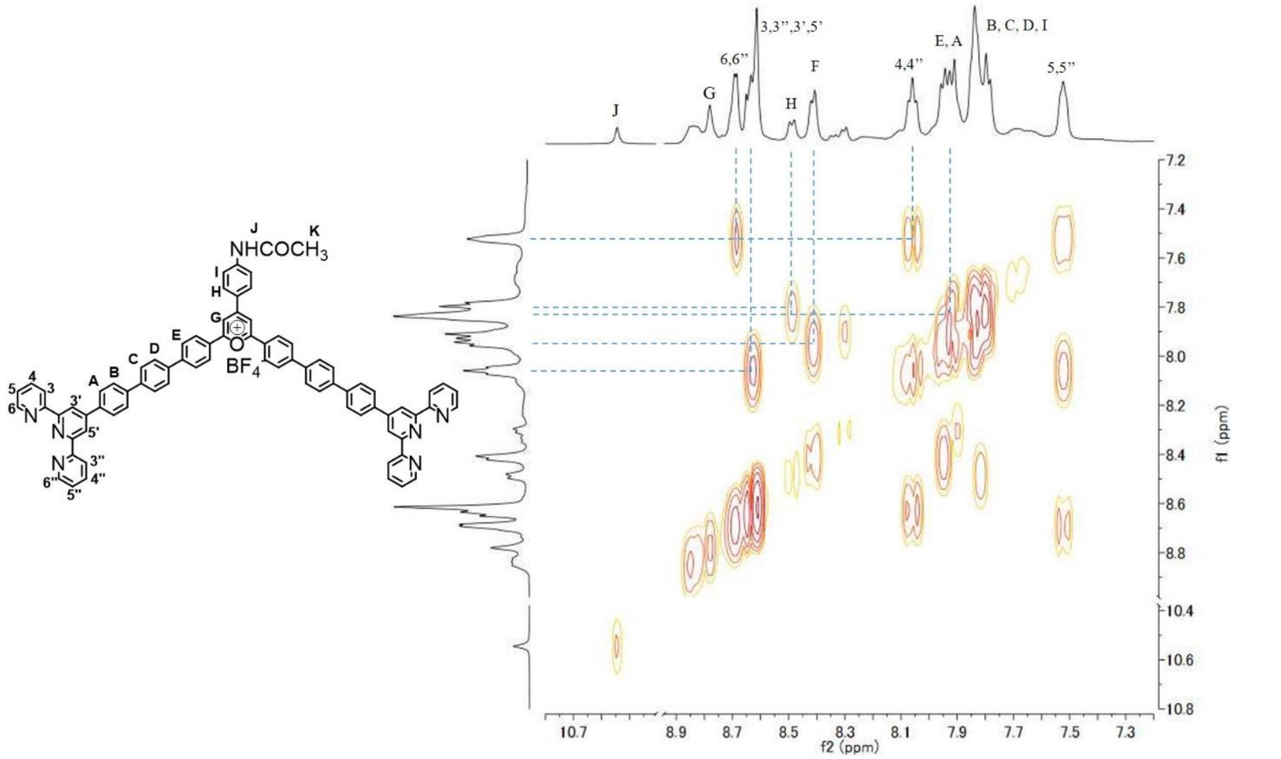

Supplementary Figure 66. 2D COSY NMR (500 MHz, DMSO-d_6, 300 K) spectrum (aromic region) of compound **19.**

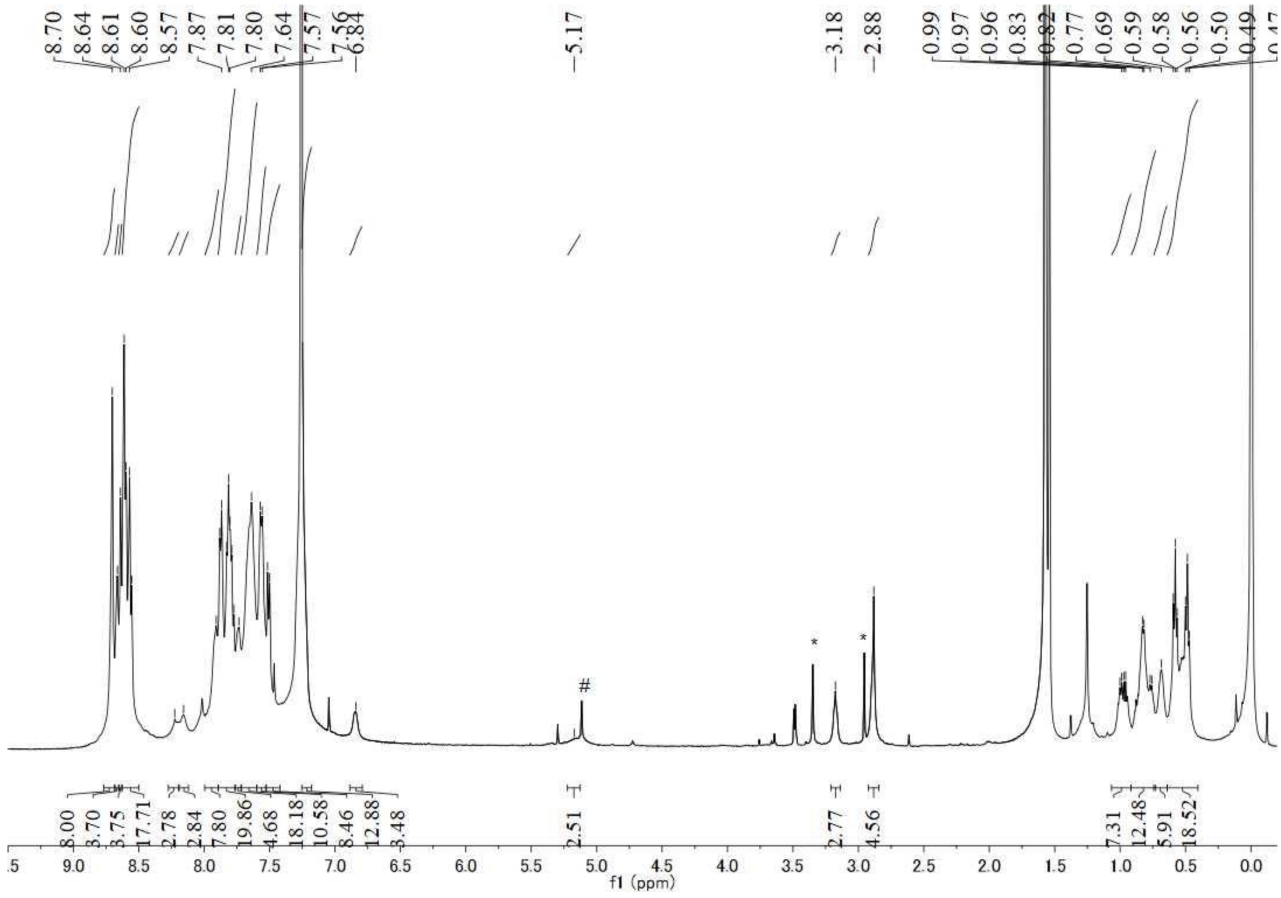

Supplementary Figure 67. ^{1}H NMR (500 MHz, $CDCl_3$, 300 K) spectrum of compound **20**, # and * refer to the peak of DCM and methanol**.**

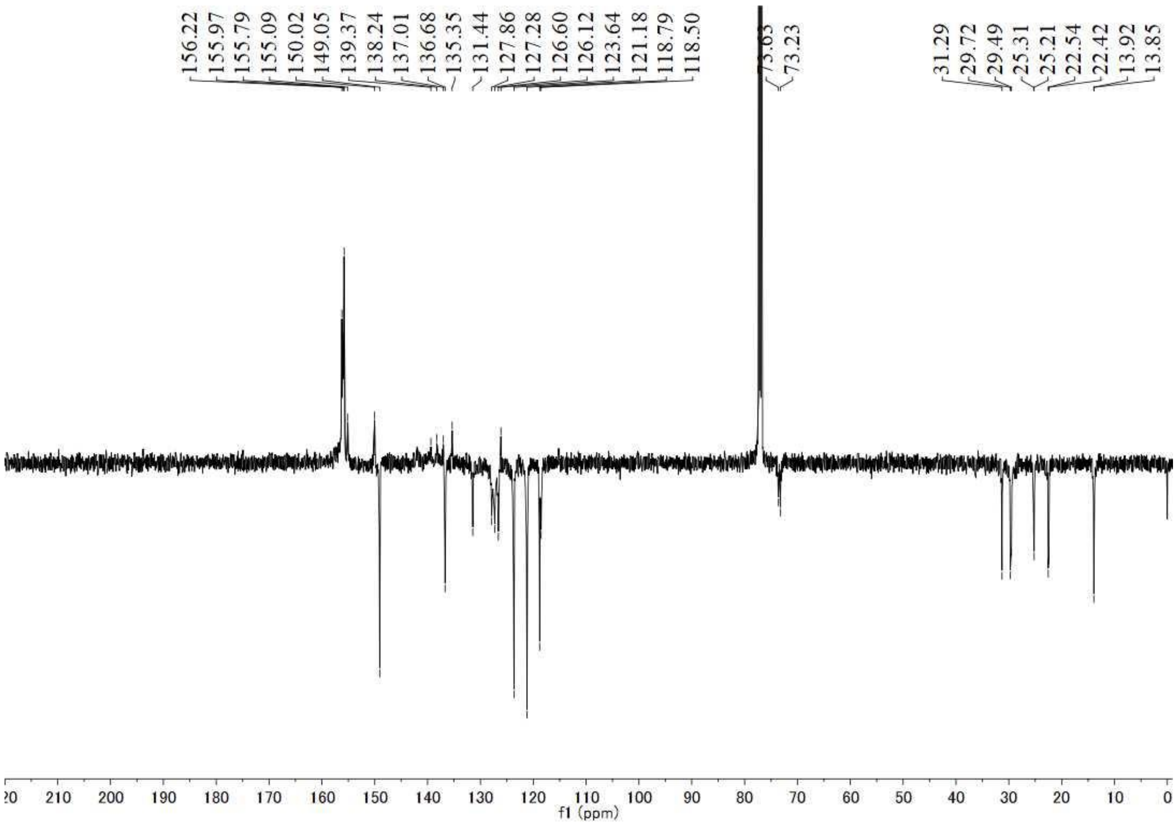

Supplementary Figure 68. ^{13}C DEPTQ NMR (125 MHz, $CDCl_3$, 300 K) spectrum of compound **20.**

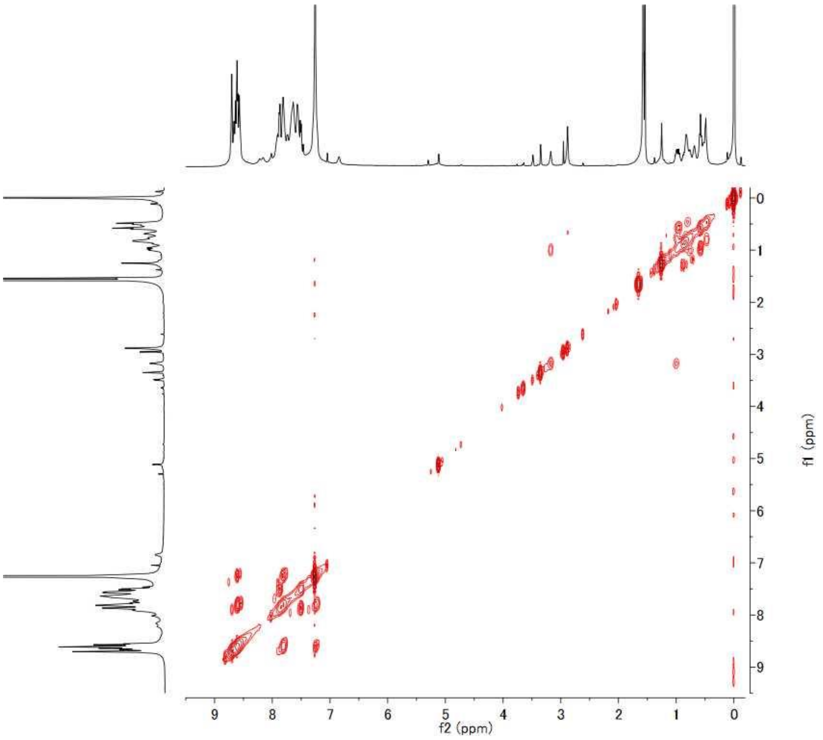

Supplementary Figure 69. 2D COSY NMR (500 MHz, $CDCl_3$, 300 K) spectrum of compound **20.**

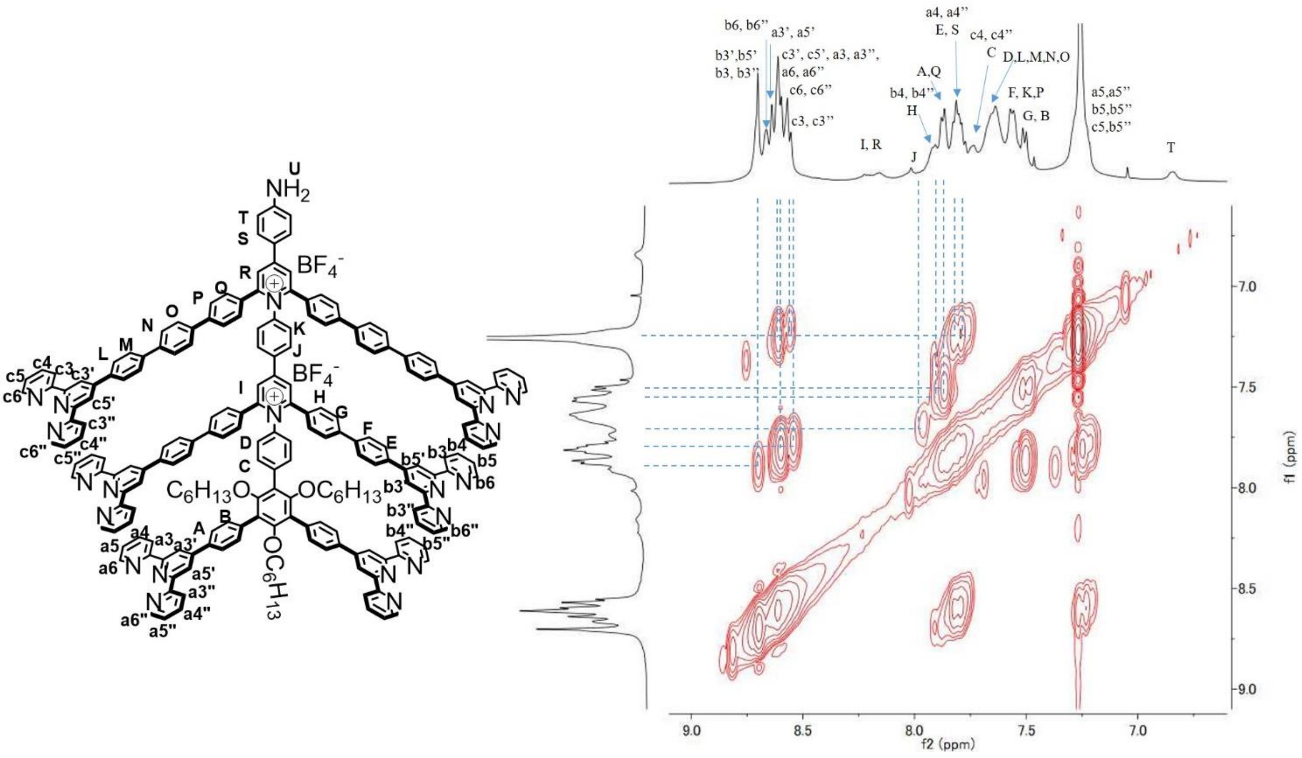

Supplementary Figure 70. 2D COSY NMR (500 MHz, $CDCl_3$, 300 K) spectrum (aromic region) of compound **20.**

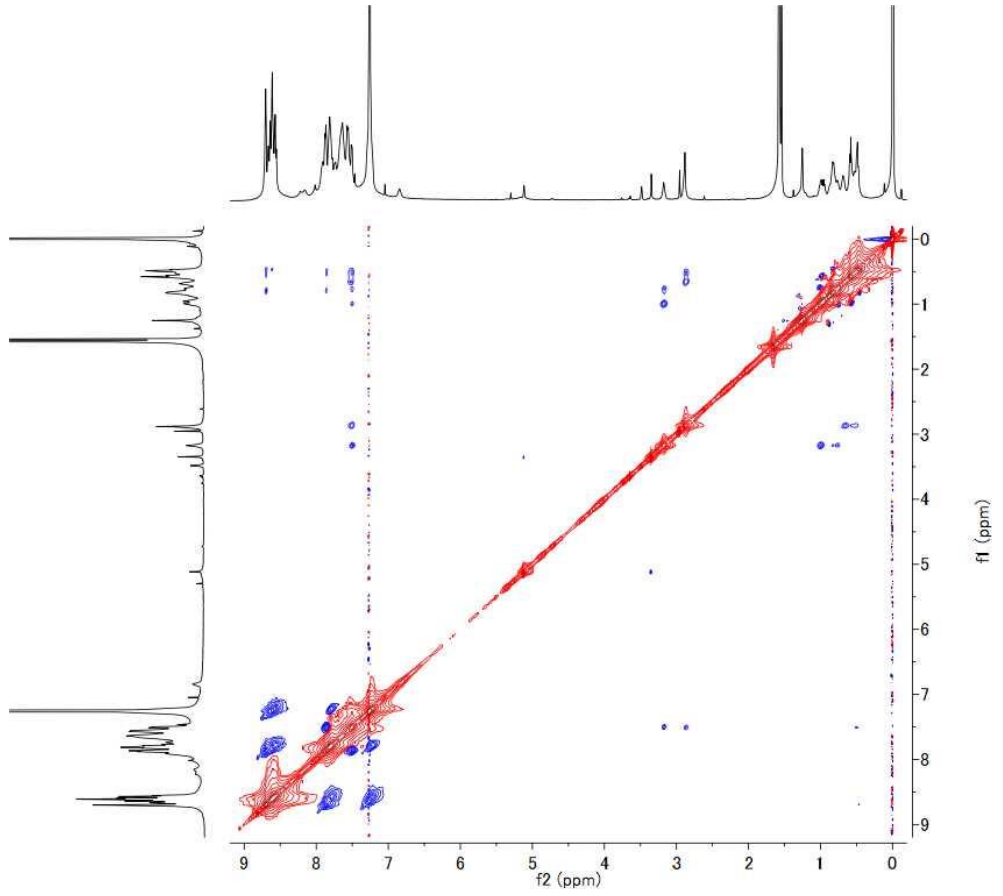

Supplementary Figure 71. 2D ROESY NMR (500 MHz, $CDCl_3$, 300 K) spectrum of compound **20.**

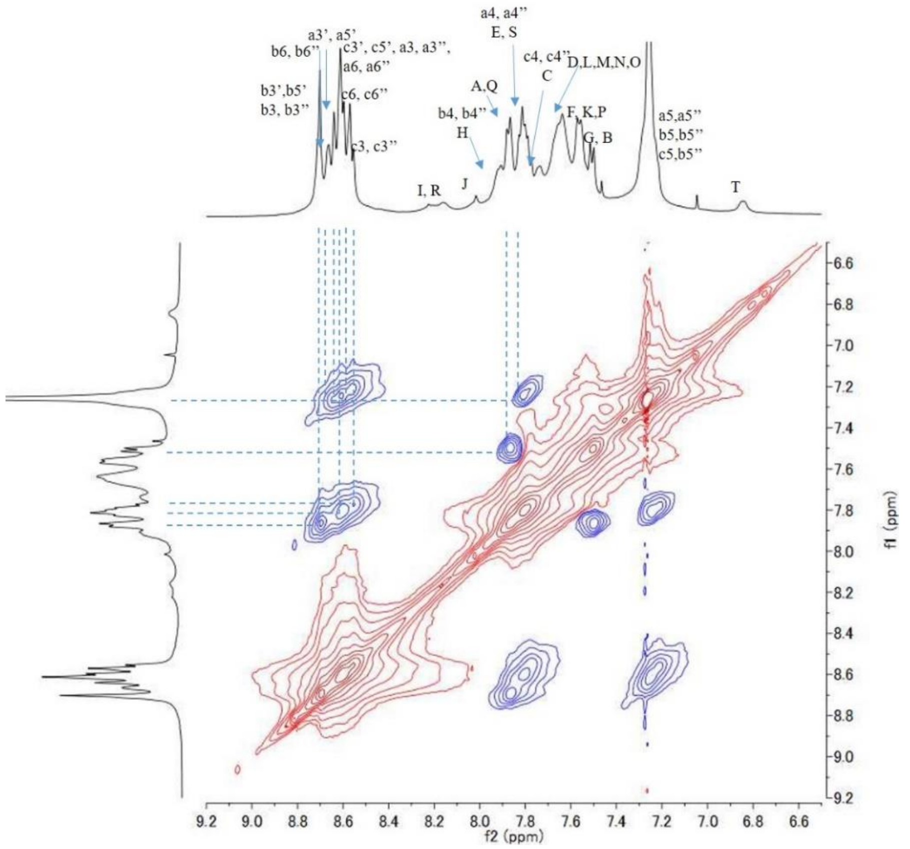

Supplementary Figure 72. 2D ROESY NMR (500 MHz, $CDCl_3$, 300 K) spectrum (aromic region) of compound **20.**

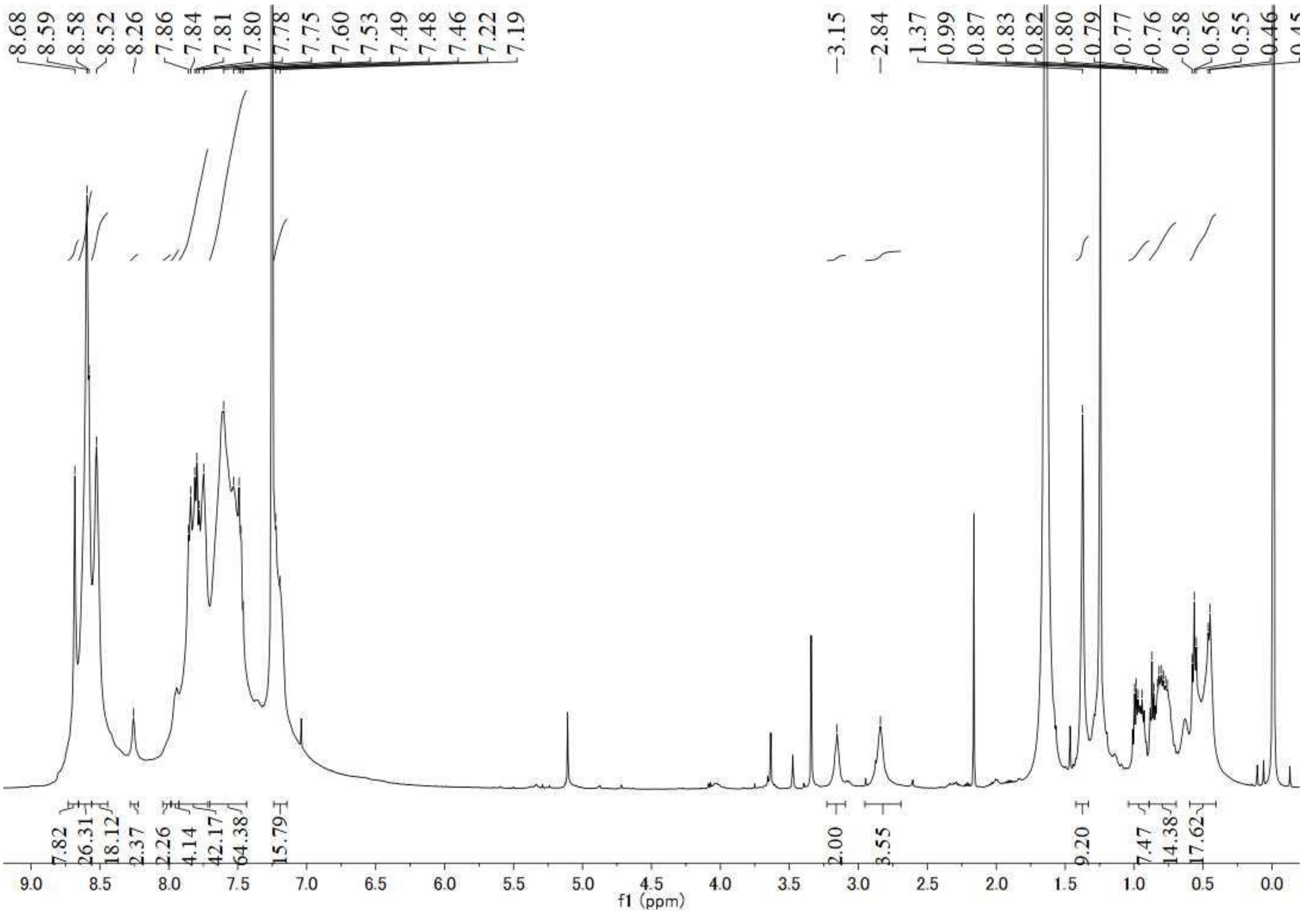

Supplementary Figure 73. ^{1}H NMR (500 MHz, $CDCl_3$, 300 K) spectrum of **L4**

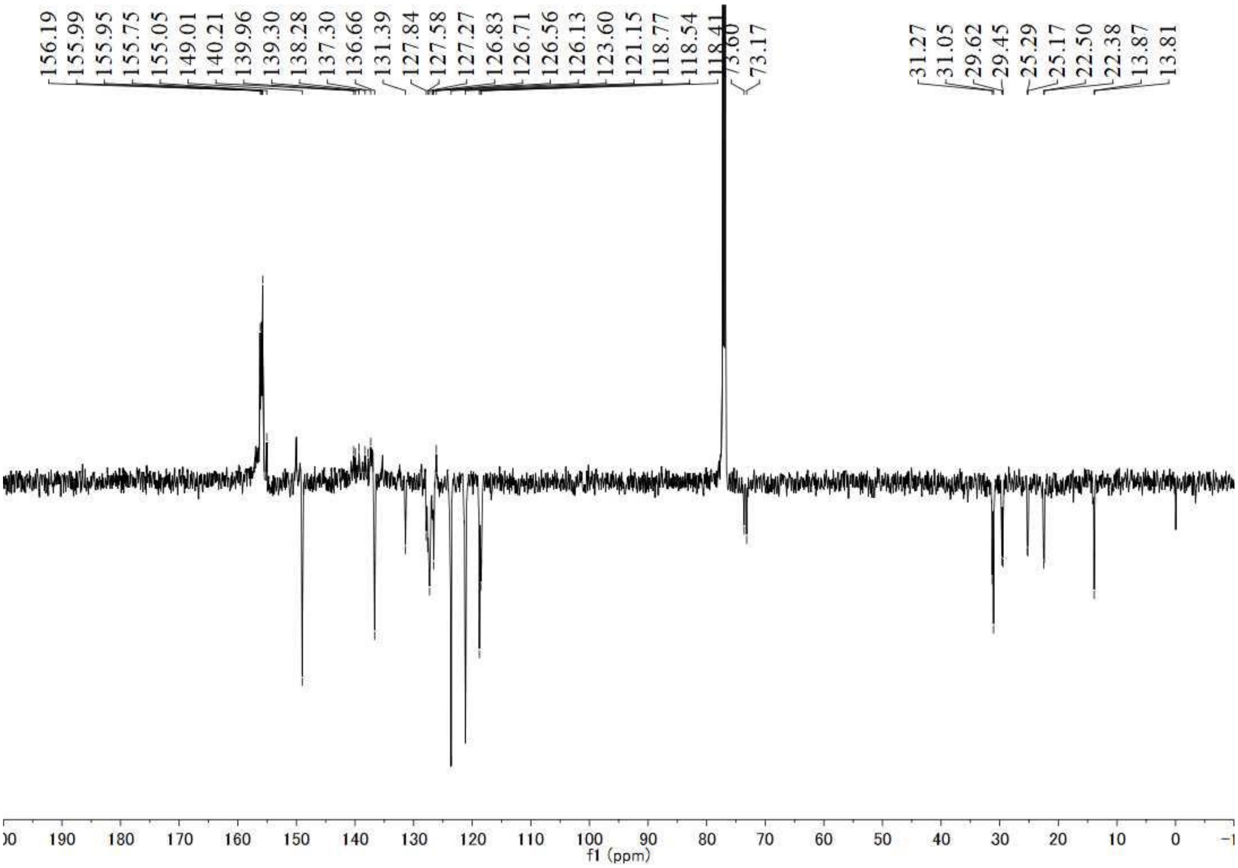

Supplementary Figure 74. ^{13}C DEPTQ NMR (150 MHz, $CDCl_3$, 300 K) spectrum of ligand **L4.**

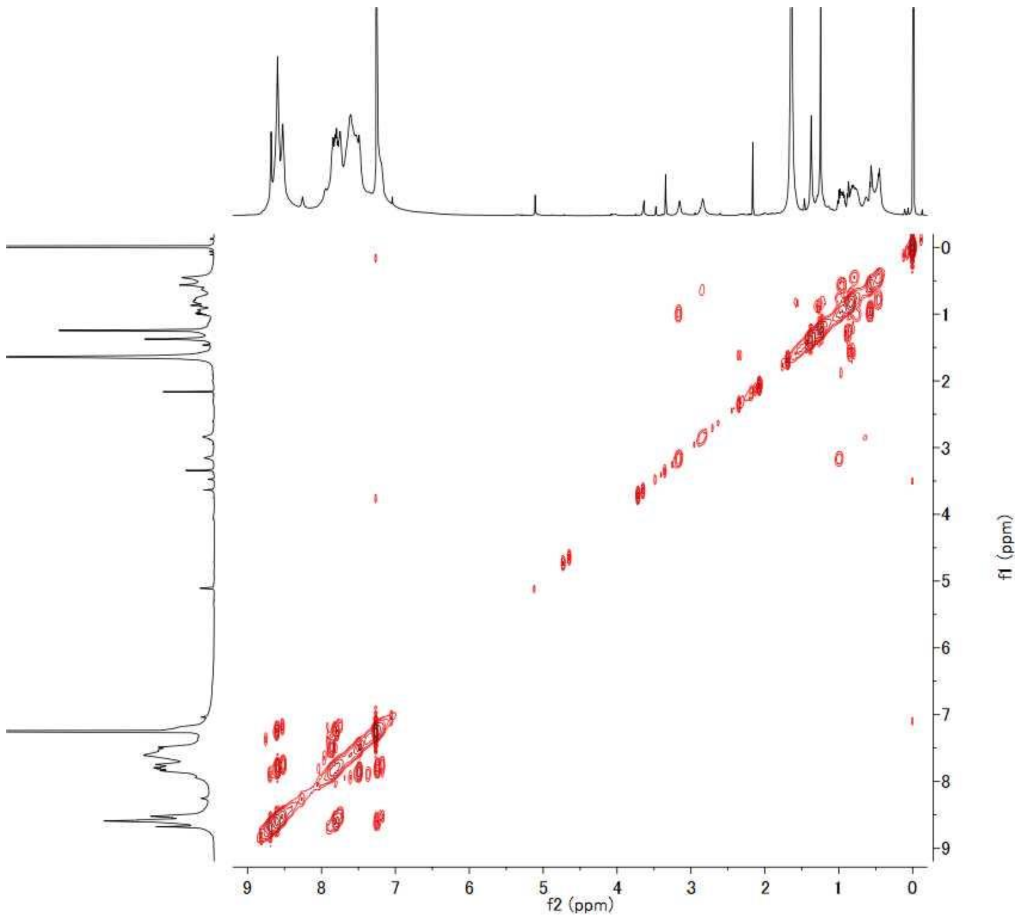

Supplementary Figure 75. 2D COSY NMR (500 MHz, $CDCl_3$, 300 K) spectrum of ligand **L4**.

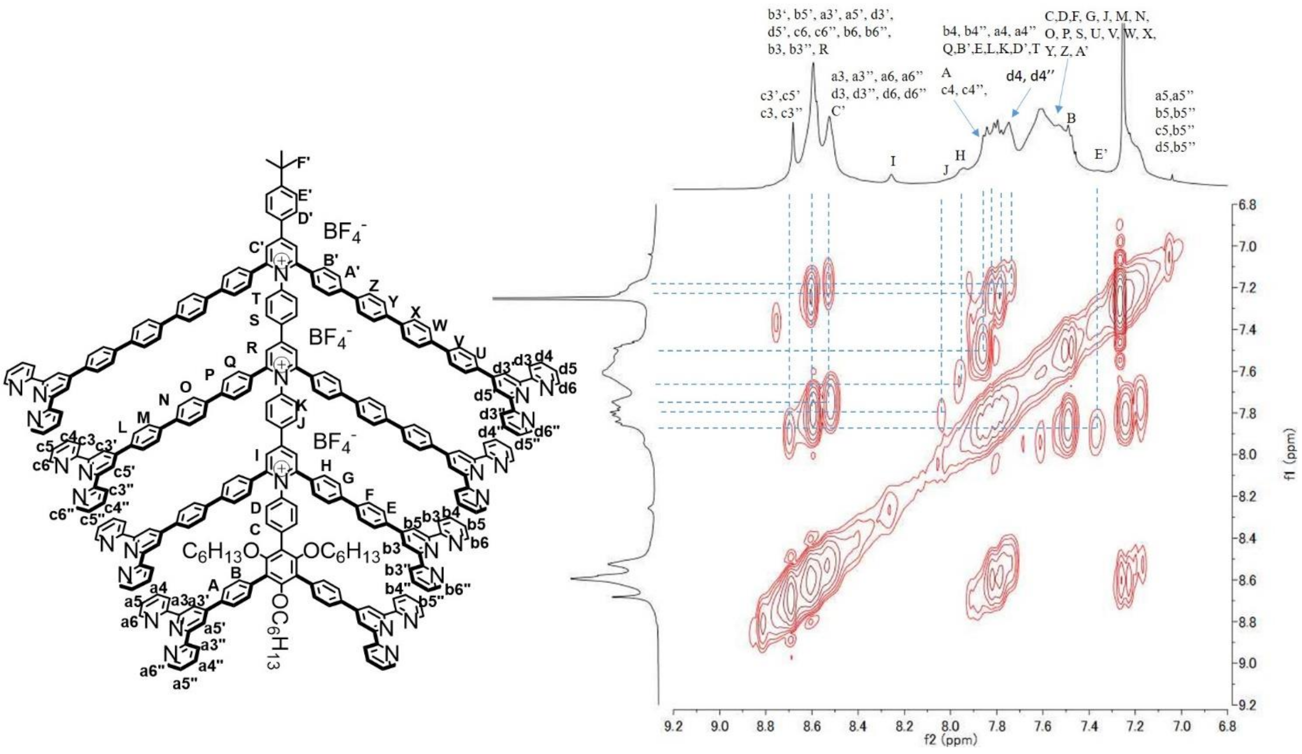

Supplementary Figure 76. 2D COSY NMR (500 MHz, $CDCl_3$, 300 K) spectrum (aromic region) of ligand **L4**.

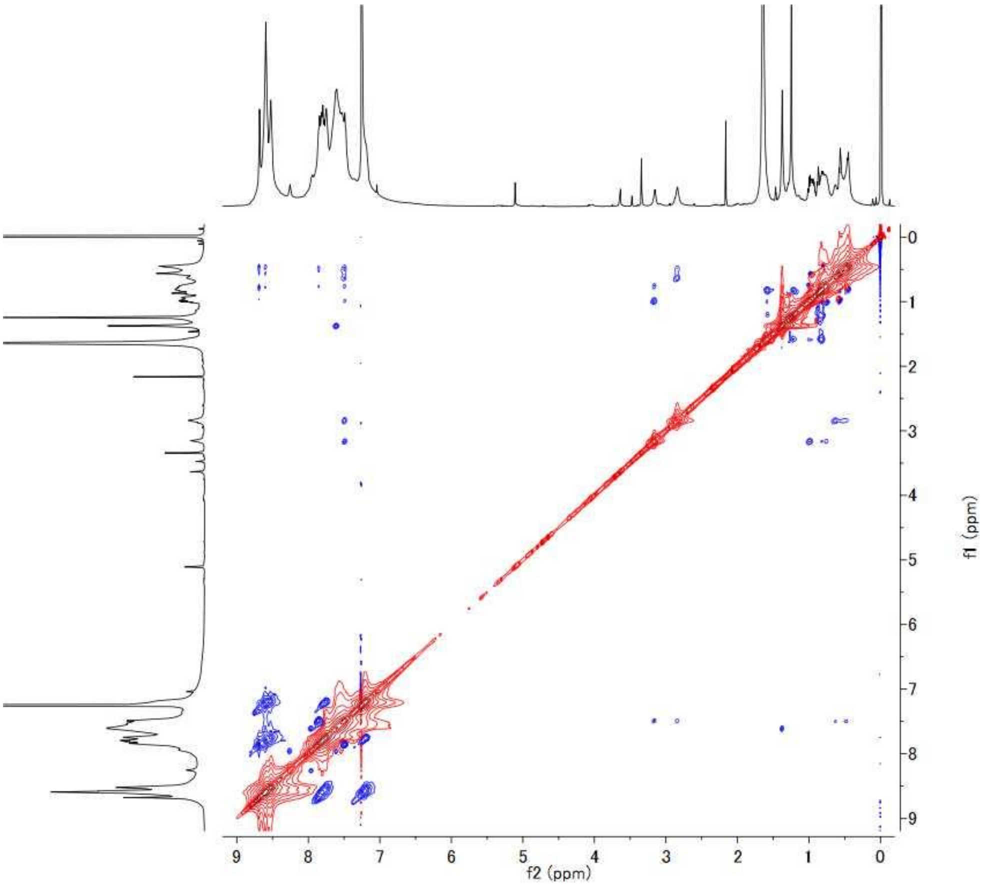

Supplementary Figure 77. 2D ROESY NMR (500 MHz, $CDCl_3$, 300 K) spectrum of ligand **L4**.

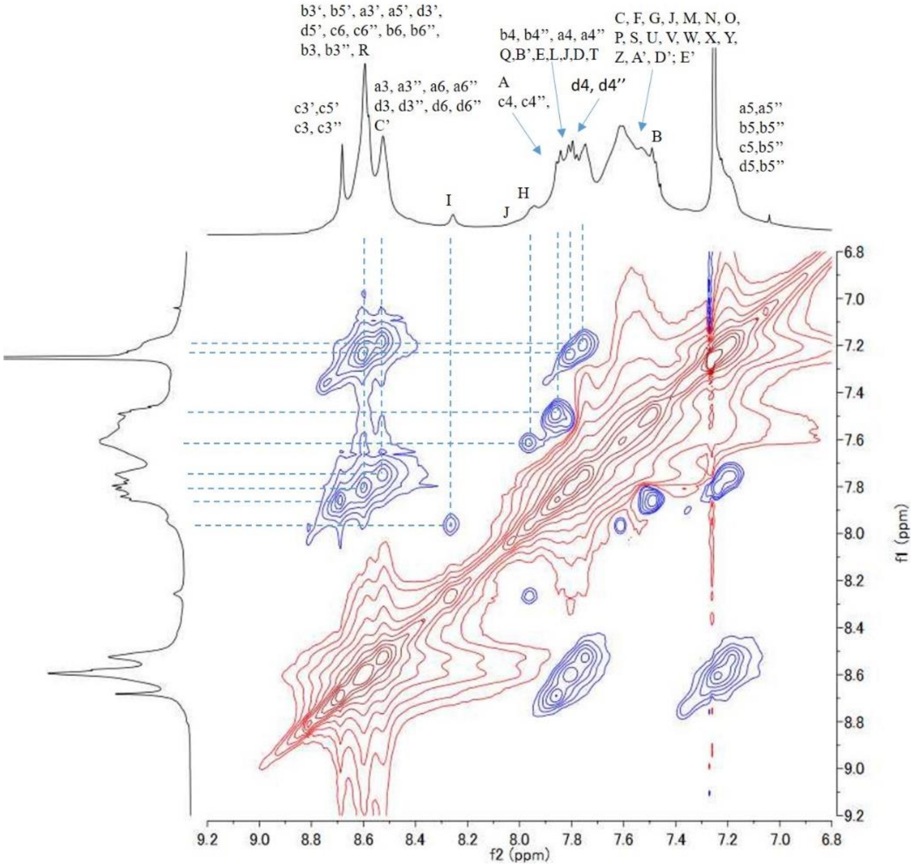

Supplementary Figure 78. 2D ROESY NMR (500 MHz, $CDCl_3$, 300 K) spectrum (aromic region) of ligand **L4**

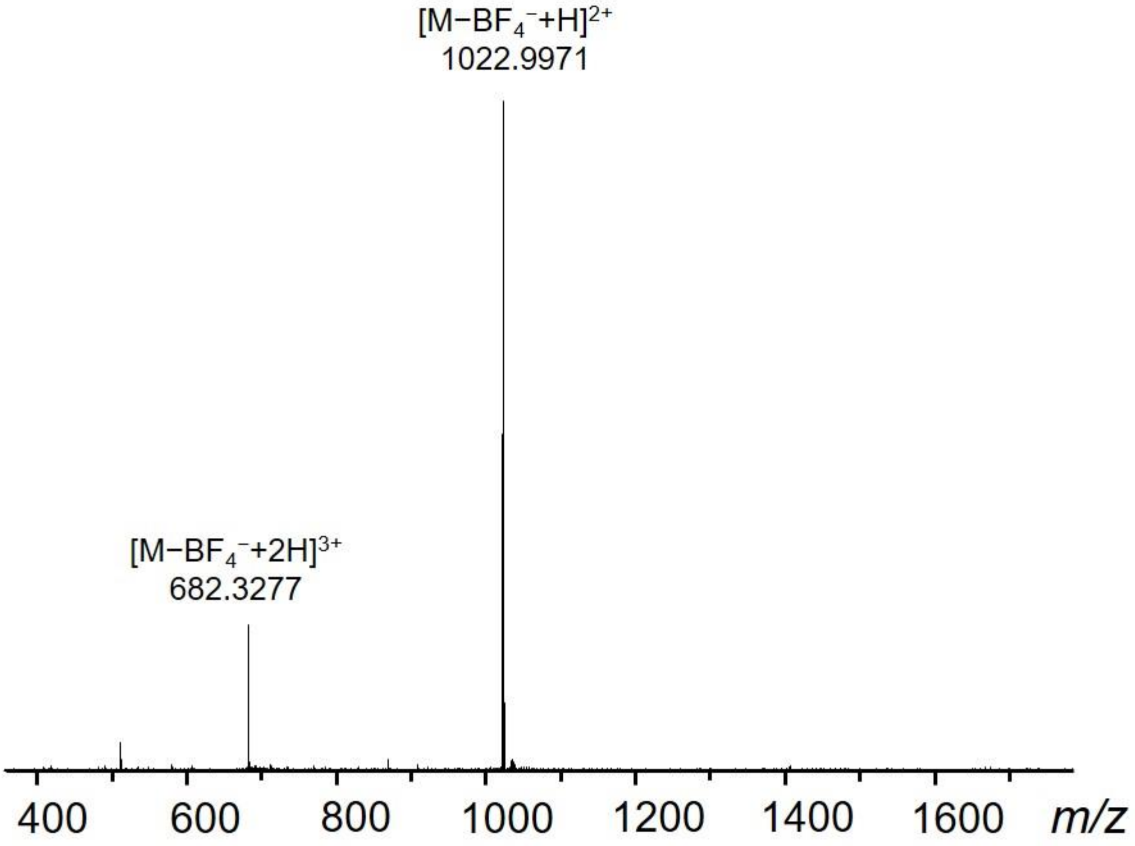

Supplementary Figure 79. HR ESI-MS spectrum of ligand **L2** in $CHCl_3$/MeOH (1/3)

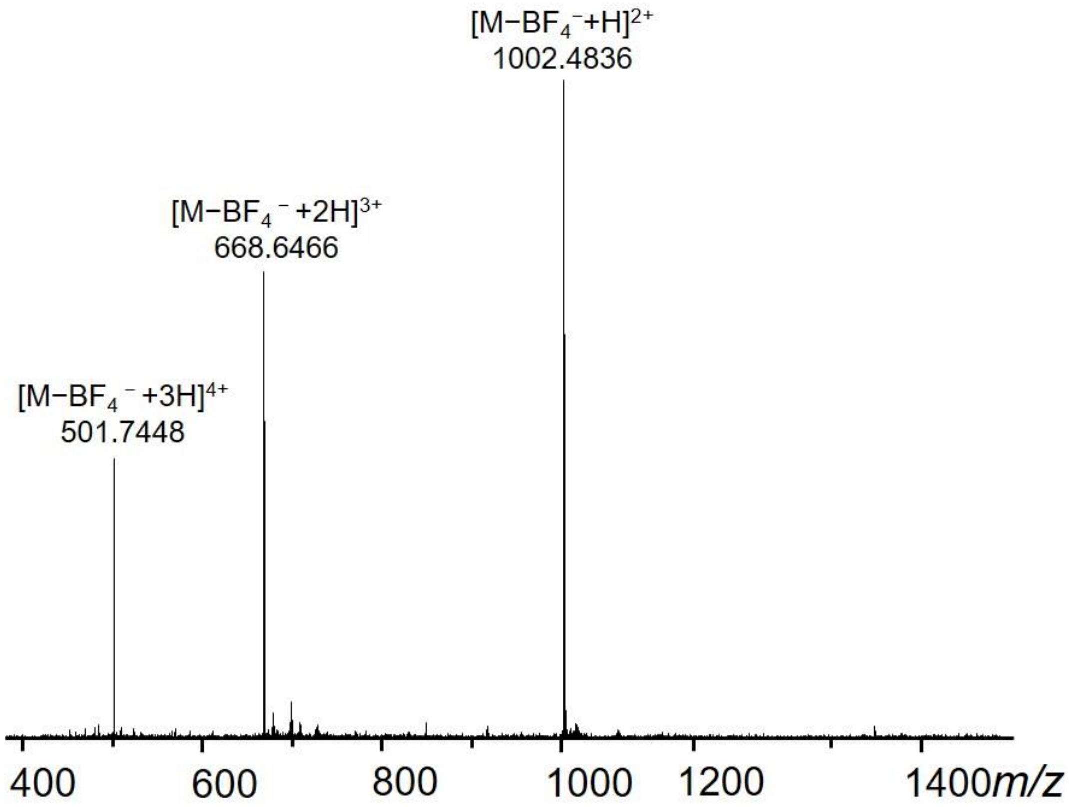

Supplementary Figure 80. HR ESI-MS spectrum of compound **13** in $CHCl_3$/MeOH (1/3)

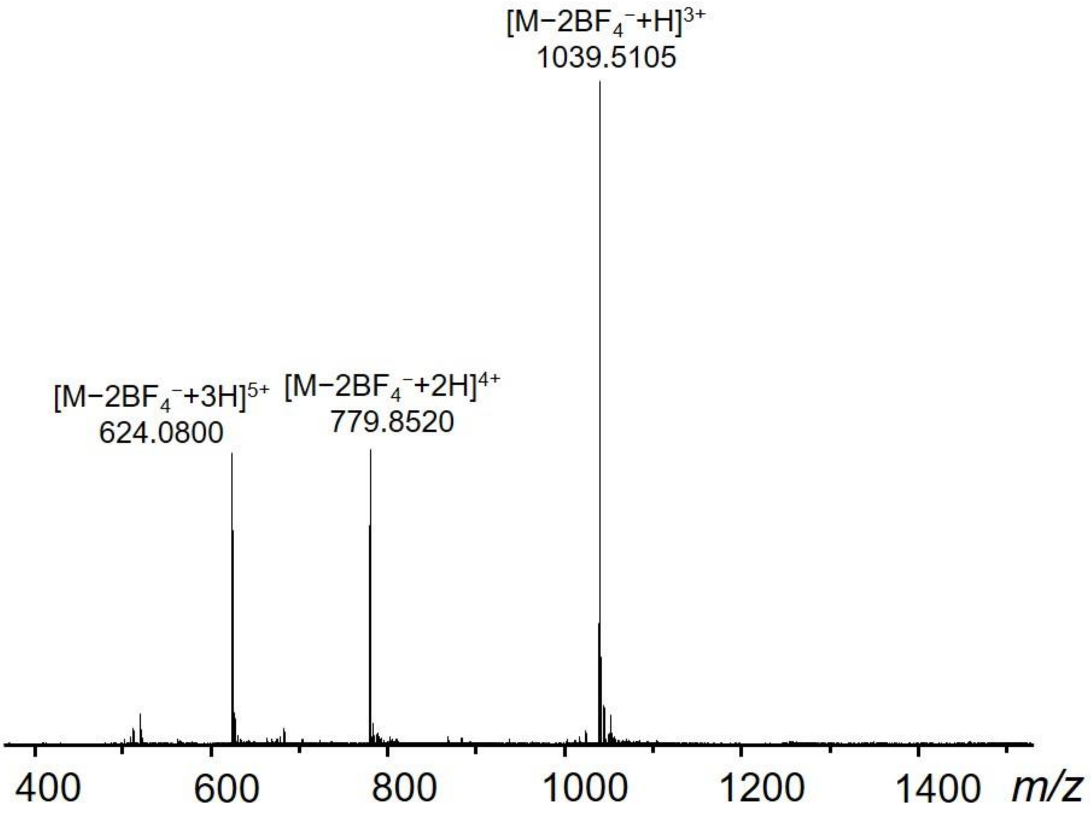

Supplementary Figure 81. HR ESI-MS spectrum of ligand **L3** in $CHCl_3$/MeOH (1/3)

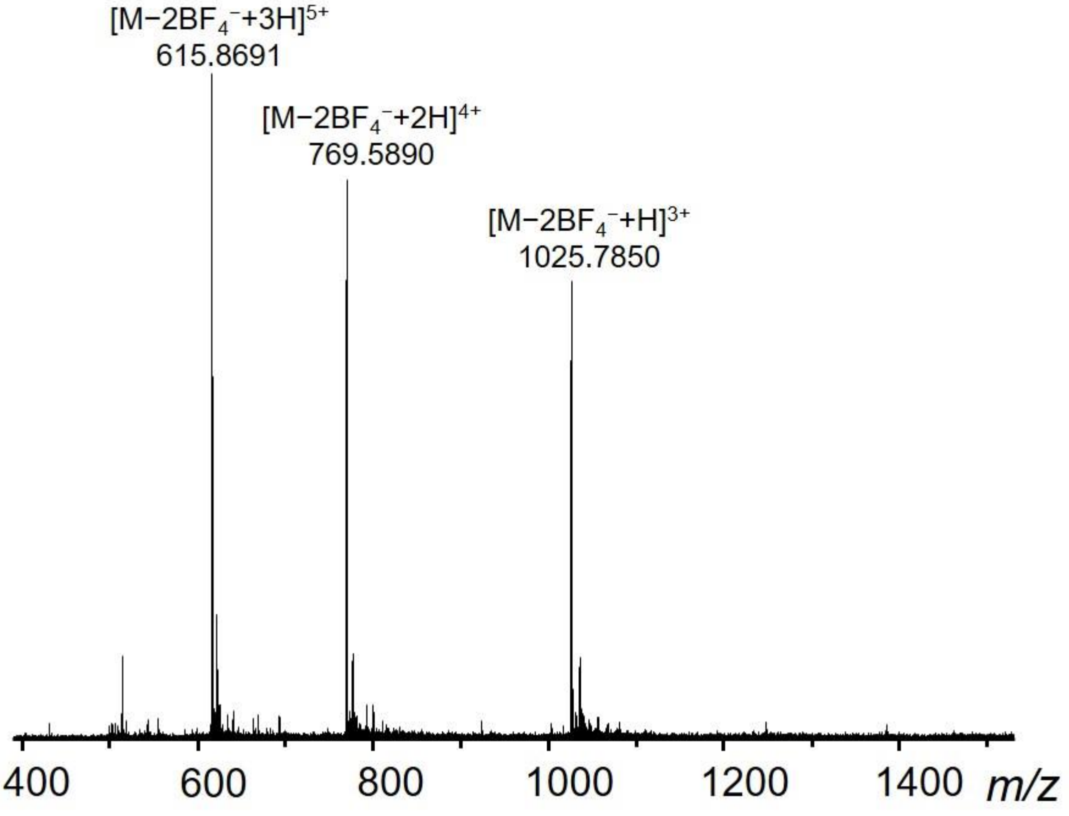

Supplementary Figure 82. HR ESI-MS spectrum of compound **20** in $CHCl_3$/MeOH (1/3)

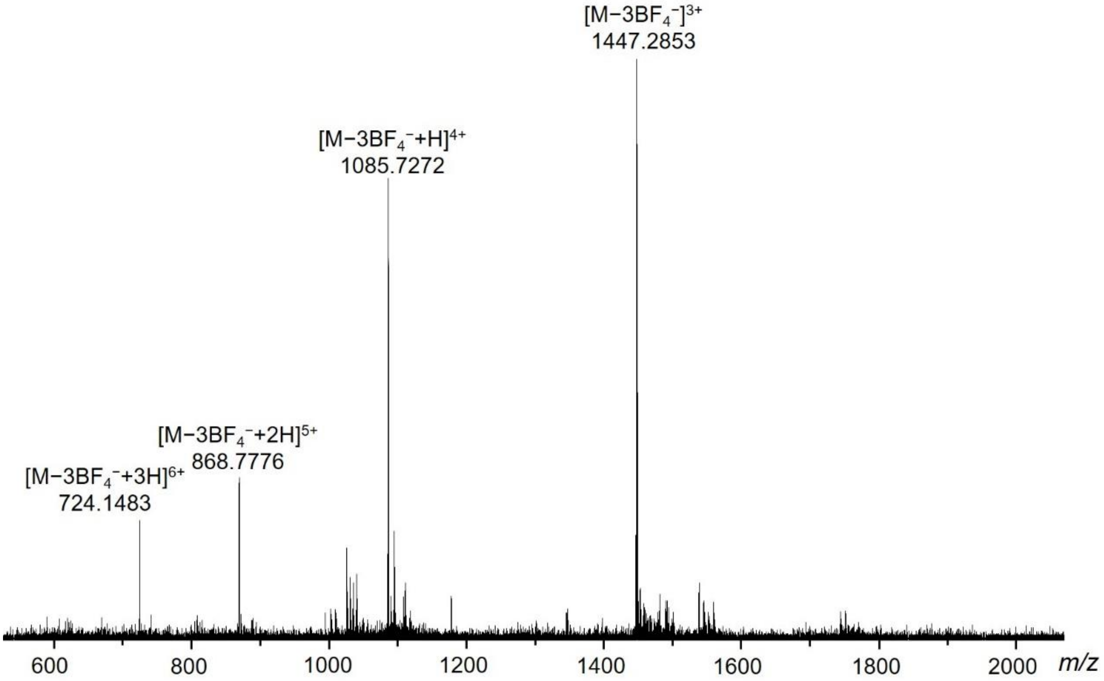

Supplementary Figure 83. HR ESI-MS spectrum of ligand **L4** in anhydrous CH_3CN

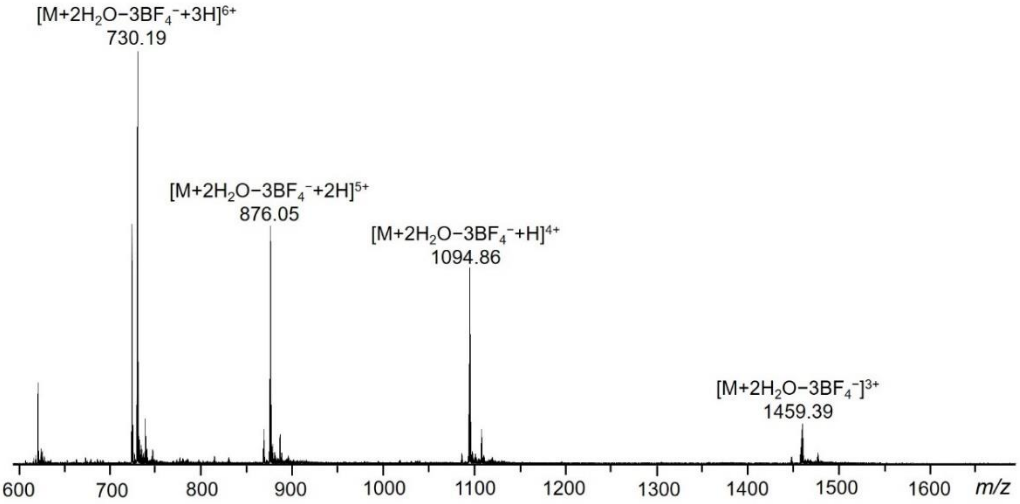

Supplementary Figure 84. ESI-MS spectrum of ligand **L4** in $CHCl_3$/MeOH (1/3)

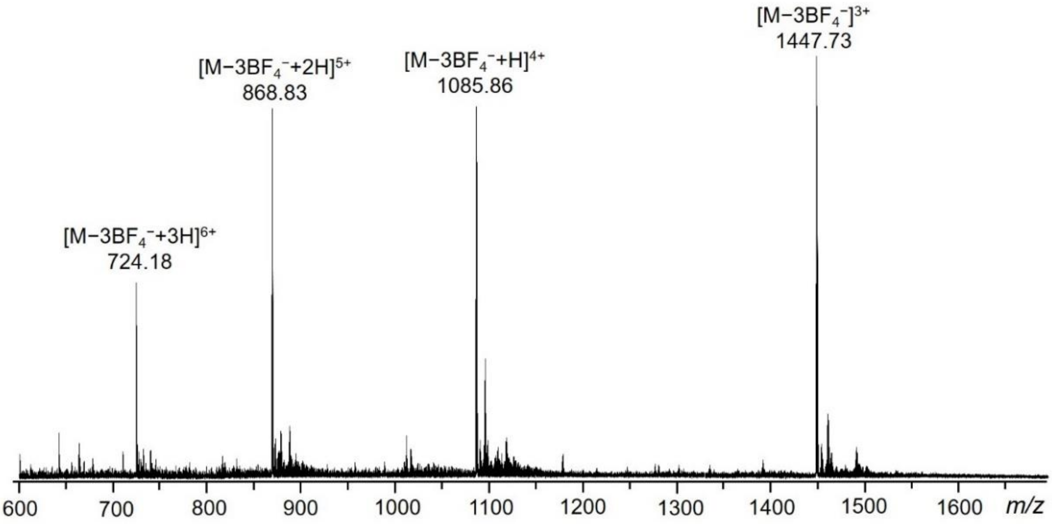

Supplementary Figure 85. ESI-MS spectrum of ligand **L4** in anhydrous CH_3CN

5. ^{1}H NMR, ^{13}C NMR, 2D COSY NMR, 2D NOESY NMR, 2D DOSY NMR and ESI-MS spectra of complexes G1−G4

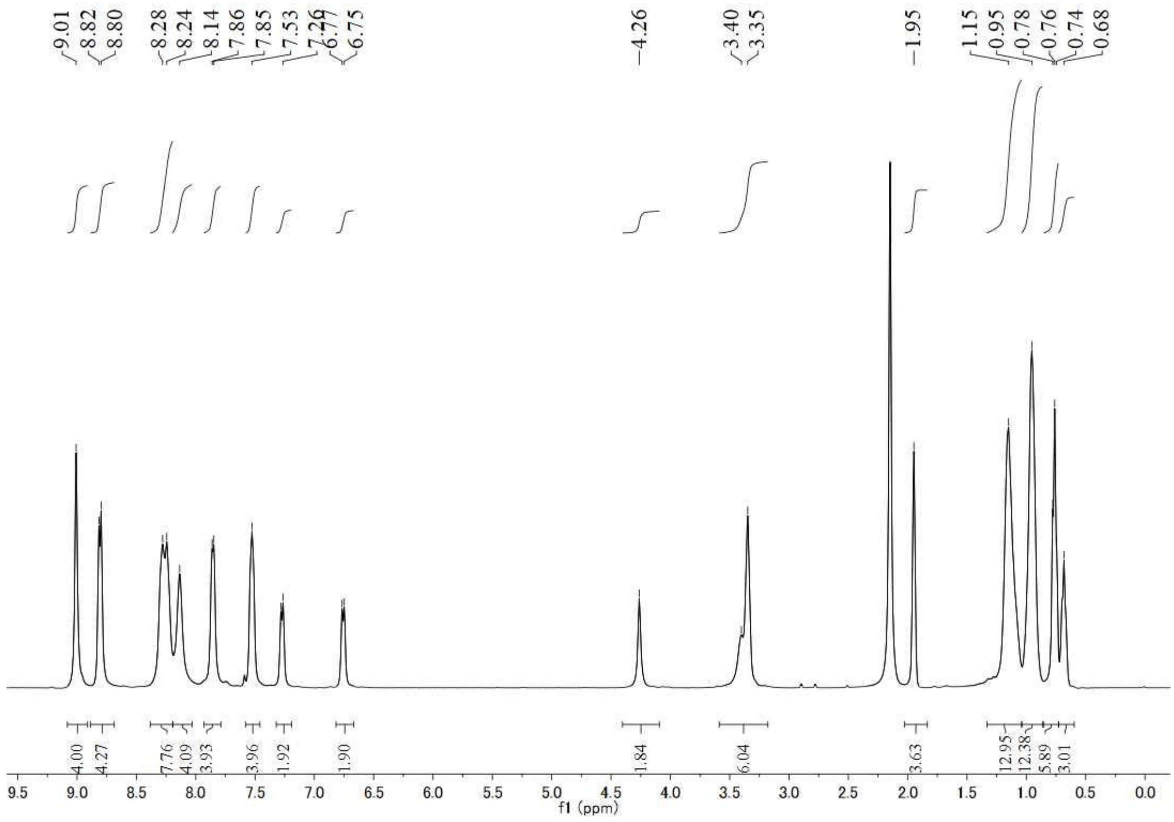

Supplementary Figure 86. ^{1}H NMR (400 MHz, CD_3CN, 300 K) spectrum of complex **G1.**

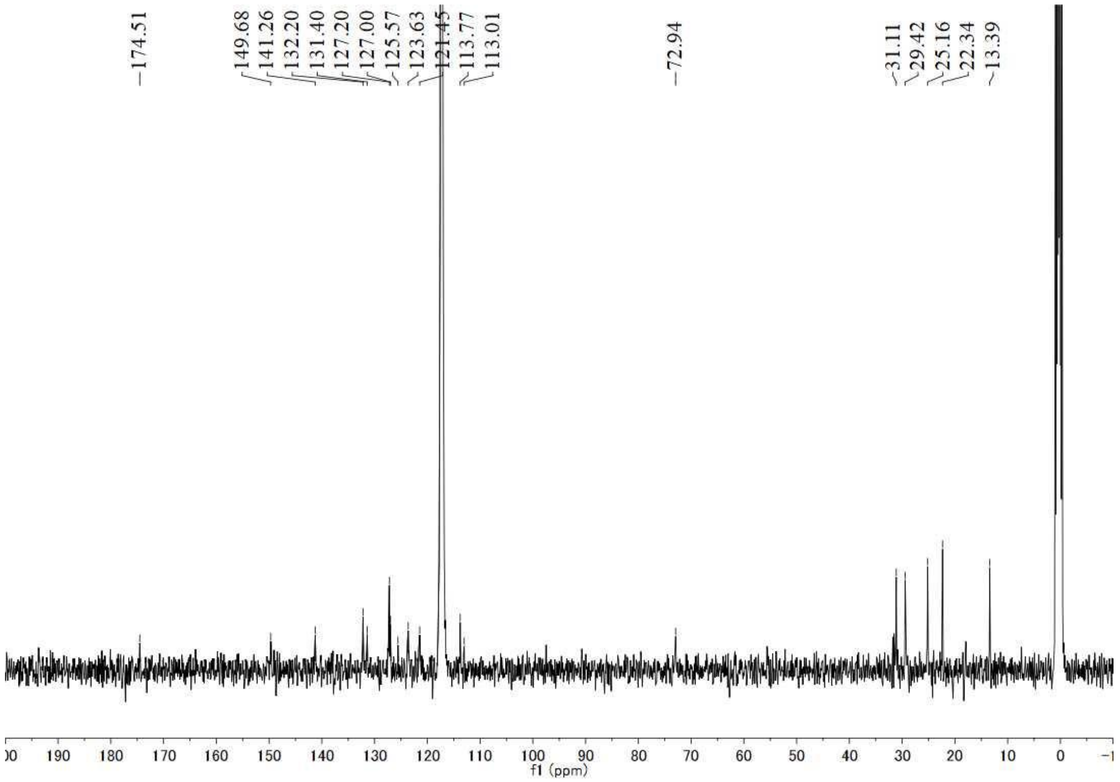

Supplementary Figure 87. ^{13}C NMR (100 MHz, CD_3CN, 300 K) spectrum of complex **G1.**

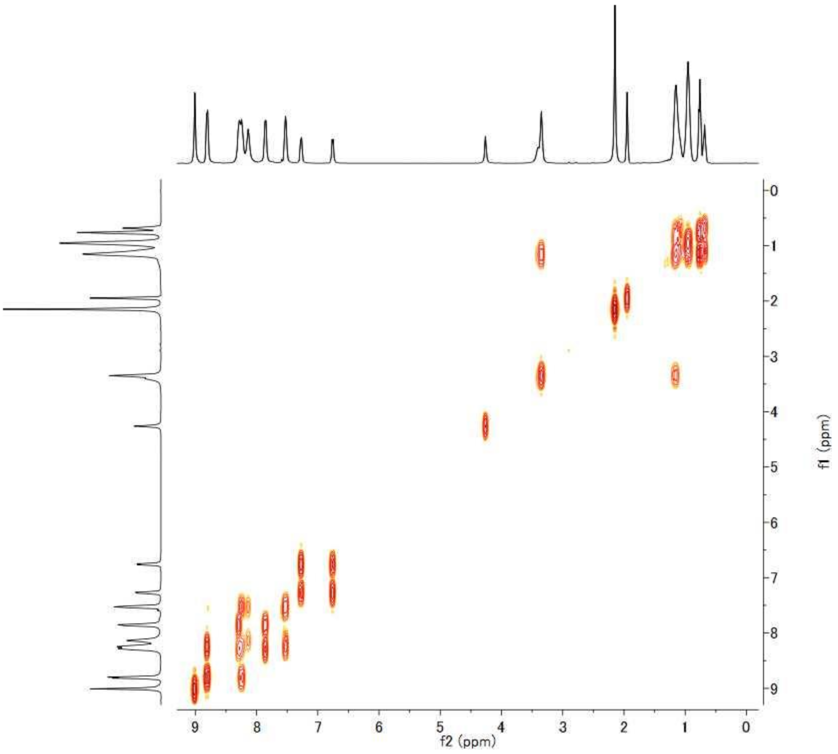

Supplementary Figure 88. 2D COSY NMR (400 MHz, CD_3CN, 300 K) spectrum of complex **G1.**

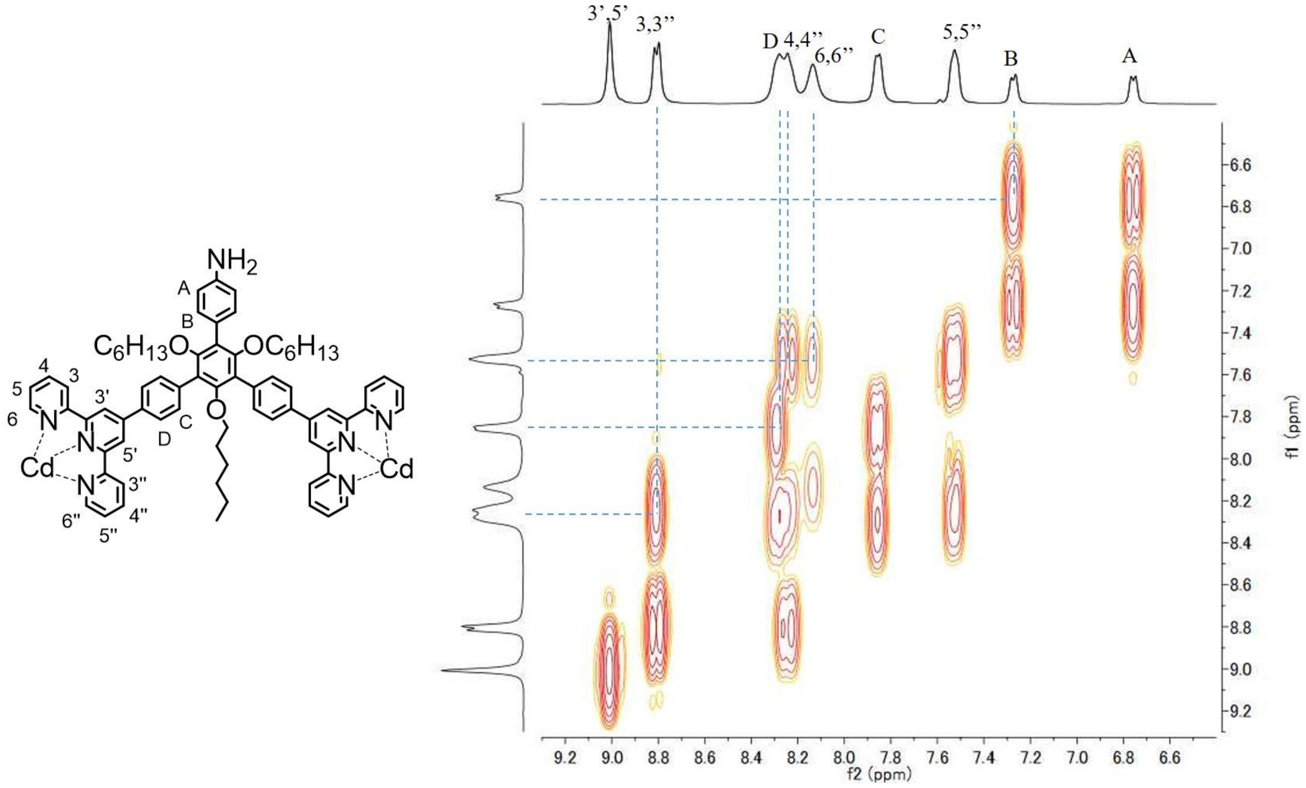

Supplementary Figure 89. 2D COSY NMR (400 MHz, CD_3CN, 300 K) spectrum (aromic region) of complex **G1.**

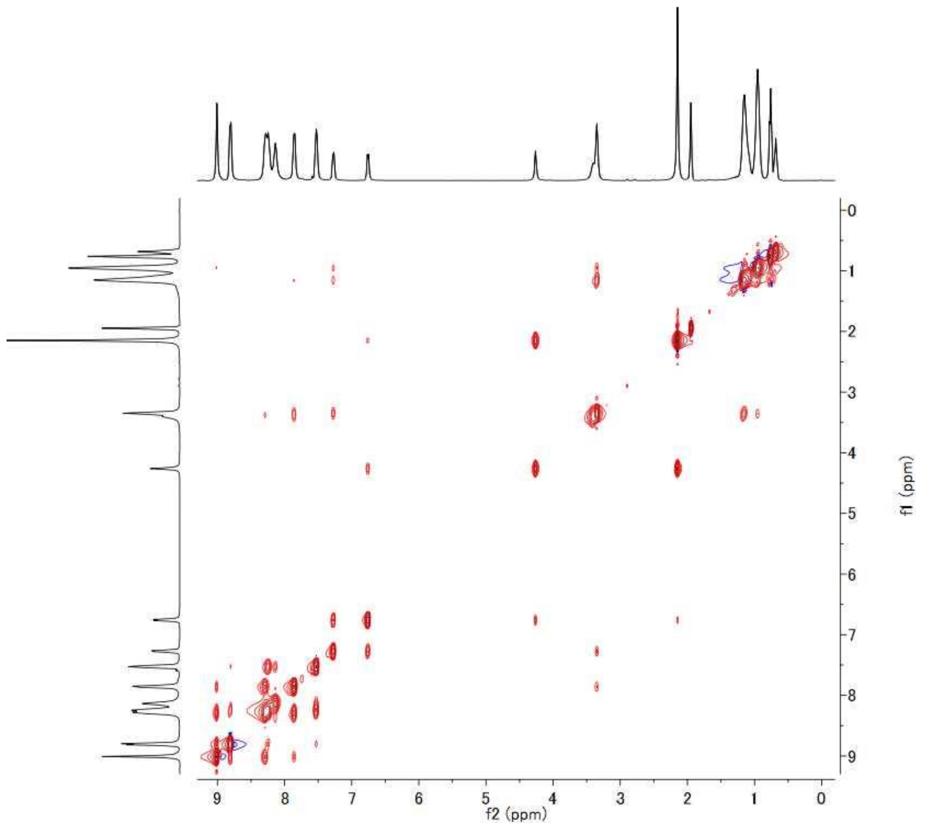

Supplementary Figure 90. 2D NOESY NMR (400 MHz, CD_3CN, 300 K) spectrum of complex **G1.**

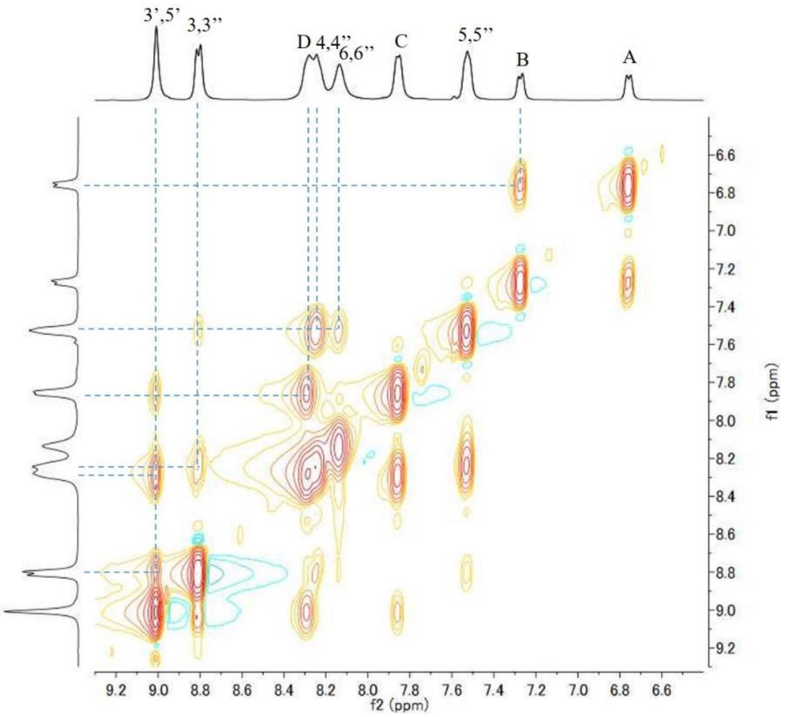

Supplementary Figure 91. 2D NOESY NMR (400 MHz, CD_3CN, 300 K) spectrum (aromic region) of complex **G1.**

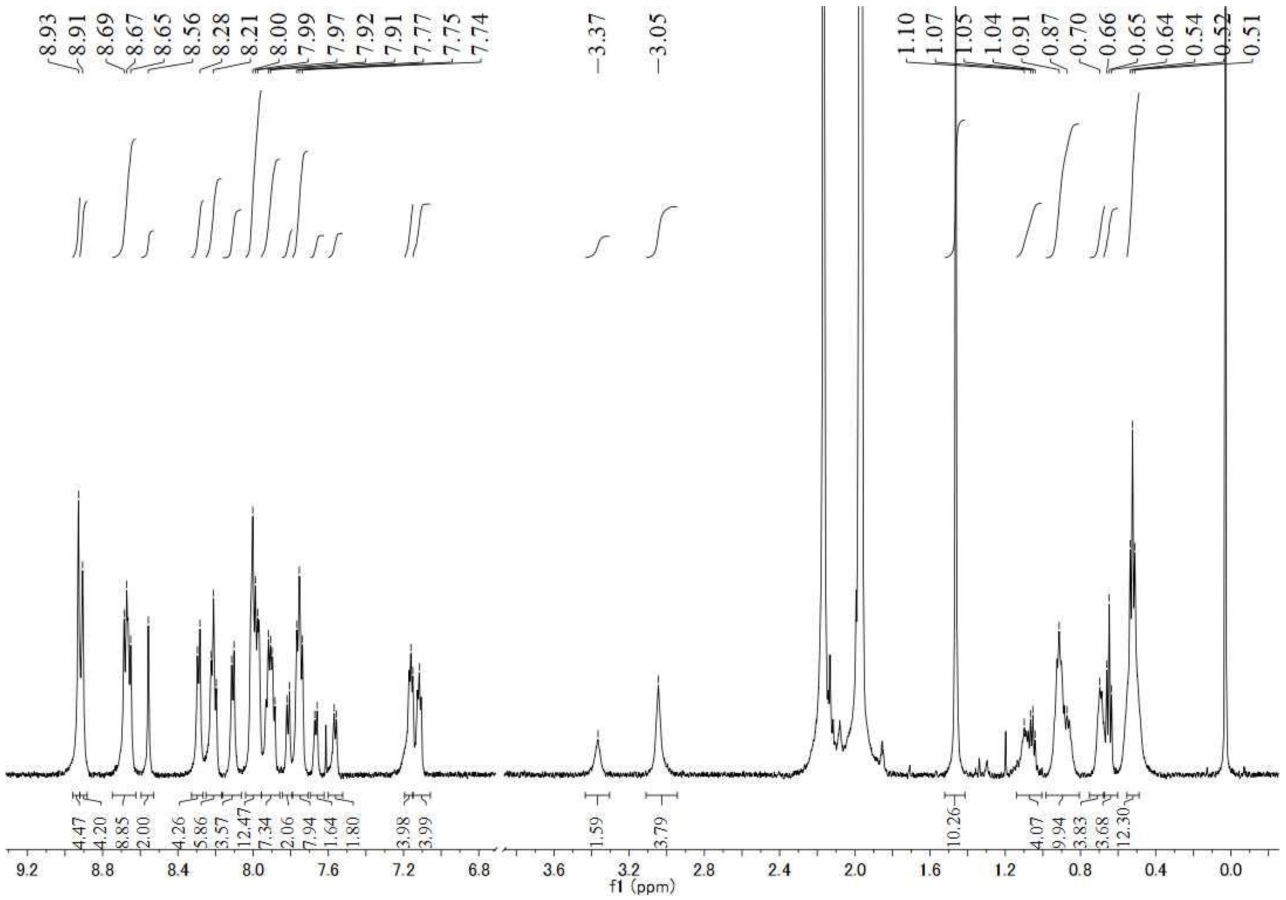

Supplementary Figure 92. ^{1}H NMR (600 MHz, CD_3CN, 300 K) spectrum of complex **G2.**

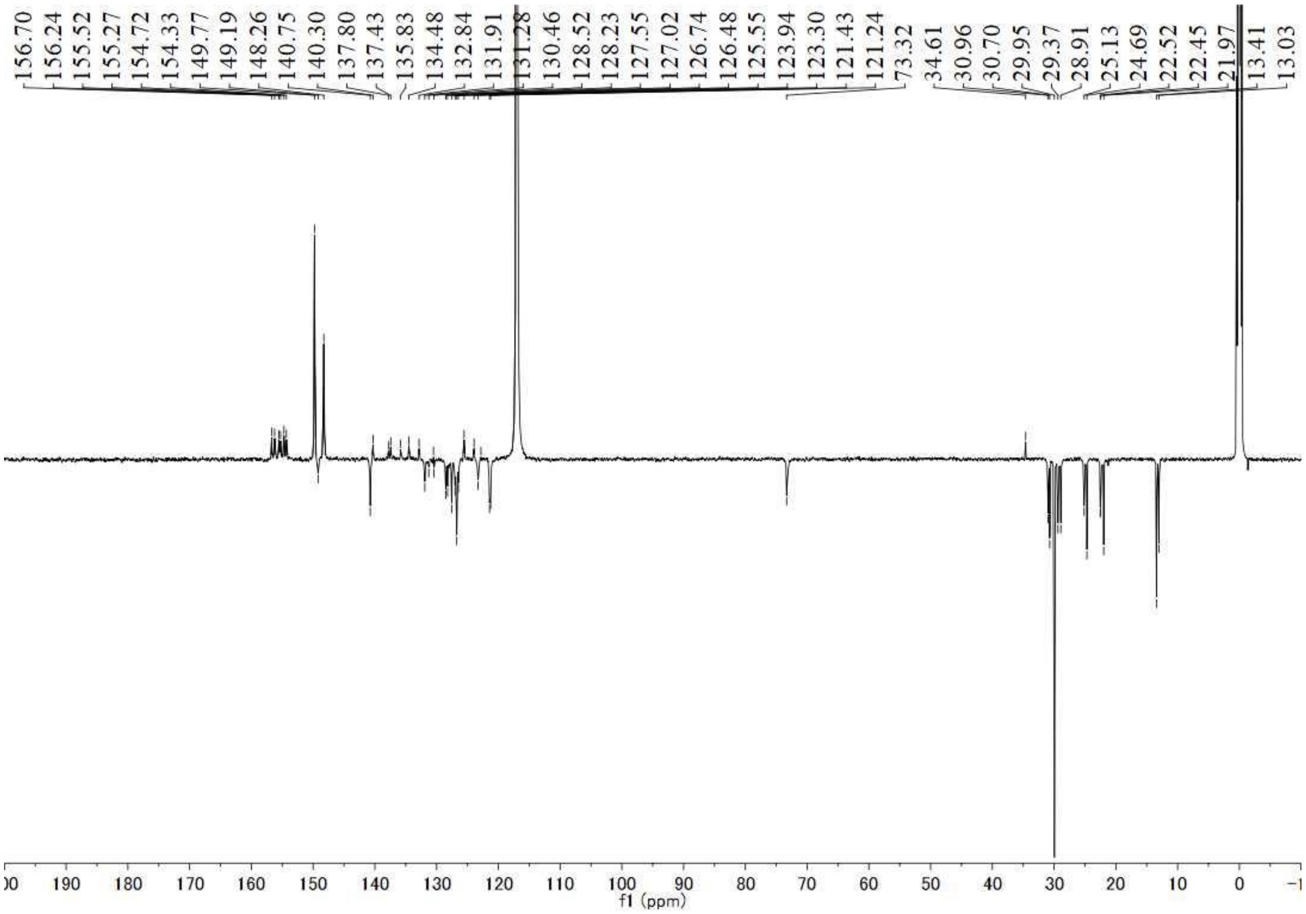

Supplementary Figure 93. ^{13}C DEPTQ NMR (600 MHz, CD_3CN, 300 K) spectrum of complex **G2.**

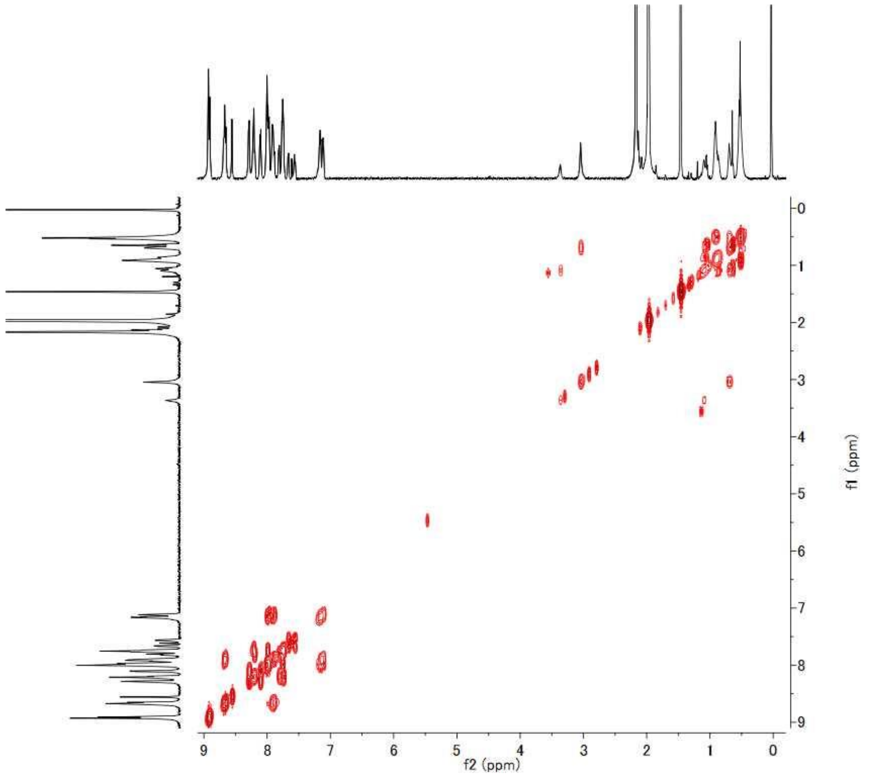

Supplementary Figure 94. 2D COSY NMR (600 MHz, CD_3CN, 300 K) spectrum of complex **G2.**

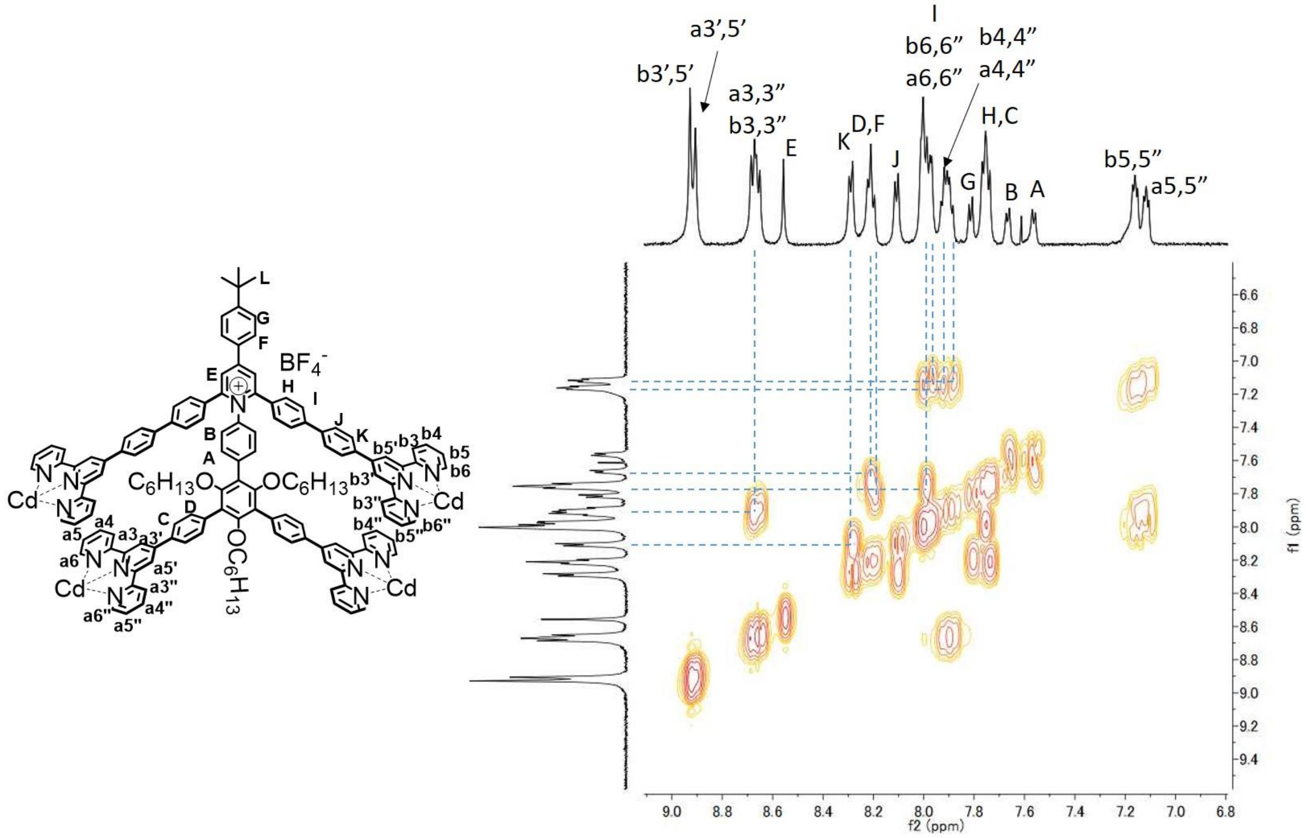

Supplementary Figure 95. 2D COSY NMR (600 MHz, CD_3CN, 300 K) spectrum (aromic region) of complex **G2.**

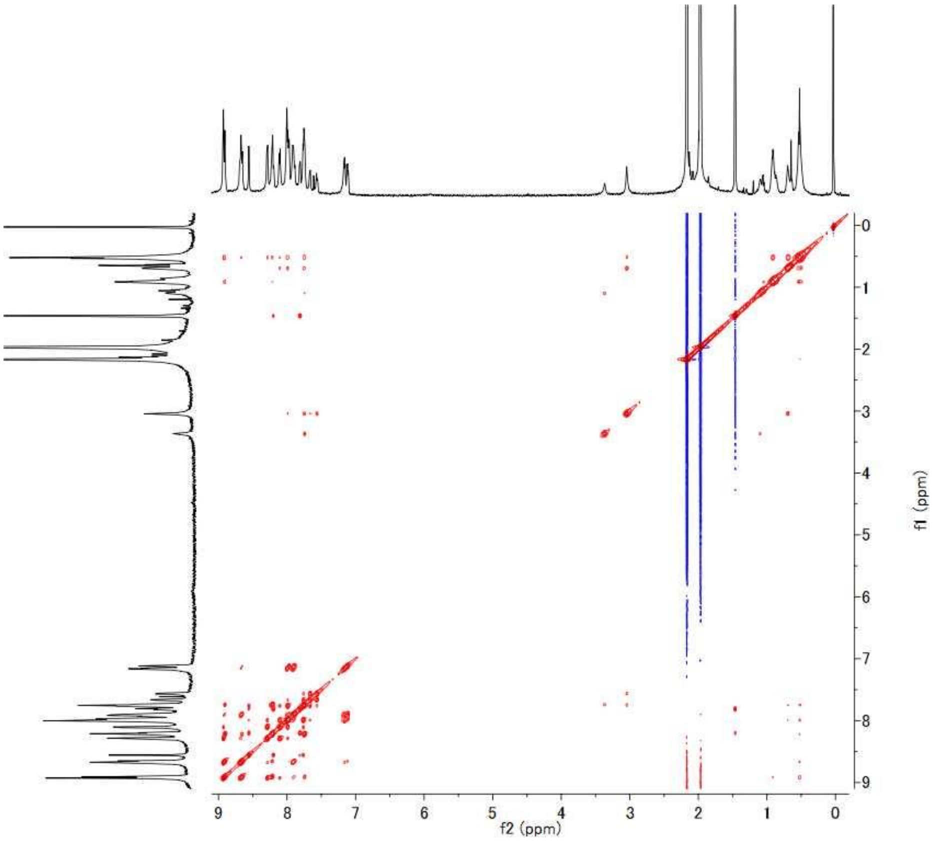

Supplementary Figure 96. 2D NOESY NMR (600 MHz, CD_3CN, 300 K) spectrum of complex **G2.**

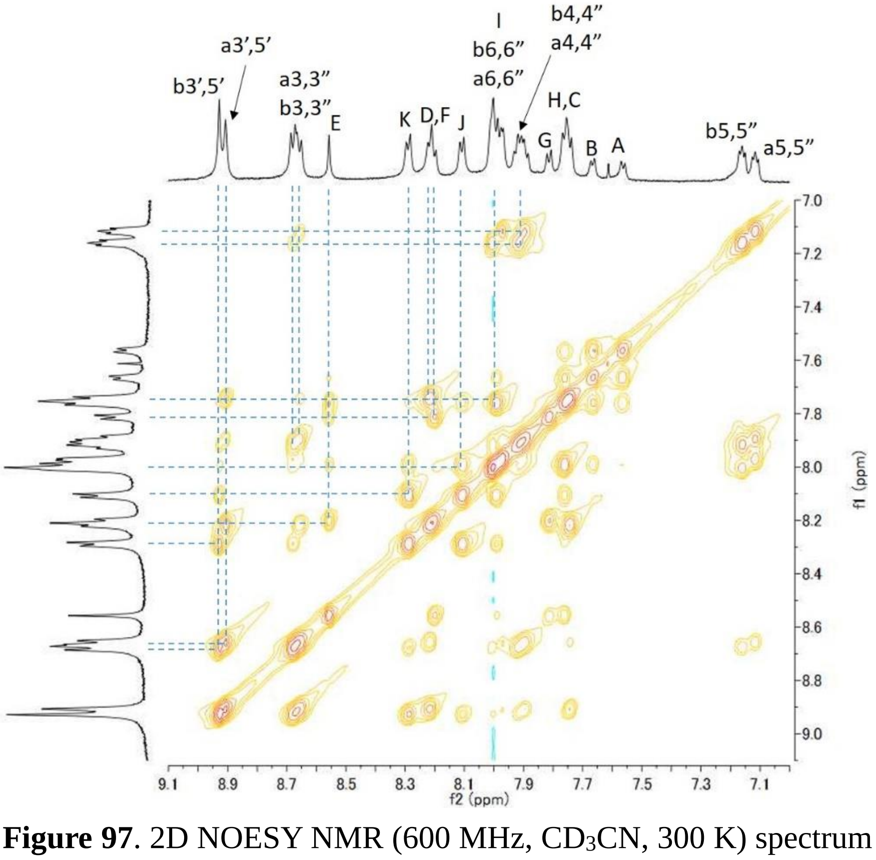

Supplementary Figure 97. 2D NOESY NMR (600 MHz, CD_3CN, 300 K) spectrum (aromic region) of complex **G2.**

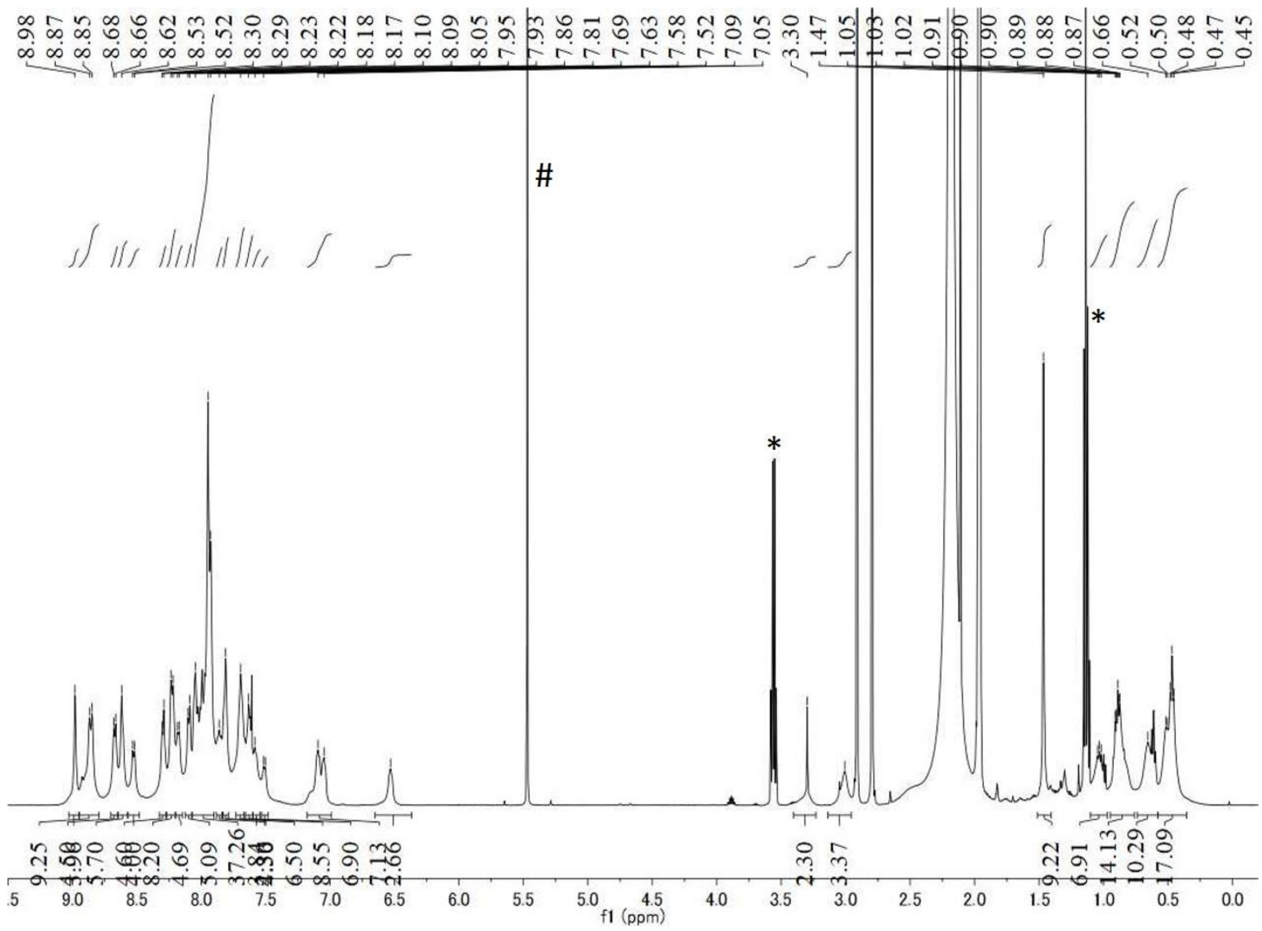

Supplementary Figure 98. ^{1}H NMR (500 MHz, CD_3CN, 300 K) spectrum of complex **G3**, # and * refer to the peak of DCM and ethanol**.**

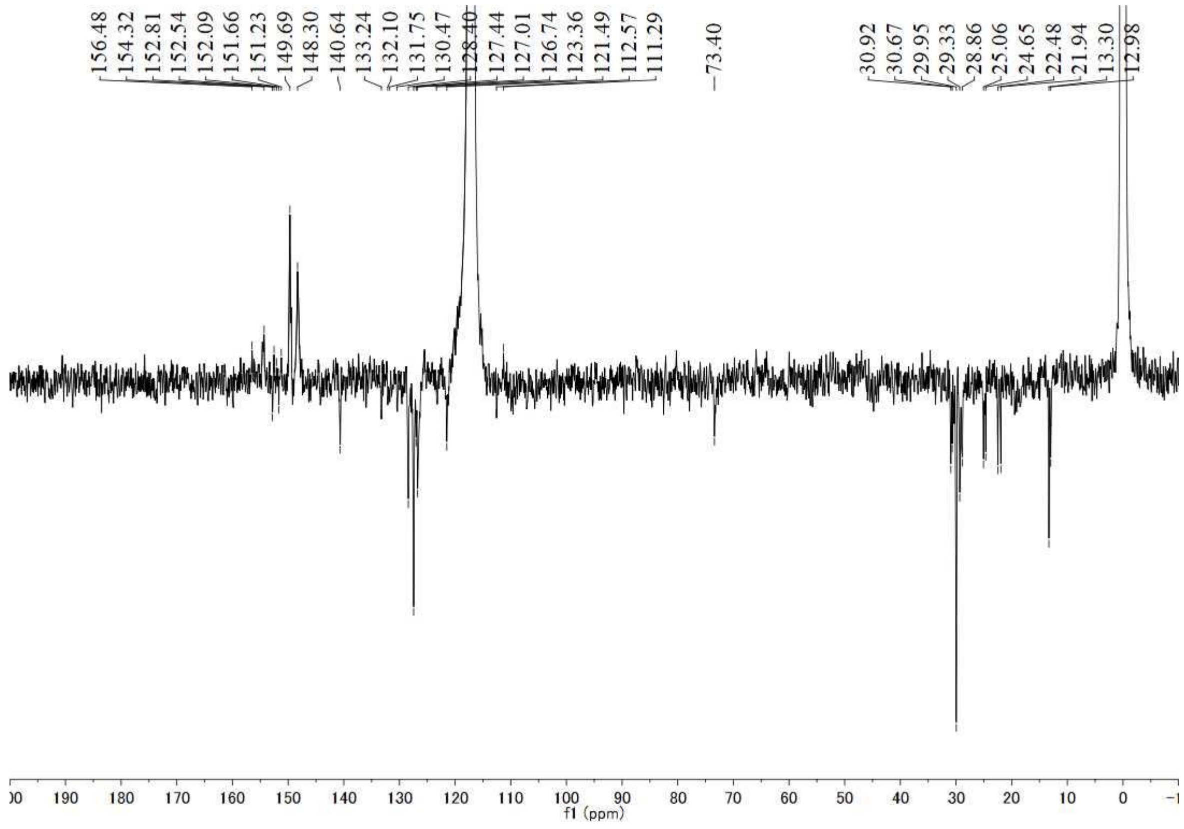

Supplementary Figure 99. ^{13}CDEPTQ NMR (125 MHz, CD_3CN, 300 K) spectrum of complex **G3.**

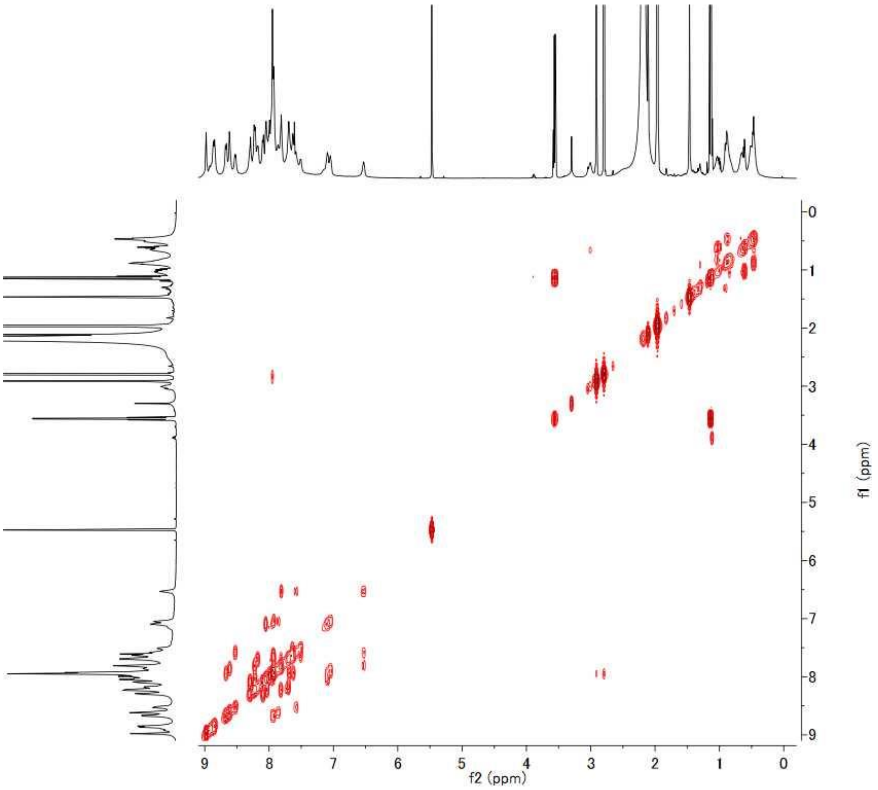

Supplementary Figure 100. 2D COSY NMR (500 MHz, CD_3CN, 300 K) spectrum of complex **G3.**

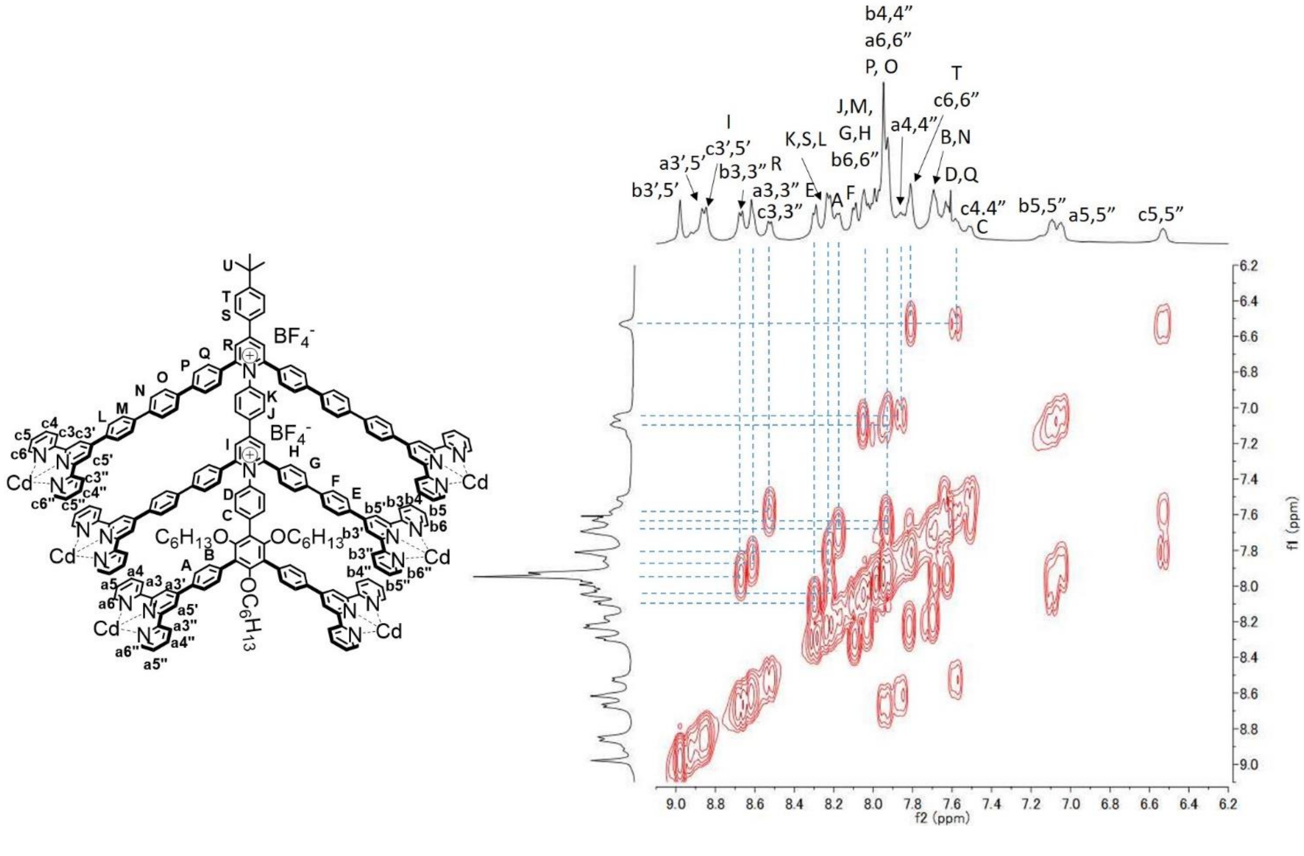

Supplementary Figure 101. 2D COSY NMR (500 MHz, CD_3CN, 300 K) spectrum (aromic region) of complex **G3.**

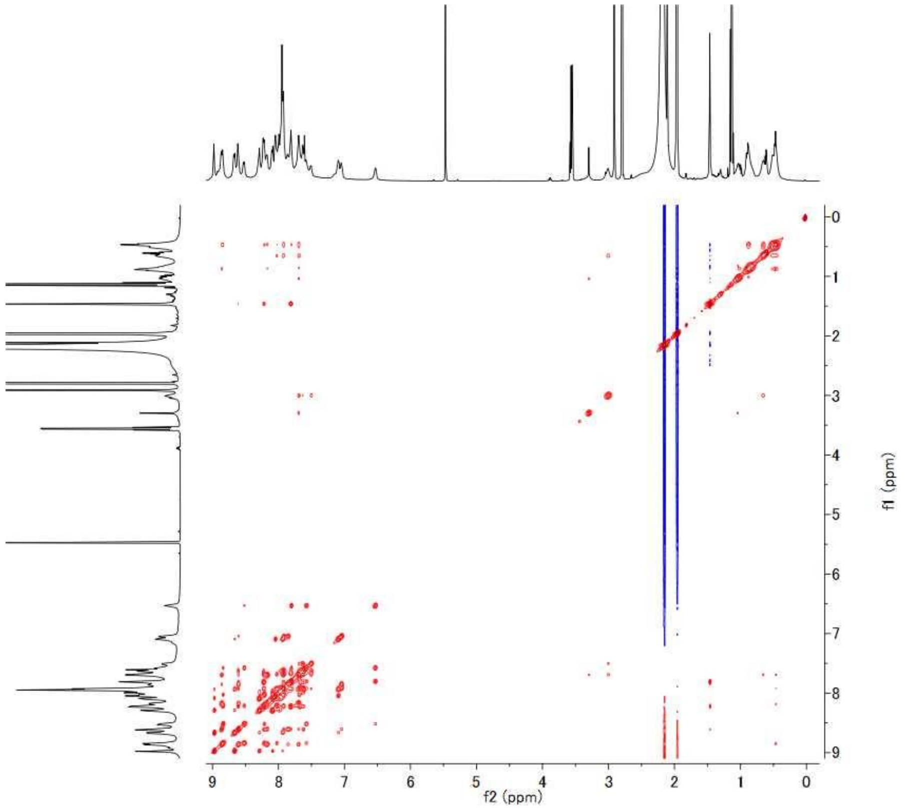

Supplementary Figure 102. 2D NOESY NMR (500 MHz, CD_3CN, 300 K) spectrum of complex **G3.**

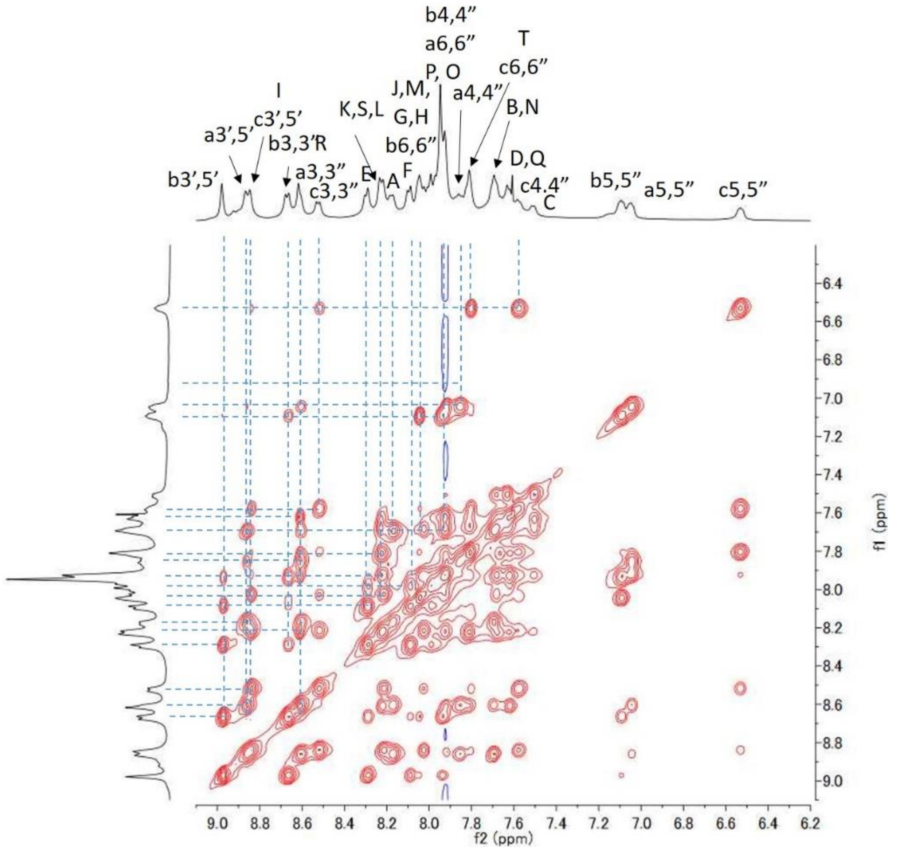

Supplementary Figure 103. 2D NOESY NMR (500 MHz, CD_3CN, 300 K) spectrum (aromic region) of complex **G3.**

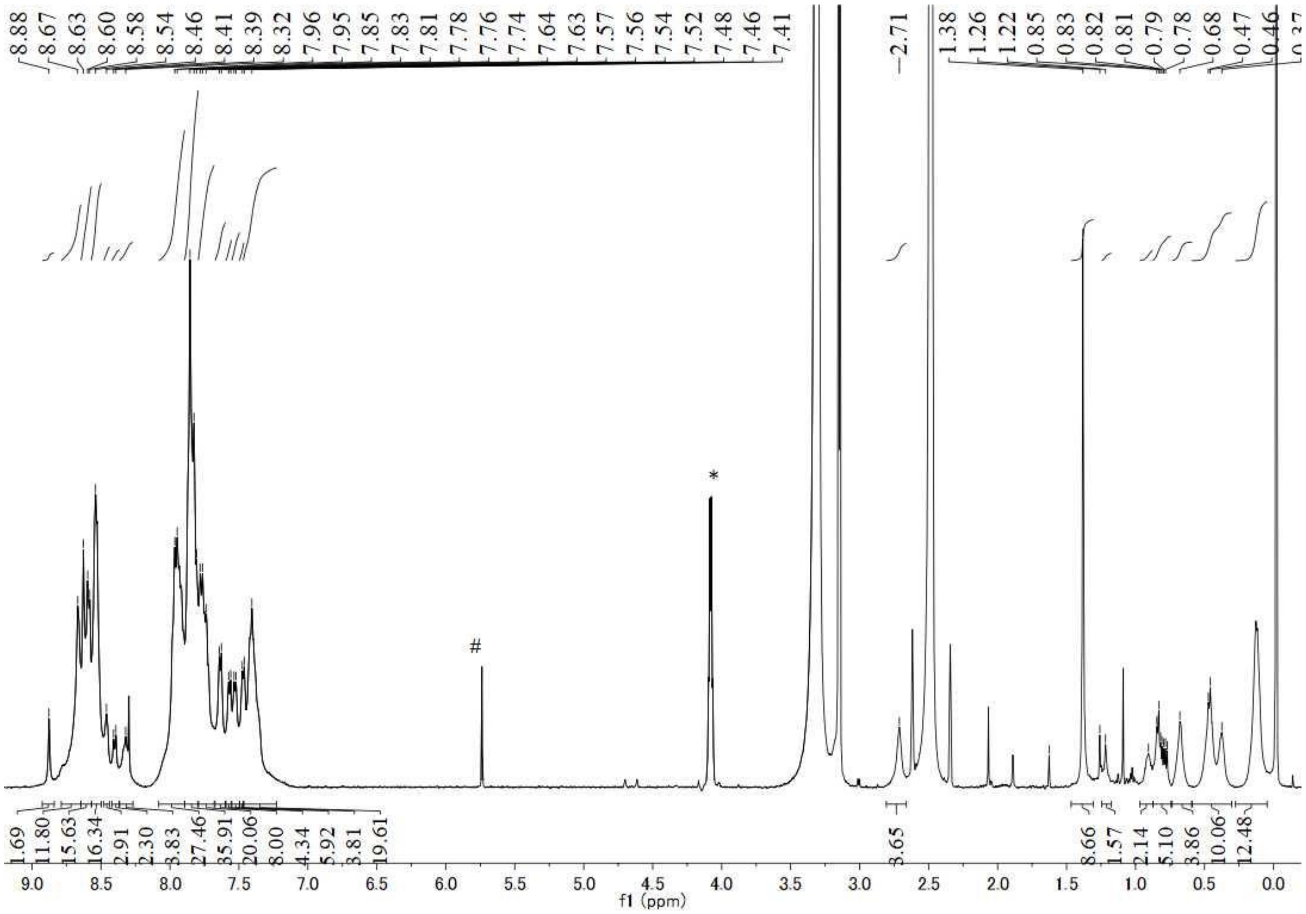

Supplementary Figure 104. ^{1}H NMR (500 MHz, DMSO-d_6, 300 K) spectrum of complex **G4**, # and * refer to the peak of DCM and methanol**.**

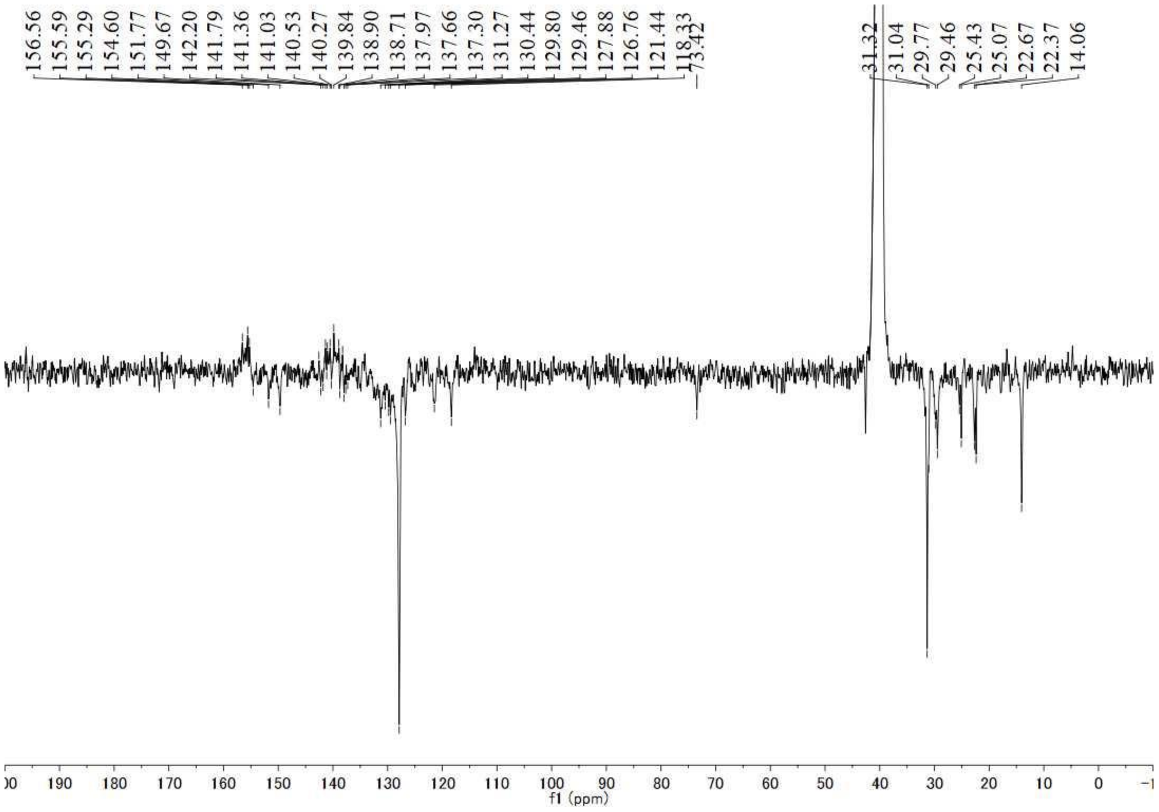

Supplementary Figure 105. ^{13}CDEPTQ NMR (125 MHz, DMSO-d_6, 300 K) spectrum of complex **G4.**

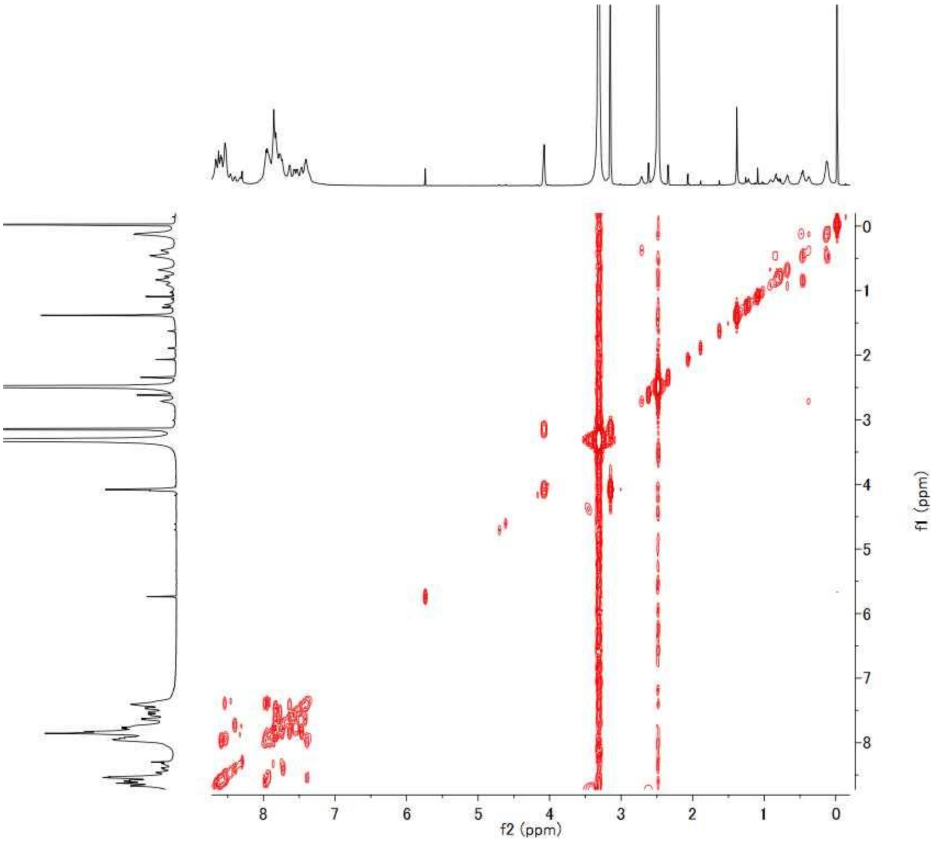

Supplementary Figure 106. 2D COSY NMR (500 MHz, DMSO-d_6, 300 K) spectrum of complex **G4.**

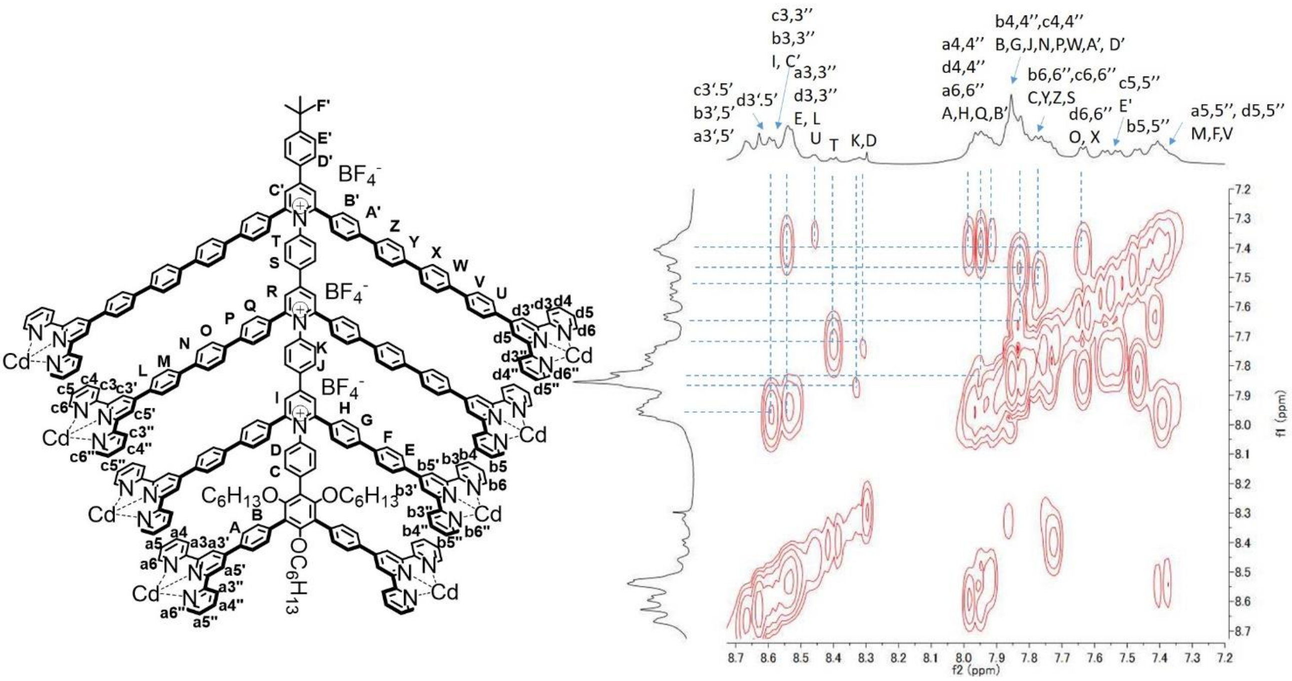

Supplementary Figure 107. 2D COSY NMR (500 MHz, DMSO-d_6, 300 K) spectrum (aromic region) of complex **G4.**

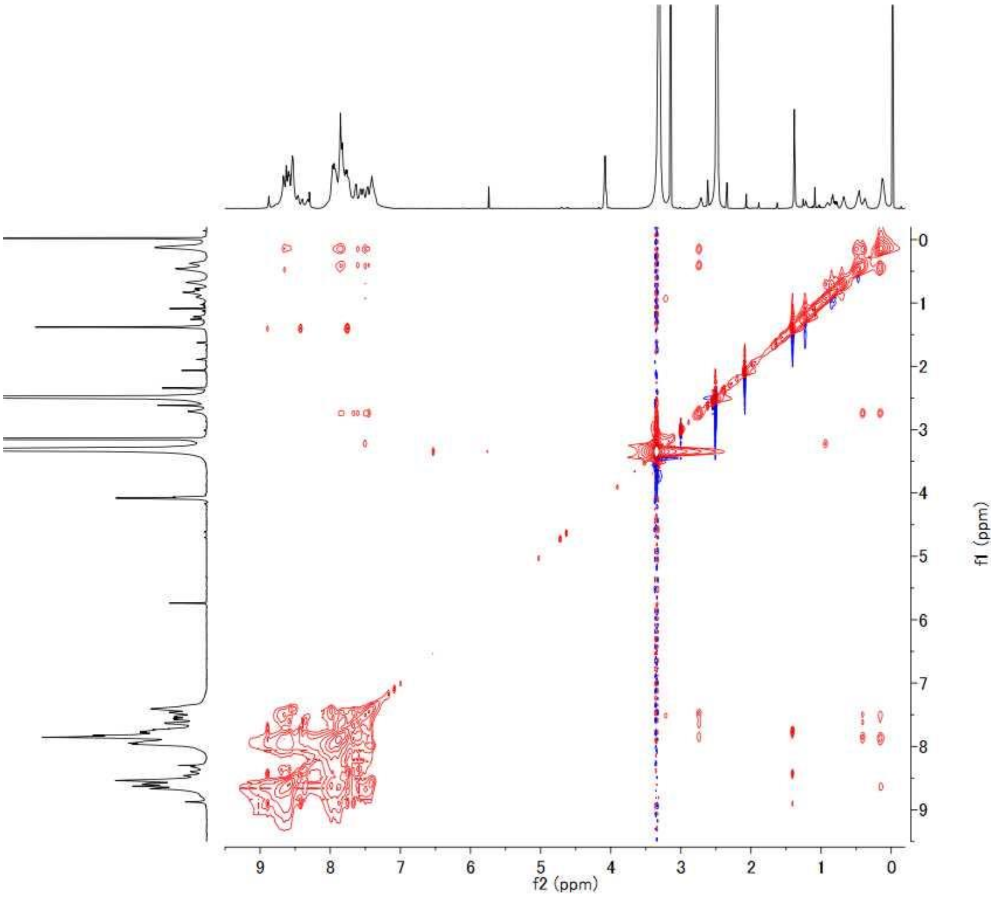

Supplementary Figure 108. 2D NOESY NMR (500 MHz, DMSO-d_6, 300 K) spectrum of complex **G4.**

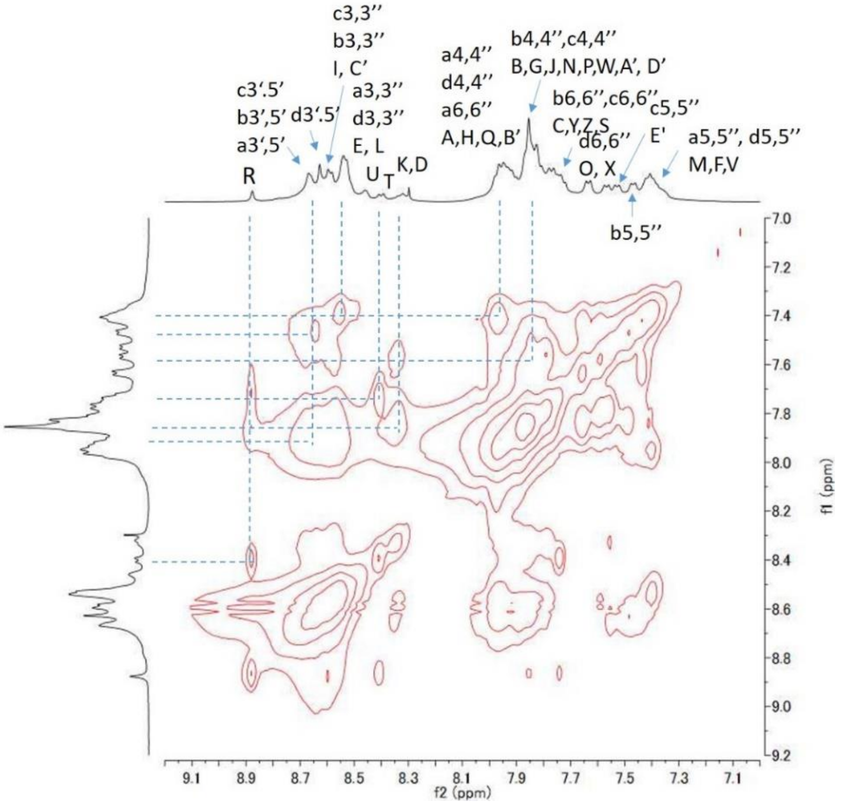

Supplementary Figure 109. 2D NOESY NMR (500 MHz, DMSO-d_6, 300 K) spectrum (aromic region) of complex **G4.**

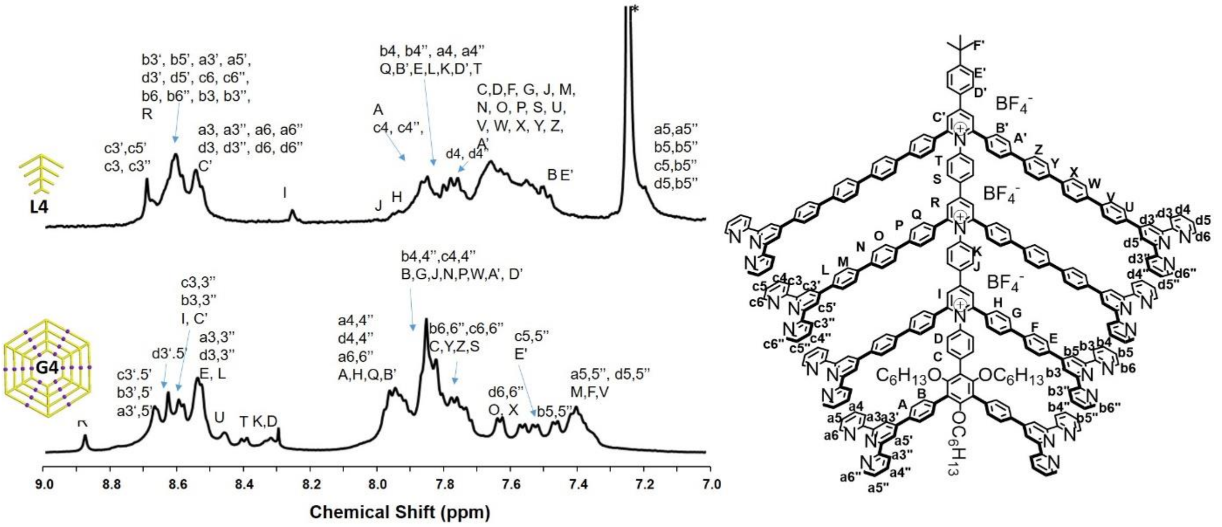

Supplementary Figure 110. ^{1}H NMR spectra of **L4** in $CDCl_3$ and **G4** in DMSO-d_6.

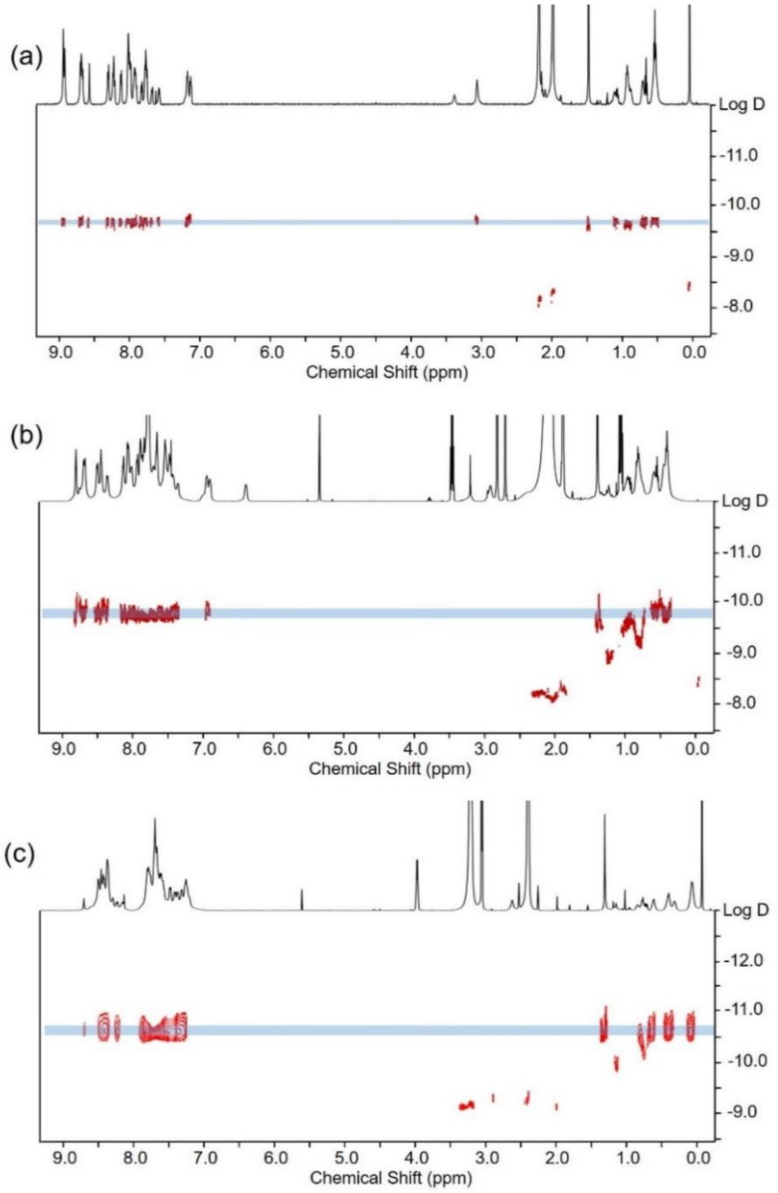

Supplementary Figure 111. 2D DOSY spectra of **G2** (a, 500 MHz, CD_3CN, 300 K), **G3** (b, 500 MHz, CD_3CN, 300 K) and **G4** (c, 500 MHz, DMSO-d_6, 300 K)

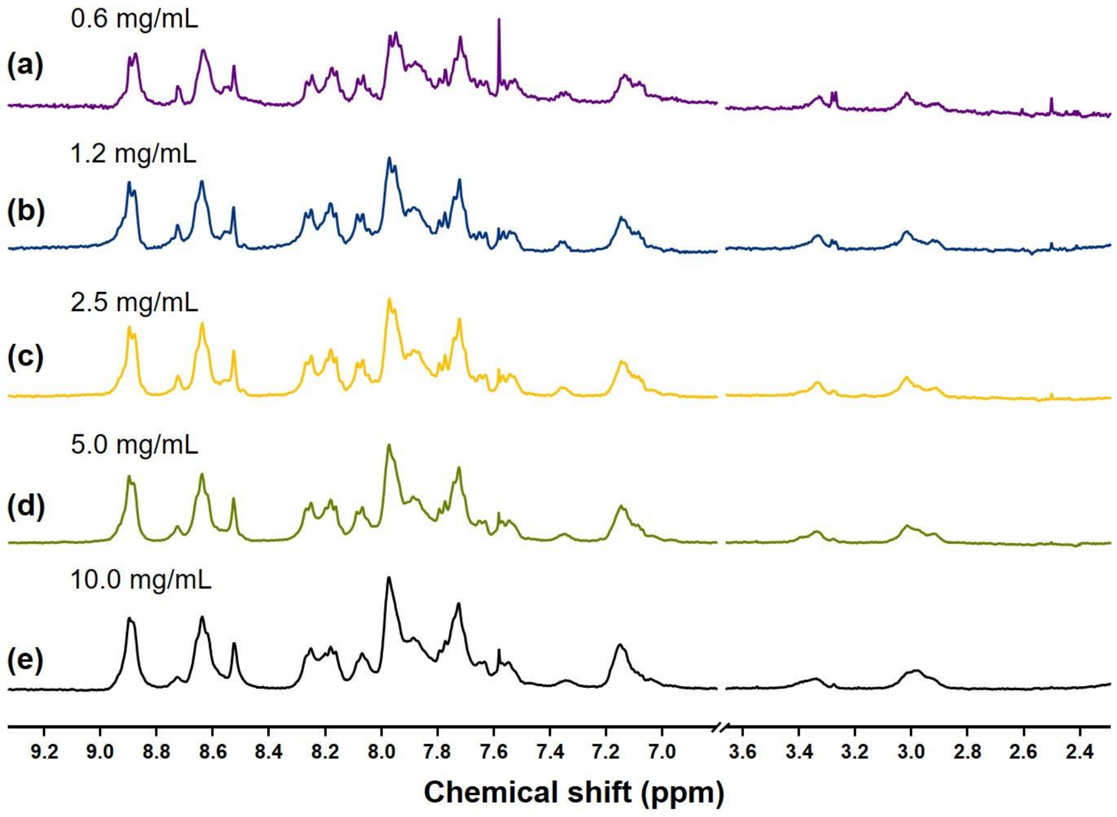

Supplementary Figure 112. Concentration dependent ^{1}H NMR (400 MHz, CD_3CN, 300 K) spectra of complex **G2**, (a) 0.6 mg/mL, (b) 1.2 mg/mL, (c) 2.5 mg/mL, (d) 5.0 mg/mL, (e) 10.0 mg/mL.

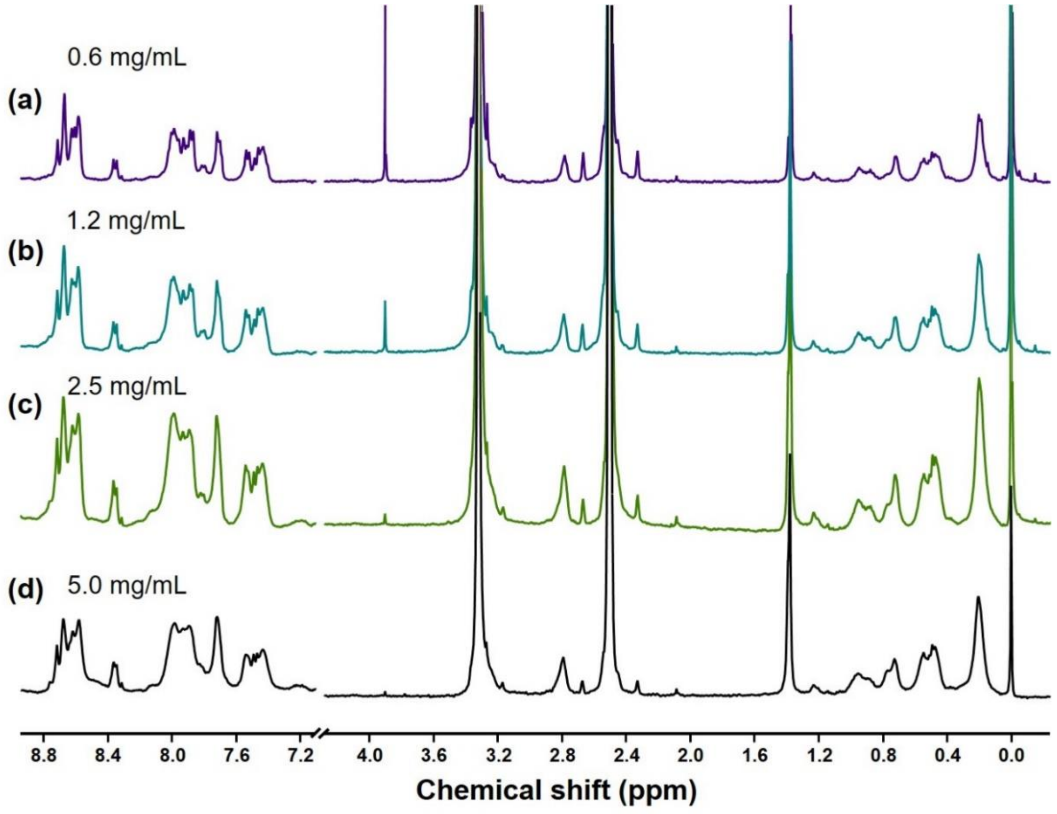

Supplementary Figure 113. Concentration dependent ^{1}H NMR (400 MHz, DMSO-d_6, 300 K) spectra of complex **G2**, (a) 0.6 mg/mL, (b) 1.2 mg/mL, (c) 2.5 mg/mL, (d) 5.0 mg/mL.

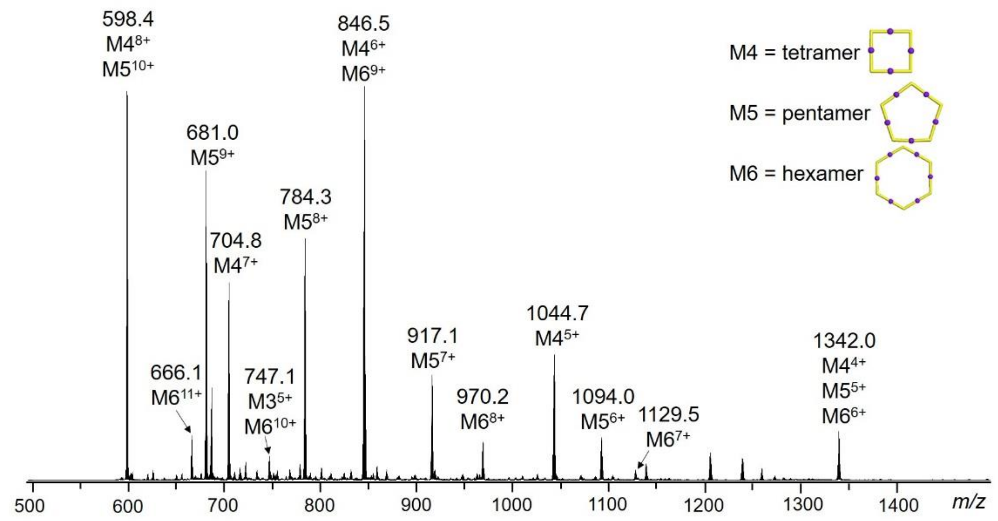

Supplementary Figure 114. ESI-MS spectrum of multiple macrocycles assembled by **L1** with Cd^{2+}.

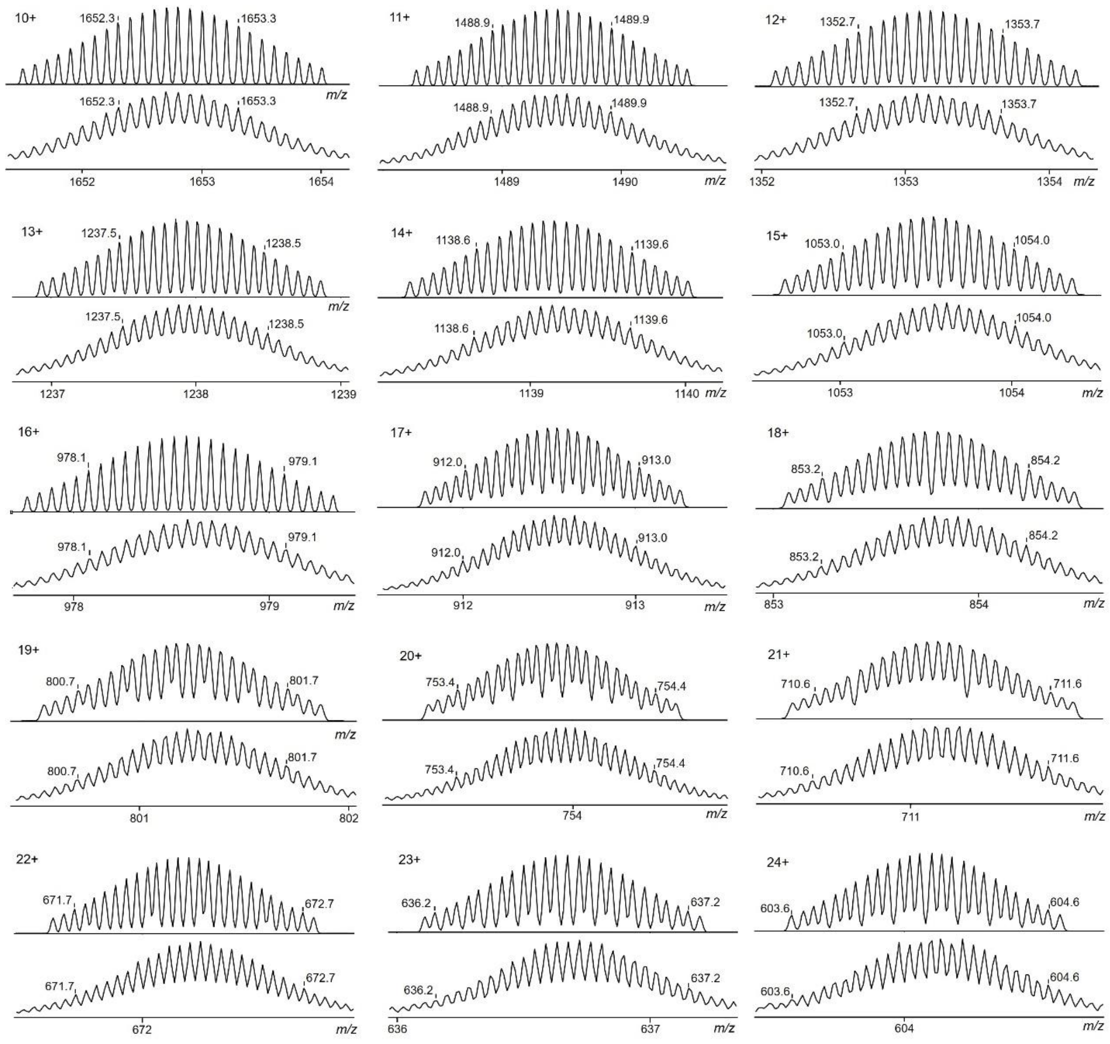

Supplementary Figure 115. Measured (bottom) and calculated (top) isotope patterns for different charge states observed from **G2** (PF_6^- as counterion).

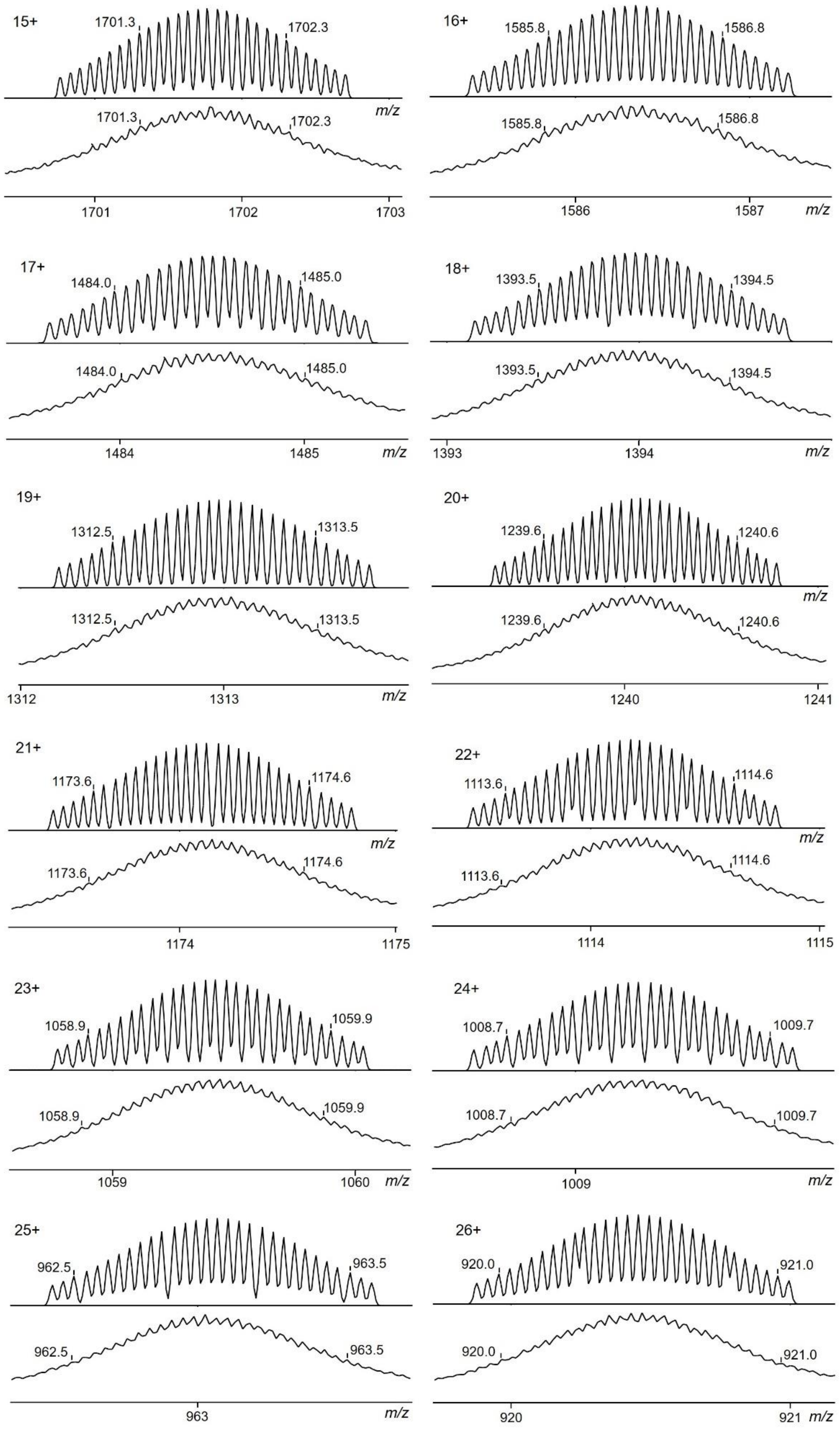

Supplementary Figure 116. Measured (bottom) and calculated (top) isotope patterns for different charge states observed from **G3** (PF_6^- as counterion).

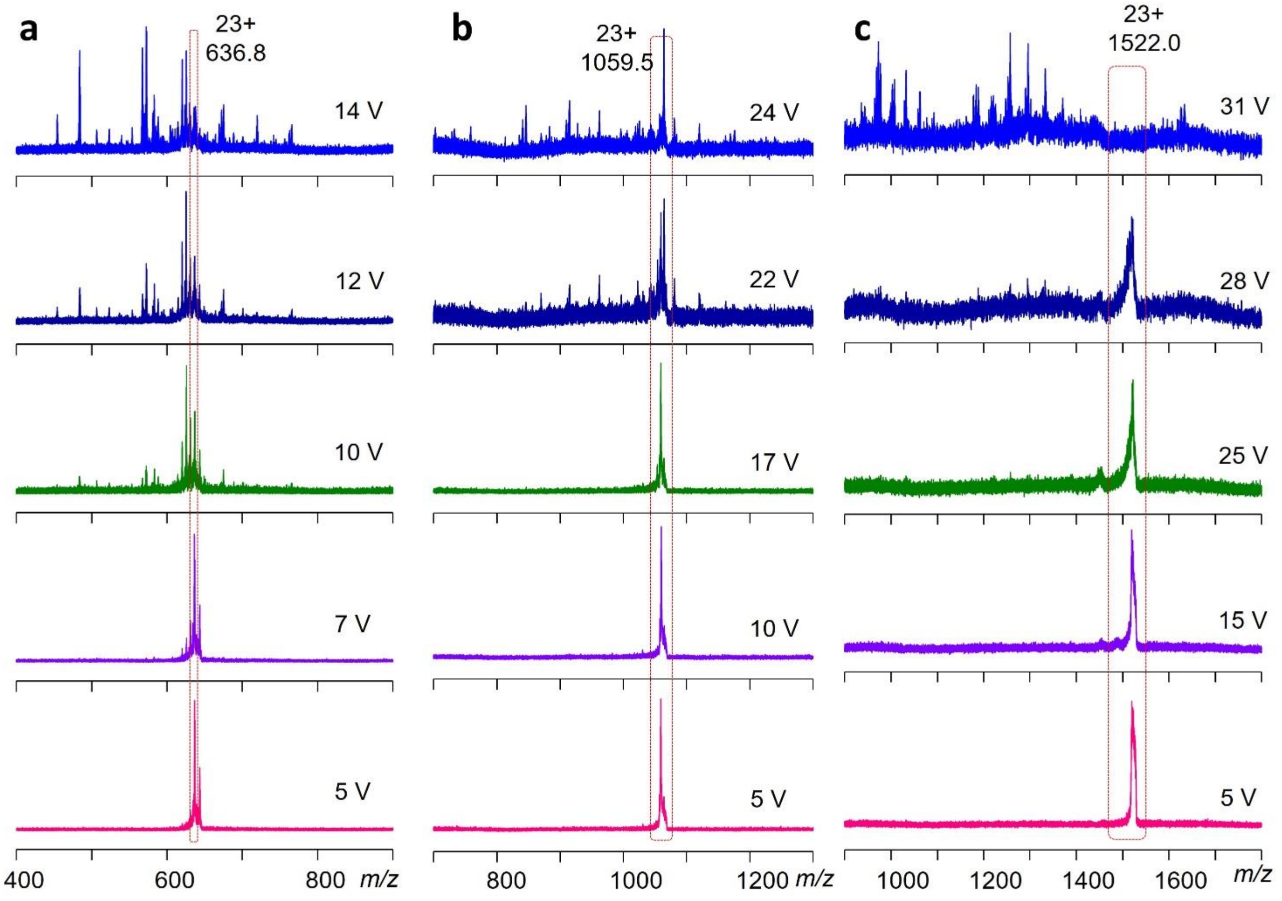

Supplementary Figure 117. gMS^2 of 23+ ions of **G2** (a, at *m/z* 636.8), **G3** (b, at *m/z* 1059.5) and **G4** (c, at *m/z* 1522.0) with various collision energies (from 5 V until total disassociation of the complexes).

6. Experimental and theoretical collision cross sections

Supplementary Table 1. Experimental and Theoretical Collision Cross Sections (CCSs).

	Drift times [ms]	CCS [Å²]	CCS (avg) [Å²]	CCS (calcd. avg) [Å²]
**G2**	6.39 (12+)	2486.6	2466.1 ± 15.0	2547.0 ± 36.9
	5.51 (13+)	2471.7		
	4.85 (14+)	2470.3		
	4.30 (15+)	2465.3		
	3.86 (16+)	2466.2		
	3.42 (17+)	2436.5		
**G3**	6.84 (17+)	3658.8	3659.8 ± 30.7	3727.7 ± 60.3
	6.17 (18+)	3652.8		
	5.62 (19+)	3652.1		
	5.07 (20+)	3619.6		
	4.74 (21+)	3653.5		
	4.52 (22+)	3722.3		
**G4**	6.95 (23+)	4994.8	5009.3 ± 30.1	5002.1 ± 78.8
	6.39 (24+)	4969.0		
	6.06 (25+)	5019.7		
	5.62 (26+)	4996.4		
	5.29 (27+)	5008.7		
	5.07 (28+)	5067.0		

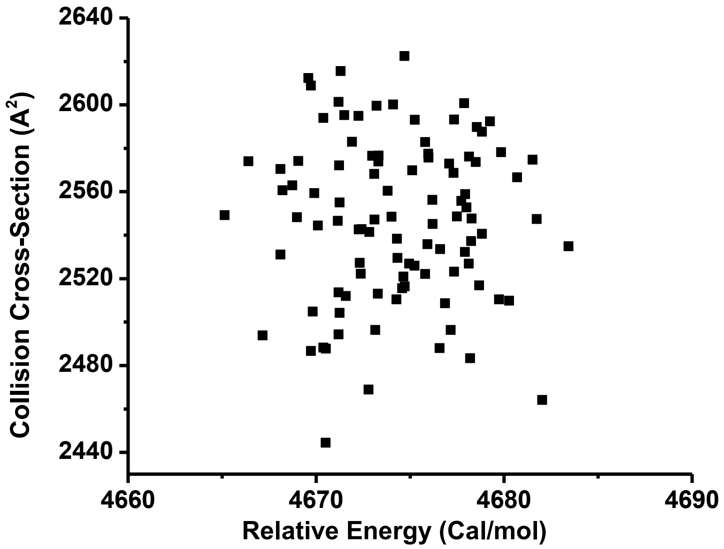

Supplementary Figure 118. Plot of collision cross-section (CCS) *vs.* relative energy for 100 candidate structures of **G2** generated by annealing simulations. CCSs were calculated by the TM method using the MOBCAL program. The average TM cross section area is 3727.7 ± 0.3 Å^2.

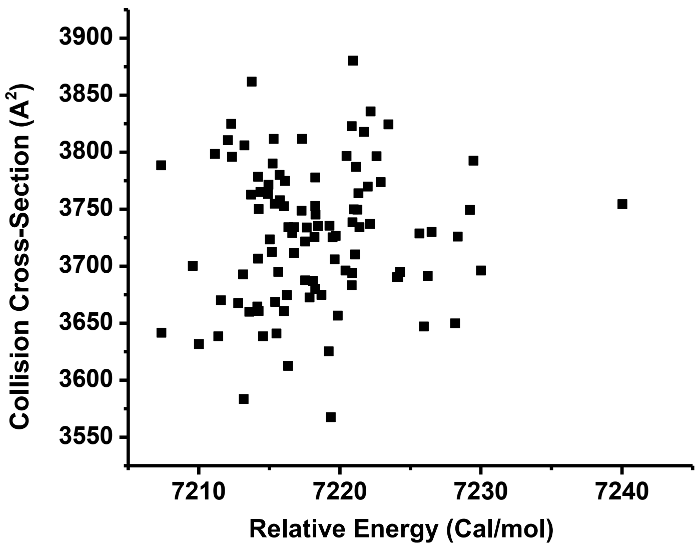

Supplementary Figure 119. Plot of collision cross-section (CCS) *vs.* relative energy for 100 candidate structures of **G3** generated by annealing simulations. CCSs were calculated by the TM method using the MOBCAL program. The average TM cross section area is 2547 ± 36.9 Å^2.

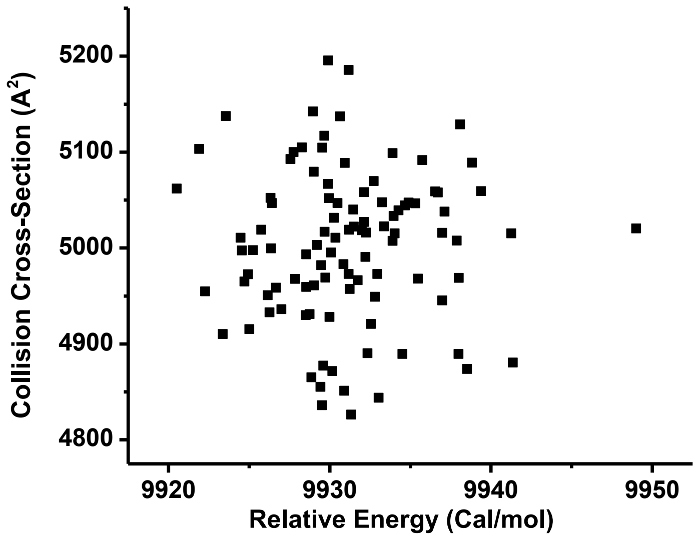

Supplementary Figure 120. Plot of collision cross-section (CCS) *vs.* relative energy for 100 candidate structures of **G4** generated by annealing simulations. CCSs were calculated by the TM method using the MOBCAL program. The average TM cross section area is 5002.1 ± 78.8 4Å^2.

7. AFM, TEM and STM images

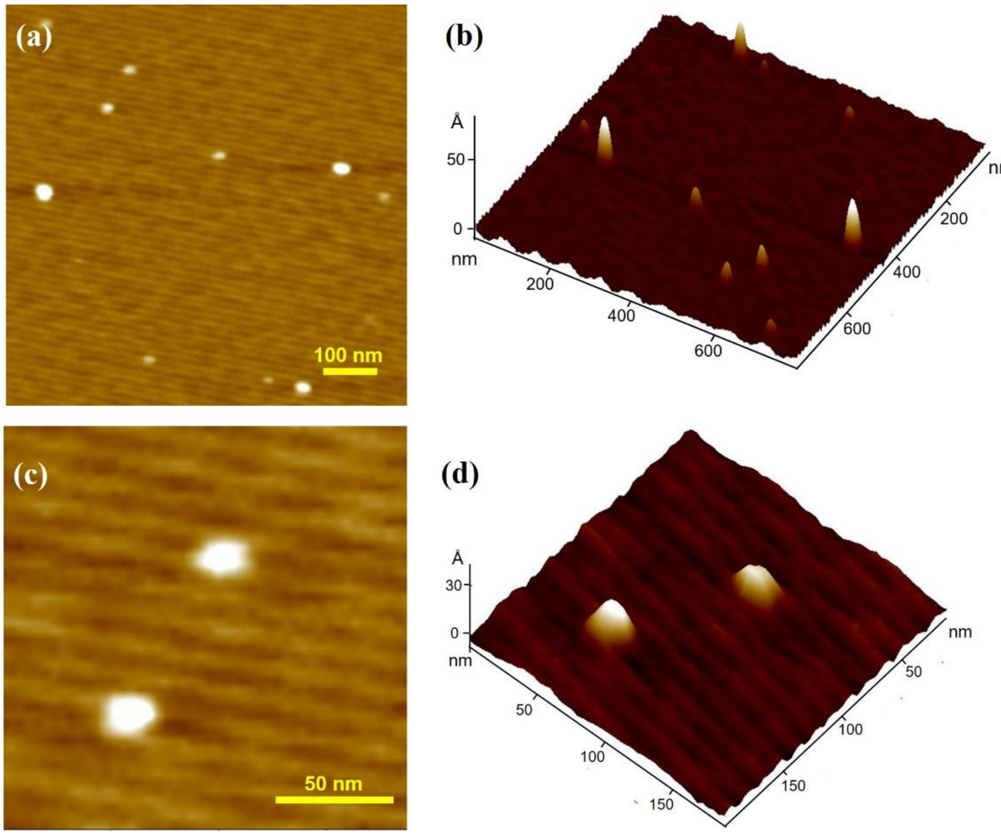

Supplementary Figure 121. AFM 2D (a, c) and 3D (b, d) images of complex **G2** on mica.

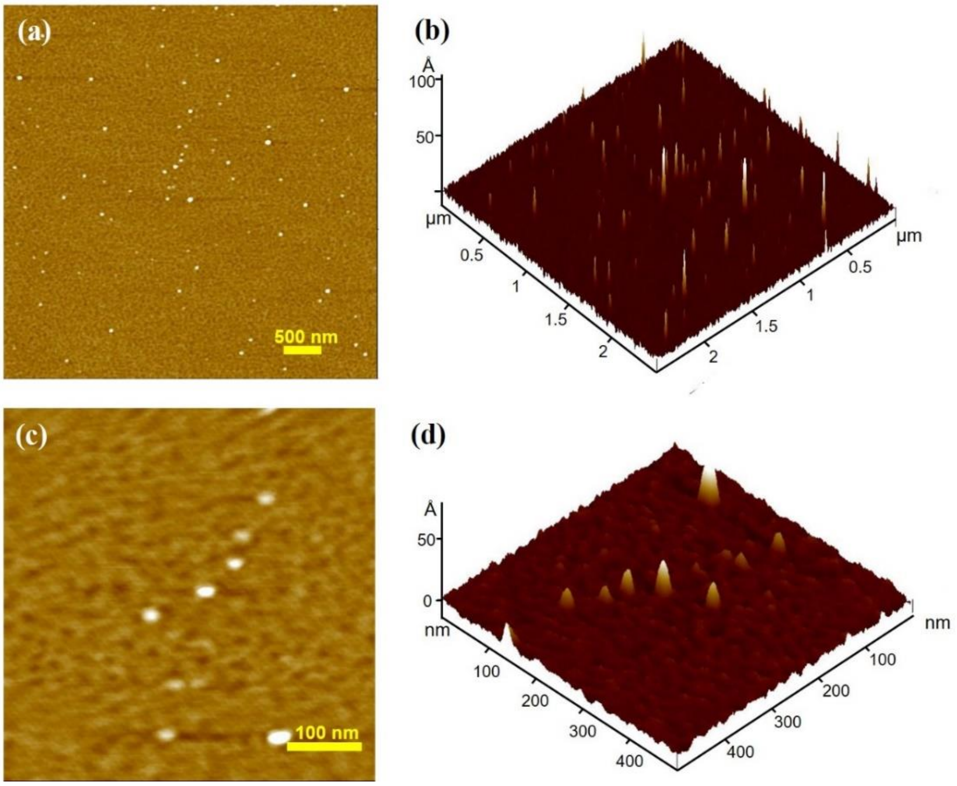

Supplementary Figure 122. AFM 2D (a, c) and 3D (b, d) images of complex **G3** on mica.

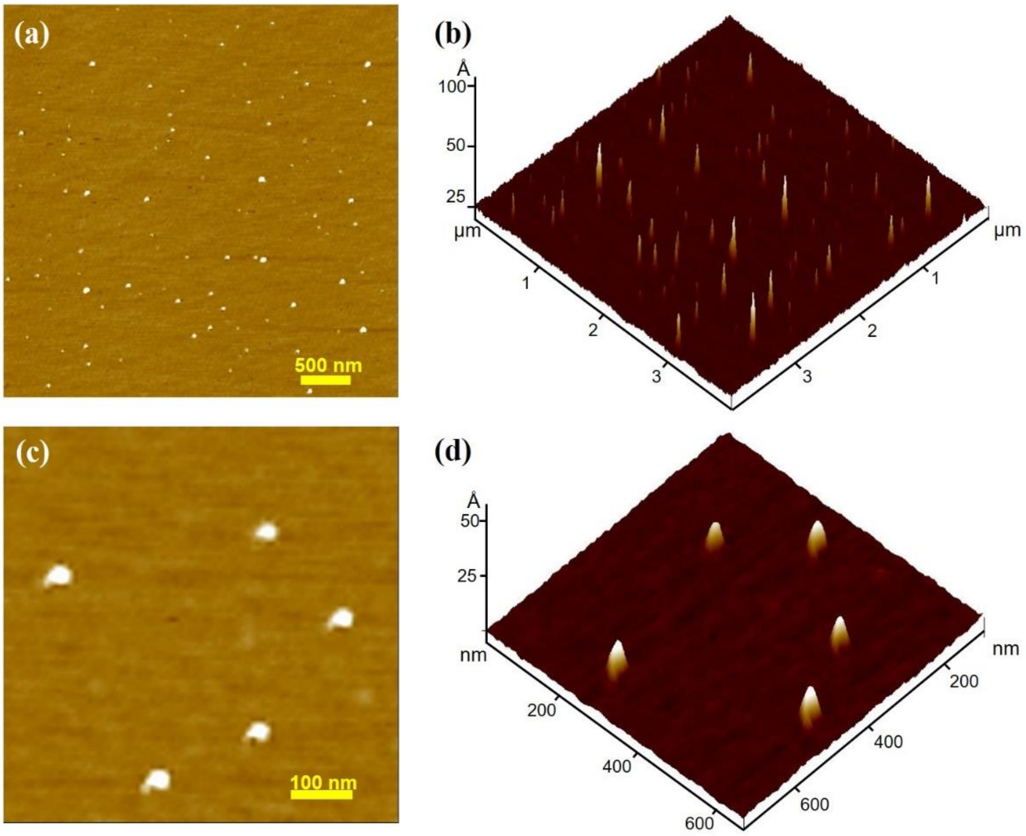

Supplementary Figure 123. AFM 2D (a, c) and 3D (b, d) images of complex **G4** on mica.

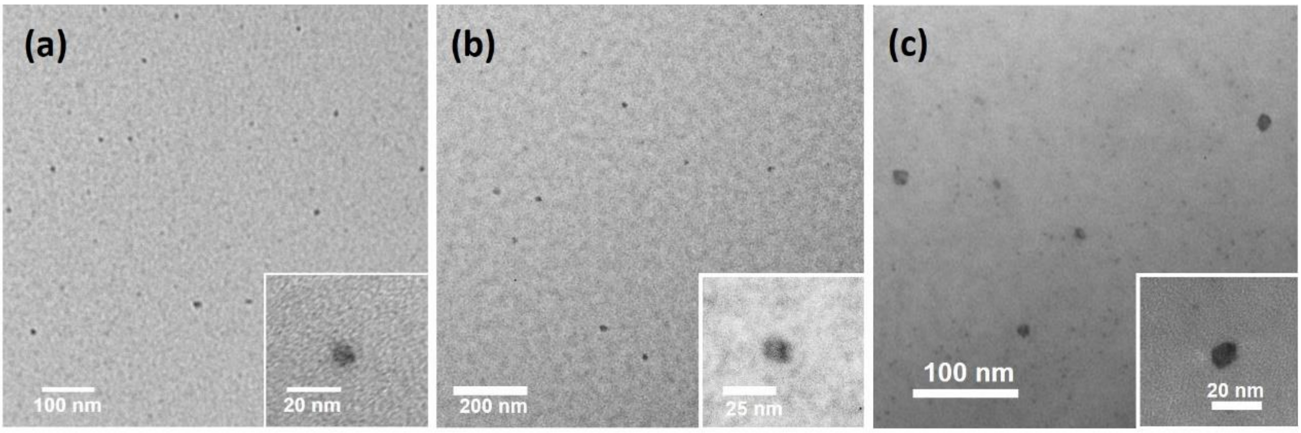

Supplementary Figure 124. TEM images of single molecular **G2** (a)**, G3** (b) and **G4** (c).

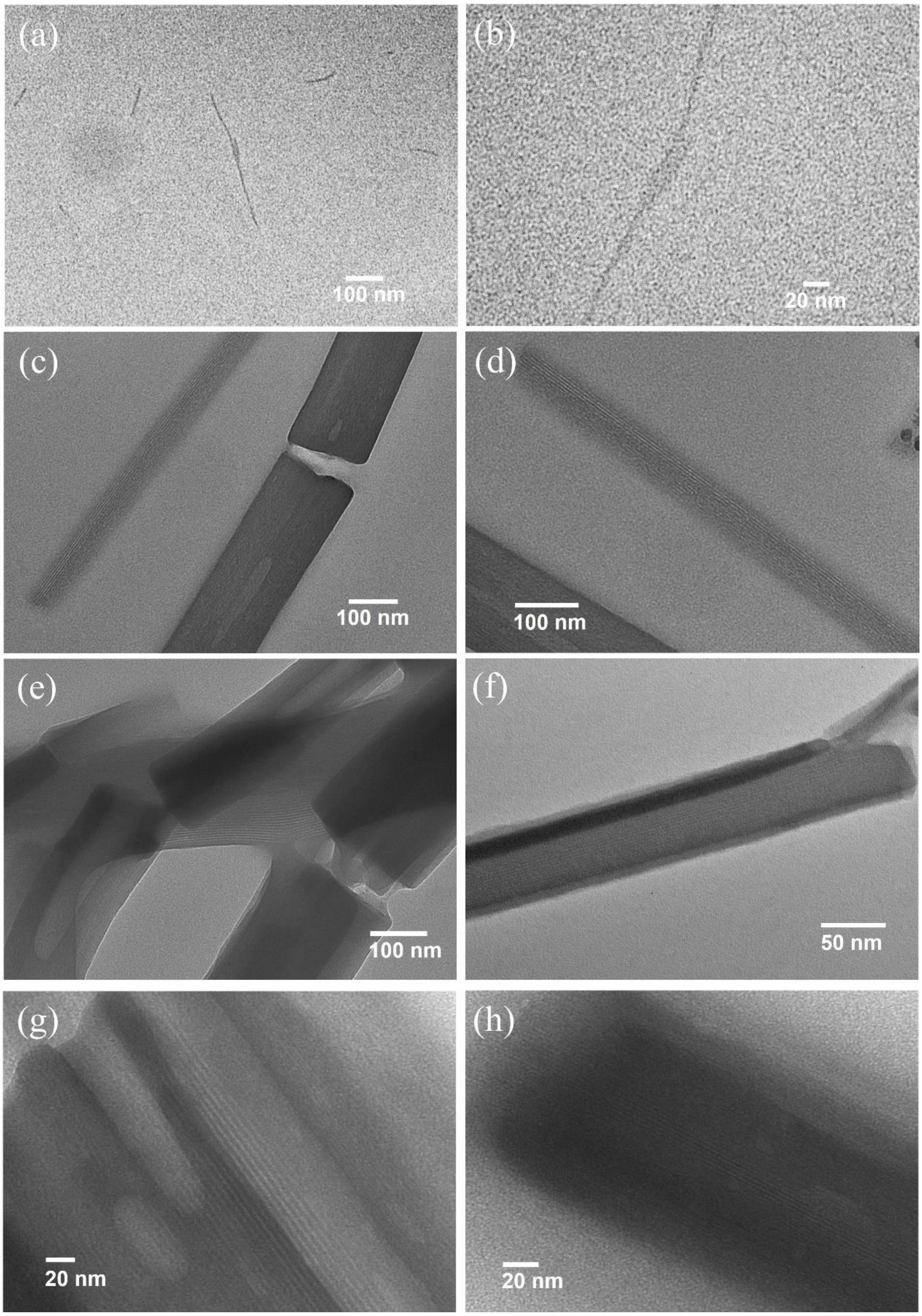

Supplementary Figure 125. TEM imaging of tubular-like nanostructures assembled by **G2**.

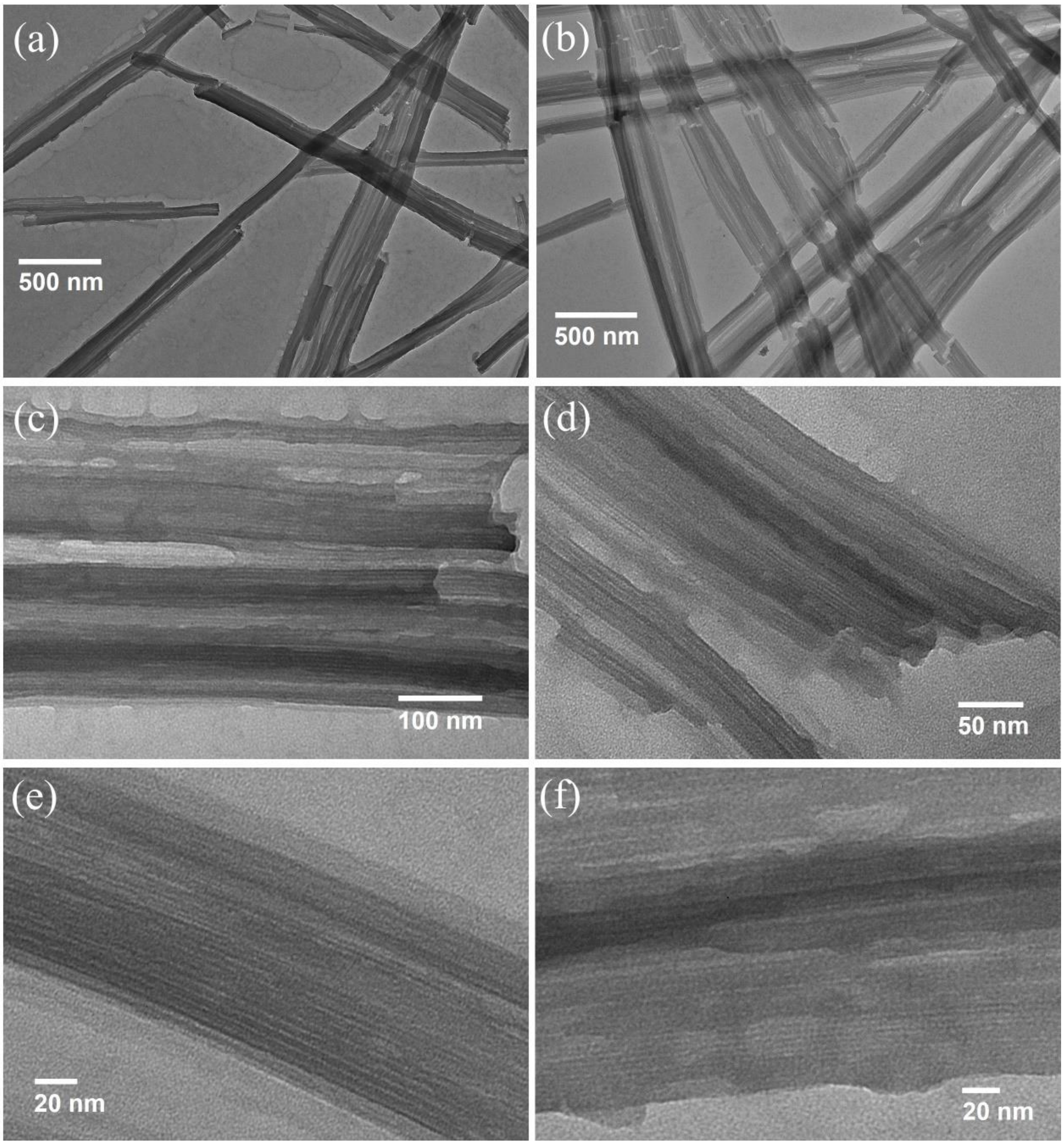

Supplementary Figure 126. TEM imaging of tubular-like nanostructures assembled by **G3**.

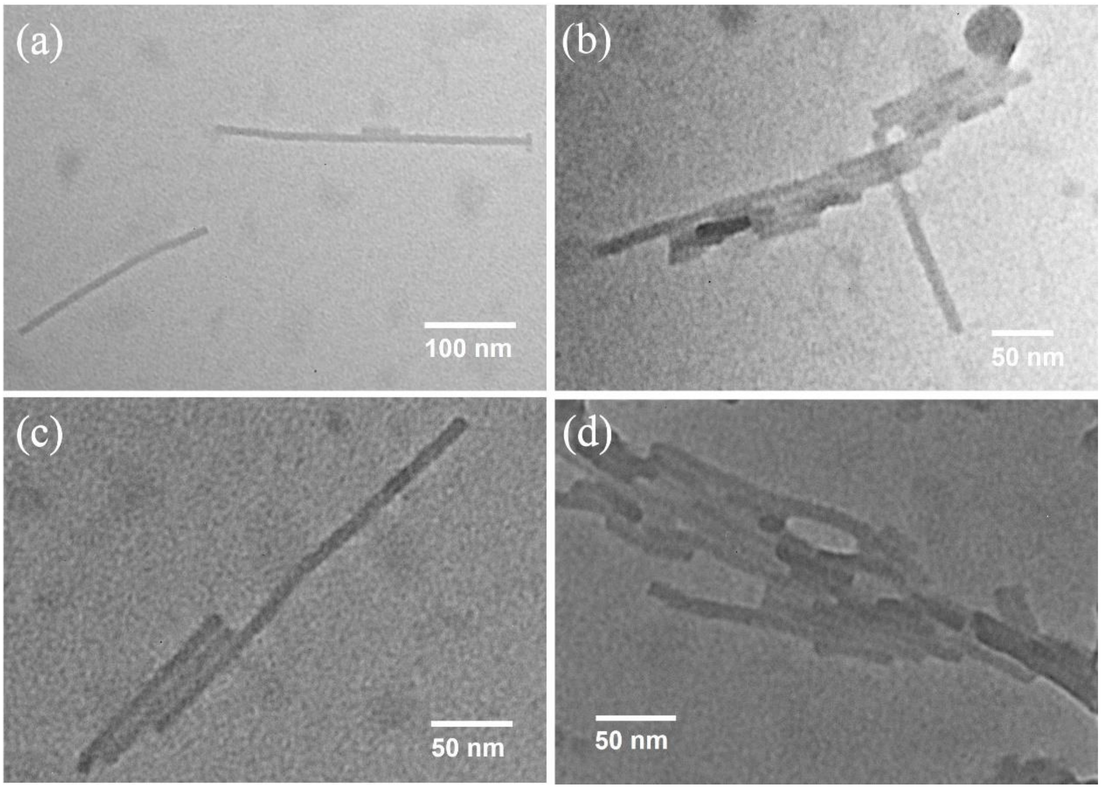

Supplementary Figure 127. TEM imaging of tubular-like nanostructures assembled by **G4**.

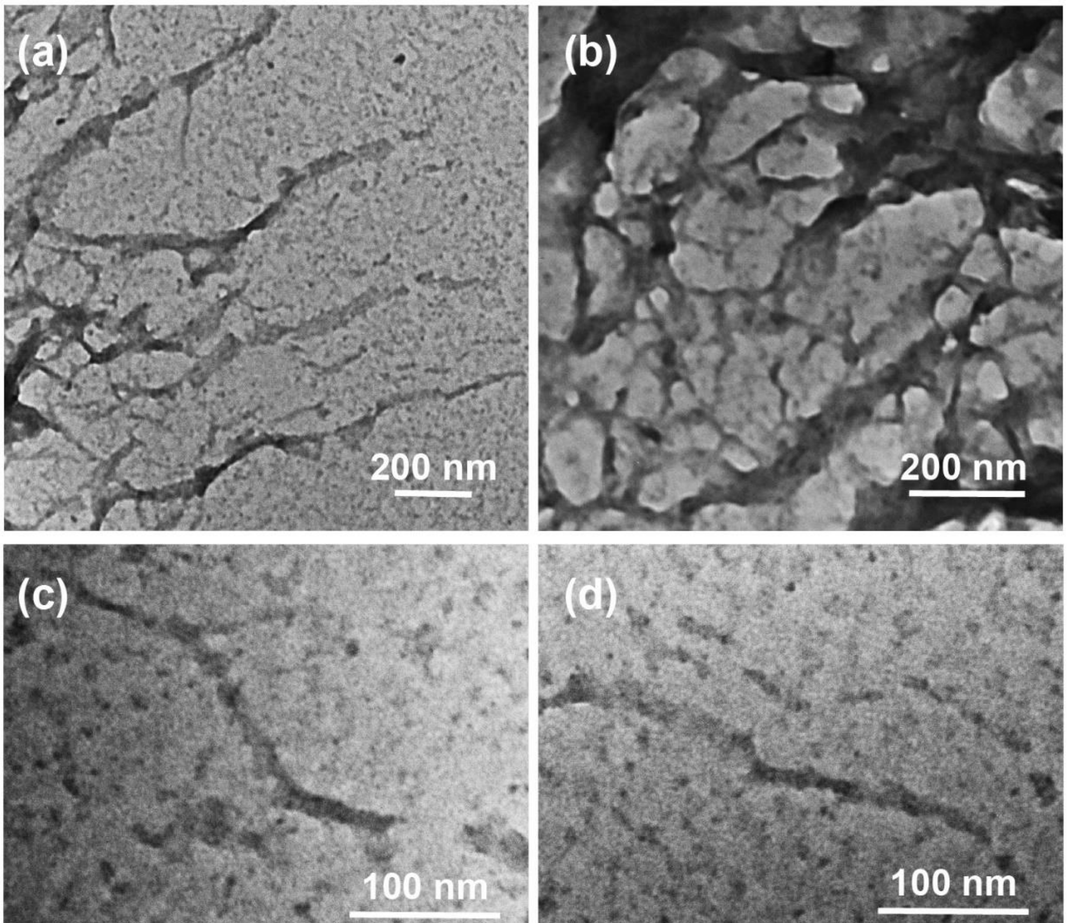

Supplementary Figure 128. TEM imaging of aggregation of **G2** in (a, b) DMSO/H_2O (v/v 1/2, 0.5 mg/mL), (c, d) DMSO/H_2O (v/v 1/2, 0.1 mg/mL).

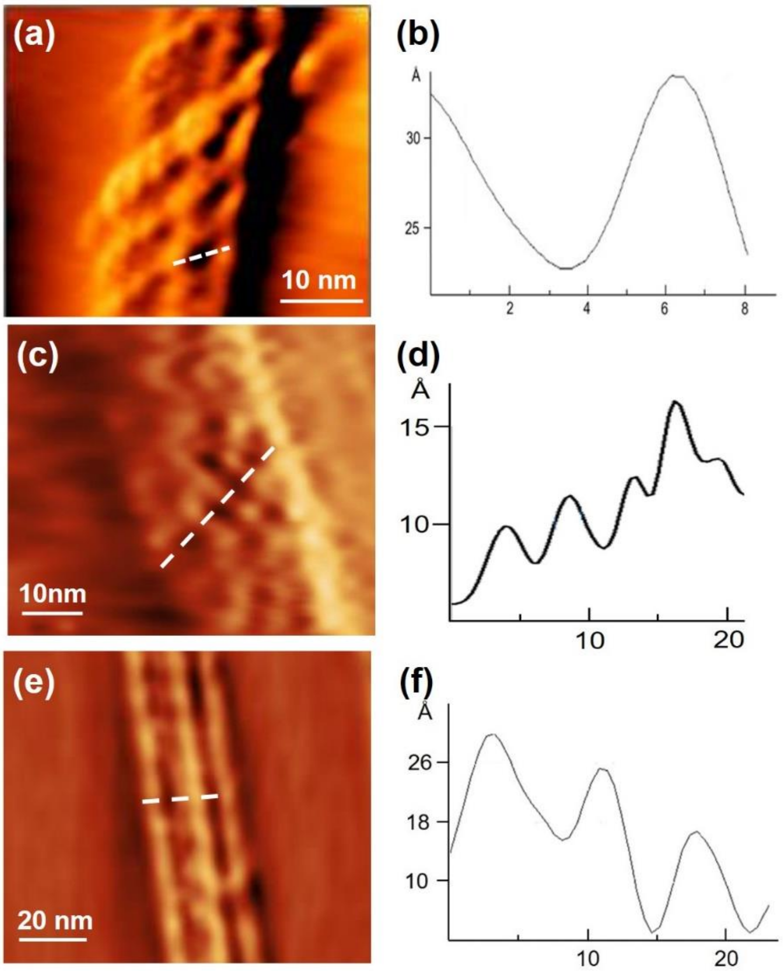

Supplementary Figure 129. STM images of supramolecular metal−organic nanoribbons assembled by **G2** (a, b), **G3** (c, d), and **G4** (e, f)

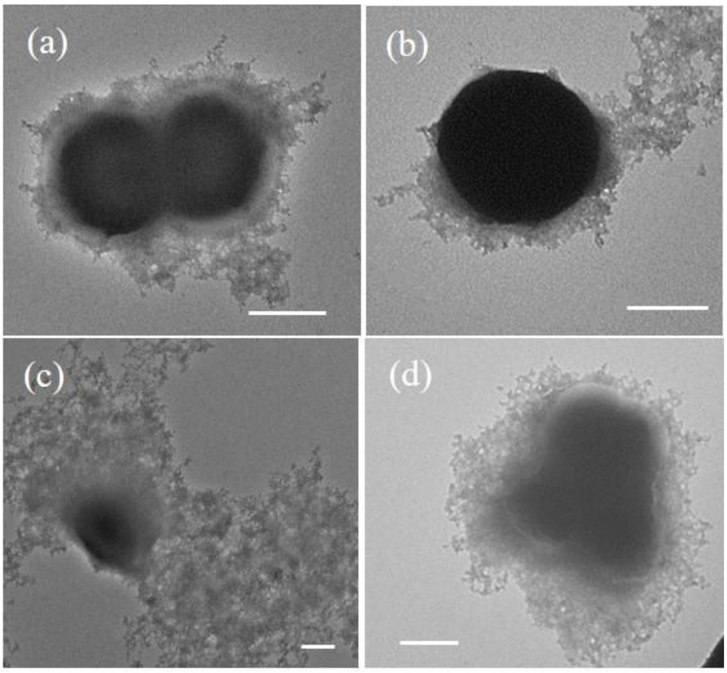

Supplementary Figure 130. TEM images of MRSA cells treated by **G3**, Scale bars: 500 nm.

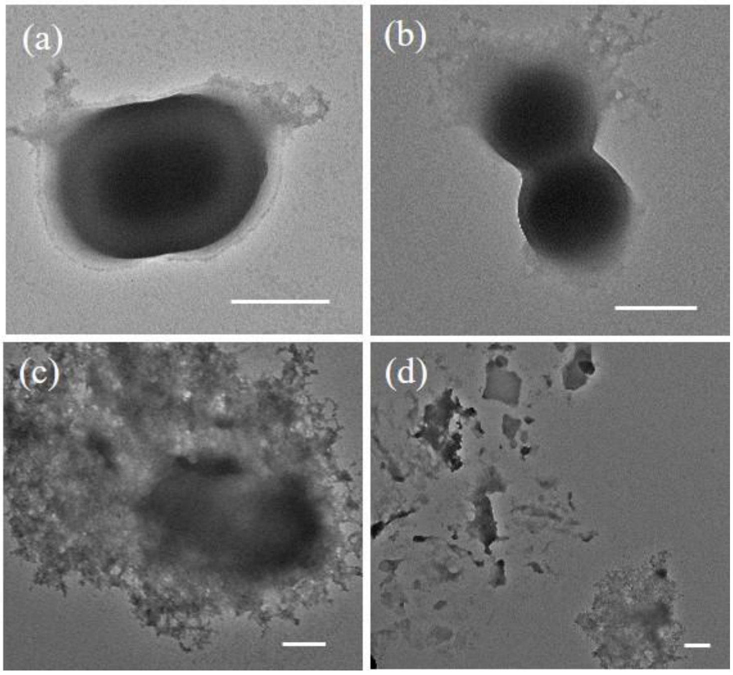

Supplementary Figure 131. TEM images of MRSA cells treated by **G4**, Scale bars: 500 nm.

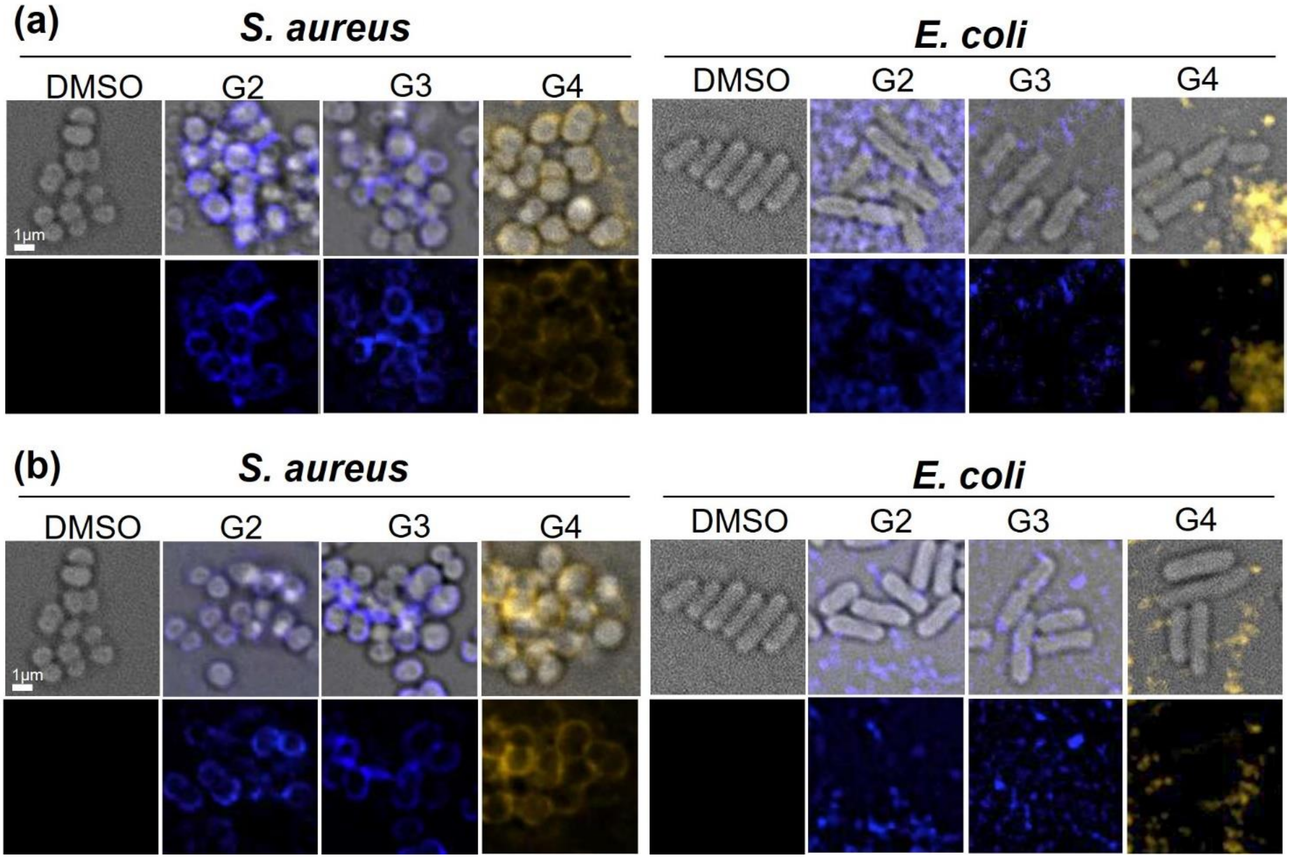

Supplementary Figure 132. 3D deconvolution fluorescence microscopy images of bacteria cells with and without treatment of 4 µM nested hexagons (a) and 2 µM complexes (b). No additional FM4-64 dye was added.

8. UV-Vis and florescence spectra of complexes G2−G4.

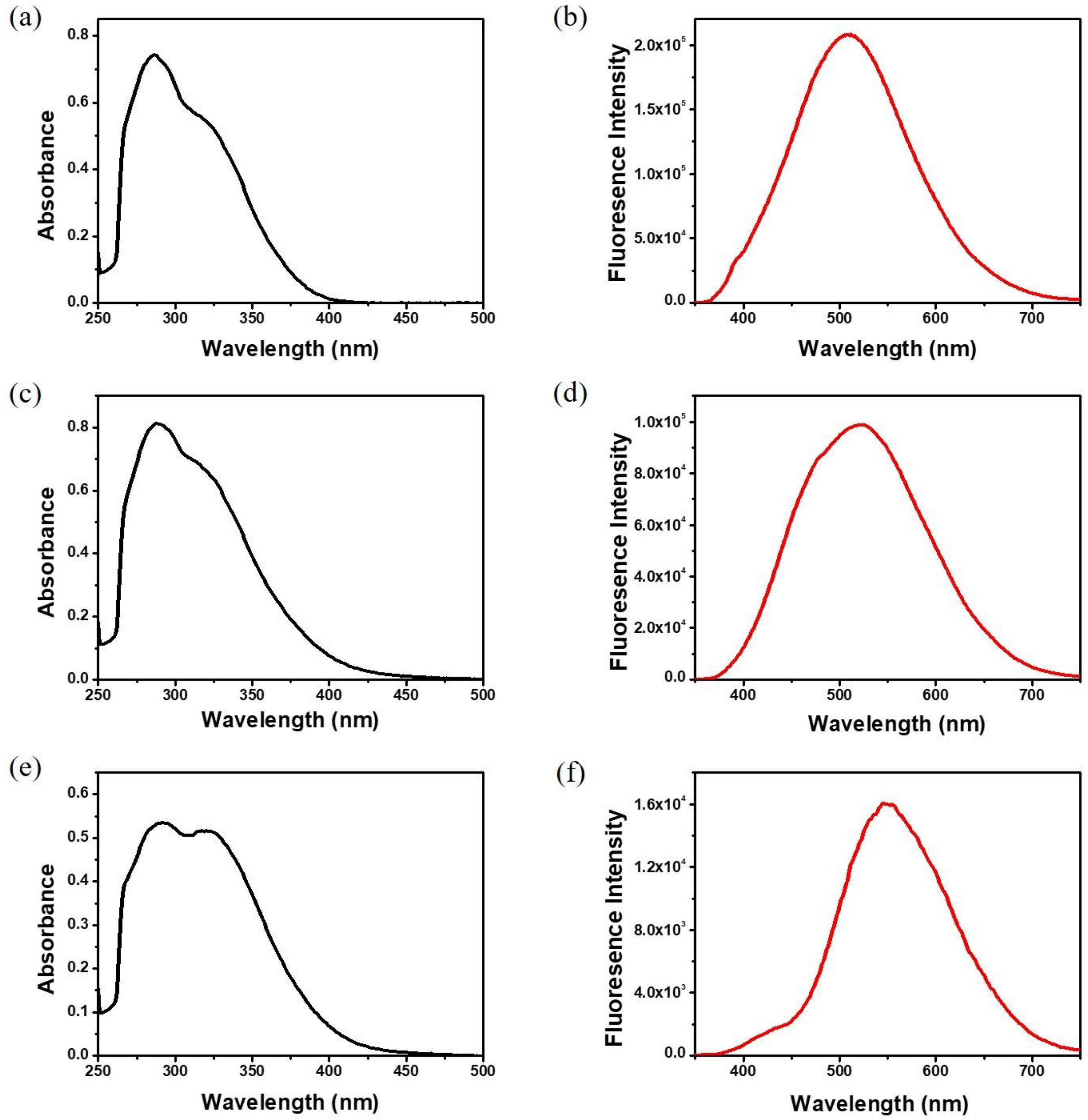

Supplementary Figure 133. UV-Vis spectra of **G2** (a, 20 μM in CH_3CN), **G3** (c, 15 μM in CH_3CN) and **G4** (e, 10 μM in DMSO) and florescence spectra of **G2** (b, 2 μM in CH_3CN, excited at 350 nm), **G3** (c, 1.5 μM in CH_3CN, excited at 350 nm) and **G4** (e, 1.0 μM in DMSO, excited at 350 nm).

9. Fitting Procedure to derive size parameter of G2−G4 from logD.[6,7]

(a)

G2											
Acetonitrile		kb	1.38E-23								
Oblate	b=b>a	p>1			p = b/a						
	a			b							
D		2.05E-10	log(D)	-9.68825							
T (K)	298										
η (Pa s)	0.000367							-9.93			
rh(sp) = kbT/6πDη		2.90E-09	Å								
	b	a	rh(sp)	rh(ob)	fs	Rcal	acal	bcal	p	1/p	rh/rcal
G2	4.00E-09	9.00E-10	2.90E-09	2.43E-09	1.192082	2.43E-09	4E-09	9E-10	4.444444	0.225	1.000067

(b)

G3											
Acetonitrile		kb	1.38E-23								
Oblate	b=b>a	p>1			p = b/a						
	a			b							
D		1.65E-10	log(D)	-9.78252							
T (K)	298										
η (Pa s)	0.000367							-9.93			
rh(sp) = kbT/6πDη		3.60E-09	Å								
	b	a	rh(sp)	rh(ob)	fs	Rcal	acal	bcal	p	1/p	rh/rcal
G3	5.10E-09	9.00E-10	3.60E-09	2.86E-09	1.259402	2.86E-09	5.1E-09	8.99E-10	5.666667	0.176471	1.000744

(c)

G4											
DMSO		kb	1.38E-23								
Oblate	b=b>a	p>1			p = b/a						
	a			b							
D		2.45E-11	log(D)	-10.6108							
T (K)	298										
η (Pa s)	0.00224							-9.93			
rh(sp) = kbT/6πDη		3.98E-09	Å								
	b	a	rh(sp)	rh(ob)	fs	Rcal	acal	bcal	p	1/p	rh/rcal
G4	5.70E-09	9.00E-10	3.98E-09	3.08E-09	1.293661	3.08E-09	5.69E-09	8.99E-10	6.333333	0.157895	1.001386

Supplementary Figure 134. Screenshot of Microsoft Excel spreadsheet used to fit **G2** (a) and **G3** (b) in CH_3CN, and **G4** (c) in DMSO using oblate spheroid model.

Spreadsheet details

- For sphere model, according to Stocks-Einstein equation, the hydrodynamic radius (r_h(sp)) of particles has the relationship with diffusion coefficients (D):

$$D = \frac{k_B T}{6\pi\eta r_h(sp)}$$

For non-sphere situation, the relationship between D and r_h(ob) is modified:

$$D = \frac{k_B T}{c f_s \pi\eta r_h(ob)}$$

Where:

$$c = \frac{6}{1 + 0.695(\frac{r_{vdW}}{r_h})^{2.234}}$$

r_{vdW} is the van der Waals radii of the solvent, acetonitrile, which is far less than r_h of **G2-G4**. As a result, $c \approx 6$. "f_s" is the frictional coefficient, and it is derived from a and b for the oblate spheroid model (b = b > a) with the formula:

$$f_s = \frac{\sqrt{(\frac{b}{a})^2 - 1}}{(\frac{b}{a})^{\frac{2}{3}} \tan^{-1}\sqrt{(\frac{b}{a})^2 - 1}}$$

As a result:

$$r_h(ob) = \frac{r_h(sp)}{f_s}$$

- Values in the red boxes (b and a in meter) are the only ones iteratively changed
- "k_b" is the Boltzmann constant, "T" is the experimental temperature (298 K), and "η" is the viscosity of CD_3CN (0.000367 Pa s)
- "a" and "b" are the semiminor and semimajor axis of the oblate spheroid. "*p*" is the aspect ratio (*p* = b/a)
- "*r*(cal)" is the radius of a sphere with an equivalent volume as the spheroid generated by a and b; since spheroid volume (V) has the relationship with a and b: $\mathrm{V} = \frac{4}{3}\pi ab^2$, and $r(\mathrm{cal}) = \sqrt[3]{\frac{3V}{4\pi}}$, *r*(cal) can be calculated by the formula: $r(\mathrm{cal}) = \sqrt[3]{ab^2}$
- "r_h(ob)/*r*(cal)" is the ratio of the hydrodynamic radius generated from the oblate model and the equivalent radius generated by the volume of the spheroid; this ratio was used to guide the adjustment of a and b until the volume generated by them matches the volume generated by r_h(ob) (r_h(ob)/*r*(cal) = 1)

10. Supplementary References

1. Thalassinos, K. et al. Characterization of phosphorylated peptides using traveling wave-based and drift cell ion mobility mass spectrometry. *Anal. Chem.* **81**, 248-254 (2008).
2. http://www.indiana.edu/~clemmer/Research/Cross%20Section%20Database/cs_database.php.
3. Horcas, I. et al. WSXM: a software for scanning probe microscopy and a tool for nanotechnology. *Rev. Sci. Instrum.* **78**, 013705 (2007).
4. Li, Y. et al. Helical antimicrobial sulfono-γ-AApeptides. *J. Med. Chem.* **58**, 4802-4811 (2015).
5. Wenzel, M. et al. Small cationic antimicrobial peptides delocalize peripheral membrane proteins. *Proc. Natl. Acad. Sci. U.S.A.* **111**, E1409-E1418 (2014).
6. Giuseppone, N., Schmitt, J.-L., Allouche, L. & Lehn, J.-M. DOSY NMR experiments as a tool for the analysis of constitutional and motional dynamic processes: implementation for the driven evolution of dynamic combinatorial libraries of helical strands. *Angew. Chem. Int. Ed.* **47**, 2235-2239 (2008).
7. Schulze, B. M., Watkins, D. L., Zhang, J., Ghiviriga, I. & Castellano, R. K. Estimating the shape and size of supramolecular assemblies by variable temperature diffusion ordered spectroscopy. *Org. Biomol. Chem.* **12**, 7932-7936 (2014).
